# Application of a high-density microelectrode array assay using a 3D human iPSC-derived brain microphysiological system model for in vitro neurotoxicity screening of environmental compounds

**DOI:** 10.1007/s00204-025-04043-x

**Published:** 2025-04-28

**Authors:** Kelly E. Carstens, Elena Gronskaya, David Jäckel, Jessica Bertoli, Kelvin Ramirez Cuevas, Julien Dorier, Shan Wang, David Lopez-Rodriguez, Timothy J. Shafer, Marie-Gabrielle Zurich, David Pamies

**Affiliations:** 1 Center for Computational Toxicology and Exposure, USA Environmental Protection Agency, Research Triangle Park, North Carolina, NC 27707 USA; 2 MaxWell Biosystems AG, Zurich, Switzerland; 3 Department of Biomedical Sciences, University of Lausanne, Lausanne, Switzerland; 4 Bioinformatics Competence Center, University of Lausanne, CH- 1015 Lausanne, Switzerland; 5 Bioinformatics Competence Center, Ecole Polytechnique Fédérale de Lausanne, CH- 1015 Lausanne, Switzerland; 6 Swiss Centre for Applied Human Toxicology (SCAHT), Basel, Switzerland; 7 Institute of Earth Surface Dynamics, University of Lausanne, Canton de Vaud, Lausanne, Switzerland; 8 Stem Cells and Organoid Facility, University of Lausanne, Rue du Bugnon 9, CH-1005 Lausanne, Switzerland

**Keywords:** Neurotoxicology, Neuronal electrical activity, Microelectrode array, MEA, High-density electrode array, iPSC, MPS, 3D cultures, BrainSphere

## Abstract

Unraveling the associations between human exposure to environmental chemicals and potential neurotoxicity presents significant challenges. Evaluation of neurotoxicity potential using animal testing is resource-intensive (financial, labor, and animal use) and faces uncertainties regarding biological relevance to human health outcomes. Therefore, there is a need to develop efficient and human-relevant in vitro new approach methodologies (NAMs) to screen and evaluate chemicals for neurotoxicity potential. Recording of neural network activity using microelectrode array (MEA) technology has been identified as a reliable and reproducible method for evaluating neurotoxicity. Much of this research has been performed in 2D rodent-derived cell models. The ‘BrainSpheres MEA assay’ described in this study offers a promising functional human induced pluripotent stem cell (iPSC)-derived 3D brain model comprising neurons, astrocytes, and oligodendrocytes. We demonstrate consistent spontaneous neuronal firing and network bursting parameters from 7-week-old BrainSpheres using a high-density MEA technology. The performance of this model as a human-relevant NAM was evaluated by conducting a multi-concentration, 13 day exposure study with a set of ten chemicals. Neural activity metrics were assessed and compared to results from a 2D-MEA assay using rodent cells. Loperamide and domoic acid (two assay positive controls) demonstrated similar bioactivity profiles in the BrainSphere MEA assay to the 2D-MEA assay, while acetaminophen (assay negative control) was inactive in both assays. The 2D-MEA model demonstrated more potent bioactivity for 4/7 chemicals that were active in both assays. In the future, reducing replicate variability and testing a larger set of chemicals will likely improve the accuracy and reliability of the assay. These preliminary findings suggest that the BrainSphere assay could be used alongside the rat network formation assay (rNFA) as part of a tiered strategy, where hits in the rNFA are confirmed and further characterized in the BrainSphere model, helping move toward animal-free toxicological testing.

**Supplementary Information:**

The online version contains supplementary material available at 10.1007/s00204-025-04043-x.

## Introduction

Environmental exposure to chemicals has been associated with many chronic, neurodevelopmental and neurodegenerative diseases (Sears and Genuis, 2012, Landrigan et al., 2016). Identifying links between human exposure and chemical toxicity remains challenging due to limitations such as exposure uncertainties, study covariates, and human variability. Considering the tremendous social and economic impacts of neurodevelopmental and neurodegenerative diseases, the public and regulatory communities are concerned about potential chemical impacts on nervous system health (Aschner et al. 2017; Costa et al. 2008). Regulatory assessment of neurotoxicity hazards faces several challenges: (1) thousands of chemicals lack toxicological hazard characterization, especially for neurotoxicity and developmental neurotoxicity (DNT) (Judson et al. 2009), (2) current test guidelines are expensive, time-consuming, and recommend arduous and resource-intensive animal studies (Bal-Price et al. 2018), and (3) in vivo studies present uncertainties such as variability, reproducibility, and interspecies translation of findings (Matthews 2008; Paparella et al. 2020; Tsuji and Crofton 2012).

Considering these limitations, the National Academy of Sciences Report on “Toxicity testing in the twenty-first century” outlined the need for efficient and more human-relevant in vitro methods to screen chemicals for their potential to cause toxicity (NRC 2007). This report emphasized the growing necessity for New Approach Methodologies (NAMs) to facilitate hazard identification and risk assessment for the vast number of compounds lacking toxicological data, marking a pivotal call for a paradigm shift in toxicology. Building on this initiative, several regulatory frameworks have incorporated NAMs to reduce reliance on animal testing. For instance, the EU Cosmetics Directive (2013) implemented a complete ban on animal testing for cosmetics and their ingredients, mandating the exclusive use of alternative methods (EU 2013). Similarly, the U.S. FDA Modernization Act 2.0 (2022) (Adashi et al. 2023), recently signed into law, enables the FDA to accept NAMs as alternatives to animal testing for drug and biologics approval processes.

NAMs can include structure–activity relationship modeling, high-throughput screening (HTS) assays, in vitro models providing human relevance, and methods to extrapolate between in vitro and in vivo exposures. The concept of utilizing NAMs has garnered support and endorsement from various governmental institutions, including the European Food Safety Authority (EFSA)(Cattaneo et al. 2023, 2022), Interagency Coordinating Committee on the Validation of Alternative Methods (ICCVAM) (ICCVAM 2024), Organization for Economic Cooperation and Development (OECD), U.S. Environmental Protection Agency (EPA), National Institutes for Environmental Health Sciences (NIEHS), and Food and Drug Administration (FDA).

Recently, the OECD issued the ‘Initial Recommendations on the Evaluation of Data from the Developmental Neurotoxicity In Vitro Testing Battery’, founded upon NAMs principles (OECD 2023). To date, 17 in vitro NAMs have been developed to measure complex functional changes in bioactivity, which is critical for evaluating neurotoxicity potential (Sachana et al. 2021; van Vliet et al. 2007). Moreover, perturbations of neuronal activity can occur prior to, or in the absence of, obvious biochemical or morphological changes (Johnstone et al. 2010). Thus, efficient screening assays that detect functional changes from complex neurobiological systems are needed. Neuronal cultures grown on microelectrode arrays (MEAs) were proposed as an in vitro neurotoxicity screening method over a decade ago (Johnstone et al. 2010) and have demonstrated high reproducibility and reliability across multiple laboratories (Novellino et al. 2011; Vassallo et al. 2017). Since then, MEAs have been increasingly utilized for the evaluation of both acute and developmental neurotoxicity hazards (Gerber et al. 2021; Shafer et al. 2019).

Rodent and human neural networks grown on MEAs can be used to screen compounds for neurotoxic effects mediated by a variety of mechanisms (Gerber et al. 2021; McConnell et al. 2012; Shafer 2019). To date, the vast majority of this work has been conducted in 2D cultures. In recent years, evidence has emerged to suggest that 3D neural models may offer added value compared to 2D neural models, as the latter cannot mimic the intricate tissue microenvironment (Haycock 2011). 3D cultures enable cells to develop under more physiologically relevant conditions, demonstrating increased cell–cell interaction, cell–environment interaction and neuro-relevant cytoarchitecture. For instance, 3D organotypic cultures have demonstrated structures such as ventricle formation, cortical layer organization, myelin, and neuronal migration, which are either absent or less abundant in 2D neural models (Jo et al. 2016; Lancaster et al. 2013; Muguruma et al. 2015; Pasca et al. 2015). In addition, oligodendrocytes, which have traditionally been challenging to culture in 2D models (Chesnut et al. 2021), have been successfully differentiated in 3D cultures. The presence of glia cells such oligodendrocytes, astrocytes and microglia have shown to be playing and important role in neurotoxicity responses (Eskes et al. 2003; Zurich et al. 2002, 2004). Human models comprising heterogenous cell populations that mirror the complexity of the human brain are important for enhancing the biological relevance of the model, and 3D models may provide more complete coverage of complex biology than 2D models for neurotoxicity screening (Centeno et al. 2018; Zhang et al. 2014).

**Fig. 1:**
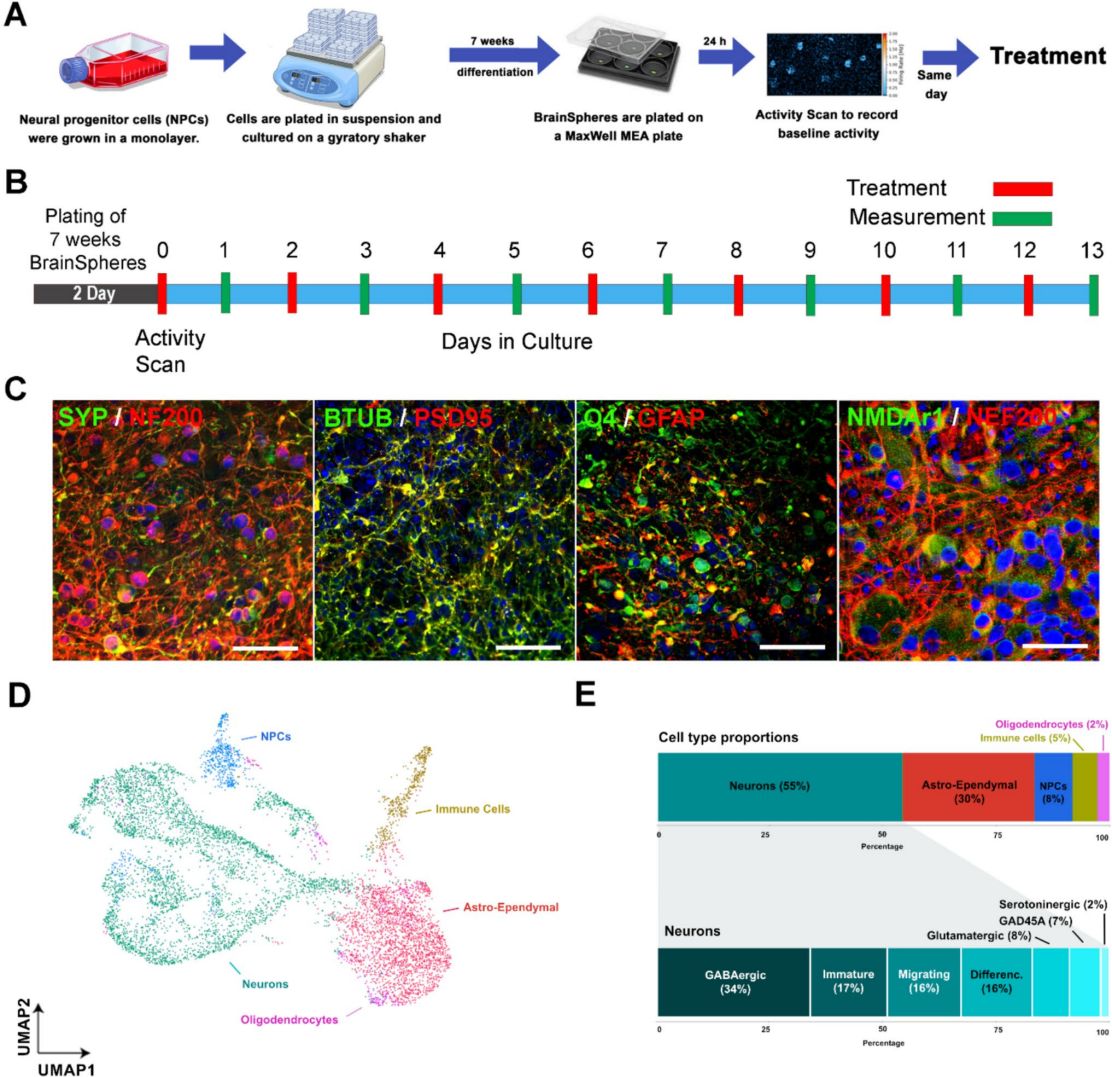
Experimental procedure and BrainSpheres characterization. The BrainSpheres underwent a 7-week differentiation process before being attached to HD-MEAs and fixed for immunohistochemistry. At 7 weeks, cells were treated with the different compounds from the training set. **A**) A diagram of the experimental procedure. **B**) A diagram of the electrical activity measurements and treatment. **C**) Representative immunohistochemical confocal images (max projections) of synaptic markers (SYP), postsynaptic markers (PSD95), NMDA receptor 1 marker (NMDAr1), neuronal markers (NF200 and BTUB), oligodendrocyte marker (O4), and astrocyte marker (GFAP) from 7-week-old BrainSpheres. (**D**) UMAP representation of scRNA-seq cell type distribution of BrainSpheres for 8516 cells, colored per cluster annotated according to known cell types. (**E**) Stacked bar graph displaying cell type proportion distribution across the whole dataset (top) and the proportion of neuronal subtypes. NPCs: neuronal progenitor cells

A promising 3D brain model, referred to as ‘BrainSpheres’, derived from human induced pluripotent stem cells (iPSCs) was previously developed and described in (Pamies et al. 2017). BrainSpheres comprise a heterogenous population of neurons (such as glutamatergic, GABAergic, dopaminergic and serotonergic), astrocytes and oligodendrocytes (Fig. 1D, E), forming a multicellular tissue with neural–glial interactions (e.g., myelination). In addition, recent studies demonstrate that spike sorting in BrainSpheres can differentiate between glutamatergic, GABAergic, dopaminergic, serotonergic, and cholinergic responses, highlighting the potential to detect complex functional activity (Hartmann et al. 2023). Furthermore, BrainSpheres show consistent morphological characteristics, such as shape, size (300 µm in diameter), and proportion of the different cell types from batch to batch, making them ideal for screening (Chesnut et al. 2021; Nunes et al. 2022; Pamies et al. 2018a; Plummer et al. 2019; Zhong 2020).

Traditional multi-well MEA systems typically incorporate 16–64 electrodes per well, arranged at distances of 200–350 µm which are recorded using external amplification circuitry. Recording neuronal activity from the BrainSpheres using such MEA systems would be limiting, considering factors such as large inter-electrode distances, limited number of electrodes contacting a single BrainSphere, and high inter-BrainSphere variability introduced by differences in positioning of the electrodes relative to the BrainSphere. In contrast, emerging high-density MEA (HD-MEA) systems (Berdondini et al. 2009; Bertotti et al. 2014; Muller et al. 2015) incorporating on-chip signal amplification circuitry provide low-noise readouts with electrodes arranged at densities of ~ 500–3200 electrodes/mm^2^. This technology allows for detection of electrical signals from virtually every cell on top of the array and are, therefore, highly suited for measuring high-content neuronal activity from BrainSpheres.

In the present study, we combined the iPSC-derived human 3D BrainSpheres with multi-well, HD-MEA technology to develop an in vitro assay system with the potential to detect chemically induced changes in neural activity using a complex tissue model. The large electrode area and high spatial resolution of the HD-MEAs allowed simultaneous recording of up to 70 BrainSpheres on a single 6-well HD-MEA plate. A ‘training set’ of 10 chemicals was tested in a multi-concentration screening approach, including chemicals that were previously reported to be active (or inactive) in an established MEA DNT assay using a 2D rat cortical model (Brown et al. 2016; Crofton et al. 2011; Frank et al. 2017b; Hogberg et al. 2011). Concentration–response modeling was performed to estimate bioactivity changes and potency estimates across a set of MEA endpoints and results from this human 3D BrainSphere model were compared to results from a comparable 2D-MEA assay using rat cortical cells. This work serves to evaluate the utility and relevance of the human 3D BrainSpheres model for neurotoxicity chemical screening, supporting the call for better coverage of human biology and mechanisms of toxicity in NAMs.

## Methods

### Neural progenitor cell (NPC) differentiation

Neural progenitor cells (NPCs) were generated from iPSCs derived from human CCD- 1079Sk fibroblasts (ATCC^®^ CRL- 2097) using Epstein–Barr virus-based vectors and embryoid body formation as previously described (Chiang et al. 2011; Wen et al. 2014). NPCs were then grown and amplified in cell culture flasks coated with Geltrex^®^ (ThermoFisher). The medium for NPCs was the Neural Expansion Medium (NEM) and consisted of Neurobasal^®^ Medium, Advanced^™^ DMEM/F- 12 Medium and Neural Induction Supplement. NPCs were kept at 37 °C with 5% CO_2_. The medium was replaced every 2 or 3 days. At 90% of confluency, cells were passaged in new culture flasks.

### BrainSpheres differentiation

At 95% NPC confluency, cells were washed with Dulbecco’s phosphate-buffered saline (DPBS). StemPro Accutase Cell Dissociation Reagent (GIBCO, A1110501) was used to detach the cells from the flask after an incubation of 2–3 min (min). Cells were placed in a 50 ml falcon tube, counted with a cell counter (Countess, Invitrogen) and centrifugated at 300×*g* for 4 min. After removing the supernatant, cells were suspended in NEM and seeded at 2 × 10^6^ cells in 2 ml/well in a non-treated 6-well plate. The plates were placed in an incubator at 37 °C and 5% CO_2_ under constant gyratory shaking (Kuhner orbital plate shaker, 88 rpm). After 2 days, medium was changed to neuronal differentiation medium (NDM). This medium consisted of neurobasal electro medium, 2% B27/Neurobasal Electro, 0.01 μg/mL BDNF (PeproTech), 0.01 μg/mL GDNF (PeproTech) and 1% Penicillin–Streptomycin (10,000 U/mL). Half-medium changes with NDM were completed three times a week and cultures were kept until 7 weeks (Fig. 1A). Immunohistochemistry followed by confocal analysis was performed at 7 weeks as a routine check for the presence of neuronal markers (NF200, Beta-III Tubulin), astrocyte markers (GFAP), oligodendrocyte markers (O4), a synaptic marker (SYP), a postsynaptic marker (PSD95), and the NMDA receptor 1 marker (NMDAr1) as shown in Fig. 1C. For immunostaining method, see below.

### Immunohistochemistry

BrainSpheres were fixed for 1 h (h) with 4% paraformaldehyde, followed by three washes with 1 × PBS for 5 min each. Next, they were incubated in a blocking solution (5% normal goat serum (NGS) and 4% 1 × Triton in 1 × PBS) for 2 h on a shaker at room temperature. Afterwards, BrainSpheres were incubated with primary antibodies (Table 1), diluted in PBS containing 5% NGS and 1% 1 × Triton, at 4 °C for 24 h. Subsequently, BrainSpheres underwent three 5 min washes with PBS and were then incubated for 1 h with secondary antibodies (goat anti-mouse Alexa Fluor 488 IgG from Invitrogen or goat anti-rabbit Alexa Fluor 568 IgG from Invitrogen, Waltham, MA, USA), at a 1:200 dilution in PBS containing 5% NGS, on a shaker at room temperature. Following this, BrainSpheres were washed with PBS three times, with each wash lasting 5 min. Nuclei were stained with Hoechst 33,342 trihydrochloride trihydrate from Invitrogen, at a 1:10,000 dilution in PBS, for 10 min on a shaker. Finally, BrainSpheres were mounted on glass slides using a mounting medium (Immu-Mount from Thermo Scientific, Waltham, MA, USA). The images were captured using a confocal microscope (Zeiss LSM 780 GaAsP) and visualized using ZEN Imaging software from Zeiss, and ImageJ (Fig. 1C).

**Table 1:** List of antibodies

Gene name	Company	Reference number	Dilution	Marker
SYP	Sigma	S5768	1: 200	Synapses
NF200	Sigma	N4142	1:100	Neurons
BTUBIII	Sigma	T8660	1:200	Neurons
PSD95	Life Technologies	700,902	1:200	Post synaptic
O4	R&D systems	MAB1326	1:200	Oligodendrocytes
GFAP	Sigma	G9269	1:200	Astrocytes
NMDAr1	Invitrogen	32–0500	1:200	NMDA receptor

### Single-cell RNA sequencing

BrainSpheres at 8 weeks old were incubated in 10 U/mL Papain enzyme solution for 3 h at 37 °C and dissociated into single cells. Cell fixation was performed according to the protocols from 10X Genomics (Manual CG000478, Revision D). Cryopreserved cells were then resuspended in 0.5 × PBS + 0.02% BSA + 0.2 U/ul RNase inhibitor and counted with LUNA FX7 (Logos Biosystems).

Fixed cells profiling was then run using the Single Cell Gene Expression Flex kit from 10X Genomics, strictly following the official protocol CG000527 (Revision E). For each sample, 500′000 cells were hybridized using the Chromium Human Transcriptome Probe Set v1.0.1 (PN- 1000456). The total number of cells per sample was then pooled and washed 3 times using the Post-Hyb Buffer as recommended by 10X Genomics. The pool was then resuspended in the Post-Hyb Resuspension Buffer, counted with the LUNA FX7, loaded onto a Chip Q and processed with the Chromium X (10X Genomics).

The sequencing library was then prepared, still following the manufacturer’s recommendations. Pre-amplification was run for 8 PCR cycles, and final amplification for 10 cycles. The library was quantified by a fluorometric method (QubIT, Life Technologies) and its quality assessed on a Fragment Analyzer (Agilent Technologies). Sequencing was performed on an Aviti instrument (Element Biosciences) using the CloudBreak FreeStyle chemistry. Small RNA primers were added for read 2 and index1 reads, according to Element Biosciences’ recommendations. Sequencing settings were 28 - 10-10–90 cycles (read1-index i7-index i5-read2) with 2% PhiX spike in. Base calling and demultiplexing was done with bases2fastq (version1.6.0) with the filter mask R1:N15Y* and the legacy-fastq option for fastq format compatibility with Illumina.

The expression matrix was used for downstream analysis in Seurat (version 5.0.1). Data were further filtered to contain cells with a minimum of 1,000 UMI/cell and a fraction of mitochondrial genes of > 0.1. Following filtering, data were normalized using scTransform(), followed by PCA decomposition with default parameters. Then, non-linear dimensionality reduction was done using RunUMAP() with the first 30 components. Based on UMAP representation, clustering was done by computing the Share Nearest Neighbor graph (with the function *FindNeighbors*) and applying a modularity-based cluster detection *FindClusters* with default parameters (Fig. 1D, E). Finally, cell type definition per cluster was done by extracting markers per cluster (using the function *FindAllMarkers* and comparing these makers with current knowledge and literature revision (Fig. 1D, E).

### Chemical selection

Different terminologies have been used to describe the compounds involved in developing NAMs, particularly in the area of DNT. We adopted the terminology outlined by (Crofton et al. 2011) and (OECD 2023). Here, ‘endpoint-selective compounds’ are defined as chemicals known to reliably and consistently alter the endpoint at a mechanistic level in the absence of cytotoxicity. This study included two endpoint-selective control positives (loperamide and domoic acid) and one endpoint-selective negative (acetaminophen) (Table 2) because they were shown to alter multiple parameters of network formation (the “endpoint”) in a rat network formation assay (rNFA), while acetaminophen did not (Brown et al. 2016). Loperamide is a mu-opioid agonist available over the counter (in the US) as an anti-diarrheal treatment. Despite being an opioid, it does not cause CNS sedation or induce developmental neurotoxicity (DNT) in vivo, as it is rapidly eliminated from the CNS by p-glycoprotein transporters. However, since these models lack a blood–brain barrier, loperamide does exhibit effects in these systems. In addition, ‘training set chemicals’ refer to a collection of chemicals used for proof-of-concept method testing. Seven training set chemicals were selected based on previously published work on the rNFA, demonstrating the capacity of MEAs to screen compounds for effects on network formation (Brown et al. 2016; Frank et al. 2017b). Six chemicals disrupted neural activity in the rNFA (Frank et al. 2017b; Shafer et al. 2019) and one chemical (amoxicillin) was negative in the rNFA and was identified as a favorable DNT negative chemical (Martin et al. 2022). Every chemical was prepared as a 10, 30 or 100 mM stock solution dissolved in DMSO and stored at − 20 °C. The stock solutions were aliquoted and diluted in NDM in a concentration series of 0.1 µM, 1.0 µM, 10 µM, 30 µM, and 90 µM for each chemical, with the exception of domoic acid, which was tested at 0.1 µM, 0.3 µM, 1.0 µM, 10 µM, and 30 µM in the MEA BrainSphere assay. These concentrations were selected based on the concentration tested in the rNFA. DMSO was added to the final dilution to achieve a consistent solvent concentration of 0.3%.

**Table 2:** List of compounds tested in the BrainSphere MEA assay

Name	CAS number	Concentration range (µM)	Solvent	Category
Acetaminophen	103 - 90- 02	0.1–90	DMSO	Endpoint-selective control negative
Loperamide	53,179 - 11- 6	0.1–90	DMSO	Endpoint-selective control positive
Domoic acid	14,277 - 97- 5	0.1–30	DMSO	Endpoint-selective control positive
Deltamethrin	52,918 - 63- 5	0.1–90	DMSO	Training set positive
Dieldrin	60 - 57- 1	0.1–90	DMSO	Training set positive
Methylmercury	22,967 - 92- 6	0.1–90	DMSO	Training set positive
BDE- 47	5436 - 43- 1	0.1–90	DMSO	Training set positive
Bisphenol A	80 - 5- 7	0.1–90	DMSO	Training set positive
Valproic acid	1069 - 66- 5	0.1–90	DMSO	Training set positive
Amoxicillin	26,787 - 78- 0	0.1–90	DMSO	Training set negative

Table of chemicals tested in the BrainSphere MEA assay indicating the chemical name, CAS number, concentration range tested, and chemical category (assay performance control versus training set), which were based on results from the rNFA (Brown et al. 2016)

### BrainSphere MEAs plating and chemical screening

A volume of 1 ml of Tergazyme (1% solution in deionized water) was added to each well of the MaxTwo 6-Well Plate (MaxWell Biosystems) and incubated overnight under constant gyratory shaking (88 RPM) at 37 °C and 5% CO_2_. The next day, Tergazyme solution was removed, and plates were washed with distilled water and ethanol. After remaining ethanol was evaporated, wells were washed with PBS. Then, 200 µl of Poly-D-Lysine (Gibco, A38904 - 01) was added on top of the electrodes and incubated at 37 °C and 5% CO_2_ for 3 h (humidified). Poly-D-Lysine was then removed, and wells were coated with 100 µl of 0.1 mg/ml laminin coating solution (Sigma-Aldrich, L2020 - 1 mg) and incubated for 2 h at 37 ℃ and 5% CO_2_ (humidified). The laminin was removed after incubation. Using a 1 ml micropipette, 1 ml of medium containing BrainSpheres (7 weeks differentiated), approximately 25 BrainSpheres per ml, was plated on top of the HD-MEA chip (Fig. 1A). Afterwards, the plate was placed in an incubator at 37 °C and 5% CO_2_ and moved in circles to accumulate the BrainSpheres on top of the electrode area (Fig. 1A). The plate was kept in an incubator (humidified) for 2 days to allow attachment of the BrainSpheres with no gyratory shaking of the plates.

### Microelectrode array recordings

ActivityScan assay and network assay recordings for BrainSphere selection.

**Fig. 2:**
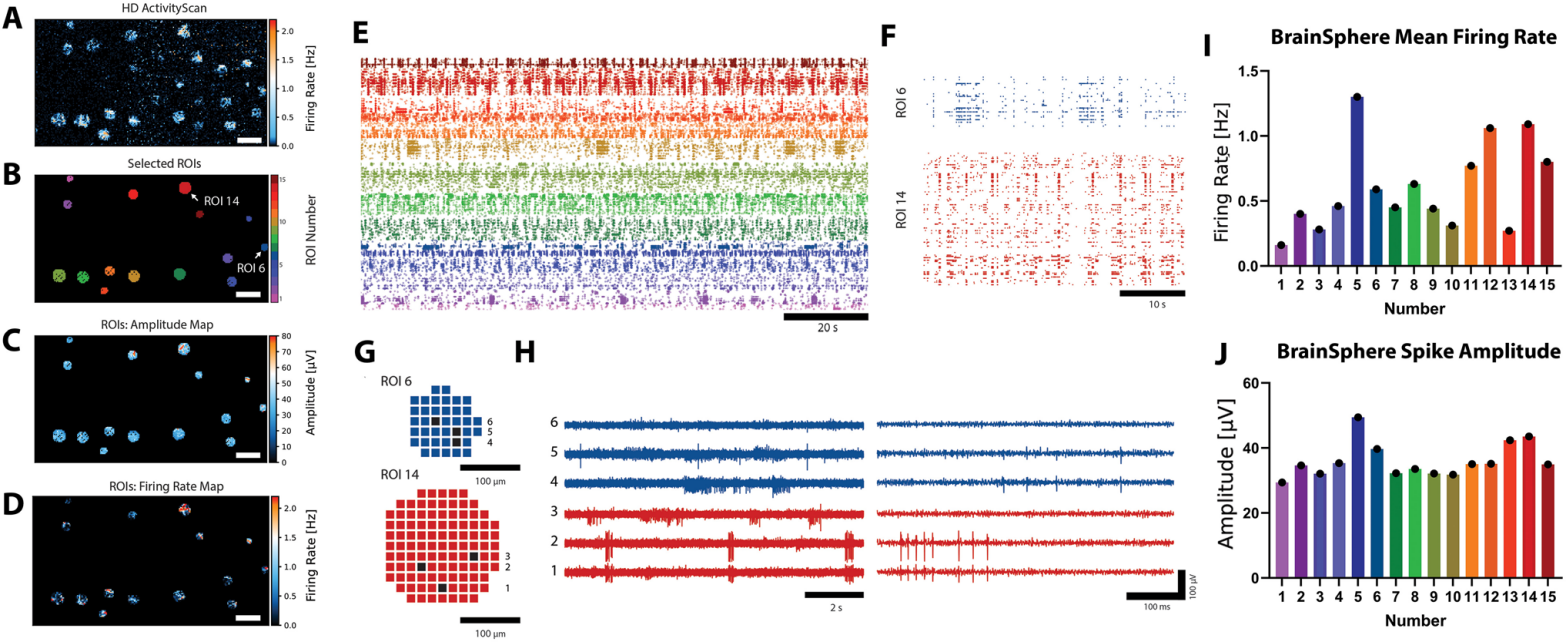
Spontaneous BrainSphere Electrical Activity recorded with MaxTwo HD-MEA. (A) Firing Rate Map of an individual MaxTwo HD-MEA well with plated BrainSpheres, where each pixel represents the Firing **Rate of an individual electrode recorded during a high-density ActivityScan Assay. Increased levels of activity can be seen** at the electrodes underneath the BrainSpheres. (B) Circular regions at the BrainSpheres were selected based on the Firing Rate **Map shown in A. The electrodes were grouped into regions of interest (ROIs) reflecting the individual BrainSpheres.** Scale bar: 400 μm. (C)-(D) Spike Amplitude and Firing Rate Maps, respectively, recorded during a Network Assay with the electrodes of the ROIs shown in B. Scale bars: 400 μm. (E) Raster plot showing detected events (represented by individual dots) for 120 s recorded with the Network Assay. Events were colored and grouped according to the ROIs shown in B. (F) Close-up raster plot (30 s) for two selected ROIs (no. 6 and 14, see B), displaying distinct types of BrainSphere firing dynamics. (G) Illustration of the electrodes underneath ROI 6 (39 electrodes) and ROI 14 (104 electrodes). Three representative electrodes were chosen per ROI. (H) Left: recorded HD-MEA signals on the electrodes indicated in H; Right: close-up visualizing individual recorded action potentials. (I) **Bar plots displaying Mean Firing Rate and (J) Spike Amplitude, respectively,** calculated for the individual ROIs shown in B

The MaxTwo high-density microelectrode array system (MaxWell Biosystems AG, Zurich, Switzerland) was used to measure neural activity with MaxTwo 6-well plates, where every well includes an integrated chip with 26,400 platinum electrodes arranged in an area of 3.85 × 2.10 mm^2^ (3150 electrodes/mm^2^) (Muller et al. 2015). The data were recorded and analyzed with the MaxLab Live Software (version 22.2.22). First, the MaxLab live ActivityScan Assay was used to identify the activity and positioning of the BrainSpheres on the array by scanning all the 26,400 electrodes in 29 configurations in a well (Assay Parameters: Configuration = Full; Recording time per configuration = 60 s (s)). The settings gain = 512, high-pass-filter (HPF): ~ 1 Hz and spike threshold = 5 X root-mean square (RMS) noise were used for all HD-MEA recordings. Circular regions of interest (ROI) were manually selected in the Activity Map where neuronal activity was apparent by an increased activity (Fig. 2). Over the course of the 13-day experiment, the same selected regions and electrodes were measured using the MaxLab Live Network Assay (600 s recording time). In this study, the total number of BrainSpheres per well ranged from 15 to 40; considering the 1020 simultaneously recorded electrodes per well, an average of 15 BrainSpheres (ROIs) per well were selected for measurement, with typically 50–150 electrodes underneath one BrainSphere for recording. We selected the BrainSpheres that exhibited a qualitative higher spike rate during the initial ActivityScan Assay.

A custom analysis framework was used to extract the MaxLab Live neuronal metrics for the individual BrainSpheres. For this, electrodes comprising a single ROI (i.e., individual BrainSphere; Fig. 2D) were grouped using distance-based clustering (fcluster method of Scipy library, Python 3.7) and neural metrics were calculated for the clustered electrodes for every ROI separately. Figure 2E presents a raster plot illustrating the activity associated with individual Regions of Interest (ROIs). For enhanced visualization, bar plots corresponding to this example are provided in Fig. 2F, G and H. To examine the data in greater detail, a 5 s close-up raster plot for two selected ROIs (Numbers 5 and 12) is shown in Fig. 2F, and the recorded HD-MEA signals from two selected electrodes are displayed in Fig. 2H. Supplemental Fig. 2 shows and example of individual ROI mean firing rate of time. Ten MaxLab Live neural metrics (endpoints) were evaluated from the MaxLab live ActivityScan and Network Assay in this study. See Table 3 for the endpoint definitions, tuned parameters, and activity type (general versus network connectivity).

**Table 3:** MaxLab Live neural metric endpoints

MaxWell Live neural metric endpoints	Endpoint description	Activity type
Mean. Spheroid. Firing. Rate	The firing rate averaged over all active electrodes of the spheroid. An electrode is considered active if it shows a spike amplitude (90 th percentile) > 20 µV	General
Spheroid. Spike. Amplitude. 90 th. Percentile	Average amplitude value over all active electrodes of the spheroid. Per electrode, the 90 th percentile of the spike amplitude distribution is considered	General
Spheroid. Burst. Frequency	The frequency of detected network bursts of the BrainSpheres. A ‘network burst’ was defined by the network burst detection algorithm, involving three steps: (1) spike times from all recorded electrodes are binned into a histogram with a fixed bin size of 10 ms. (2) The resulting histogram is smoothed by convolution using a Gaussian kernel and normalized by the total number of recorded electrodes. (3) Bursts are detected by identifying all local maxima that have a peak Firing Rate above the “Burst Detection Threshold” (default 1.2 Hz) and that are spaced further apart (in time) from a previously detected network burst than the “Minimum Peak Distance”. Only the parameter “Burst Detection Threshold” is considered for the first network burst	Network connectivity
Mean. Spheroid. Nonburst. ISI	The average interspike intervals (ISI) for all spikes outside network bursts of the spheroid	General
Mean. Spheroid. IBI	The average time between consecutive detected network bursts of the spheroid	Network connectivity
Mean. Spheroid. Burst. ISI	The average interspike intervals (ISI) for all spikes within network bursts of the spheroid	Network connectivity
Mean. Spheroid. Burst. Duration	The network burst duration averaged over all detected bursts of the spheroid	Network connectivity
Mean. Spheroid. Burst. Peak. Firing. Rate	The network burst peaks averaged over all detected bursts of the brain spheroid. The peaks are derived by binning all detected spikes at the spheroid into 10 ms bins and convolving the resulting curve with a Gaussian Kernel (0.5 s window)	Network connectivity
Mean. Spheroid. Spikes. per. Burst	The average number of spikes per network burst of the spheroid	Network connectivity
Mean. Spheroid. Spikes. per. Burst. per. Electrode	The average number of spikes per network burst of the spheroid, normalized by the total number of recorded electrodes in the ROI	Network connectivity

Table listing the 10 neural metric endpoints measured using the ActivityScan and Network Assay, including a description of each endpoint and the type of neural activity (general or network connectivity)

### BrainSphere MEA assay

The BrainSphere MEA assay measured changes in neural activity during a 13-day chemical exposure. The first recording day of the concentration–response screening protocol (day of exposure (DOE) 0) occurred after 7 weeks of BrainSphere differentiation, 48 h after plating on HD-MEA plates (Fig. 1B). The study included at least three biological replicates (one well per plate) from separate experiments (e.g., three different plates on three different days) and each well included multiple BrainSpheres which were considered technical replicates. In each well, baseline activity was recorded from the selected ROIs for 10 min using the MaxLab live network assay. After the baseline recording, the treatment was added to each well (one control well per plate was treated with DMSO). Recordings were measured every other day starting on DOE 1 and ending on DOE 13 (Fig. 1B). Media changes occurred every other day starting 48 h before the initiation of the experiment (DOE 0) and termination on DOE 13. Chemical treatment was replenished with each media change.

### Cytotoxicity evaluation.

For the resazurin assay, BrainSpheres were cultured for 7 weeks in non-treated 6-well plates under gyratory shaking. Subsequently, the cells were exposed in the same manner as the HD-MEA plates. This assay was used to determine cell viability after exposure for 13 days to the different compounds. After exposure, resazurin diluted in PBS was added directly to the 6-well plates to a final concentration of 0.1 mg/ml. The plates were then put in an incubator for 3 h at 37 °C, 5% CO_2_. Then, 100 µl of the media was collected and transferred in a 96-well plate for fluorescence analysis, which was done at ex:530 nm/em:590 nm in a multi-well fluorometric reader CytoFluor series 4000 (PerSeptive Biosystems, Inc).

### Assay performance evaluation

Assay performance metrics commonly used for quality control of high-throughput bioactivity screening assays were evaluated. The coefficient of variation (CV) of control wells was computed as the standard deviation (SD) of controls divided by the mean of controls. A Z’ statistic was computed to evaluate the degree of separation between the assay performance controls and the vehicle control values per endpoint with the equation:

$$Z{\prime} = 1 - \left( {\frac{{3 \times \left( {sd.pos + sd.ctrl} \right)}}{{abs\left( {mean.pos - mean.ctrl} \right)}}} \right)$$

where *sd.pos* is the SD of the endpoint-selective control positive, *sd.ctrl* is the SD of vehicle controls, *mean.pos* is the mean of the endpoint-selective control positive, and *mean.ctrl* is the mean of vehicle controls. The strictly standardized mean difference (SSMD) was computed to distinguish positive responses from control responses on the basis of effect size (Bray et al. 2004) and is calculated as

$$SSMD=\frac{mean.po-mean.ctrl }{sqrt({sd.pos}^{2}+{sd.ctrl}^{2})})$$

### Concentration–response data analysis

Ten metrics were measured to evaluate changes in neural activity in the BrainSphere MEA assay (Table 3). Considering the complexity of the data which includes seven recording days for each single ROI in a well, an area under the curve (AUC) metric was used to reduce activity across time as described in previous publications (Brown et al. 2016; Frank et al. 2017b; Shafer et al. 2019). Briefly, the AUC was calculated using trapezoidal integration of responses from days 1, 3, 5, 7, 9, 11 and 13 for each chemical treatment and control wells. For concentration–response modeling, the mean AUC values across all the BrainSpheres per well (technical replicates) was computed for each endpoint, such that each concentration included biological replicates from at least three separate wells on three separate plates, representing each individual experiment. The mean AUC values were used for concentration–response modeling using the R package tcplfit2 (version 0.1.6) (Sheffield et al. 2022). Data were normalized as a percent of vehicle control wells by plate using the equation:

$$resp.pc=\frac{rval-bval}{bval}\times 100$$

where *resp.pc* is the percent of control value, *rval* is the response value, and *bval* is the baseline value (defined as the mean of the vehicle control and the lowest tested concentration). Concentration–response modeling was performed using bi-directional curve-fitting of nine parametric models in tcplfit2 (‘cnst’, ‘hill’, ‘gnls’, ‘poly1’, ‘pow’, ‘exp2’, ‘exp3’, ‘exp4’, and ‘exp5’), and the winning model and bioactivity hit calls (active or inactive) were determined for each curve. The activity cutoff was defined as 3 times the median absolute deviation (bmad) of baseline values. A curve was defined as ‘active’ if the continuous hit call was ≥ 0.9. See Supplemental Table 1 for the bmad, activity cutoff threshold, SD, and benchmark response for each endpoint in the BrainSphere MEA. Potency was defined as the concentration at 50% maximal activity (AC_50_ log10 µM).

### Comparison between BrainSpheres and rNFA

**Fig. 3:**
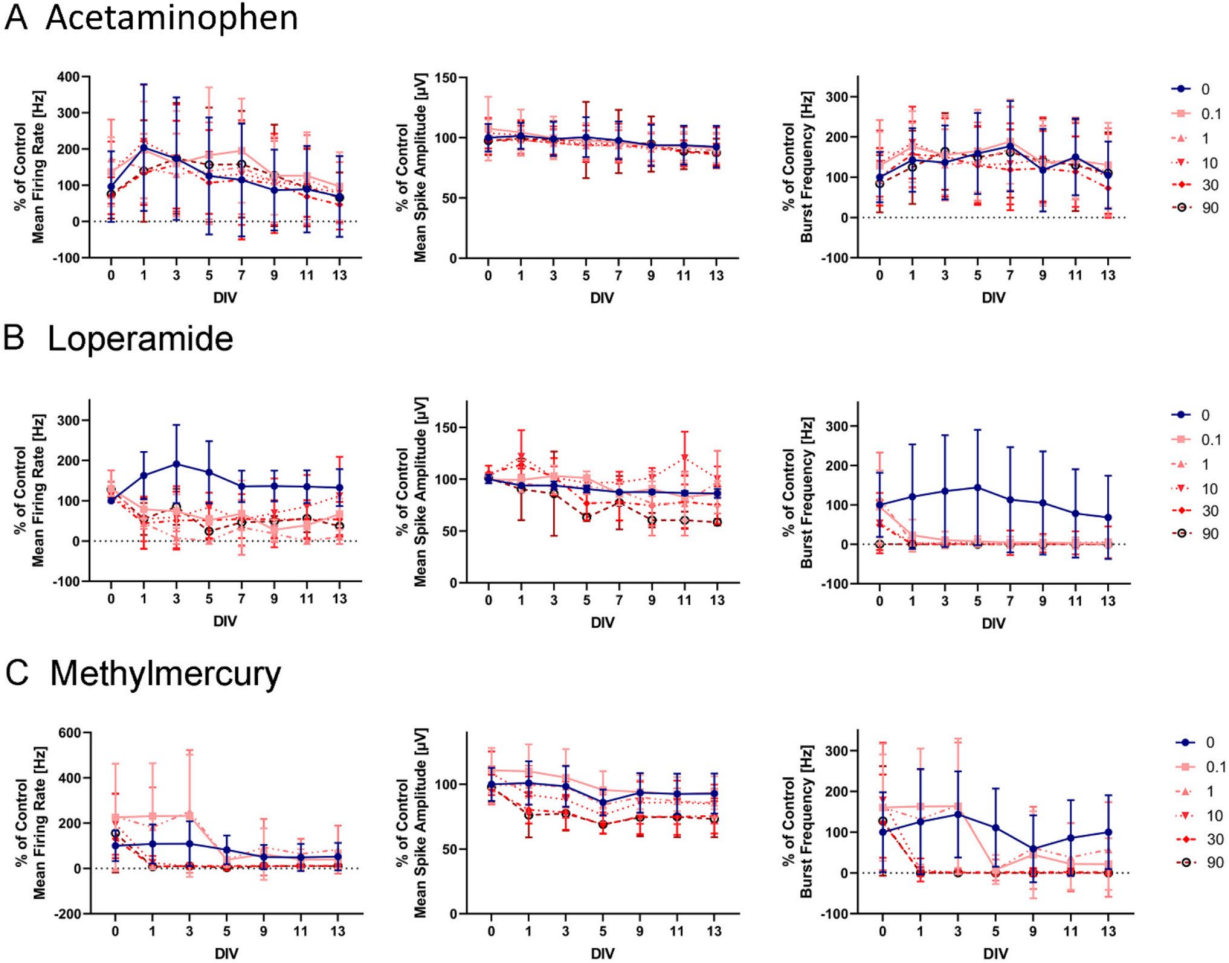
Three example MEA endpoints for acetaminophen, loperamide, and methylmercury. Graphs represent the mean firing rate (Hz), mean spike amplitude (µV), and burst frequency (Hz) for acetaminophen (endpoint-selective control negative), loperamide (endpoint-selective control positive), and methylmercury (positive evaluation chemical) during the 13-day exposure. Line colors indicate the concentration tested (µM). Data were normalized to vehicle control (concentration 0) on DIV 0 and data represent the mean across biological and technical replicates. The error bars indicate the standard error of the mean (SEM)

Data extraction, analyses, and figures were performed using the R statistical programming language (version 4.4.1), with the exception of plots in Fig. 3 and Supplemental Fig. 1 which were performed using Prism 9.0. The heatmaps were made with the R package ‘pheatmap’ (v 1.0.12), the scatter plots were made with R package ‘ggplot2’ (v 3.5.1) and the correlation plot was made with the R package ‘corrplot’ (v 0.92). Concentration–response modeling results from the BrainSphere MEA assay were compared to the rNFA using previously published data (Brown et al. 2016; Carstens et al. 2022; Frank et al. 2017b; Shafer et al. 2019) and extracted from ToxCast invitrodb (v4.2) (Feshuk et al. 2023) (Supplemental Table 2 for tcpl analysis methods and Supplemental Table 3 for bioactivity results from tcpl ‘level 5’ data). rNFA endpoints were labeled with the assay name ‘CCTE_Shafer_MEA_dev’, which included 17 endpoints measuring changes in general activity, bursting activity, network connectivity. The rNFA includes a cytotoxicity measurement using a resazurin dye-based assay similar to the BrainSphere MEA (AlamarBlue assay), labeled as the ‘CCTE_Shafer_MEA_dev_AB’ endpoint. The rNFA endpoints were mapped to the ‘equivalent’ endpoint in the BrainSphere MEA (Supplemental Table 4). Four pairs of equivalent endpoints were identified and subsequently used for the comparison of concentration–response results.

**Table 4:** Baseline neural activity metrics from BrainSpheres in a single well

Activity metric	Mean	SD	CV	Median	P10	P90
Mean. Spheroid. Firing. Rate [Hz]	0.17	0.18	1.07	0.08	0.05	0.43
Spheroid. Spike. Amplitude. 90 th. Percentile [Hz]	38.05	5.63	0.15	36.06	32.6	42.64
Spheroid. Burst. Frequency [Hz]	0.16	0.19	1.22	0.11	0.04	0.21
Mean. Spheroid. Spikes. per. Burst	51.98	63.36	1.22	30.2	17.26	92.18
Mean. Spheroid. Spikes. per. Burst. per. Electrode	0.68	0.75	1.1	0.37	0.16	2.08
Mean. Spheroid. Burst. Duration [s]	0.54	0.21	0.39	0.52	0.31	0.76
Mean. Spheroid. Burst. Peak. Firing. Rate [Hz]	1.13	0.86	0.76	0.8	0.52	2.53
Mean. Spheroid. IBI [s]	12.71	9.36	0.74	8.67	5.64	25.72
Mean. Spheroid. Burst. ISI [ms]	102.8	35.6	0.35	100.22	66.59	154.01
Mean. Spheroid. Nonburst. ISI [ms]	1795.76	1741.82	0.97	789.6	527.52	3593.24

MEA neural activity metrics from a representative well (untreated) comprising 12 BrainSpheres, measuring features such as general activity and network connectivity. The mean, standard deviation (SD), coefficient of variation (CV = SD/mean), median, 10 th percentile (P10) and 90 th percentile (P90) of each endpoint were computed across the 12 BrainSpheres in the well

## Results

### BrainSphere baseline neural activity characterization

The morphology and molecular expression profile of the BrainSphere model used in this study have been extensively characterized (Chesnut et al. 2021; Pamies et al. 2017, 2022). Here, we show that the BrainSphere model in this study contained astrocytes (GFAP-positive), oligodendrocytes (O4-positive) and expressed synapses (synaptophysin and PSD95-positive) (Fig. 1C) (Chesnut et al. 2021; Pamies et al. 2017, 2018b, 2022). Single-cell RNA seq have shown that the majority of cells presented in the BrainSpheres are neurons (Fig. 1D, E), followed by astrocytes, neuroprogenitor cells (NPC), immune cells and oligodendrocytes. In terms of neuronal population, we identified distinct neuronal populations, including GABAergic and glutamatergic neurons, migrating neurons, GADD45 A-specific neurons, immature neurons, and serotonergic neurons (Fig. 1E). Markers characteristic of GABAergic neurons were detected, including GAD1, GABRB3, and GABBR1, while glutamatergic neurons expressed SLC17 A7, SLC17 A8, and GRIN1. Notably, despite the presence of excitatory and inhibitory neuronal markers, the majority of neuronal populations remained in an immature state, as indicated by the expression of DCX, a marker associated with neuronal immaturity and migration.

A preliminary scan of neural activity using the MaxLab Live ActivityScan Assay was performed to measure baseline spontaneous activity of the BrainSpheres in a single well of the HD-MEA plates. Spontaneous neuronal activity was recorded for 10 min from a representative well (untreated) that contained 12 BrainSpheres and ten different metrics of activity were measured (Table 4). The mean firing rate (MFR) across the 12 spheroids was 0.17 Hz (± 0.18 SD), with a mean spike amplitude of 38.5 µV (± 5.63 SD). Network bursting activity demonstrated a 0.16 Hz burst rate (± 0.19 SD), 51.98 spikes per burst (± 63.98 SD), and a 0.54 s burst duration (± 0.21 SD), indicating that the BrainSpheres formed functional neural activity that demonstrates network connectivity and bursting activity.

### BrainSphere MEA assay

The BrainSphere MEA assay was developed to measure chemically induced changes in neural activity during a 13-day exposure of 7-week-old BrainSpheres (Fig. 1B). Neural activity was measured on seven recording days after chemical exposure (DOE 1, 3, 5, 7, 9, 11, and 13). A set of ten chemicals were tested in a multi-concentration series, comprising two endpoint-selective positive controls (loperamide and domoic acid), one endpoint-selective negative (acetaminophen), and seven evaluation chemicals (Table 2). In Fig. 3, bioactivity trends over time (DOE 0–13) are shown for loperamide, acetaminophen, and methylmercury (training set positive) for three MEA endpoints. The mean vehicle control responses (concentration 0) were relatively consistent over time but exhibited considerable variability across biological replicates, noting larger variability in the burst frequency endpoint relative to MFR and mean spike amplitude (data were normalized to the mean across all controls on DOE 0 (both technical and biological replicates); error bars indicate the standard error of the mean (SEM) across all technical and biological replicates). Qualitatively, the highest tested concentrations of loperamide and methylmercury appeared to decrease activity for the MFR, mean spike amplitude, and burst frequency endpoints throughout the 13-day exposure, while no clear chemical-dependent effects were observed from acetaminophen. Cytotoxicity, which was measured on the last day of the experiment (Day 13), may have played a role in decreasing neural activity effects (Supplemental Fig. 1), particularly for loperamide which appeared to demonstrate cytotoxic effects at 30 and 90 µM. These results suggest that activity from vehicle controls on average was relatively consistent across the 13-day exposure in the BrainSphere MEA assay, and that chemical-dependent effects can be observed, noting some endpoints may demonstrate more variability in responses compared to others (quantified below).

### Concentration–response modeling

An area under the curve (AUC) approach was applied to summarize activity changes over time for concentration–response modeling, which was previously established and described in (Frank et al. 2017a; Martin et al. 2024; Shafer et al. 2019). The distribution of AUC values by treatment and plate (one biological replicate) are shown in Supplemental PDF 1, where data points represent technical replicates. Several assay performance metrics were evaluated to determine the separation of the endpoint-selective controls relative to baseline for each endpoint. For the endpoint-selective controls loperamide and domoic acid, Z’ and SSMD did not exceed 0 suggesting weak separation between positive controls and baseline (Supplemental Table 5). The CVs of controls ranged from 0.12 (spike amplitude) to 0.85 (MFR) across endpoints indicating some endpoints demonstrate high baseline variability across biological replicates. We compared these results to the rNFA, a similar MEA assay using 2D rat cortical cultures, and found that the CVs ranged from 0.34 to 1.3 and the Z’ and SSMD did not exceed 0 for loperamide and domoic acid (Supplemental Table 6), suggesting comparable assay performance metrics to the MEA BrainSphere assay. In the future, increasing the number of biological replicates per plate and applying additional neural activity filters will likely improve the robustness of the assay for chemical screening.

**Table 5:** Chemical screening results of the BrainSphere MEA assay

Chemical	Hits	Percent active	Cytotoxicity hit	Minimum potency (µM)	Mean potency (µM)	SD potency (µM)
Acetaminophen	0	0	0	NA	NA	NA
Amoxicillin	0	0	0	NA	NA	NA
BDE- 47	8	80	0	8.22	11.37	2.76
Bisphenol A	9	90	0	29.14	36.29	8.27
Deltamethrin	10	100	0	1.6	5.23	3.25
Dieldrin	9	90	0	9.51	14.27	6.21
Domoic acid	9	90	0	1.3	3.32	1.34
Loperamide	10	100	1*	0.03	2.68	5.74
Methylmercury	10	100	0	2.41	6.15	4.05
Sodium valproate	0	0	0	NA	NA	NA

Table summarizing the hit call and potency results from the chemical screening of 10 chemicals in the BrainSphere MEA assay. ‘Hits’ indicates the total number of active endpoints out of 10 activity metrics evaluating MEA activity, ‘percent active’ indicates the number of metrics active out of the total number of metrics screened, ‘cytotoxicity hit’ indicates whether the chemical was active in the cytotoxicity resazurin assay (1) or inactive (0). The minimum, mean, and SD potency indicate the 5 th percentile, mean, and standard deviation (SD) across active AC50 values per chemical. *The potency of the cytotoxic hit for loperamide was 18.1 µM (AC_50_)

**Fig. 4:**
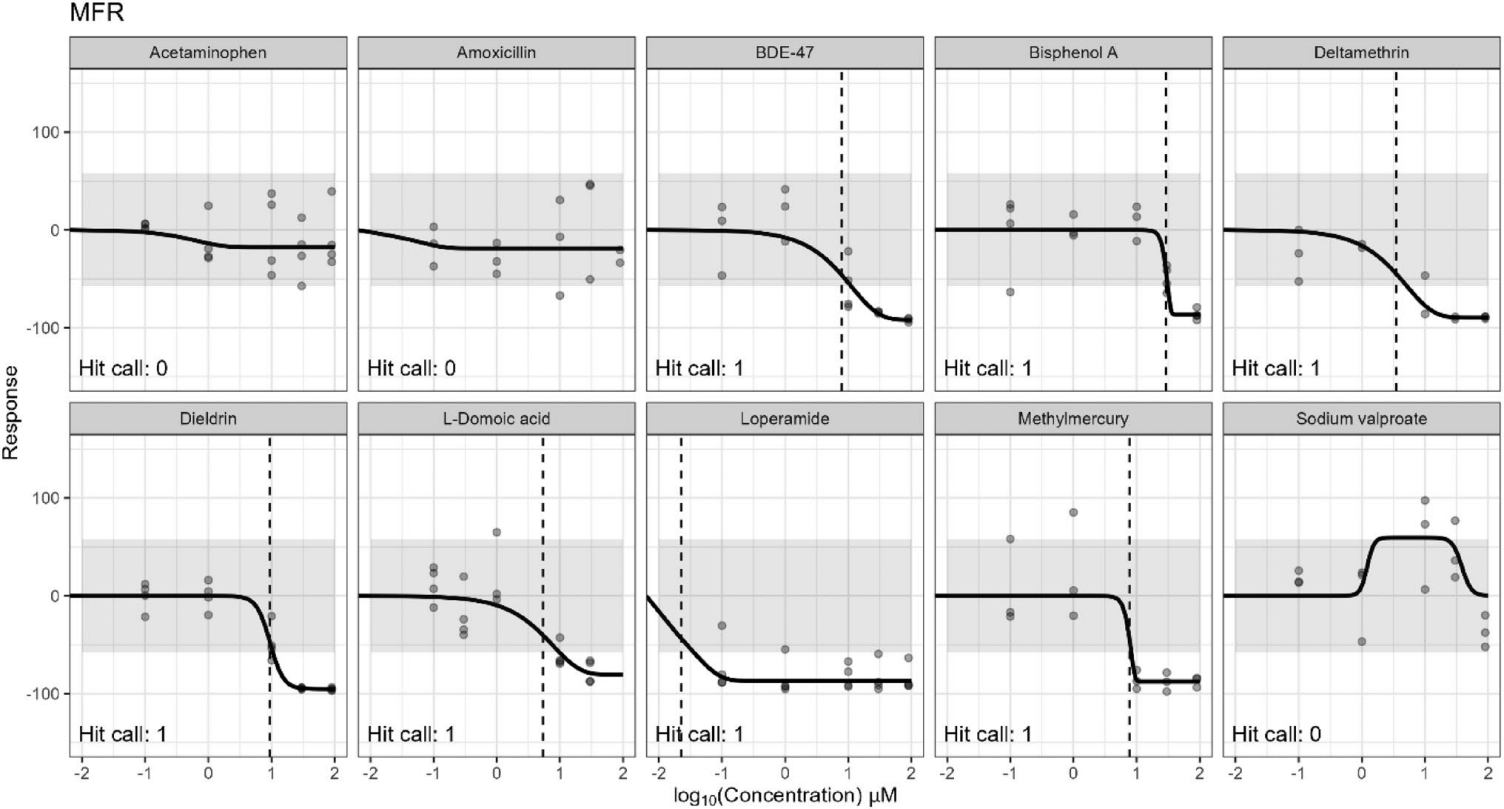
Concentration–response curve for the MFR endpoint. Concentration–response curves for the MFR endpoint indicate the hit call determination of 1 (active) or 0 (inactive) for the ten tested chemicals. The dotted line indicates the concentration at 50% maximal activity (AC_50_). The gray box indicates the activity threshold cutoff as defined by baseline noise

Concentration–response modeling was performed on the 10 chemicals screened in the BrainSphere MEA assay for 10 neural activity endpoints and a cytotoxicity metric measured on the last day of the experiment (13 days of exposure). Bioactivity results are shown in Supplemental Table 7 and representative curves for the MFR endpoint are shown in Fig. 4 (see Supplemental PDF 2 for all endpoints and chemicals). Loperamide was the only chemical that demonstrated a positive hit call for cytotoxicity at an AC_50_ of 18.1 µM. Decreased cell viability in the resazurin assay was suggested for several chemicals (methylmercury, domoic acid, and deltamethrin) at high concentrations, but the magnitude of the response was not sufficient to indicate a positive response. Out of the 10 MEA endpoints, the endpoint-selective control positives, loperamide and domoic acid, were active in 9 and 10 endpoints, respectively, while the negative control acetaminophen was inactive across all endpoints (Table 5). Five evaluation chemicals were active in ≥ 8 endpoints, with the exception of sodium valproate and amoxicillin, which were inactive across all metrics. Amoxicillin was selected as a ‘negative’ evaluation chemical based on previous findings that amoxicillin was inactive in the rNFA. The inactivity of sodium valproate, which was expected to be active, was likely explained by the maximal concentration tested being too low. Sodium valproate was active in the rNFA at concentrations exceeding 450 µM, while the maximum concentration tested in the study herein was 100 µM. We might expect to see activity in the BrainSphere MEA from sodium valproate at higher concentrations. The compounds, ranked by increasing minimum potency (5 th percentile across active AC_50_ values), are as follows: loperamide (0.03 µM), deltamethrin (1.6 µM), domoic acid (1.3 µM), methylmercury (2.41 µM), dieldrin (9.51 µM), and bisphenol A (29.14 µM).

### Comparison of MEA assays using a 3D human model versus 2D rat model

**Fig. 5:**
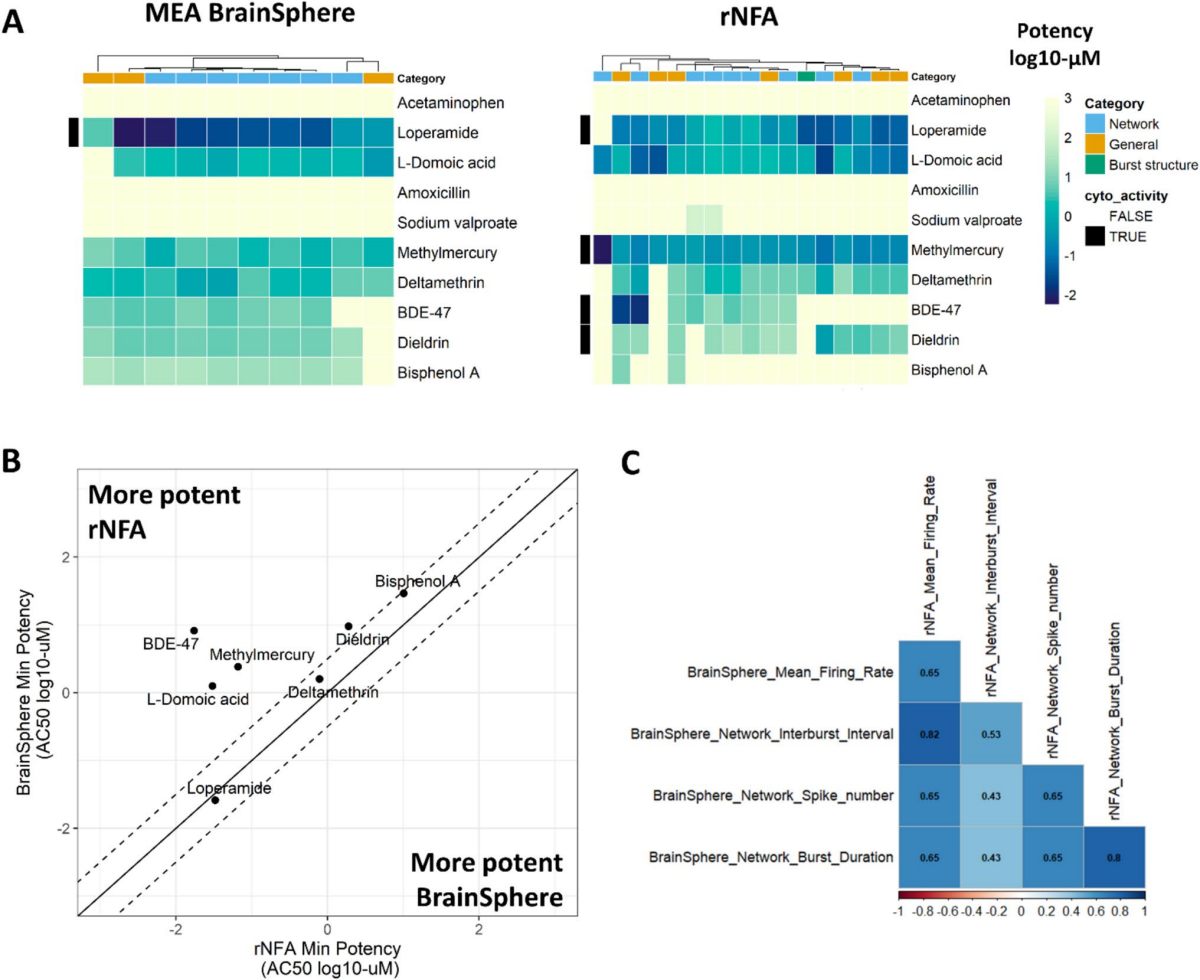
Comparison of MEA BrainSphere assay and rNFA concentration–response modeling results. A. Heatmap showing potency results across the MEA endpoints (columns) for the ten tested chemicals (rows) in the MEA BrainSphere (left) and the rNFA (right). The MEA BrainSphere comprised of 10 endpoints measuring general and network activity (Categories) and the rNFA comprised of 17 endpoints measuring general, network, and burst structure (Categories). Color key values indicate the potency (AC_50_ log10 µM) for each active curve with dark blue indicating more potent values and lighter green/yellow indicating less potent values. Inactive curves are colored yellow. B. Comparison of minimum potency (5 th percentile across active hits) between the two assays. Solid line indicates the line of unity and dotted lines indicate ± 0.5 log10 µM from the unity line. Upper left quadrant indicates chemicals that were more potent in the rNFA. C. Correlation matrix of bioactivity hit call determinations for four endpoints that were measured in both assays with darker blue indicating an increasing positive correlation coefficient

We next compared the bioactivity results from the BrainSphere MEA assay to the rNFA (Fig. 5). The BrainSphere MEA assay measured 10 endpoints representing general and network activity categories and the rNFA evaluated 17 MEA endpoints representing general, network and burst structure categories. While each assay measured a distinct set of metrics, four metrics were considered comparable between the two assays (MFR, network interburst interval, mean network burst duration, and network spike number) (Supplemental Table 4). We compared patterns of activity across all MEA endpoint measured in each model (Fig. 5A). Loperamide, domoic acid, methylmercury, deltamethrin, BDE- 47, and dieldrin demonstrated similar bioactivity profiles in both models, decreasing endpoints that measured general and network activity. Acetaminophen and amoxicillin demonstrated inactivity across all metrics in both models, indicating both assays demonstrate specificity, as these chemicals were expected to be negative. As indicated above, sodium valproate was inactive in the BrainSphere MEA but was active in two network activity endpoints in the rNFA, which was likely explained by differences in the maximum concentration tested. Bisphenol A was active in 9/10 endpoints in the BrainSphere MEA assay, while the rNFA demonstrated activity in only two endpoints. Notably, the rNFA detected more cytotoxicity effects in the resazurin assay with methylmercury, BDE- 47, and dieldrin demonstrating cytotoxicity in the rNFA but not the BrainSphere MEA model. Because of the dense cell mass of the BrainSpheres, the possibility that resazurin might underestimate cytotoxicity, particularly in the center of the cell mass, cannot be eliminated, and future work may be necessary to confirm the accuracy of this cytotoxicity metric for this model.

Minimum potency estimates were compared between the BrainSphere MEA assay and the rNFA. Of the seven chemicals that were active in at least one endpoint in each assay, we found that three chemicals estimated a minimum potency (5 th percentile of selective AC_50_ values by chemical) that was within 0.5 log10 µM between models (loperamide, deltamethrin, and bisphenol A) (Fig. 5B). Four chemicals demonstrated minimum potency values that were more potent in the rNFA by more than 0.5 log10-µM: domoic acid, methylmercury, dieldrin, and BDE- 47. No chemicals were more potent in the BrainSphere MEA assay. Lastly, we evaluated the correlation of bioactivity (hit call) for endpoints that were considered comparable between the two assays (Fig. 5C). The ‘network burst duration’ endpoint was the most highly correlated, demonstrating a correlation of 0.8, while the metric measuring network interburst interval was the least correlated (0.53) across the four endpoints. These data indicate that bioactivity profiles are similar between assays for 8/10 chemicals tested and minimum potency estimates appear to be comparable or more potent in the rNFA.

## Discussion

The present proof-of-concept study sought to develop and optimize a novel assay using a 3D BrainSphere model on a HD-MEA plate and evaluate the performance of this assay for concentration–response screening. We found that the BrainSphere MEA assay successfully exhibited spontaneous electrical activity across a 13-day recording window and detected chemically induced changes in neural activity from a set of ten chemicals screened in multiple-concentration. Two endpoint-selective control positives demonstrated perturbations in bioactivity occurring below the threshold of cytotoxicity in the BrainSphere MEA assay and an endpoint-selective control negative was inactive across all ten MEA endpoints. Application of concentration–response modeling demonstrated the ability to derive bioactivity and potency estimates using a 3D BrainSphere model on a HD-MEA, noting high baseline variability across most endpoints. Bioactivity and potency results were compared to an established MEA assay using a 2D rat cortical cell model (rNFA), revealing relatively similar trends in bioactivity, with the rNFA estimating more potent minimum effects for 4/7 chemicals. These findings exhibit a successful implementation of the HD-MEA technology with a human iPSC-derived 3D BrainSpheres and inform the potential added value of a 3D human-relevant model to detect chemically induced changes in neural activity for neurotoxicity hazard evaluation.

Primary cortical rat cultures, containing excitatory and inhibitory neurons as well as glial cells, have proven to be a valuable tool for evaluating neurotoxicity (Kosnik et al. 2020; McConnell et al. 2012; Strickland et al. 2018), and developmental neurotoxicity (Brown et al. 2016; Frank et al. 2017b; Hogberg et al. 2011; Shafer 2019). During the development of these assays, human cells were not widely available, expensive and did not exhibit consistent electrical activity on MEAs. More recently, the development of iPSCs and new differentiation protocols have made human cells more attainable and have resulted in consistent performance on MEAs (Tukker et al., 2016, 2018; Saavedra et al., 2021). It is hypothesized that NAMs using human-derived cell lines hold greater potential in predicting human toxicological effects compared to animal-based models. Human-relevant NAMs, for instance, can inform human biology and provide mechanistic information that may be relevant to disease or sensitive populations, thereby offering a more comprehensive representation of human phenotypes. Here, we adapted the MEA technology from traditional 2D rodent cultures to 3D human spheroids by employing a HD-MEA system and an analysis framework tailored for this application.

For years, in vitro studies have primarily relied on 2D cultures; consequently, the development of various technologies and readouts has also been grounded in monolayer cultures. Frequently, due to the complexity of 3D cultures, readouts are not always appropriately adapted. A notable example is the use of MEA systems, which have primarily based their technology on the measurement of adherent brain tissue. Measuring 3D cultures presents challenges not encountered with 2D monolayers, as the wells are not uniformly covered by a single layer of cells.

In conventional MEA systems with fewer electrodes (e.g., 16–64 electrodes) per well, arranged at larger inter-electrode distances (e.g., 100–200 µm), the positioned BrainSpheres might make contact with only one or two electrodes, which drastically increase the variability between wells and limits the functional readout for the analysis of network characteristics such as bursting and synchronous activity. The HD-MEA system used in this study offers a solution to this challenge due to the higher density of electrodes thereby facilitating greater numbers of electrodes in contact with the spheroids. This reduced spacing between electrodes in the HD-MEA allowed for the sampling of individual spheroids with typically 50–150 tightly spaced electrodes, enhancing the study of network characteristics by recording multiple neurons.

Another challenge with adapting the MEA system for a 3D cell model arises from the design of the standard MEA recording software. Typically, the recording software assumes that the cell model covers the entire electrode array in the well, which includes the HD-MEA system used in this study. However, in our experiments, which involved plating multiple spheroids in each well, software and analysis approaches geared towards 2D cultures do not support the extraction of data from individual spheroids, thereby complicating the investigation of spheroid-specific parameters. To address this, we used a ROI-based analysis method capable of extracting metrics from up to 25 individual BrainSpheres per well, which allowed for the collection of data at the level of individual BrainSpheres. This method is distinct from recent efforts to characterize organoids on HD-MEAs (Cai et al. 2023; Schröter et al. 2022; Sharf et al. 2022) (Elliott et al. 2023) in that the analysis method described herein pioneers recording from multiple organoids within well and between wells concurrently in a single HD-MEA plate. Employing this novel approach allowed us to simultaneously record from up to 25 BrainSpheres on a single well using MaxTwo 6-well plates.

Nevertheless, the methodology still faces challenges regarding the variability of the readout that need addressing to improve the assay’s robustness for chemical screening. Indeed, some variability is expected due to the biological complexity of this model. However, variability may also be explained by some aspects of the MEA recording. Although neural activity from the BrainSphere model is relatively consistent across the 13-day recording window, high CVs of vehicle controls suggest high variability across replicates. One contributing factor to the variability may be the inclusion criteria for neural activity. In this study, filtering criteria were applied on an electrode level (MFR > 0.1 Hz and a spike amplitude of > 20 µV), while no filters were applied to activity readouts from a whole BrainSphere. In the future, applying filtering criteria at the level of the BrainSphere may greatly improve replicate variability, such as setting a minimum burst frequency threshold on day 0, prior to chemical exposure. Moreover, variability was likely exacerbated by limitations from the assay plate format with the 6-well HD-MEA. Typically, MEA chemical screening assays are performed on a 48-well plate that allow for multiple controls wells per plate. Although wells in this study contained ~ 15 technical replicates per well, each plate only had one vehicle control well, limiting the robustness of plate-by-plate normalization. Future work to decrease variability in controls, such as applying more stringent filtering criteria of BrainSphere baseline activity or additional control wells per plate, may improve the robustness of this assay for chemical screening.

Comparative analysis of the BrainSphere MEA assay and rNFA indicated that the rNFA was more sensitive to chemical perturbation for four out of seven chemicals active in both assays and that the rNFA demonstrated more cytotoxic activity. The differences in sensitivity could be due to both technical and biological differences between the assays. Technically, data from these two assays were collected on different MEA systems with known differences in electrode density, endpoint parameterization, and data acquisition and analysis workflows. These differences likely have some impact on activity readouts; however, different MEA systems likely would not explain robust changes in neural activity. The BrainSphere MEA assay demonstrated similar Z’ and SSMD values as the rNFA assay, suggesting high variability may be common for neural recordings and may not necessarily be explained by differences in the biological complexity of the model. We consider that the 3D nature of the culture may be more adaptive to changes in neural activity due to its complex cytoarchitecture. Another possibility is that the BrainSphere model may be less sensitive due to the inability of compounds to penetrate the center of the spheroid. However, BrainSpheres are ~ 300 µM in diameter, and, nutrient diffusion issues have not been observed in spheroids smaller than 500 µM in diameter (McMurtrey 2016; Takebe et al. 2014). Therefore, we believe that inadequate diffusion of chemicals to the core of the BrainSpheres is an unlikely explanation for the lower sensitivity. Lower cytotoxicity detection in the BrainSphere MEA assay compared to the rNFA may be explained by several factors, such as reduced uptake and reduction of resazurin dye due to cell compactness (Walzl et al. 2014) or perhaps due to differing number of BrainSpheres per well. Future work to evaluate cytotoxicity detection limits of the resazurin assay or alternative cytotoxicity detection methods for the BrainSphere model should be considered.

Differences in sensitivity may also be explained by biological differences between the assays. For example, the maturity of the cells may play a role in functional differences, with the chemical exposure in the rNFA starting on postnatal day 0 (the day the rat cortical neurons are harvested and plated) compared in the BrainSphere MEA in which the chemical exposure started on 7-week-old iPSCs. Moreover, although the two models comprised mostly similar cell types, the functional role of the cells may be different in each cell model due to differences in cell–cell or cell–environment interactions. The rNFA was observed to be more sensitive compared to another human MEA model, the human neural network formation assay (hNNF) (Bartmann et al. 2023), which comprised of a similar co-culture of iPSC-derived excitatory and inhibitory neurons, and primary human astrocytes (described in more detail below). These findings together indicate that species differences cannot be ruled out as a contributing factor, suggesting that the human cell model may be more resistant to chemical perturbation compared to a rat model. We also consider reproducibility of cellular composition and function between BrainSpheres. Our findings indicate functional variability between biological replicates and minimal differences from immunohistochemical readouts (data not shown); however, additional work is needed to fully characterize reproducibility across experiments, cell line, and laboratories. Improving variability in controls, screening of additional compounds, and evaluation of model reproducibility will provide insight into whether these two MEA models may provide complementary readouts for neurotoxicity hazard evaluation.

In an effort to compare the 3D BrainSphere MEA assay to a 2D hNNF assay (Bartmann et al. 2023), we compared bioactivity results for deltamethrin (the only chemical that was tested in both assays). In the hNNF, the 2D cultures matured on an MEA plate for 7 days prior to chemical exposure, at which time the chemical was added, and activity was recorded weekly for 28 days (24 h prior to recordings a washout was performed). The hNNF co-culture was similarly derived from a human iPSC-based model but did not include oligodendrocytes. In comparing the mean potency of deltamethrin from both assays, Bartmann and collaborators reported a mean potency of 3.53 µM, while we observed 4.45 µM in our 3D cultures. In addition, the minimum potency was 2.74 µM in the hNNF versus 1 µM in our assay, indicating similar estimated potencies. Despite differences in the cell models, plating time, exposure window and length, results from these two assays appear to be relatively comparable for this particular chemical. In the future, a more extensive comparison of human 2D vs 3D cultures on MEAs should be performed to understand the relevance of using 3D cultures instead of classic monolayers for this assay. It could also be valuable to generate a 3D rat NNF model to study the contribution of the 3D structure to the assay itself.

Ongoing assay evaluation and refinement, such as increasing number of replicates, implementation of baseline activity filtering, improvement of BrainSphere adhesion to the HD-MEA plate, and continued optimization of neural metric data acquisition parameters, will continue to improve the robustness of the assay for chemical screening. Moreover, testing of a larger and diverse chemical set, targeting neuro-relevant pathways, will permit comparisons with other NAMs and further inform the added value of the BrainSphere MEA assays as a more complex, human-relevant biological system for neurotoxicity screening.

## Conclusion

In recent years, a consensus has emerged among scientists from academia, industry, and regulatory agencies worldwide on the need for a standardized in vitro testing strategy. The objective is to facilitate more cost-effective and swift generation of data for toxicological evaluations that also incorporates biology that is relevant to humans. The data presented herein demonstrate that the BrainSphere MEA assay with further improvements could be developed into a viable screening assay that could either be used as a primary screen or in conjunction with the rNFA as part of a tiered screening approach where hits in the rNFA are confirmed and further characterized in the human BrainSphere assay (e.g., measuring axonal conductance rates, and axon tracking and/or evaluation of brain regions outside the cortex through brain region specific differentiation). This model holds the potential to minimize interspecies differences and advance towards animal-free assays. The assay was able to detect activity in both positive assay control chemicals as well as in a small set of training compounds, while negative assay control and some training compounds were without activity. While additional work needs to be done to improve the variability of the assay and to test larger numbers of chemicals, the results of this proof-of-concept study strongly support moving forward with development of this assay.

## Supplementary Information

Below is the link to the electronic supplementary material.

Supplementary file1 (XLSX 29 KB)

Supplementary file2 (PDF 1789 KB)

Supplementary file3 (PDF 290 KB)

Supplementary file4 (JPG 814 KB)

Supplementary file5 (PDF 589 KB)

## Data Availability

Supplementary tables containing key data are provided with this publication. The full raw dataset is available from the authors upon reasonable request.

## Abbreviations

DOE: Day of exposure
DNT: Developmental neurotoxicity
EFSA: European Food Safety Authority
EPA: Environmental Protection Agency
FDA: Food and Drug Administration
HD-MEA: High-density microelectrode array
HTS: High-throughput screening
iPSC: Induced pluripotent stem cell
MEA: Microelectrode array
MFR: Mean firing rate
NAM: New approach methodologies
NEM: Neural expansion medium
NIEHS: National Institute of Environmental Health Sciences
NDM: Neuronal differentiation medium
NPC: Neural progenitor cell
NFA: Network formation assay
OECD: Organisation for Economic Cooperation and Development
rNFA: Rat network formation assay
ROI: Region of interest
