# Supplementary figures and images for: Application of a high-density microelectrode array assay using a 3D human iPSC-derived brain microphysiological system model for in vitro neurotoxicity screening of environmental compounds
Source: Arch Toxicol. 2025 Apr 28;99(7):2917–35. doi: 10.1007/s00204-025-04043-x (PMC12198282; doi:10.1007/s00204-025-04043-x)

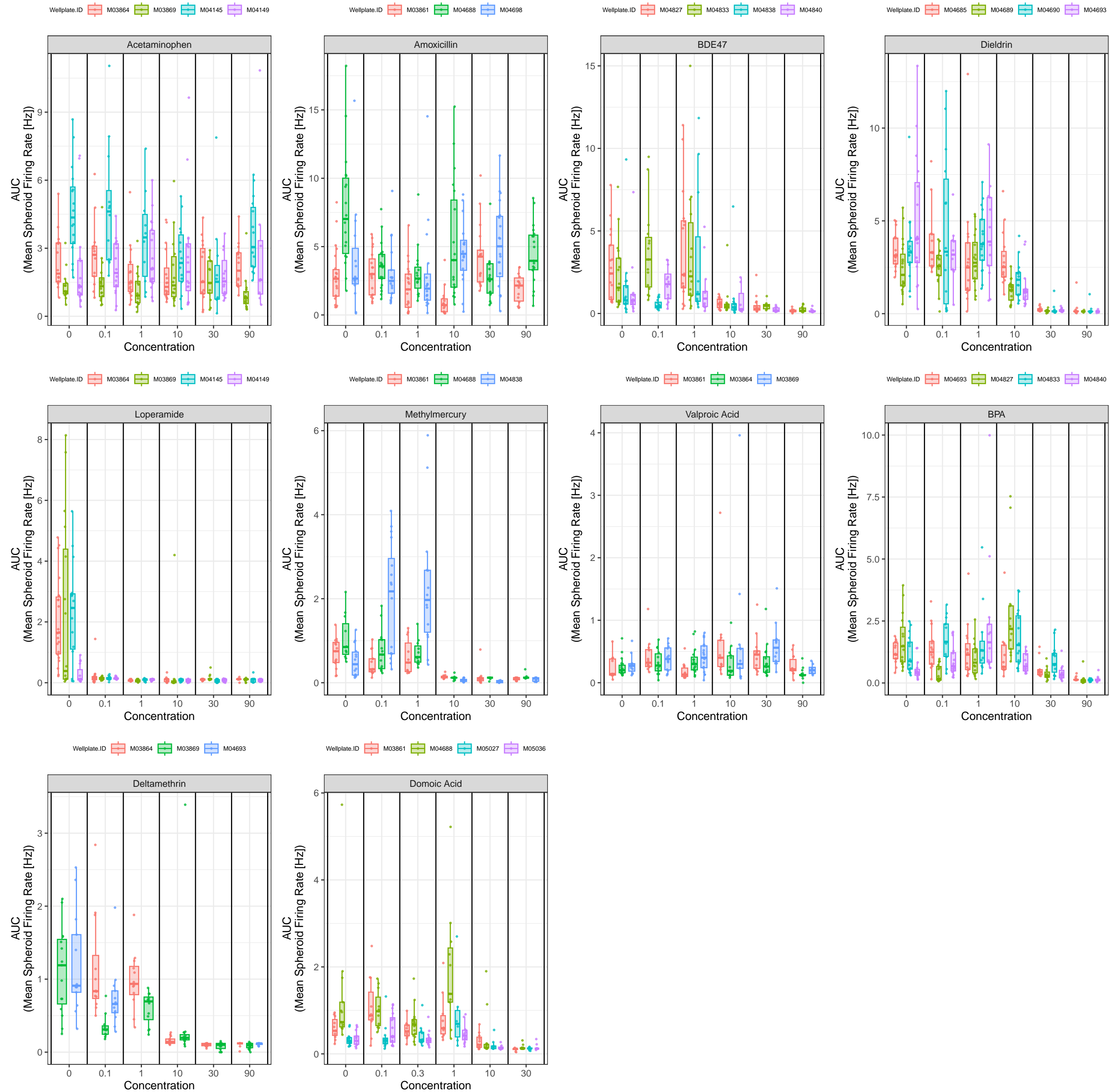

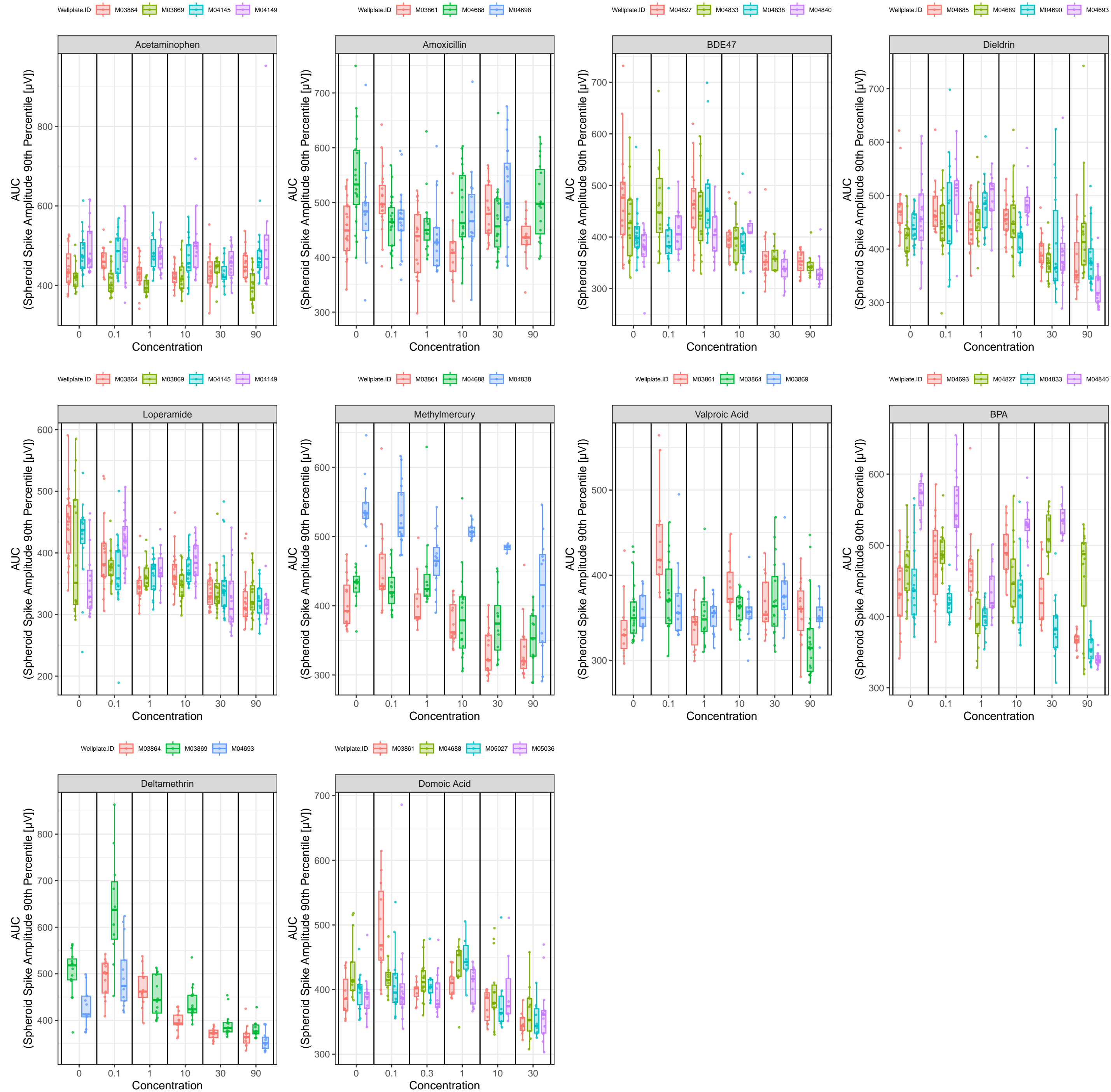

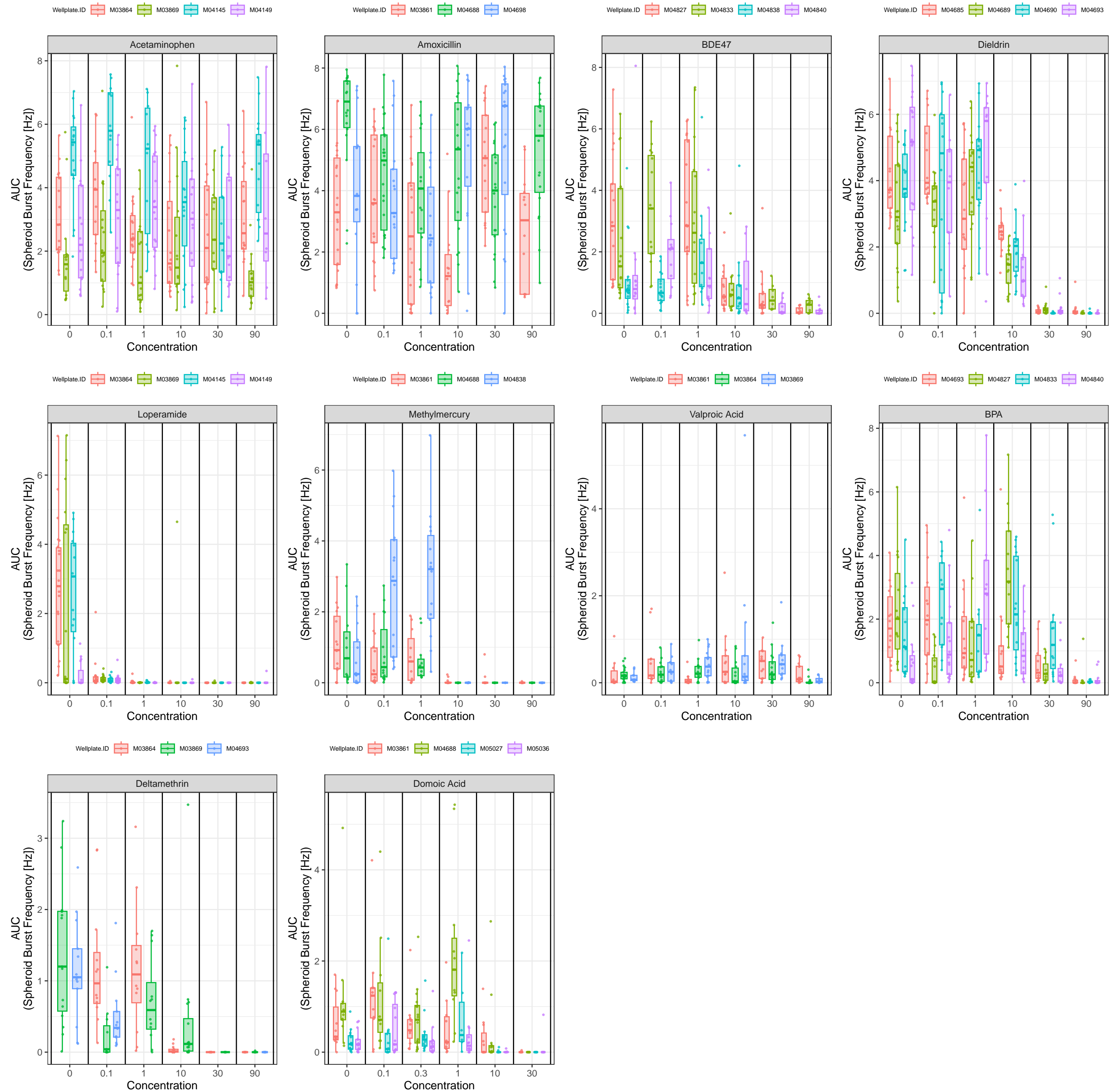

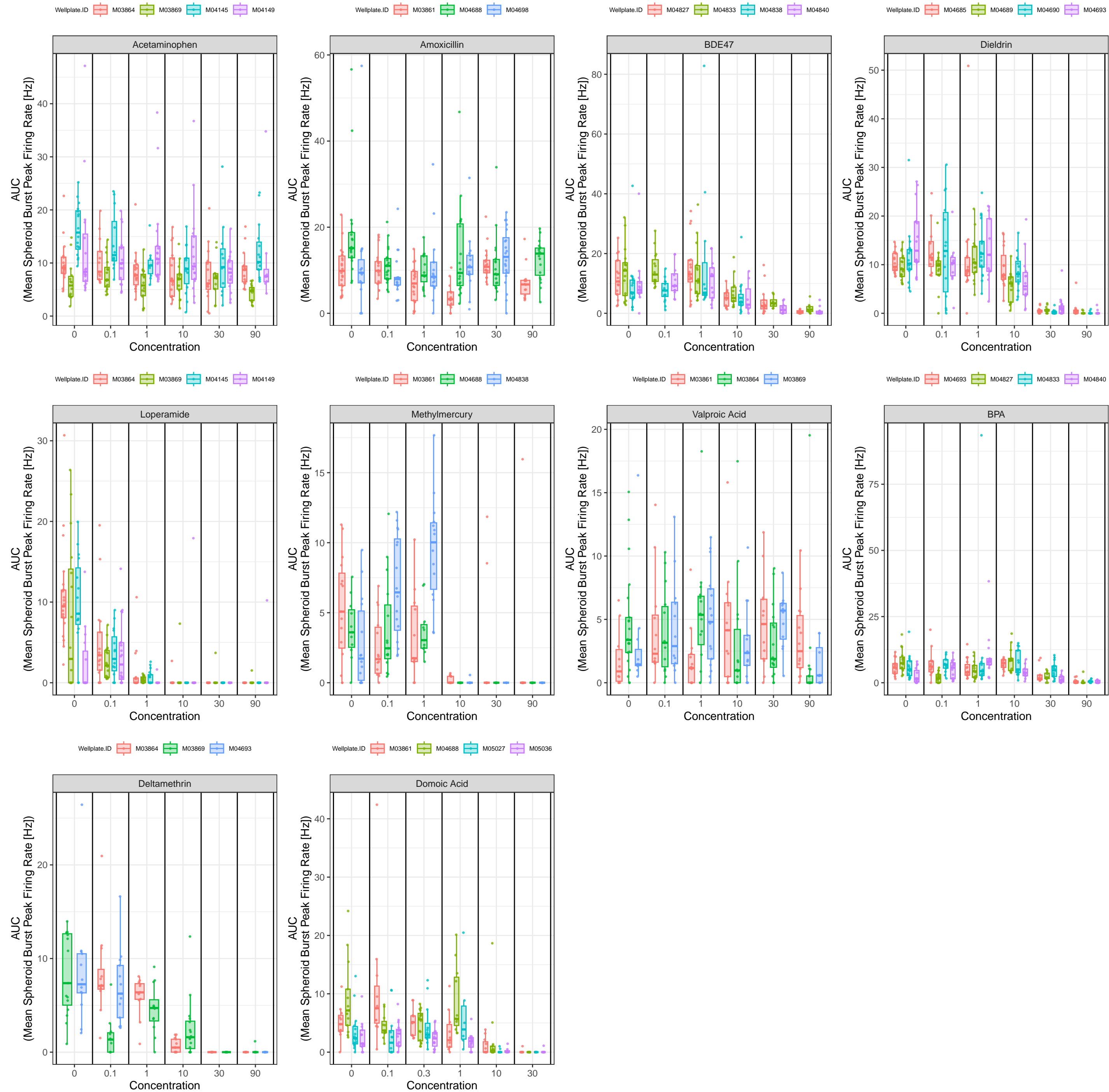

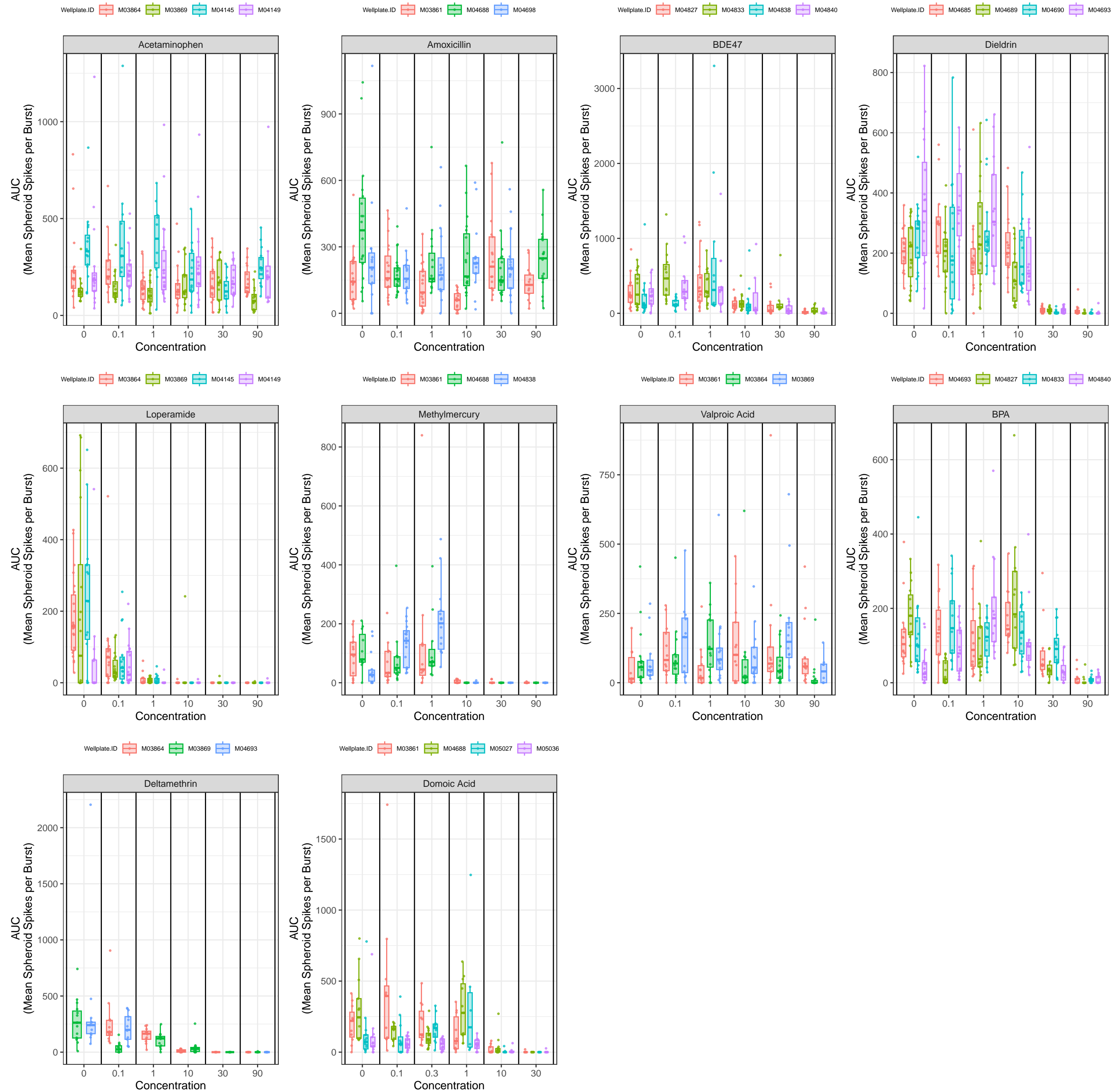

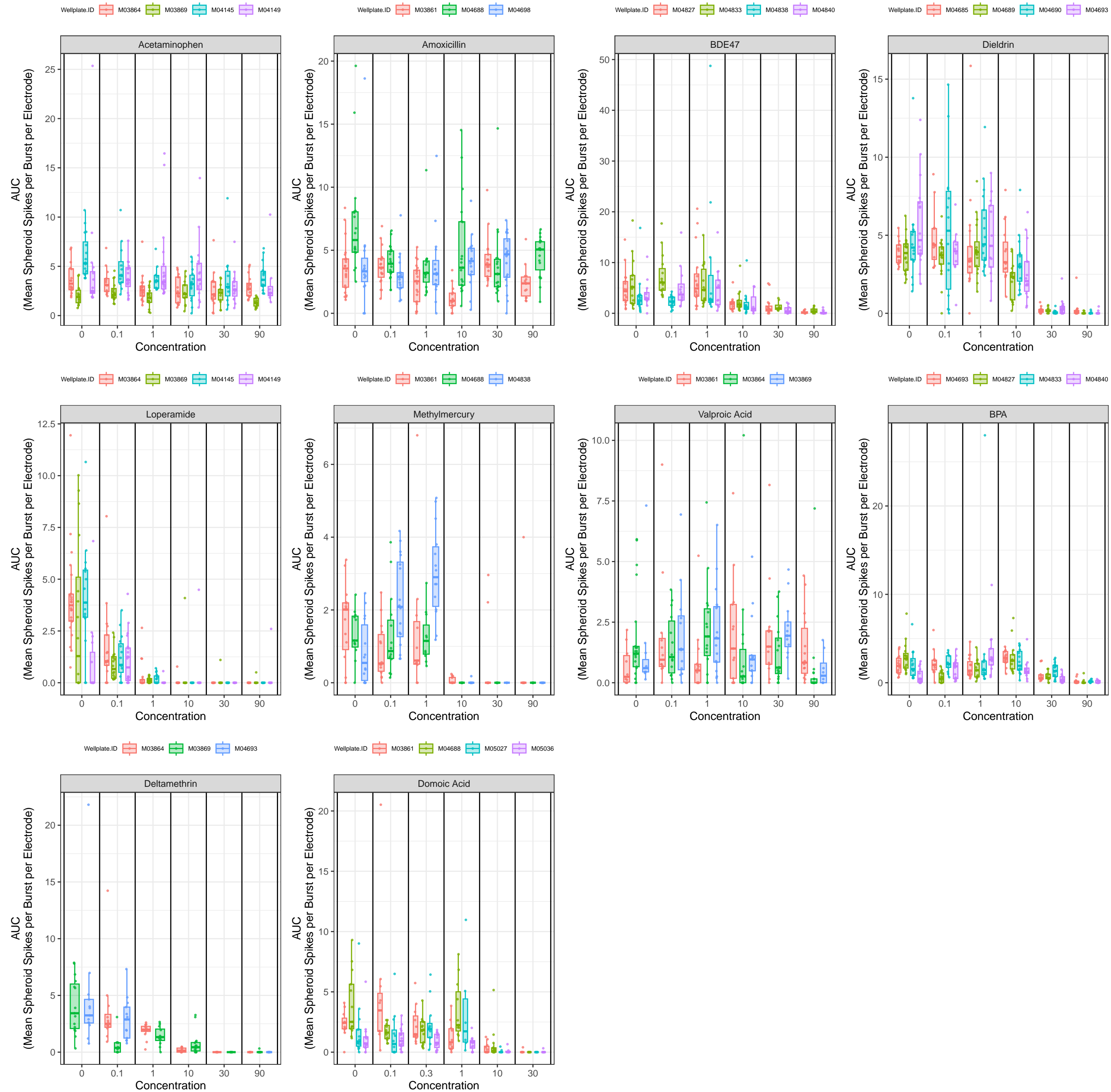

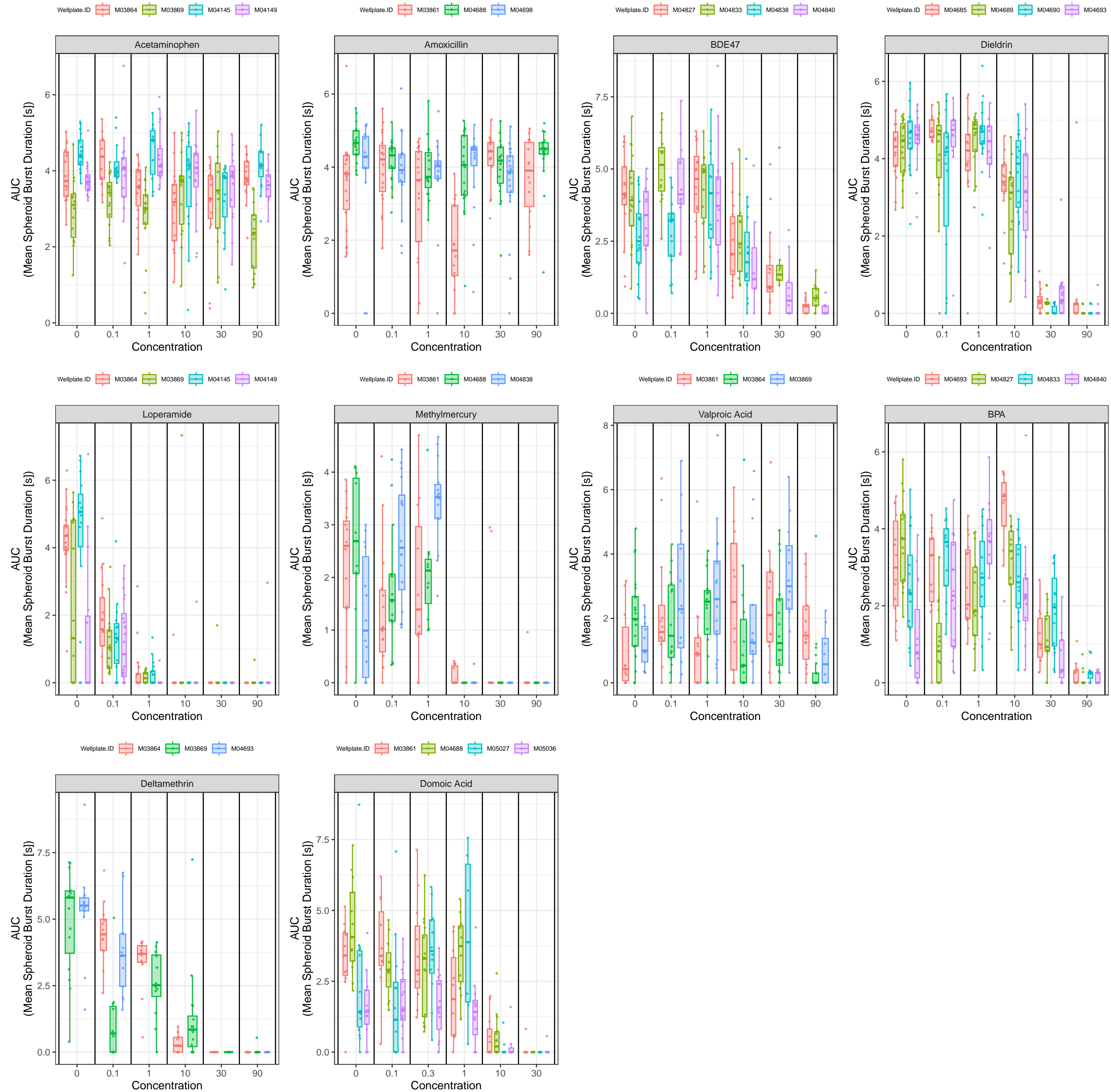

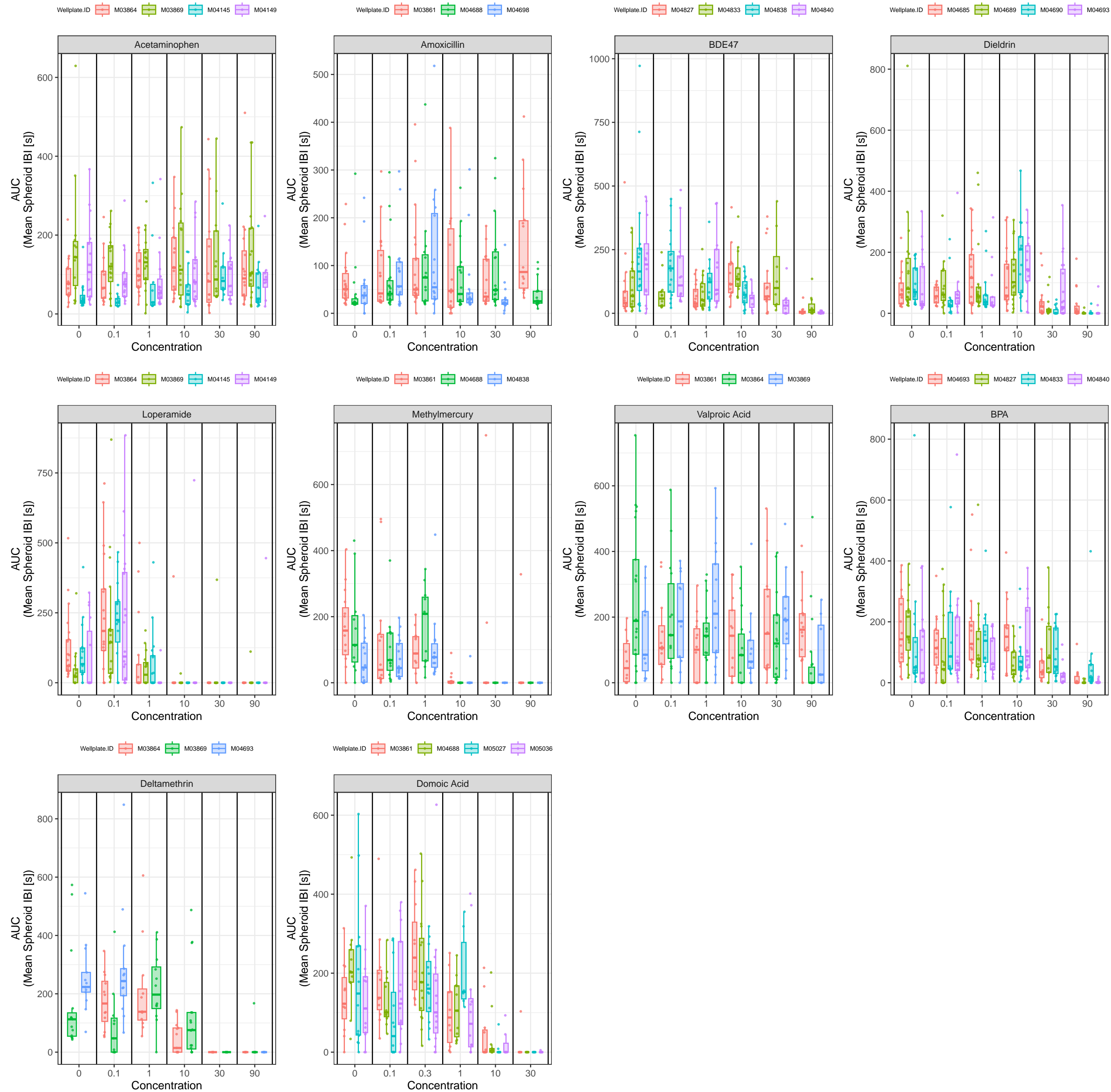

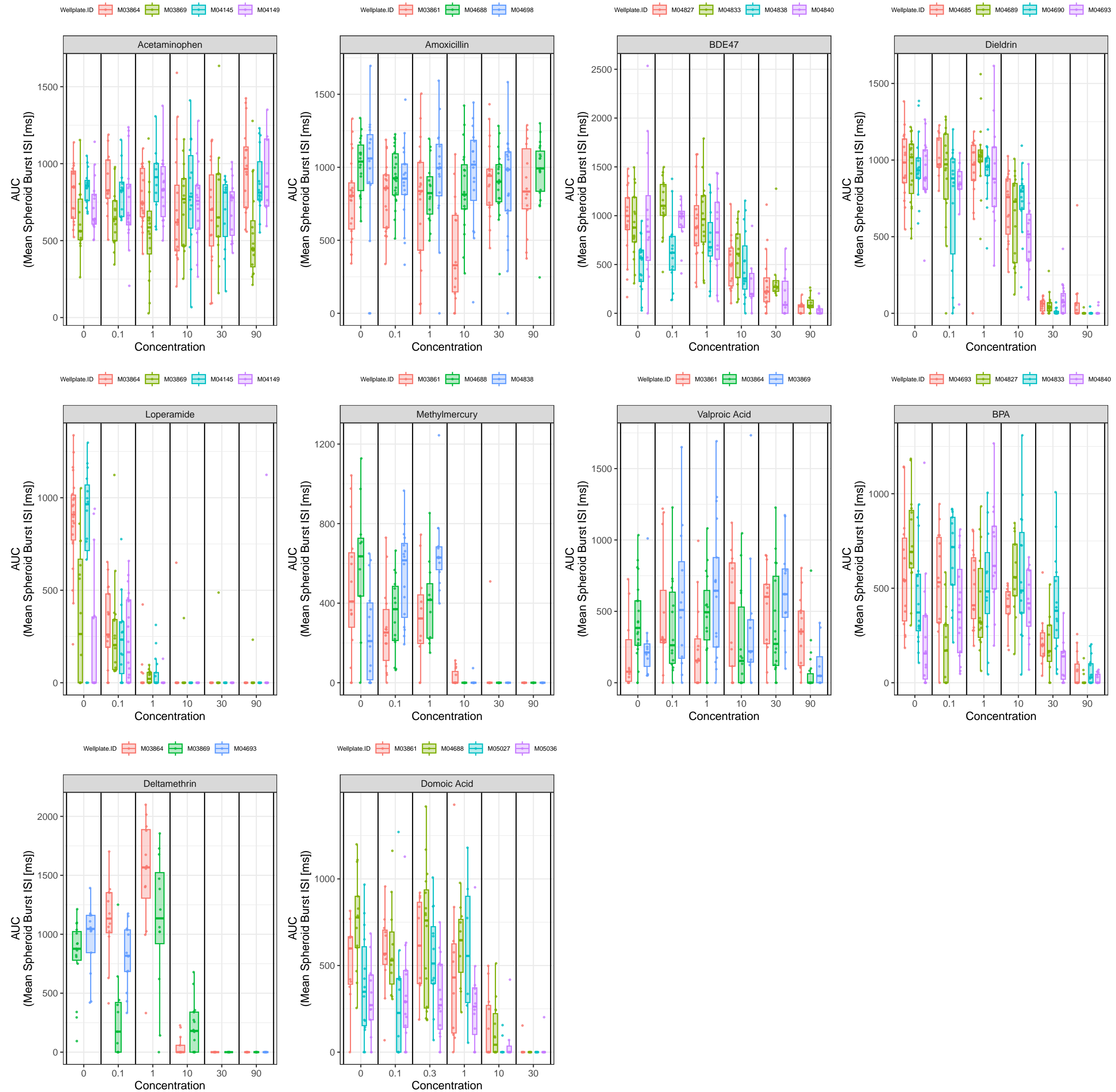

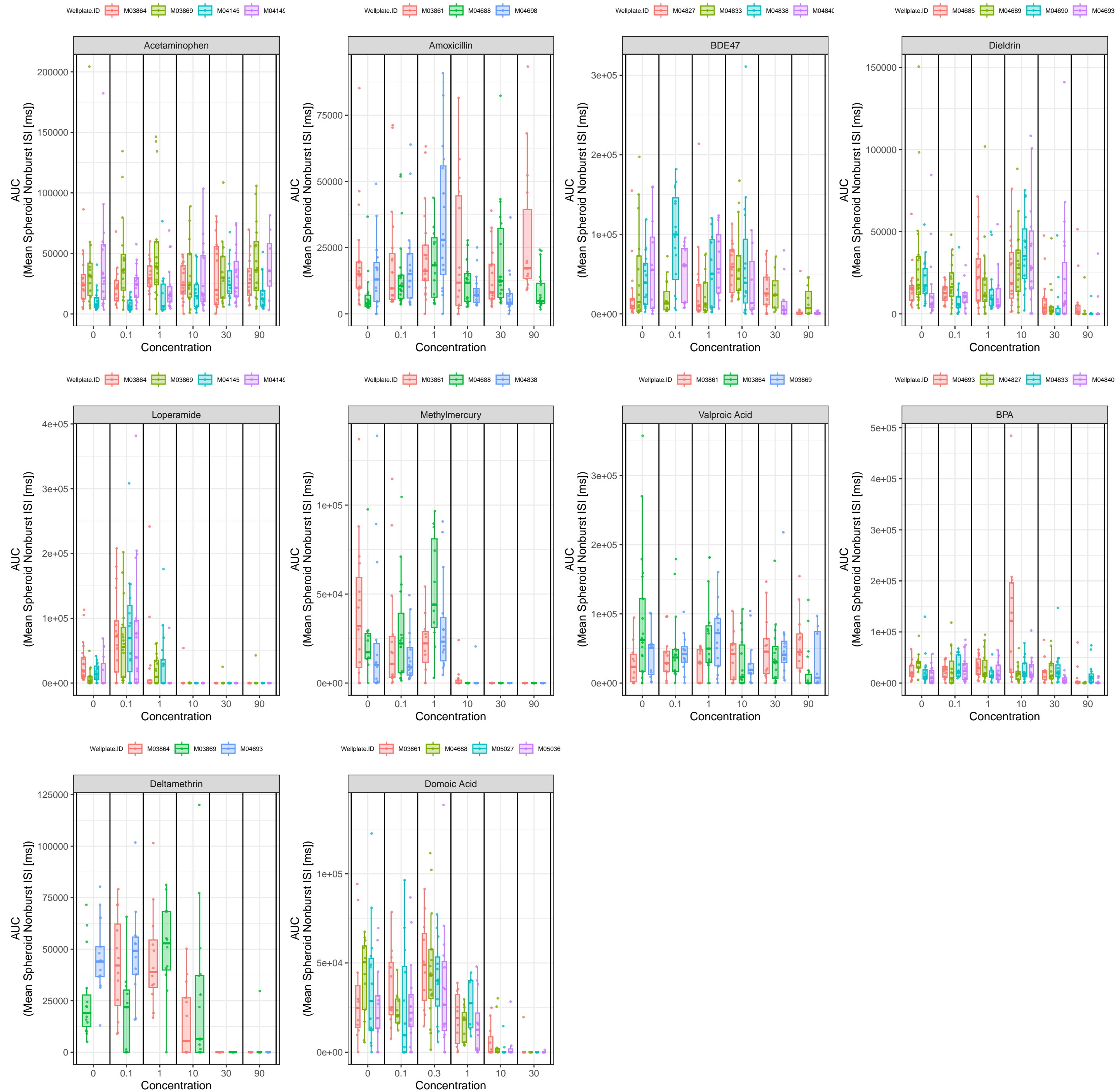

Supplement: Supplementary file 2 — Supplementary file2 (PDF 1789 KB) [file 204_2025_4043_MOESM2_ESM.pdf]

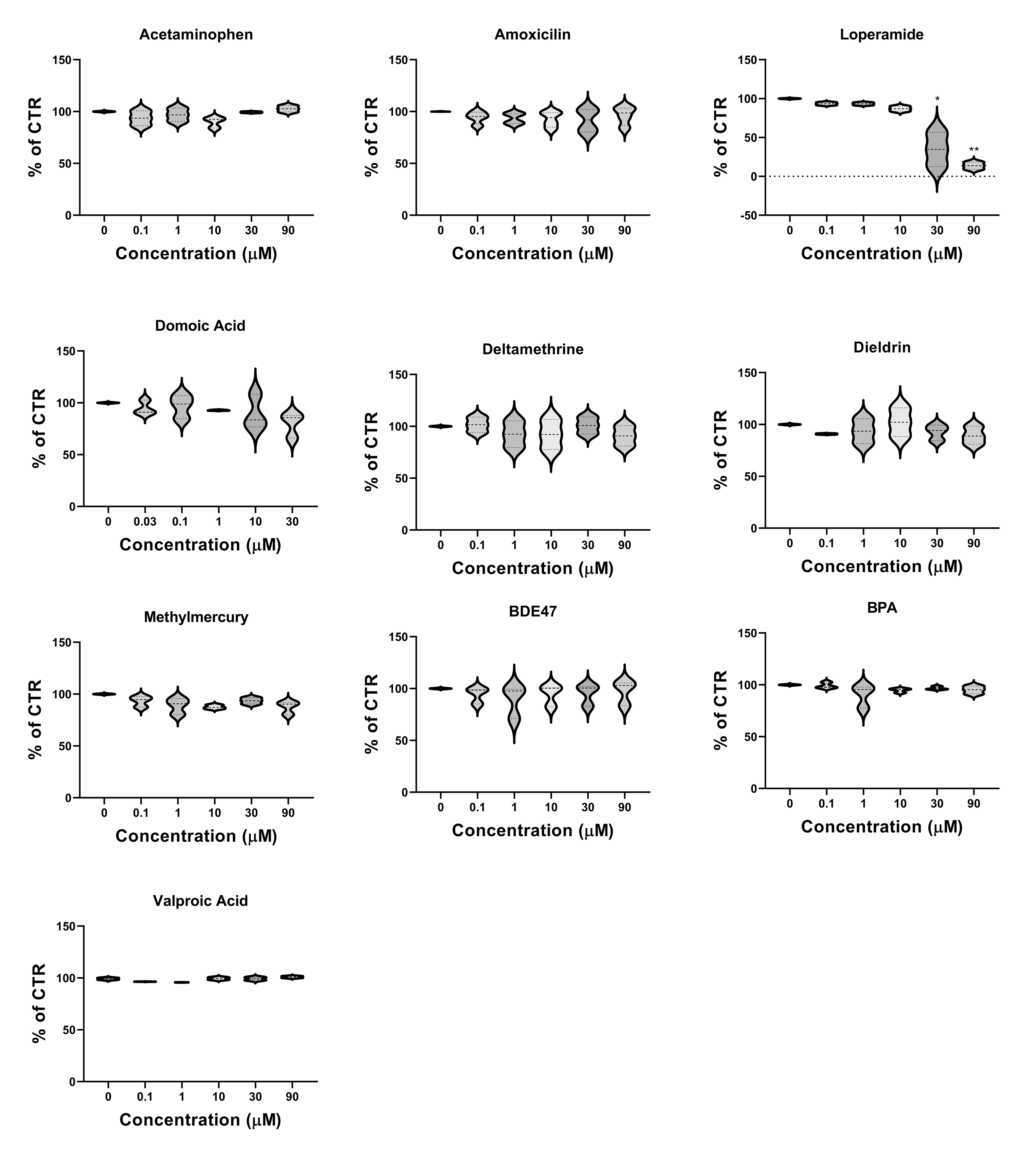

Supplement: Supplementary file 4 — Supplementary file4 (JPG 814 KB) [file 204_2025_4043_MOESM4_ESM.jpg]

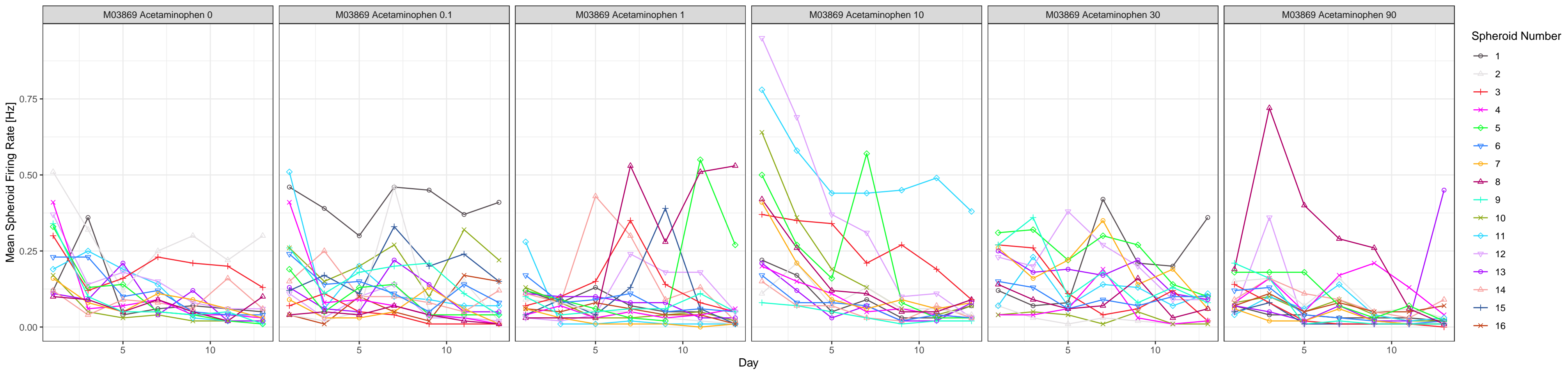

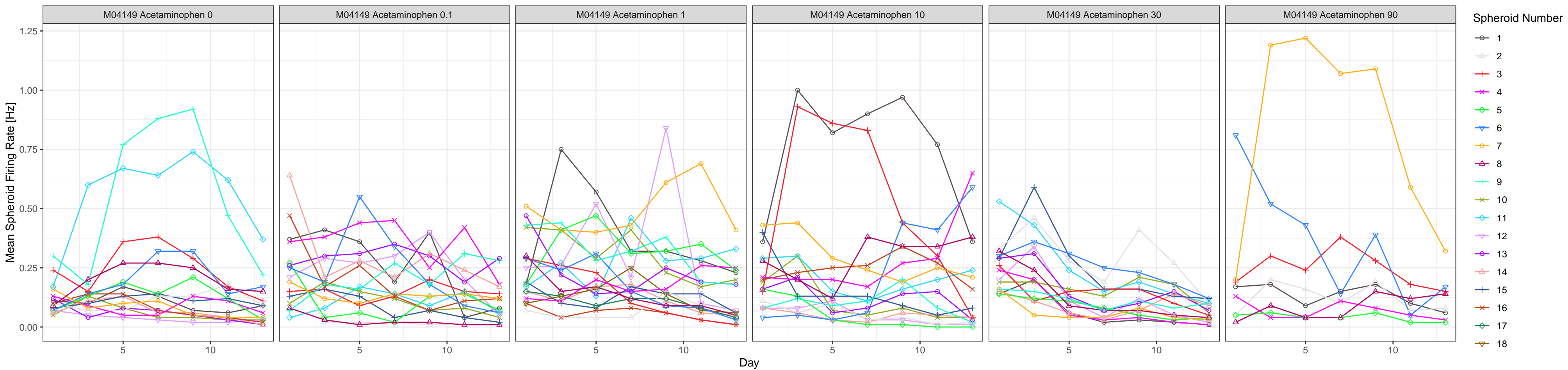

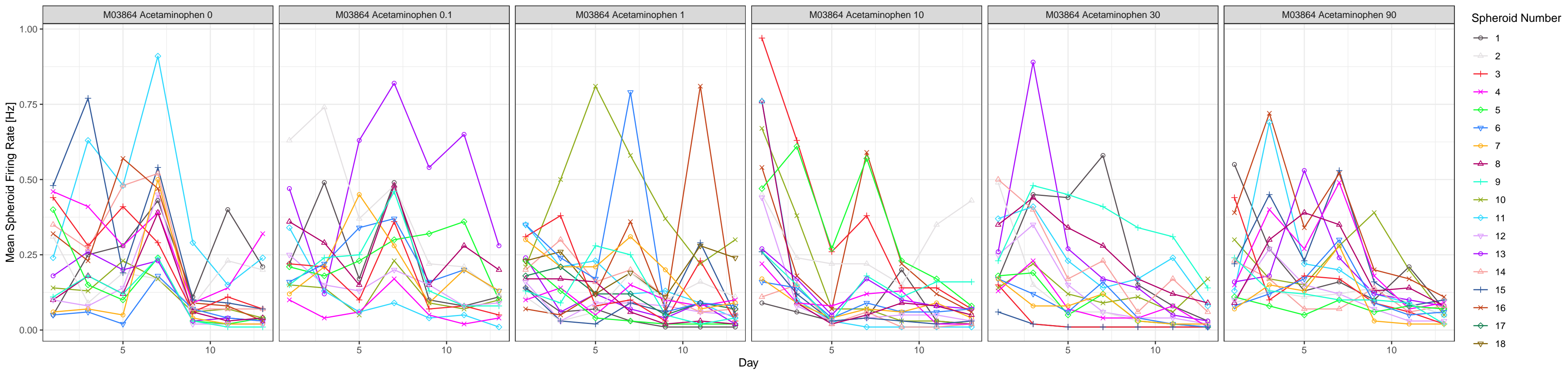

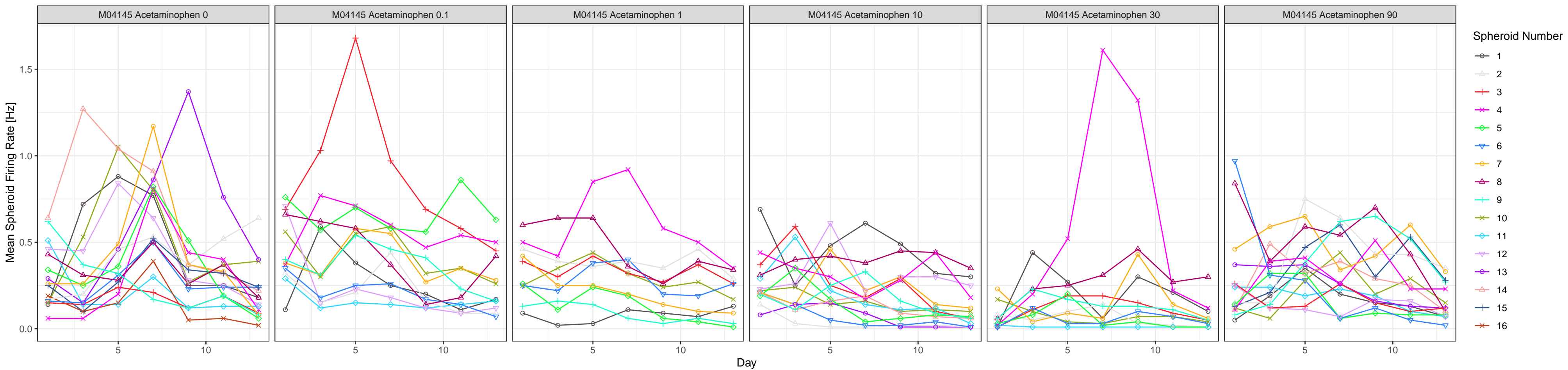

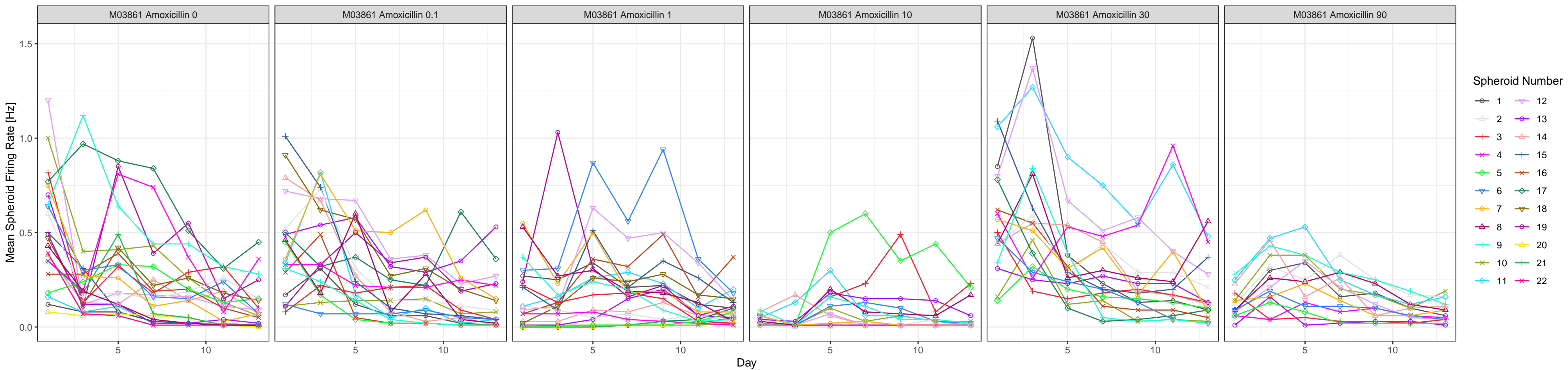

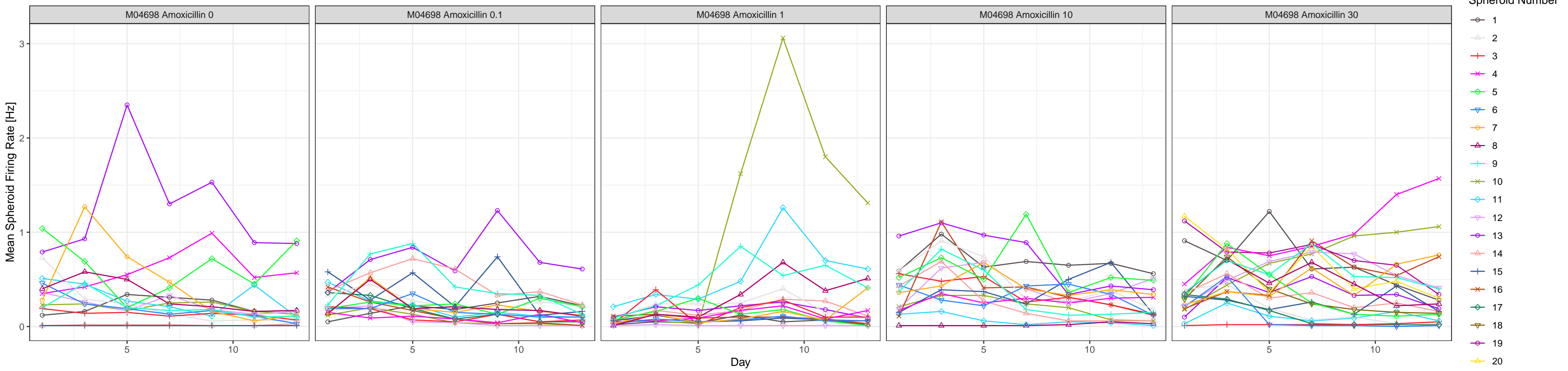

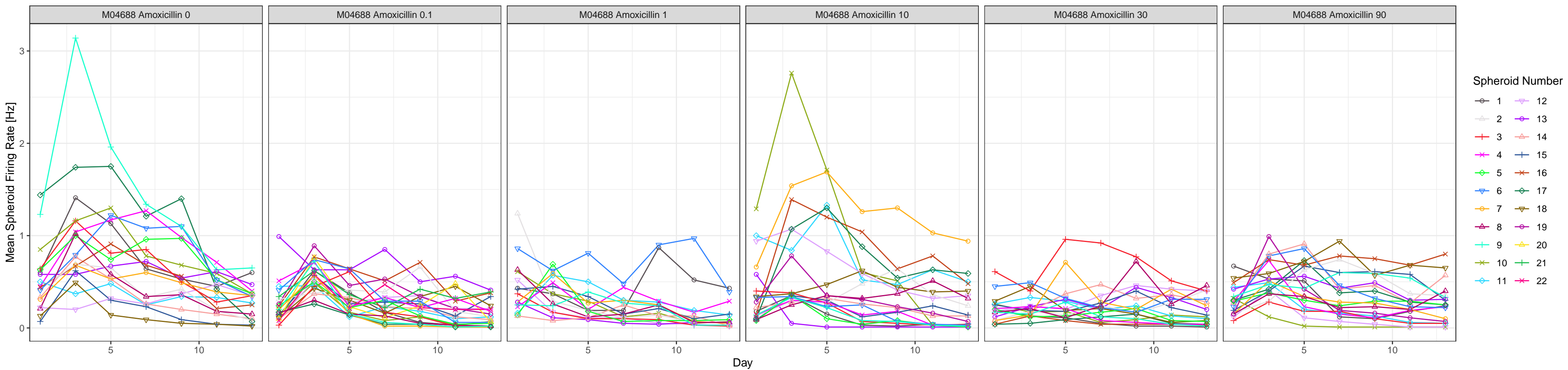

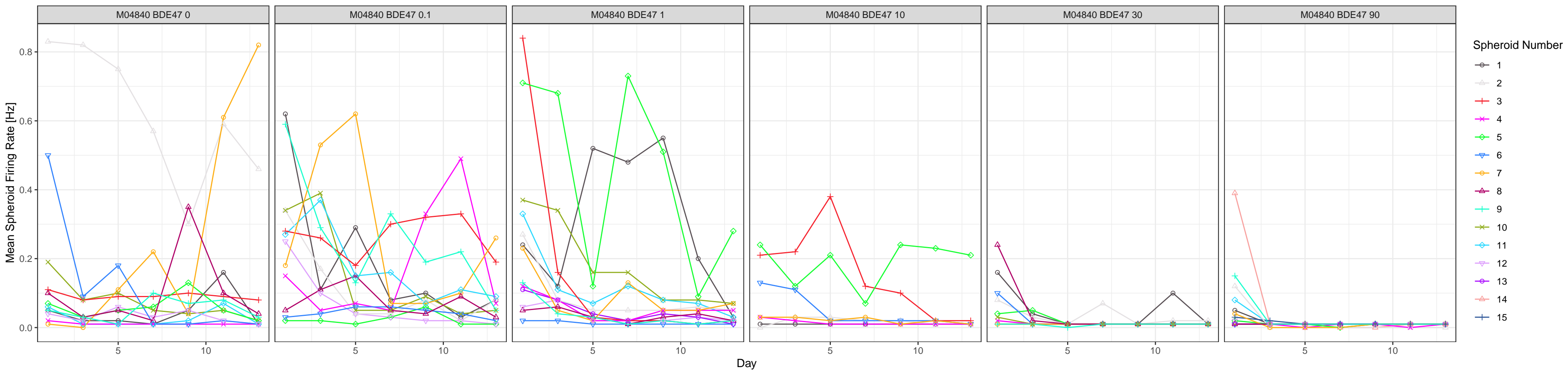

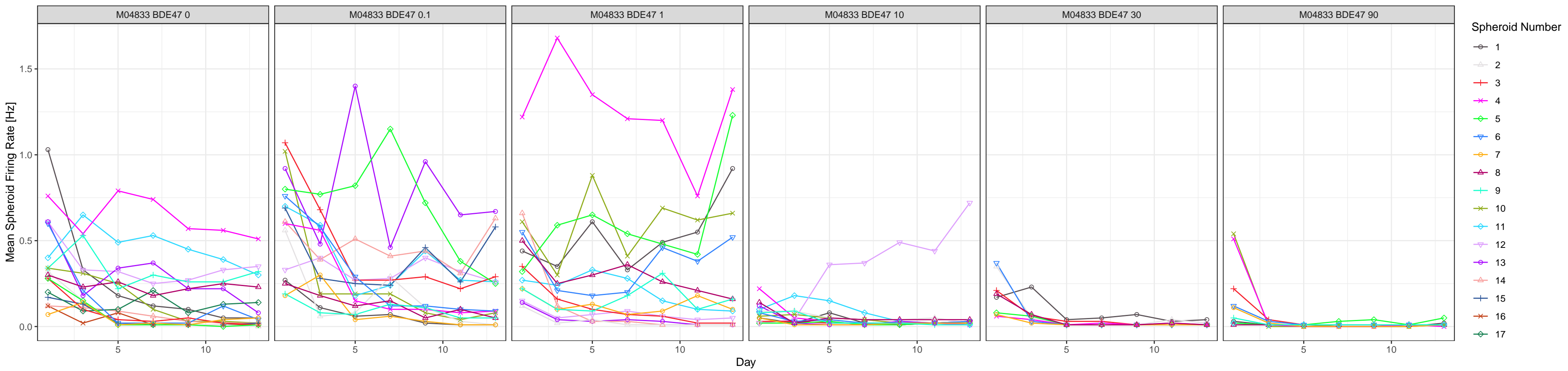

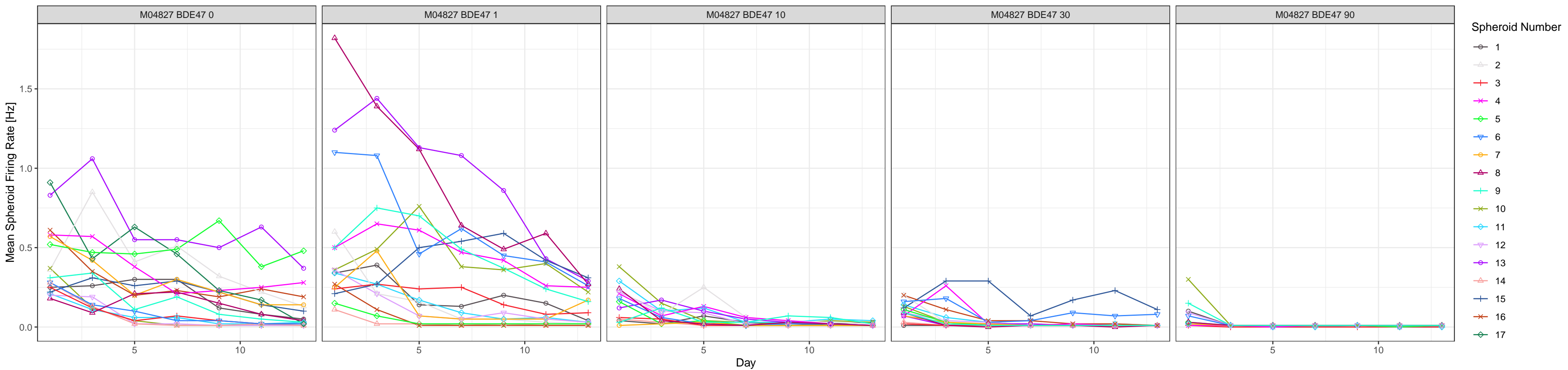

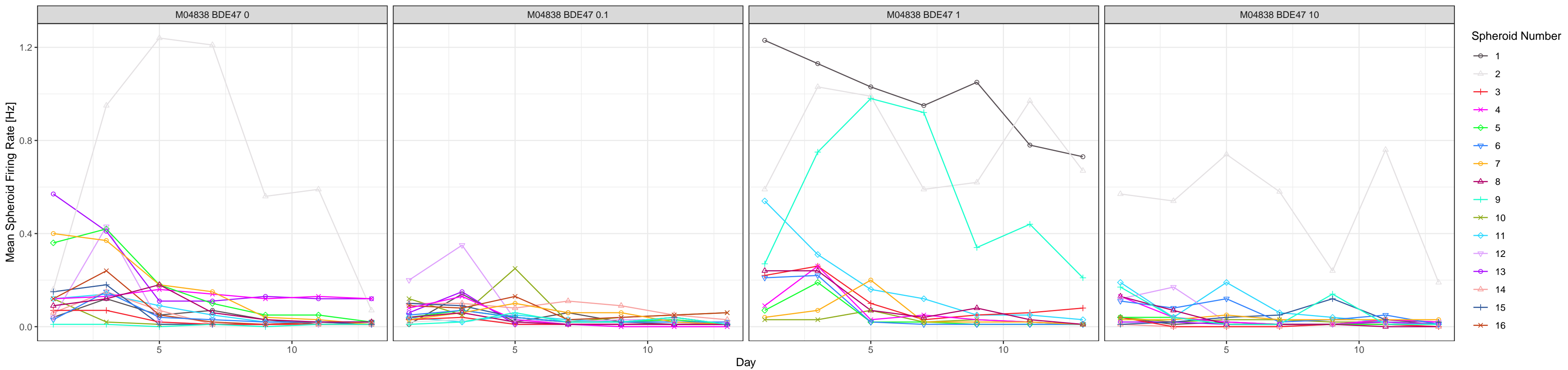

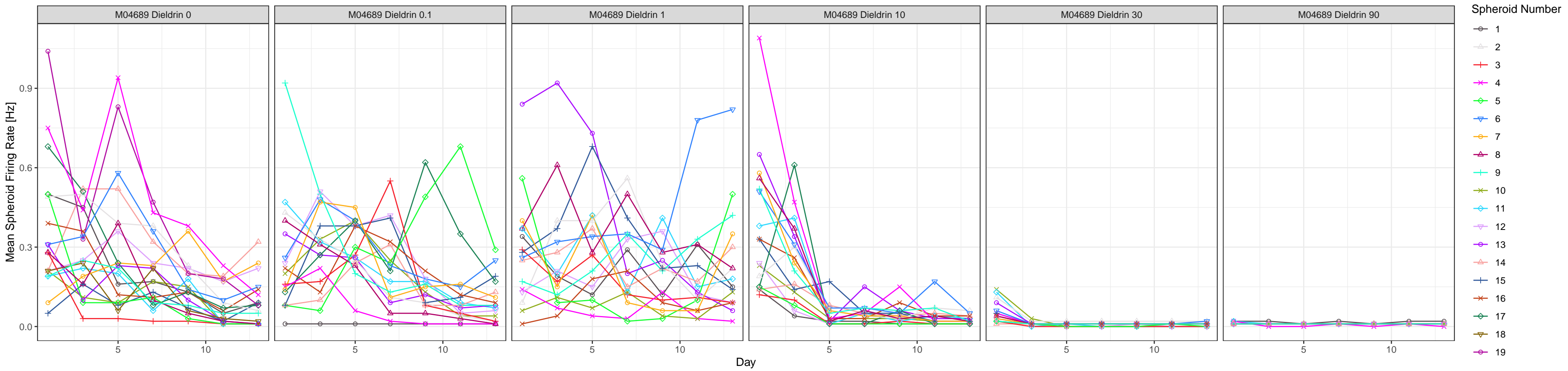

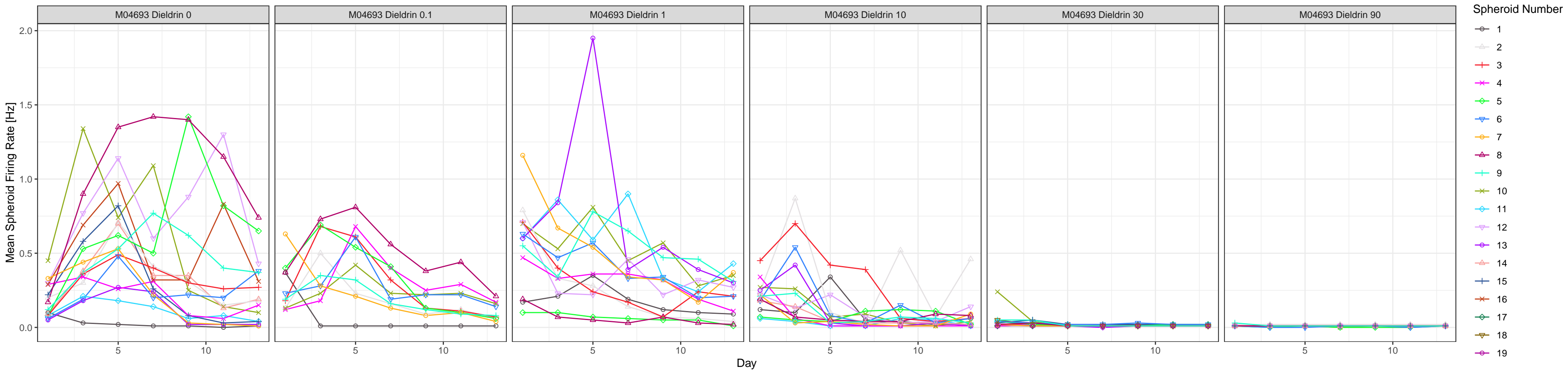

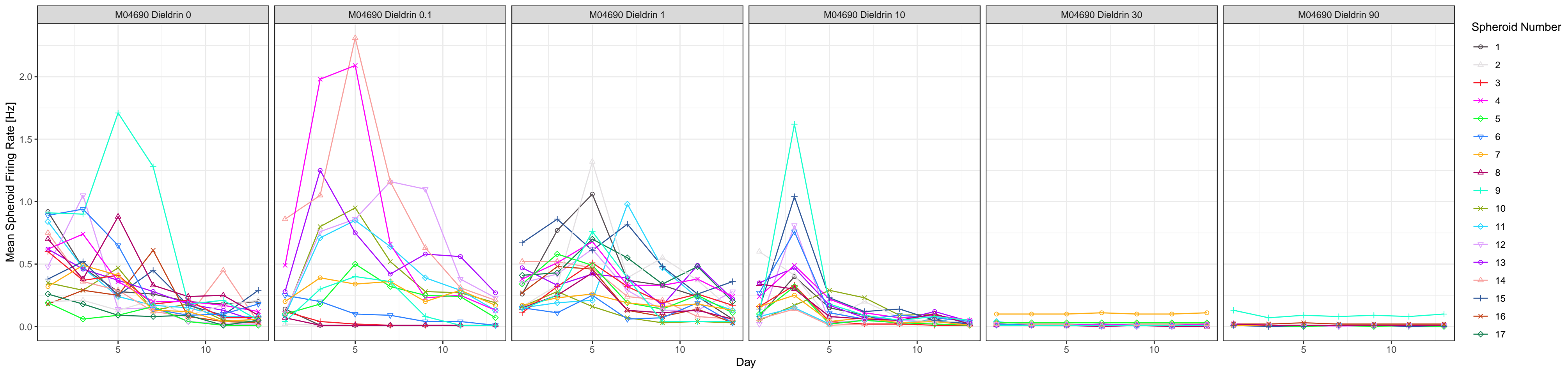

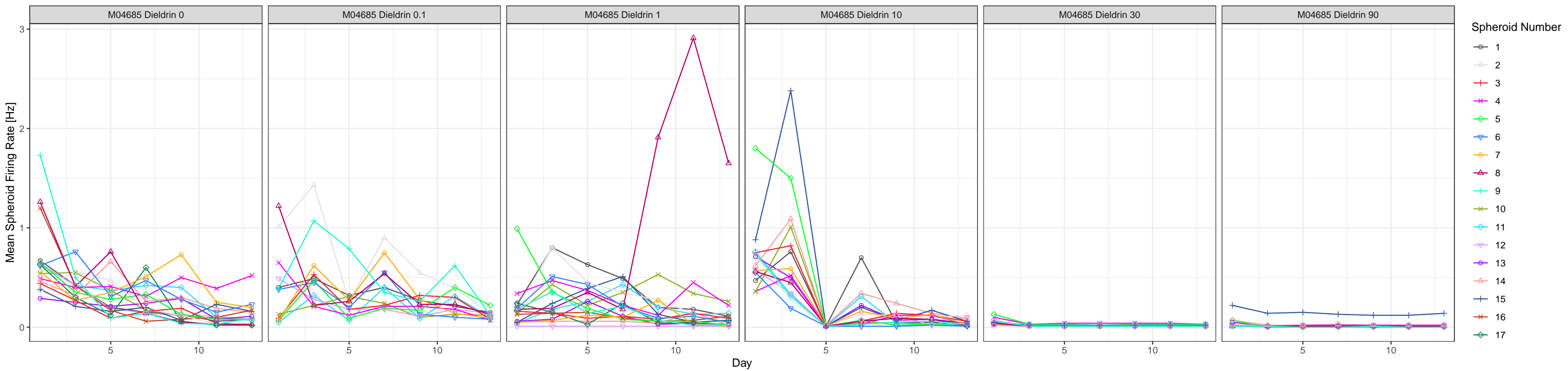

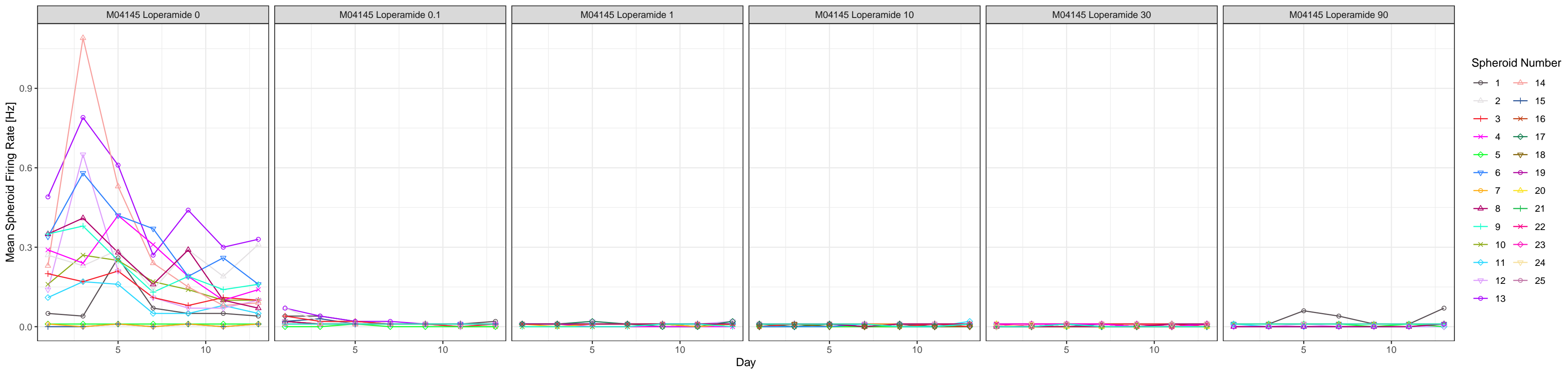

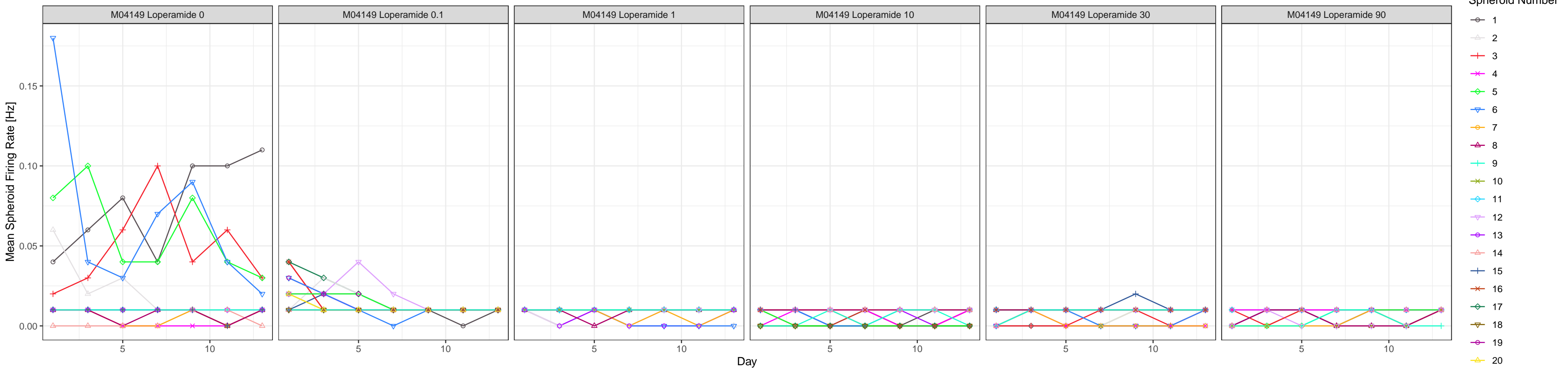

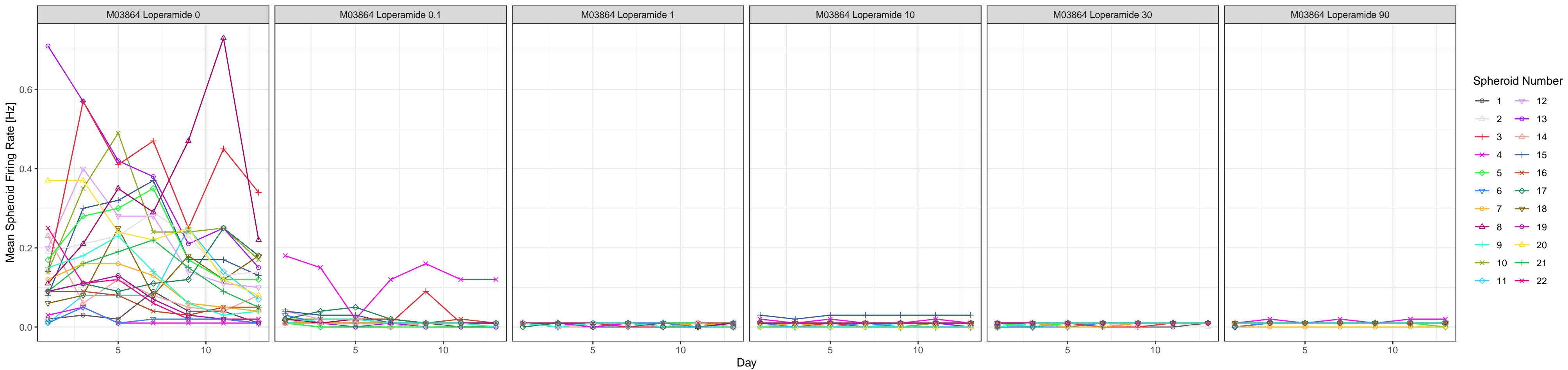

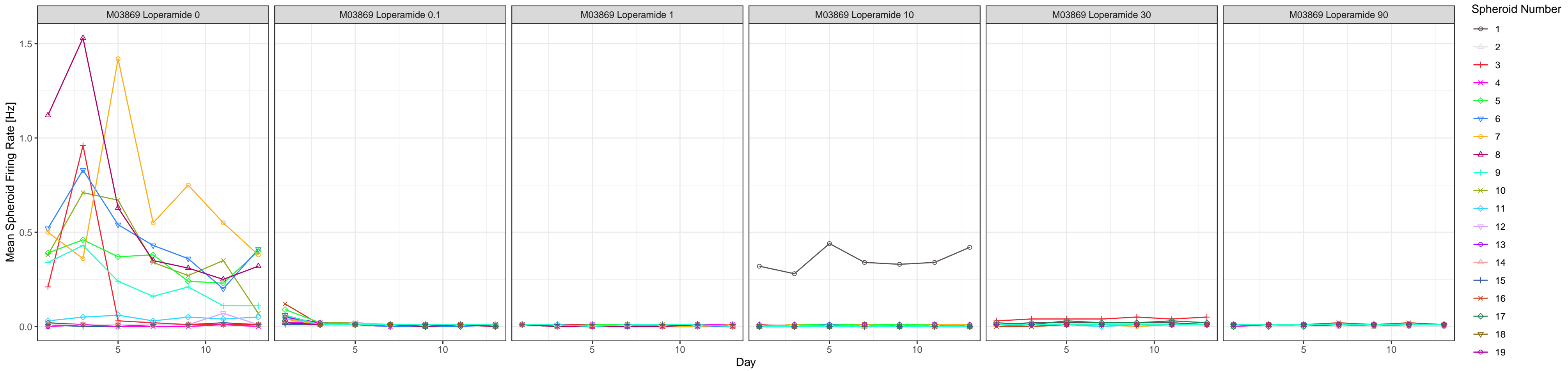

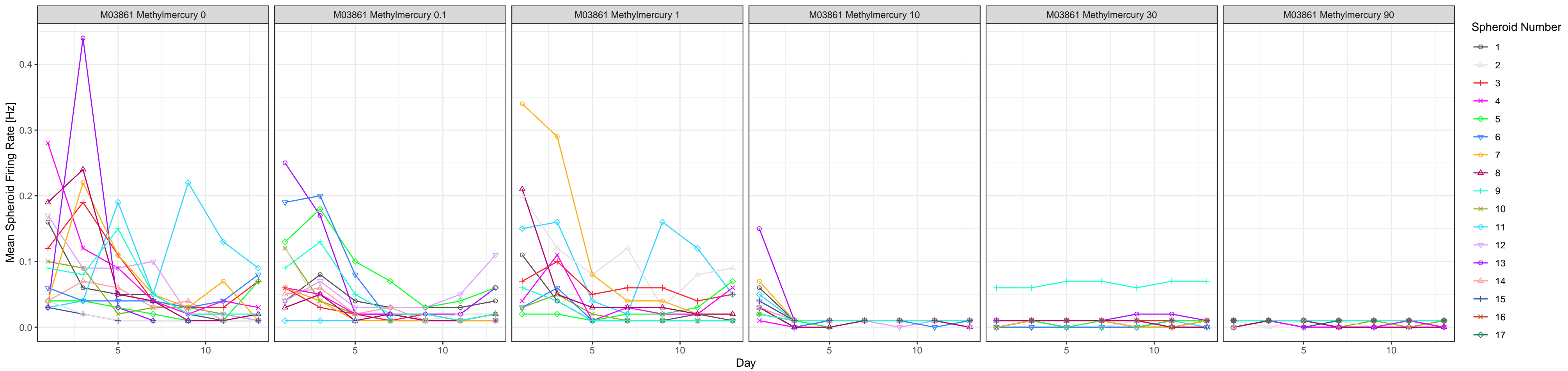

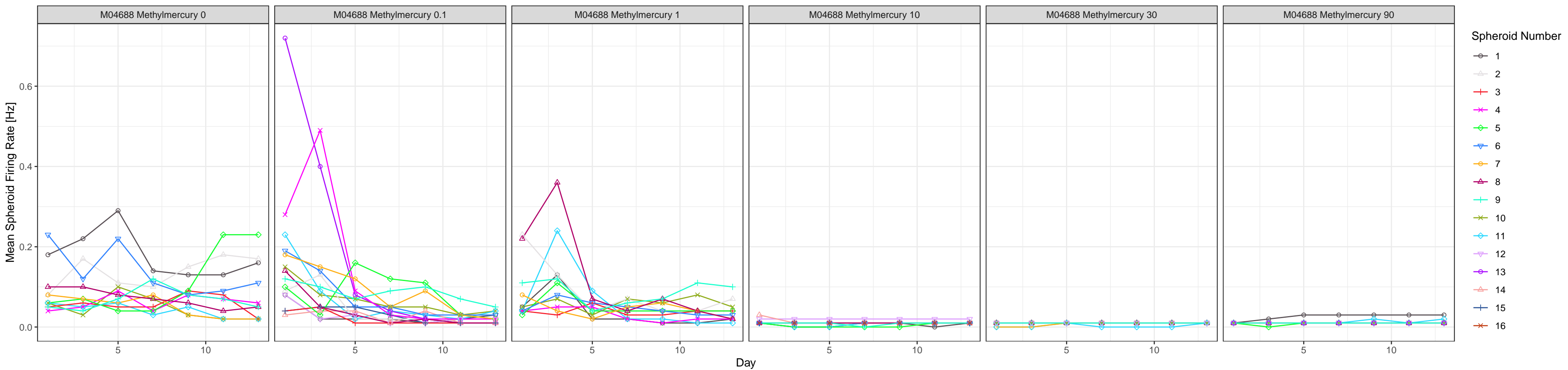

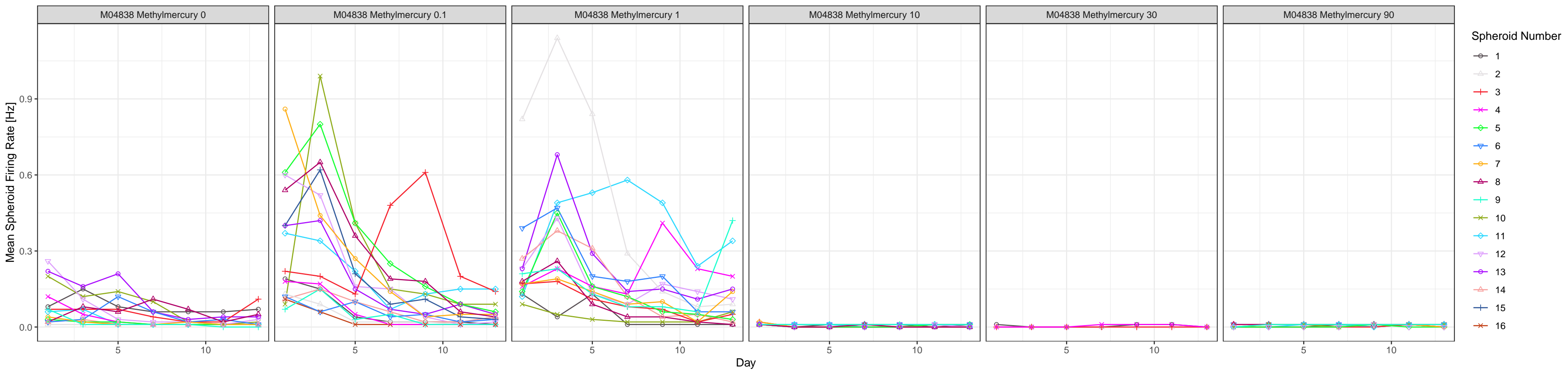

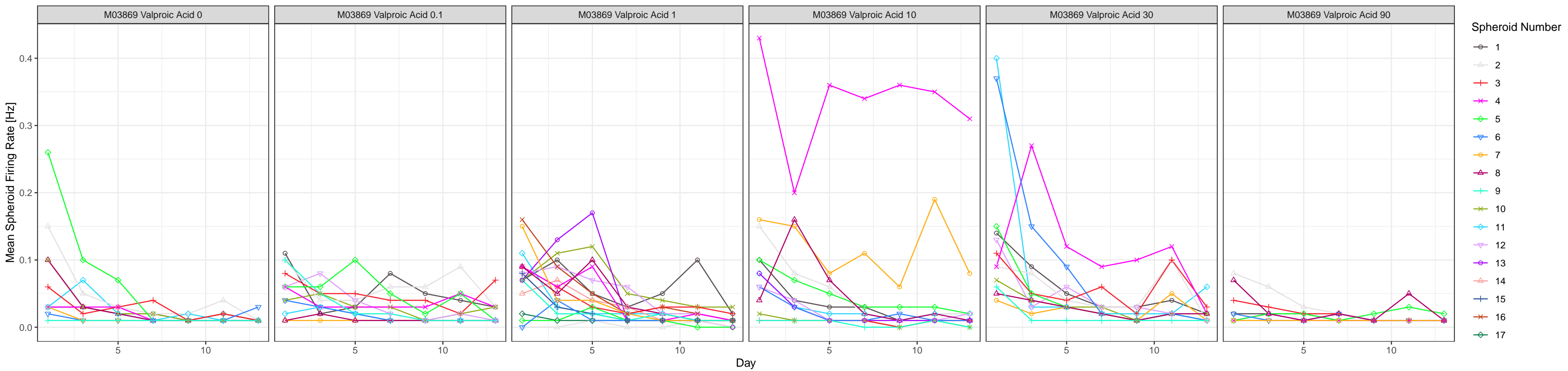

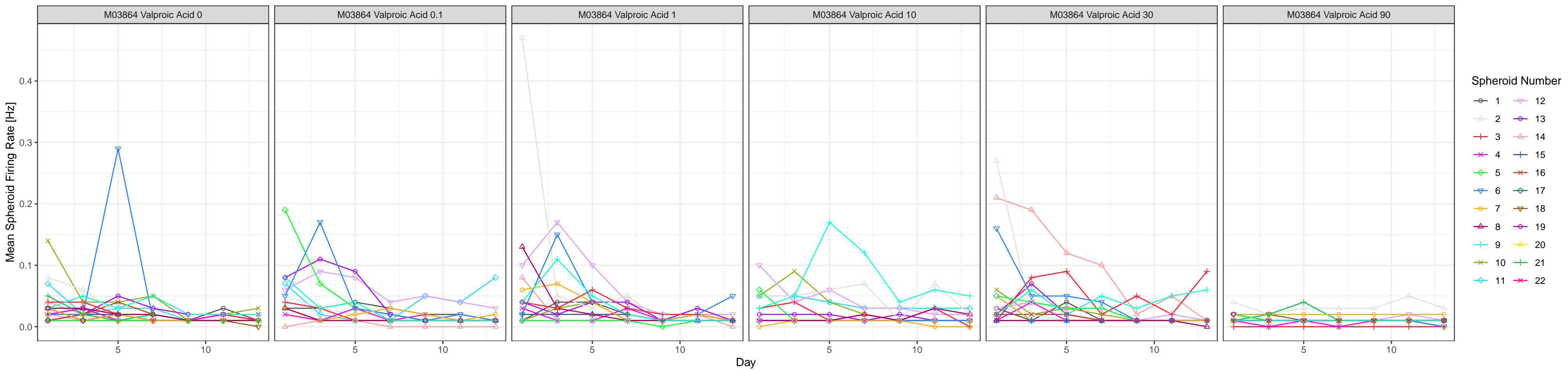

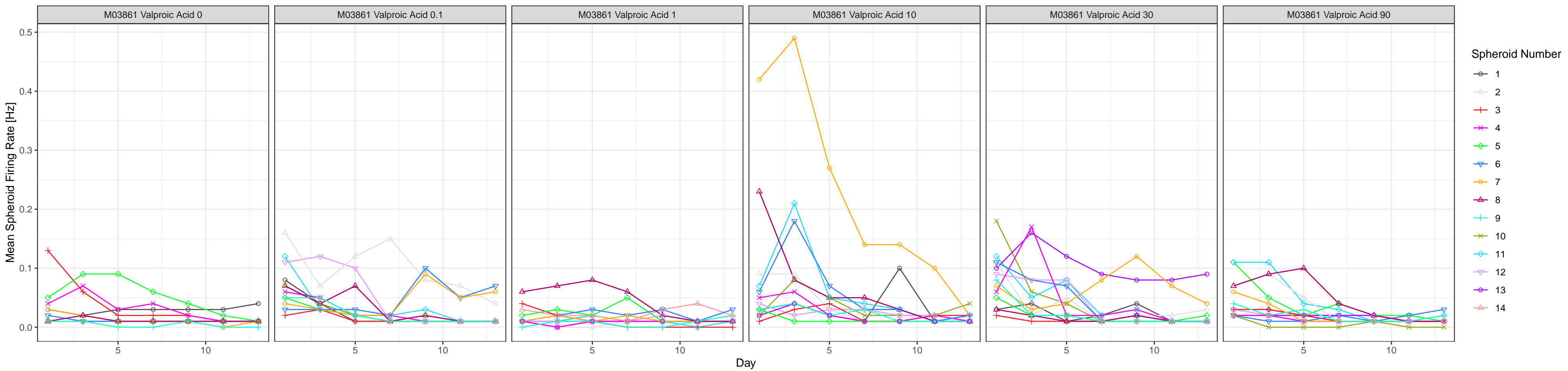

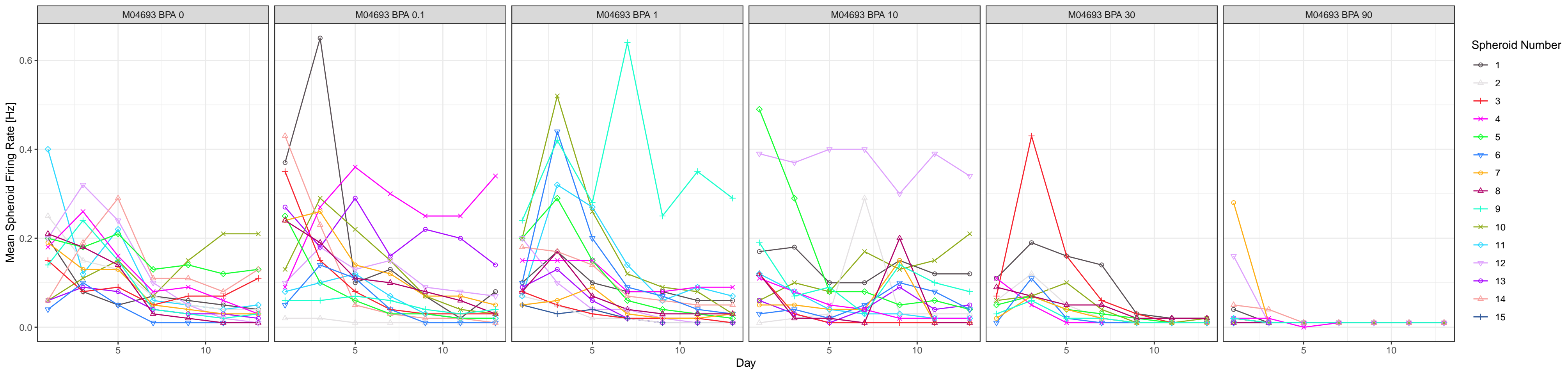

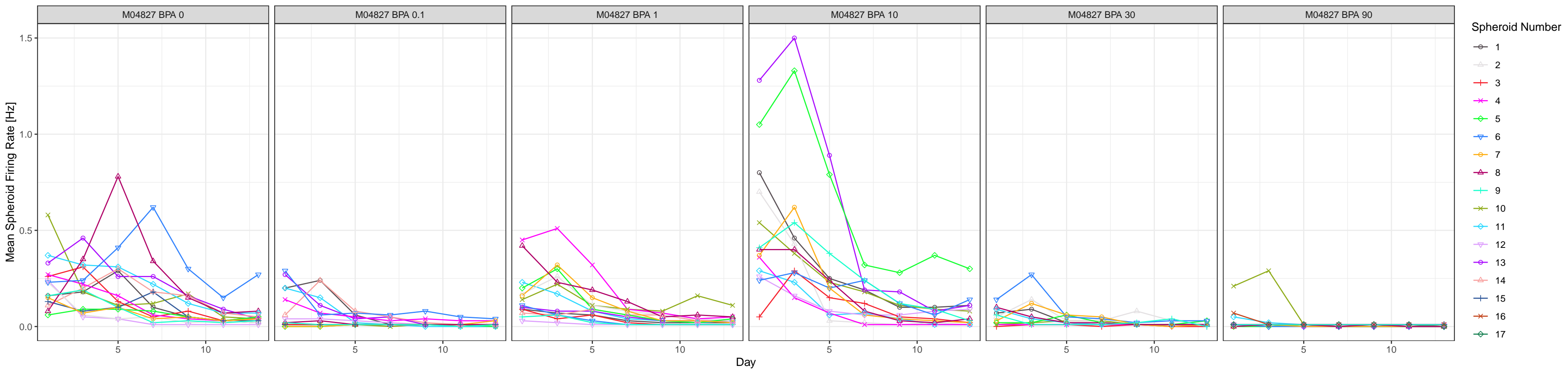

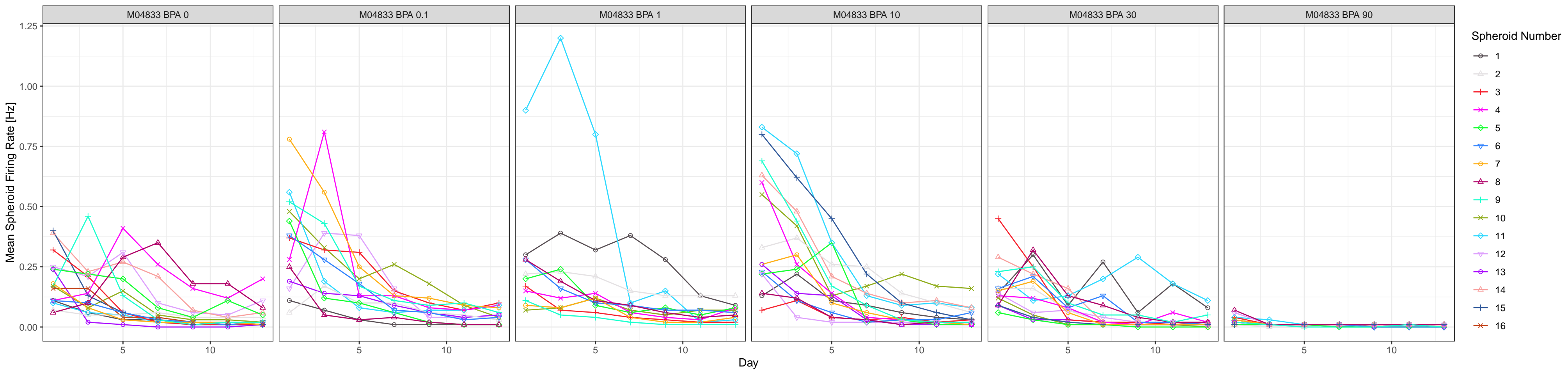

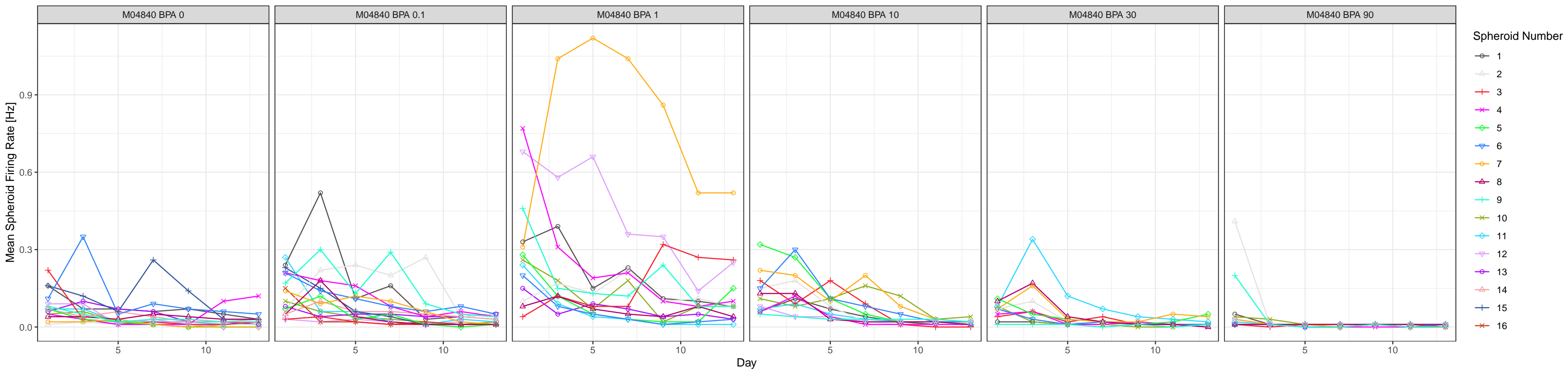

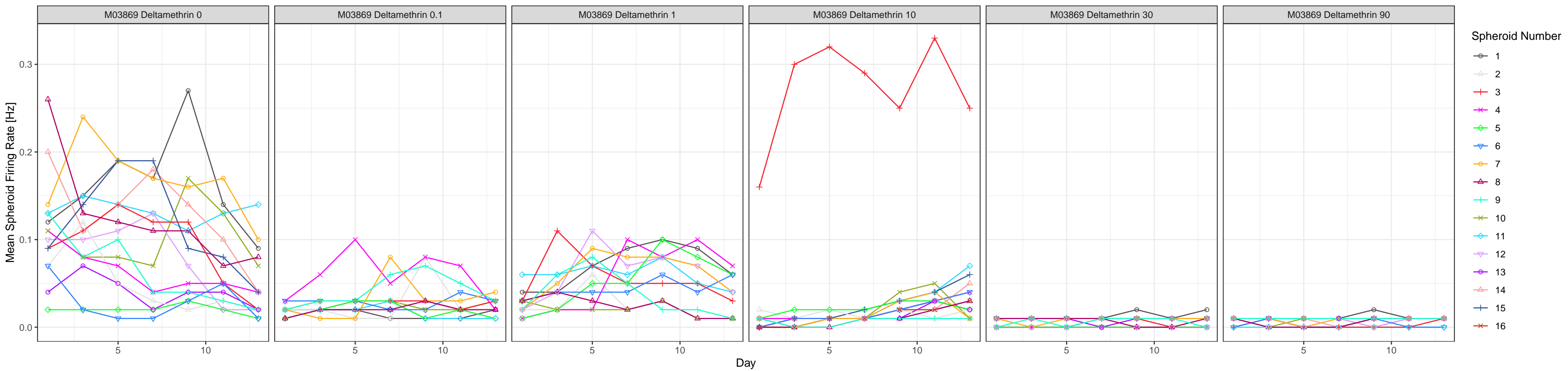

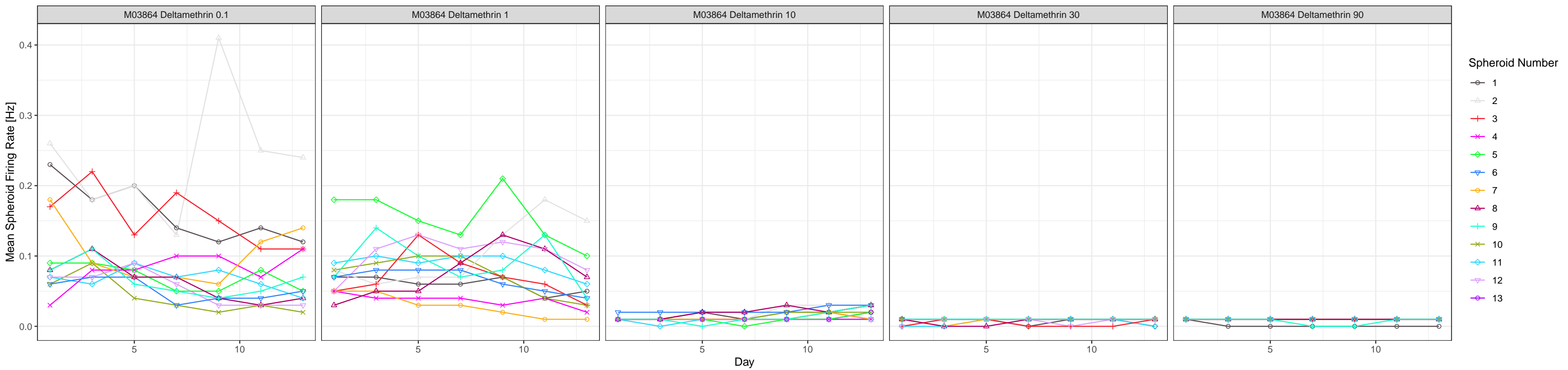

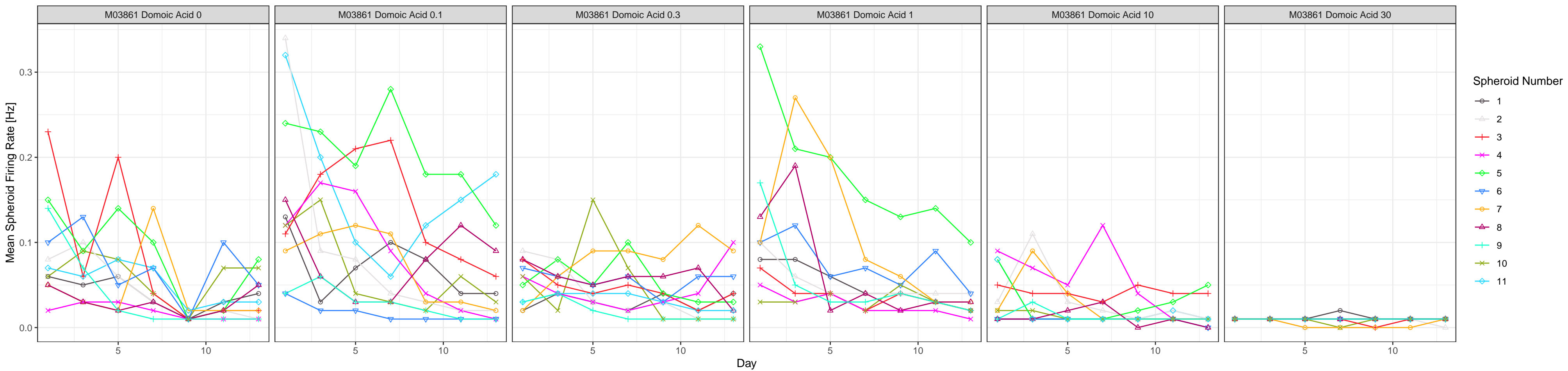

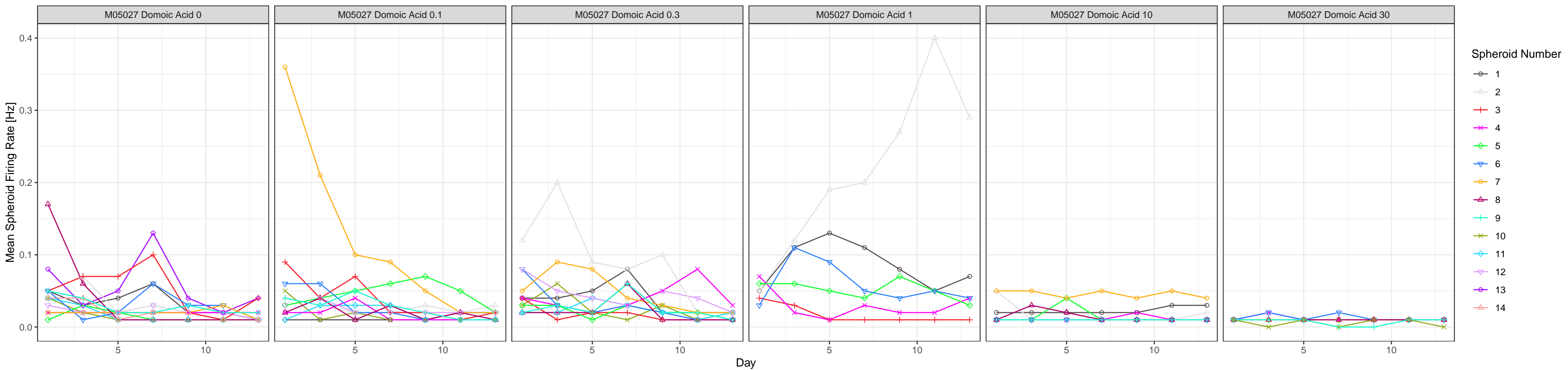

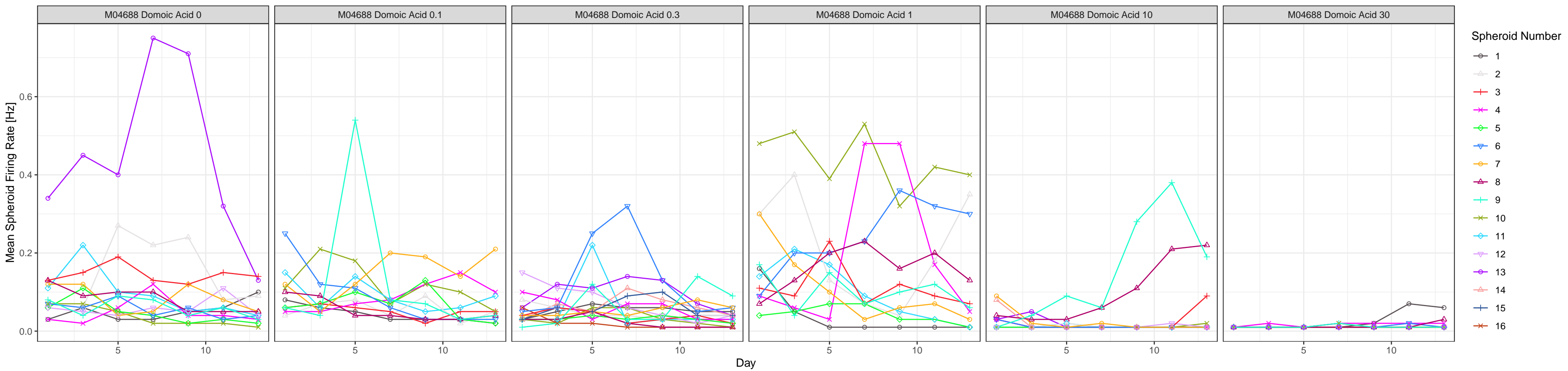

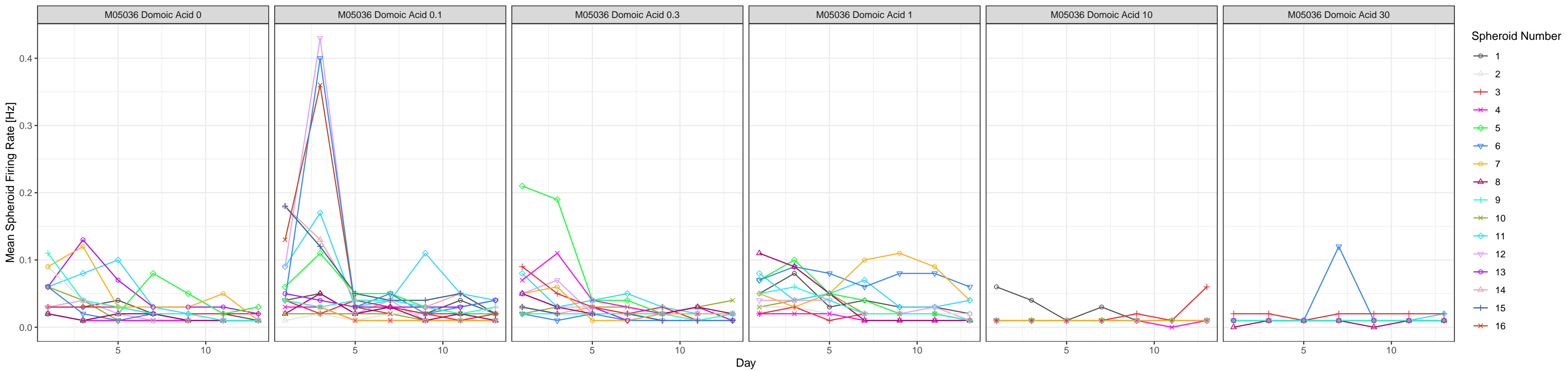

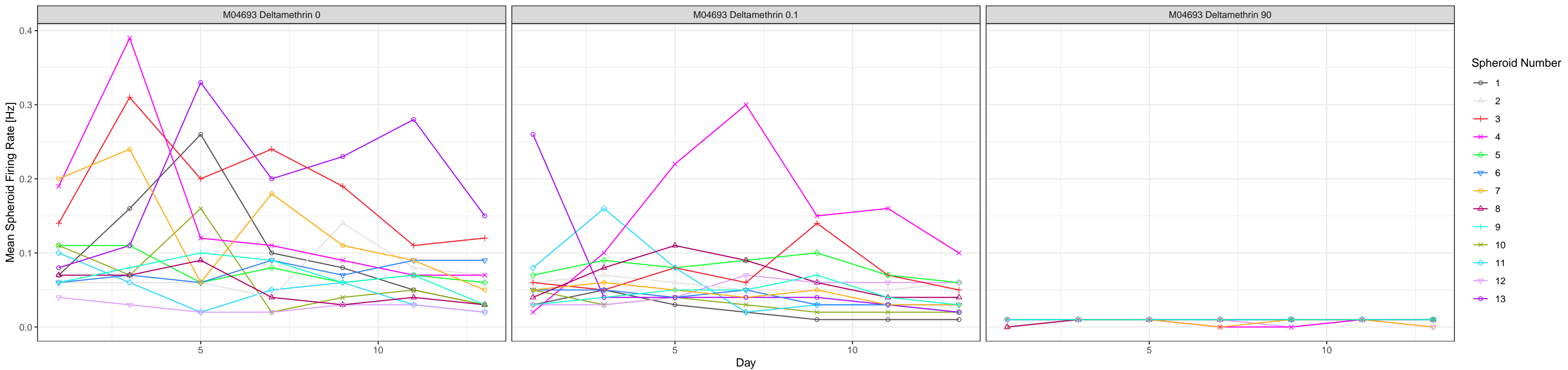

Supplement: Supplementary file 5 — Supplementary file5 (PDF 589 KB) [file 204_2025_4043_MOESM5_ESM.pdf]
