# Supplementary material for: Application of a high-density microelectrode array assay using a 3D human iPSC-derived brain microphysiological system model for in vitro neurotoxicity screening of environmental compounds
Source: Arch Toxicol. 2025 Apr 28;99(7):2917–35. doi: 10.1007/s00204-025-04043-x (PMC12198282; doi:10.1007/s00204-025-04043-x)
Supplement: Supplementary file 3 — Supplementary file3 (PDF 290 KB) [file 204_2025_4043_MOESM3_ESM.pdf]

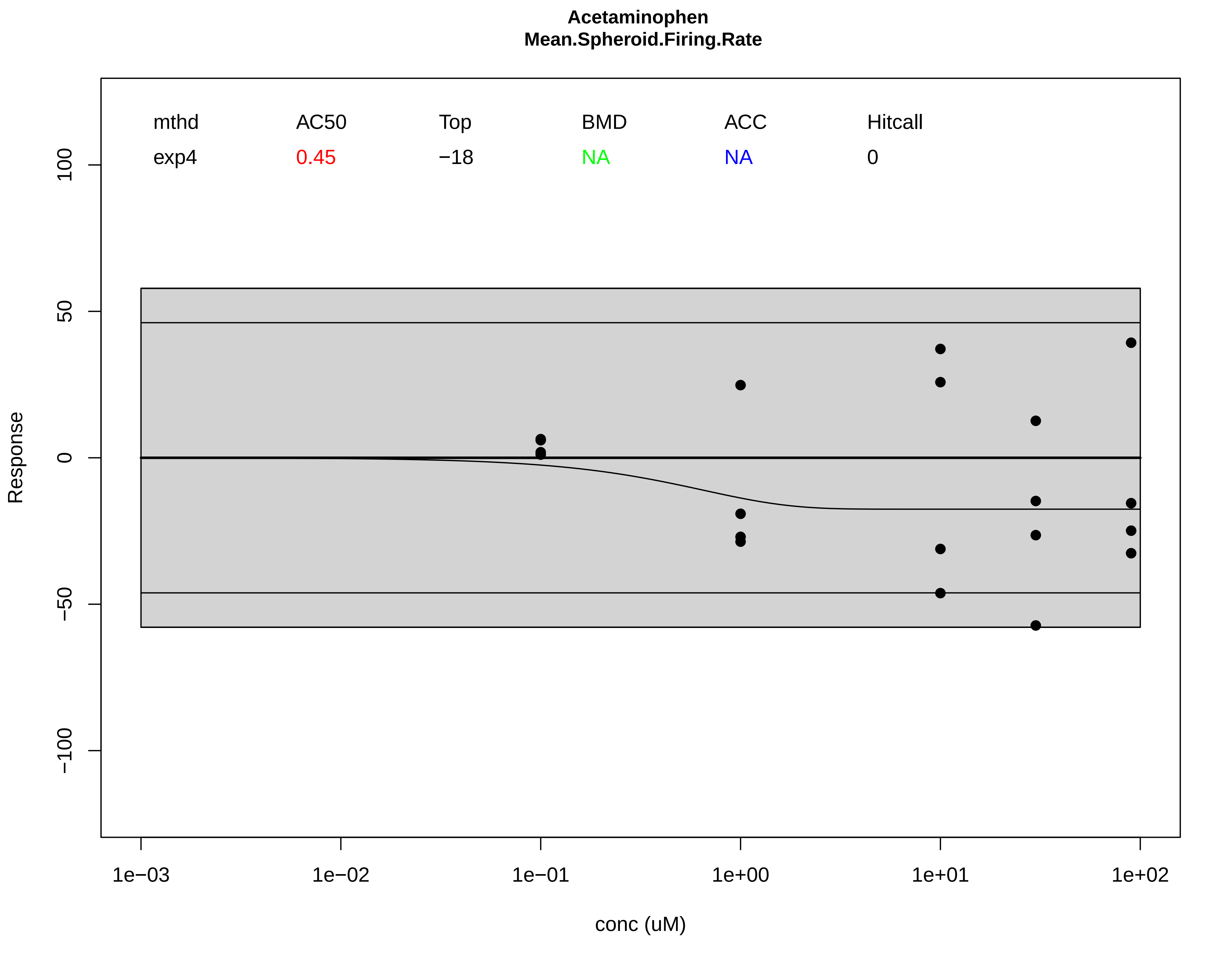

Acetaminophen
Mean.Spheroid.Firing.Rate
mthd
AC50
Top
BMD
ACC
Hitcall
exp4
0.45
−18
NA
NA
0
Response
100
50
0
−50
−100
1e−03
1e−02
1e−01
1e+00
1e+01
1e+02
conc (uM)

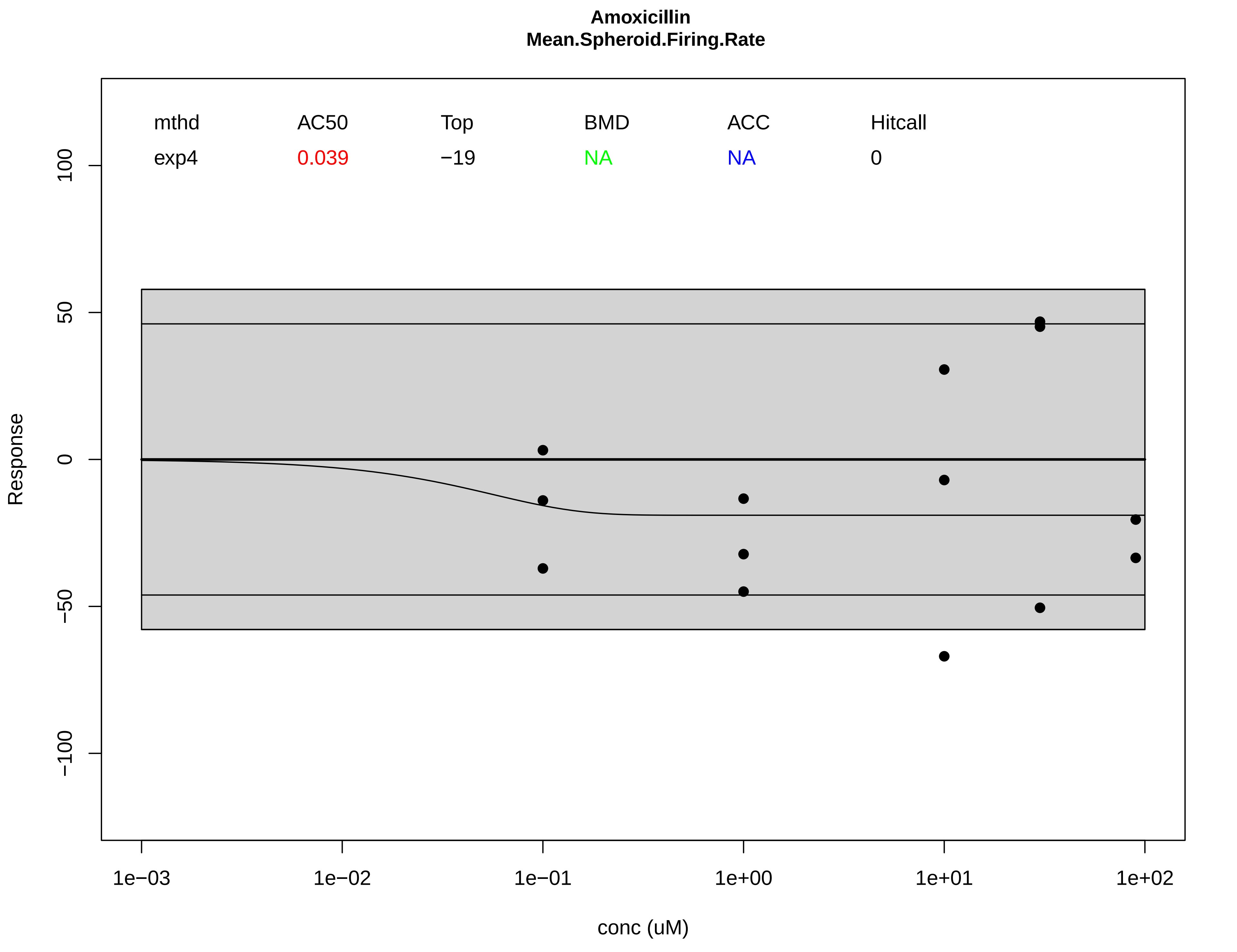
Amoxicillin
Mean.Spheroid.Firing.Rate
mthd
AC50
Top
BMD
ACC
Hitcall
exp4
0.039
−19
NA
NA
0
100
50
0
−50
−100
Response
1e−03
1e−02
1e−01
1e+00
1e+01
1e+02
conc (uM)

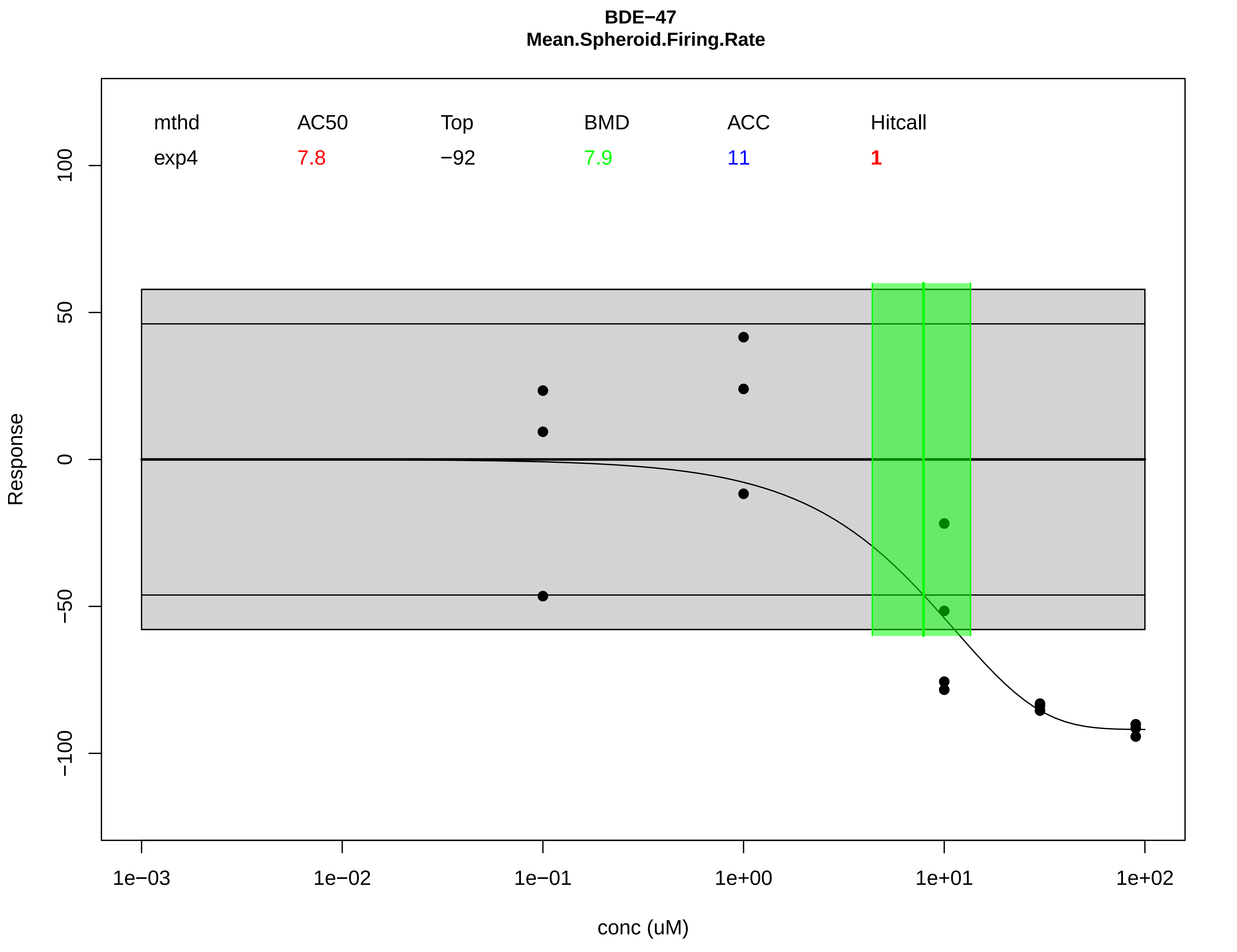

BDE−47
Mean.Spheroid.Firing.Rate
mthd
AC50
Top
BMD
ACC
Hitcall
exp4
7.8
−92
7.9
11
1
Response
100
50
0
−50
−100
1e−03
1e−02
1e−01
1e+00
1e+01
1e+02
conc (uM)

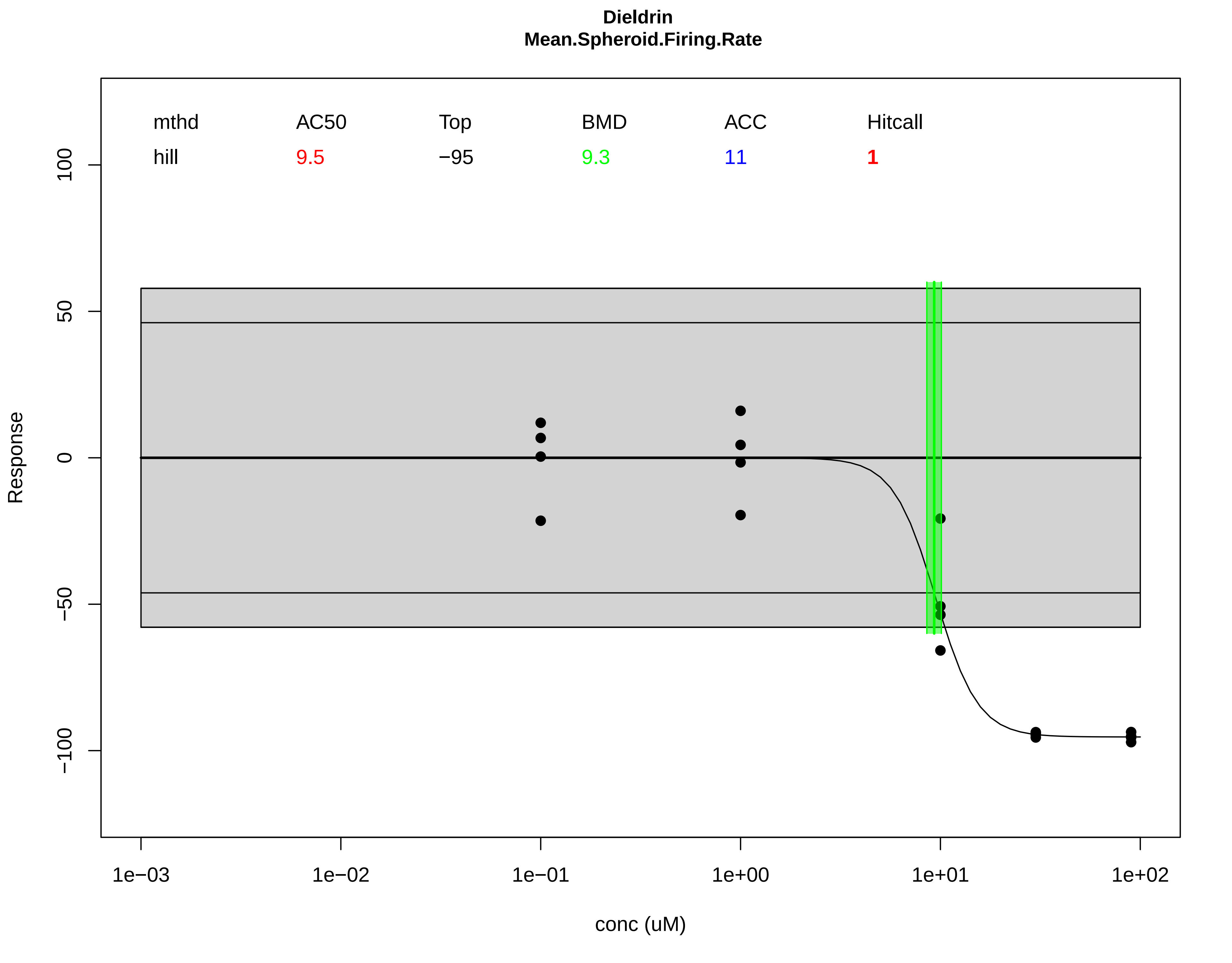
Dieldrin
Mean.Spheroid.Firing.Rate
mthd
AC50
Top
BMD
ACC
Hitcall
hill
9.5
−95
9.3
11
1
Response
100
50
0
−50
−100
1e−03
1e−02
1e−01
1e+00
1e+01
1e+02
conc (uM)

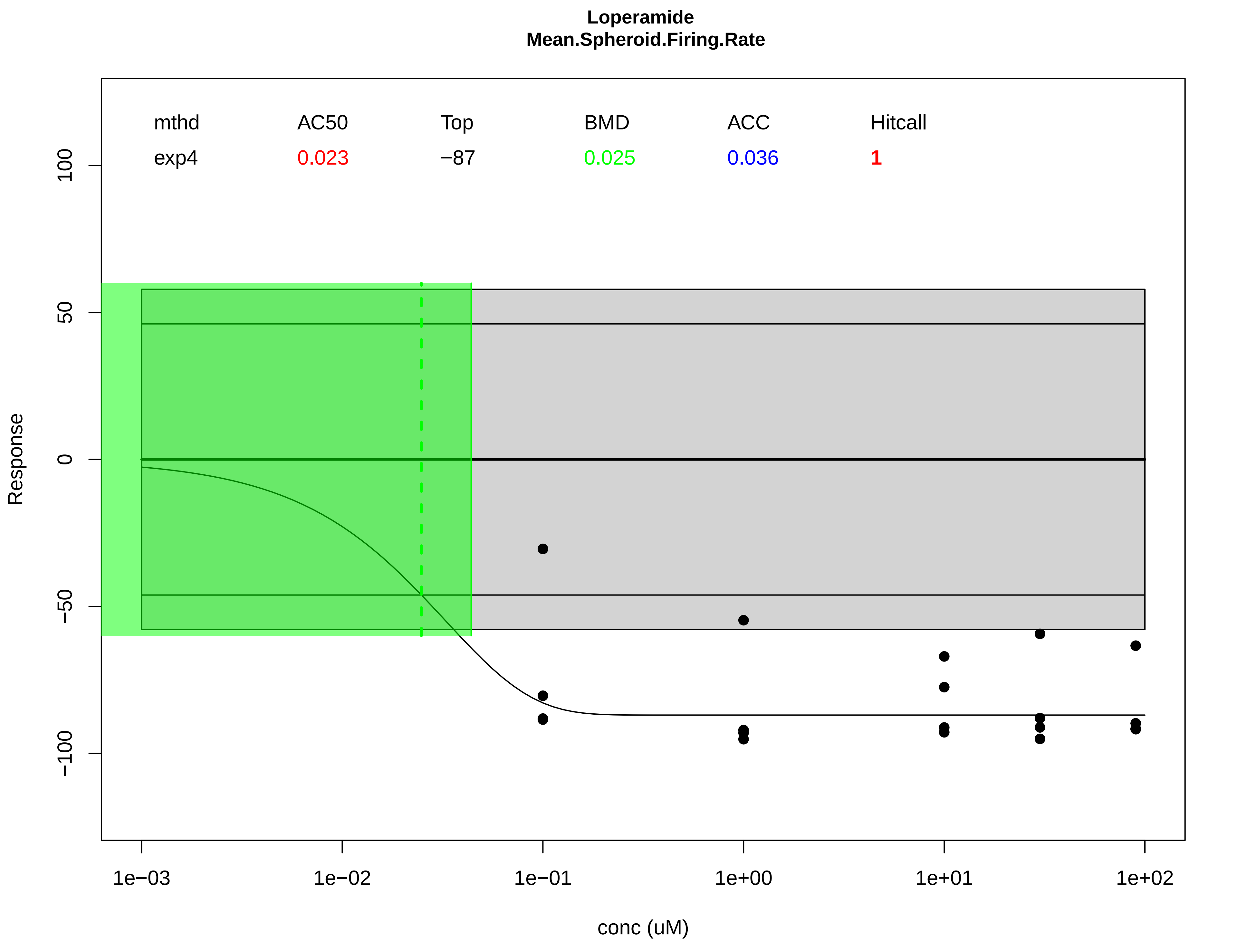
Loperamide
Mean.Spheroid.Firing.Rate
mthd
AC50
Top
BMD
ACC
Hitcall
exp4
0.023
−87
0.025
0.036
1
100
50
0
−50
−100
Response
1e−03
1e−02
1e−01
1e+00
1e+01
1e+02
conc (uM)

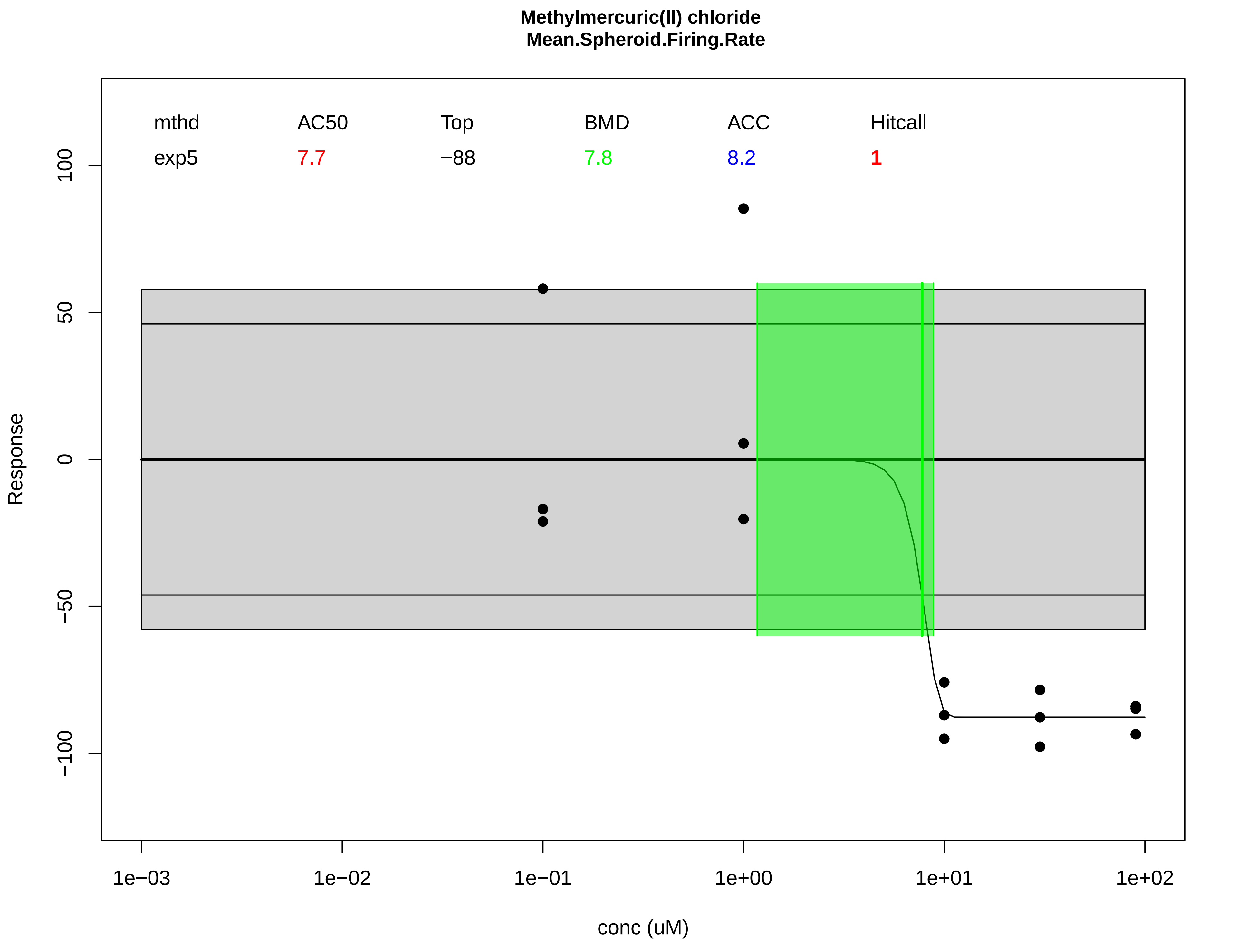
Methylmercuric(II) chloride
Mean.Spheroid.Firing.Rate
mthd
AC50
Top
BMD
ACC
Hitcall
exp5
7.7
−88
7.8
8.2
1
100
50
0
−50
−100
Response
1e−03
1e−02
1e−01
1e+00
1e+01
1e+02
conc (uM)

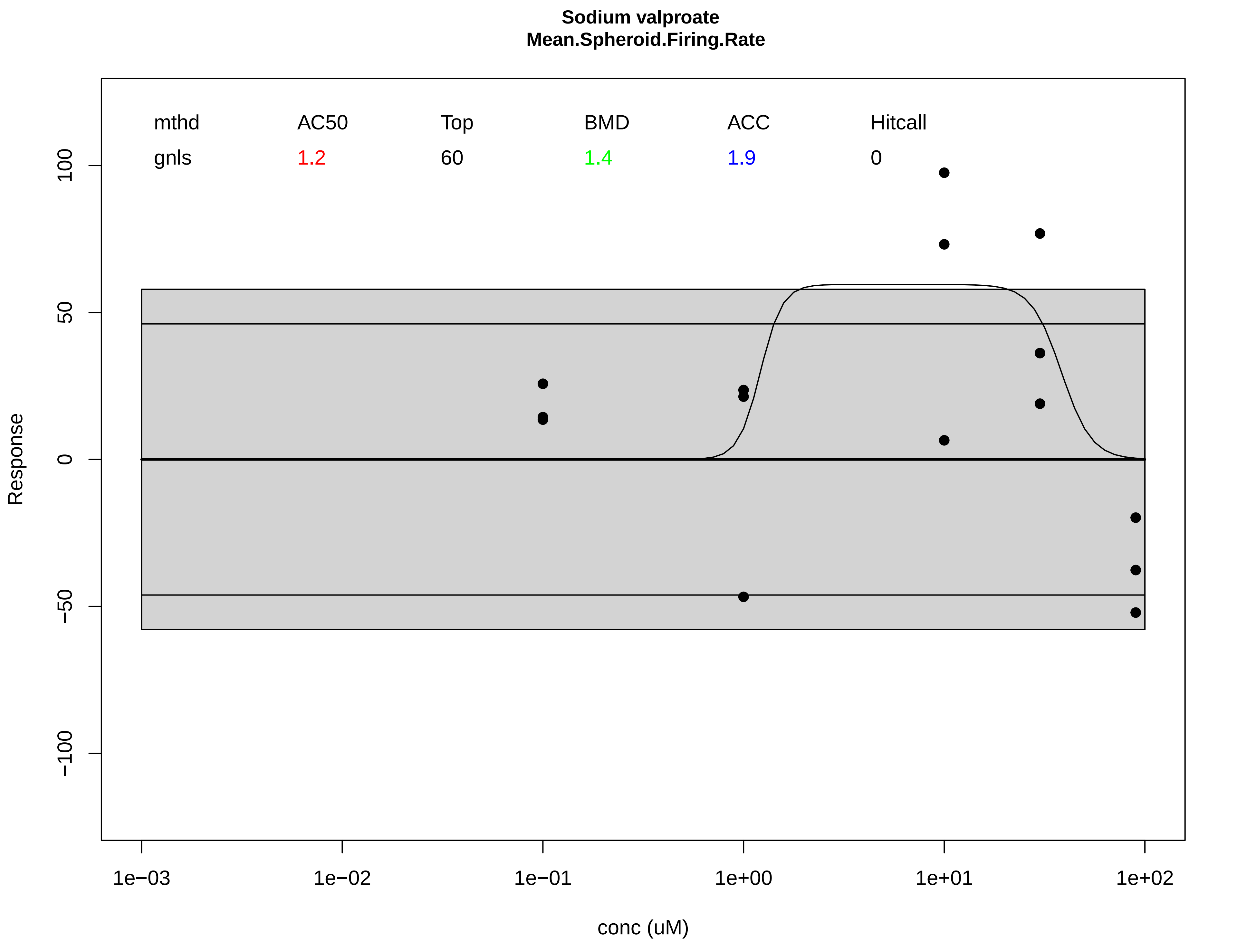
Sodium valproate
Mean.Spheroid.Firing.Rate
mthd
AC50
Top
BMD
ACC
Hitcall
gnls
1.2
60
1.4
1.9
0
100
50
0
−50
−100
Response
1e−03
1e−02
1e−01
1e+00
1e+01
1e+02
conc (uM)

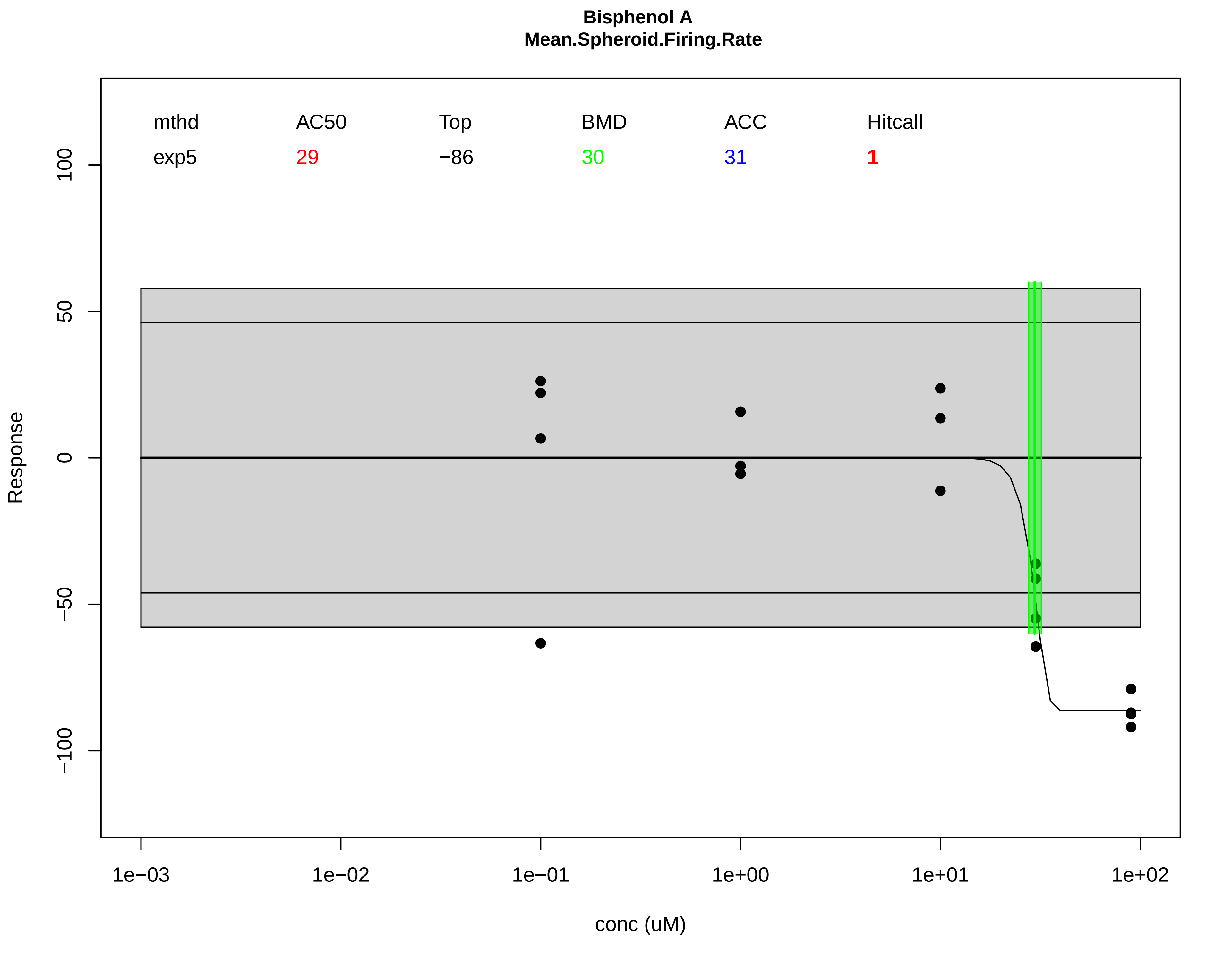

Bisphenol A
Mean.Spheroid.Firing.Rate
mthd
AC50
Top
BMD
ACC
Hitcall
exp5
29
−86
30
31
1
100
50
0
−50
−100
Response
1e−03
1e−02
1e−01
1e+00
1e+01
1e+02
conc (uM)

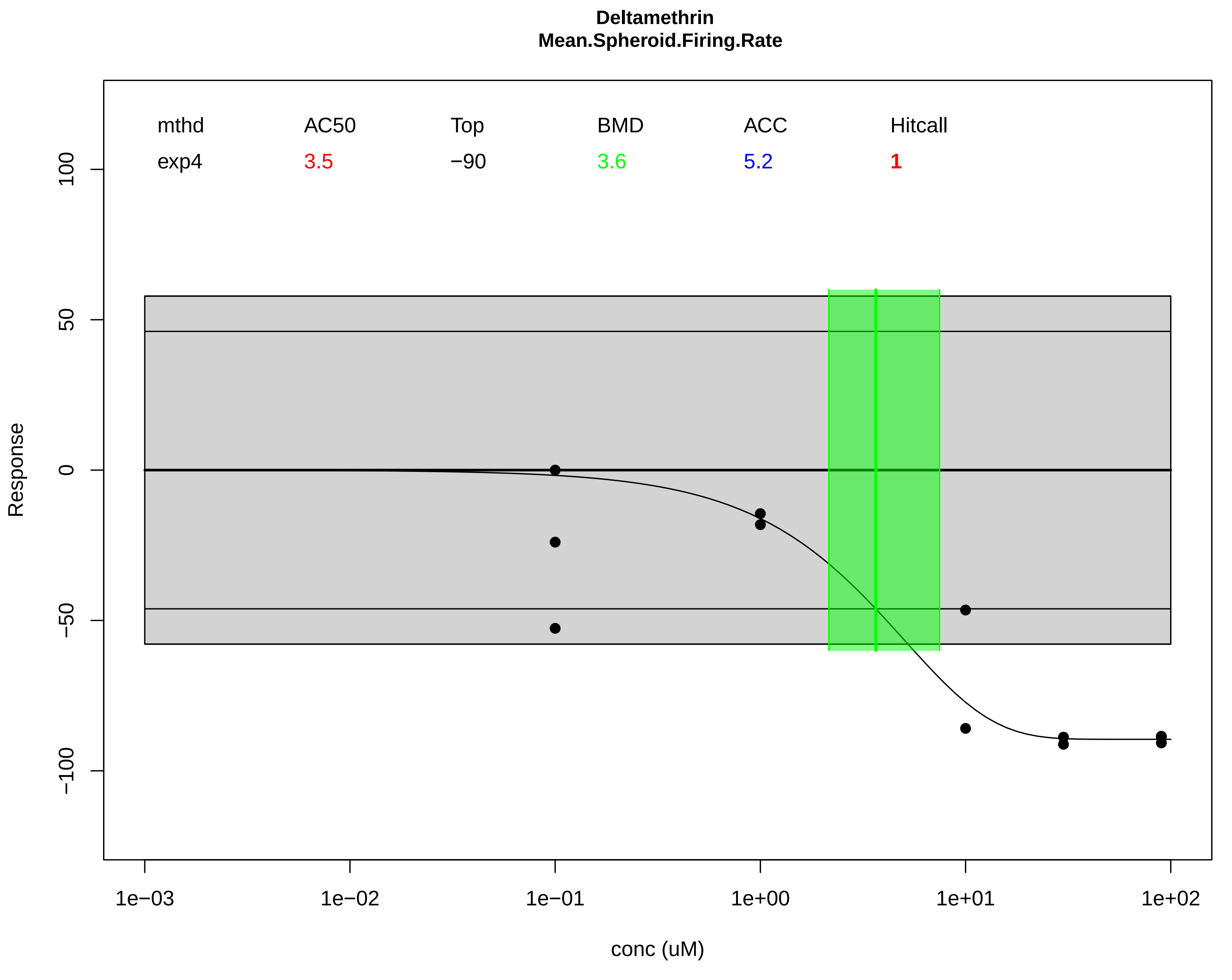

Deltamethrin
Mean.Spheroid.Firing.Rate
mthd
AC50
Top
BMD
ACC
Hitcall
exp4
3.5
−90
3.6
5.2
1
Response
100
50
0
−50
−100
1e−03
1e−02
1e−01
1e+00
1e+01
1e+02
conc (uM)

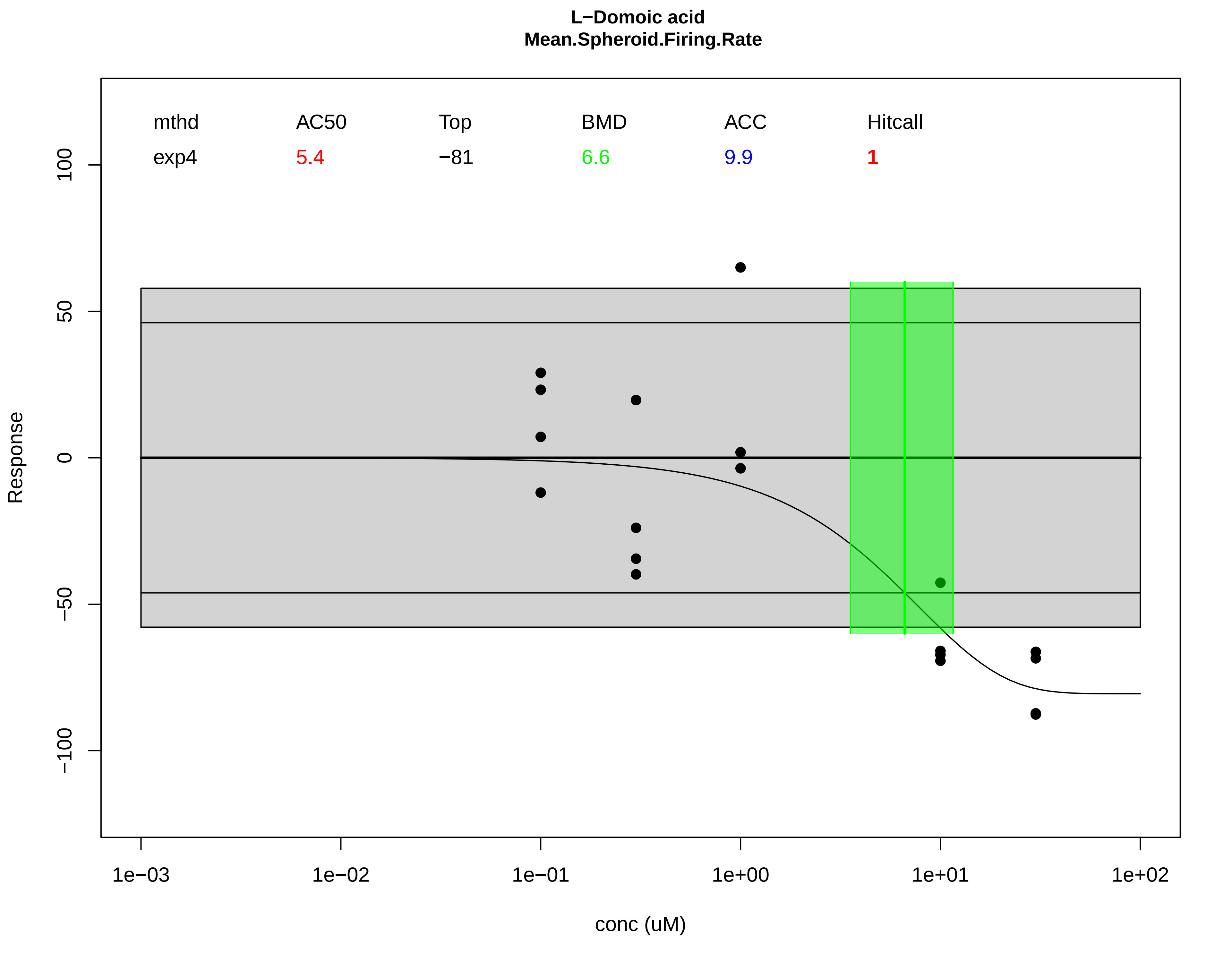
L−Domoic acid
Mean.Spheroid.Firing.Rate
mthd
AC50
Top
BMD
ACC
Hitcall
exp4
5.4
−81
6.6
9.9
1
100
50
0
−50
−100
Response
1e−03
1e−02
1e−01
1e+00
1e+01
1e+02
conc (uM)

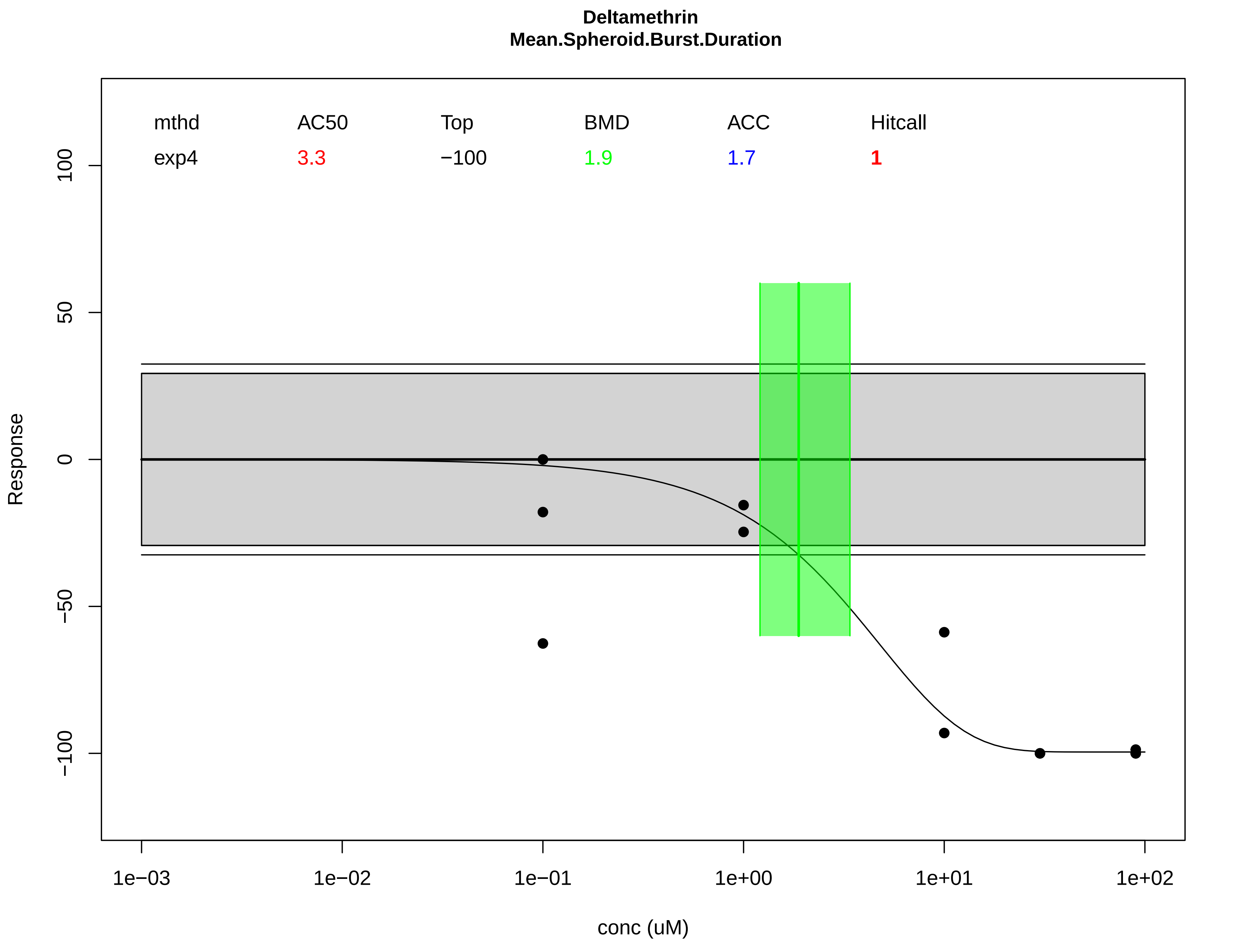

Deltamethrin
Mean.Spheroid.Burst.Duration
mthd
AC50
Top
BMD
ACC
Hitcall
exp4
3.3
−100
1.9
1.7
1
Response
100
50
0
−50
−100
1e−03
1e−02
1e−01
1e+00
1e+01
1e+02
conc (uM)

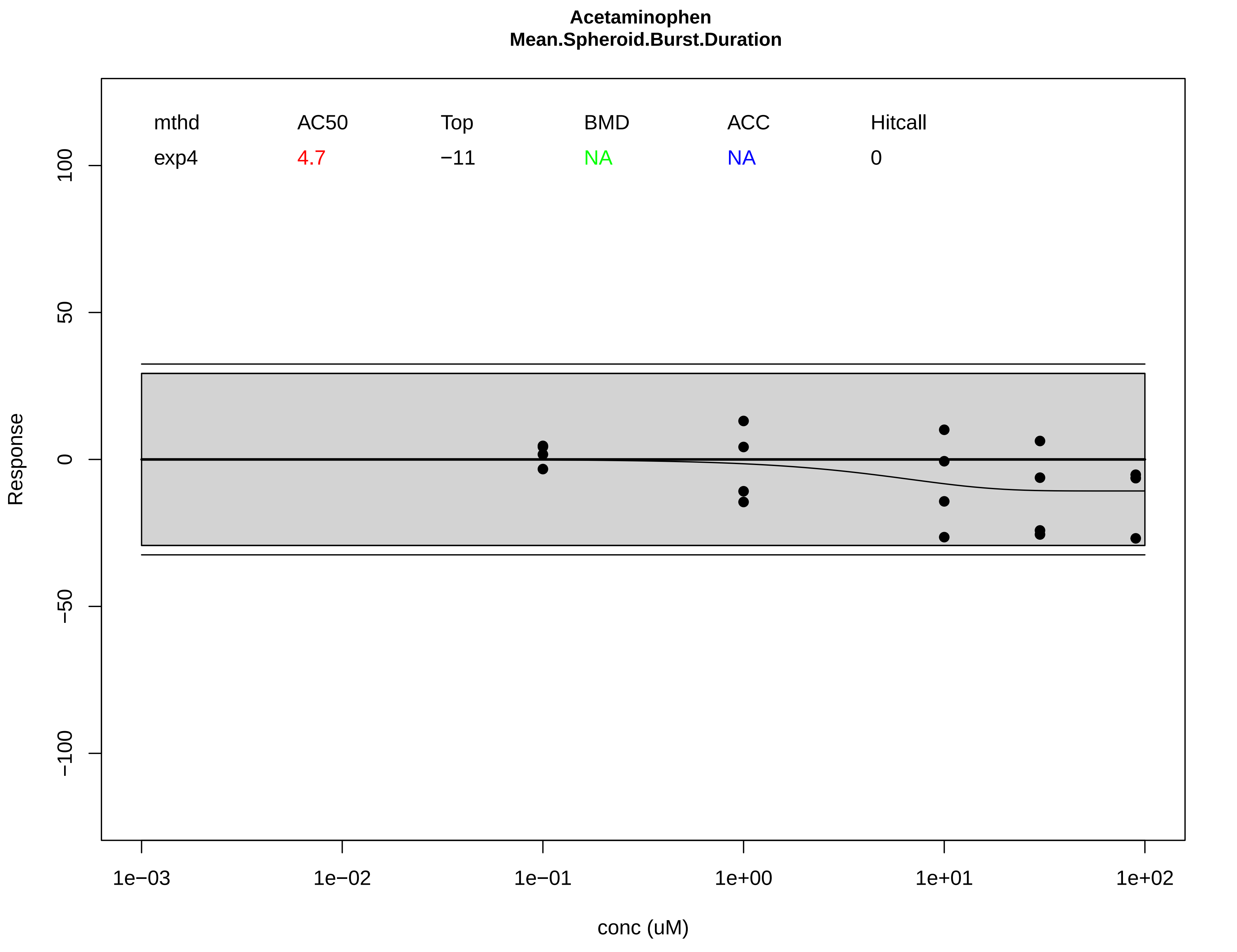
Acetaminophen
Mean.Spheroid.Burst.Duration
mthd
AC50
Top
BMD
ACC
Hitcall
exp4
4.7
−11
NA
NA
0
100
50
0
−50
−100
Response
1e−03
1e−02
1e−01
1e+00
1e+01
1e+02
conc (uM)

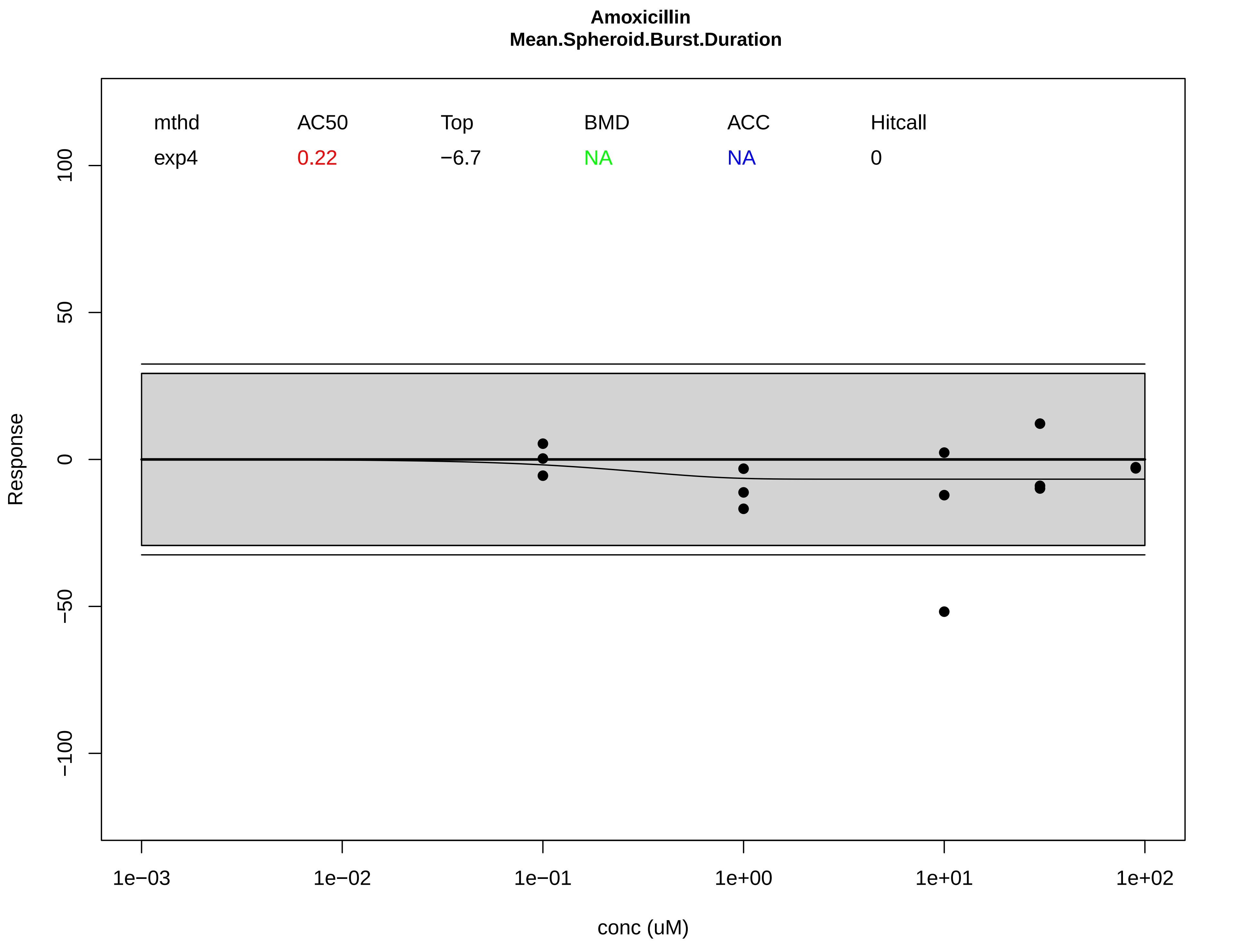
Amoxicillin
Mean.Spheroid.Burst.Duration
mthd
AC50
Top
BMD
ACC
Hitcall
exp4
0.22
−6.7
NA
NA
0
100
50
0
−50
−100
Response
1e−03
1e−02
1e−01
1e+00
1e+01
1e+02
conc (uM)

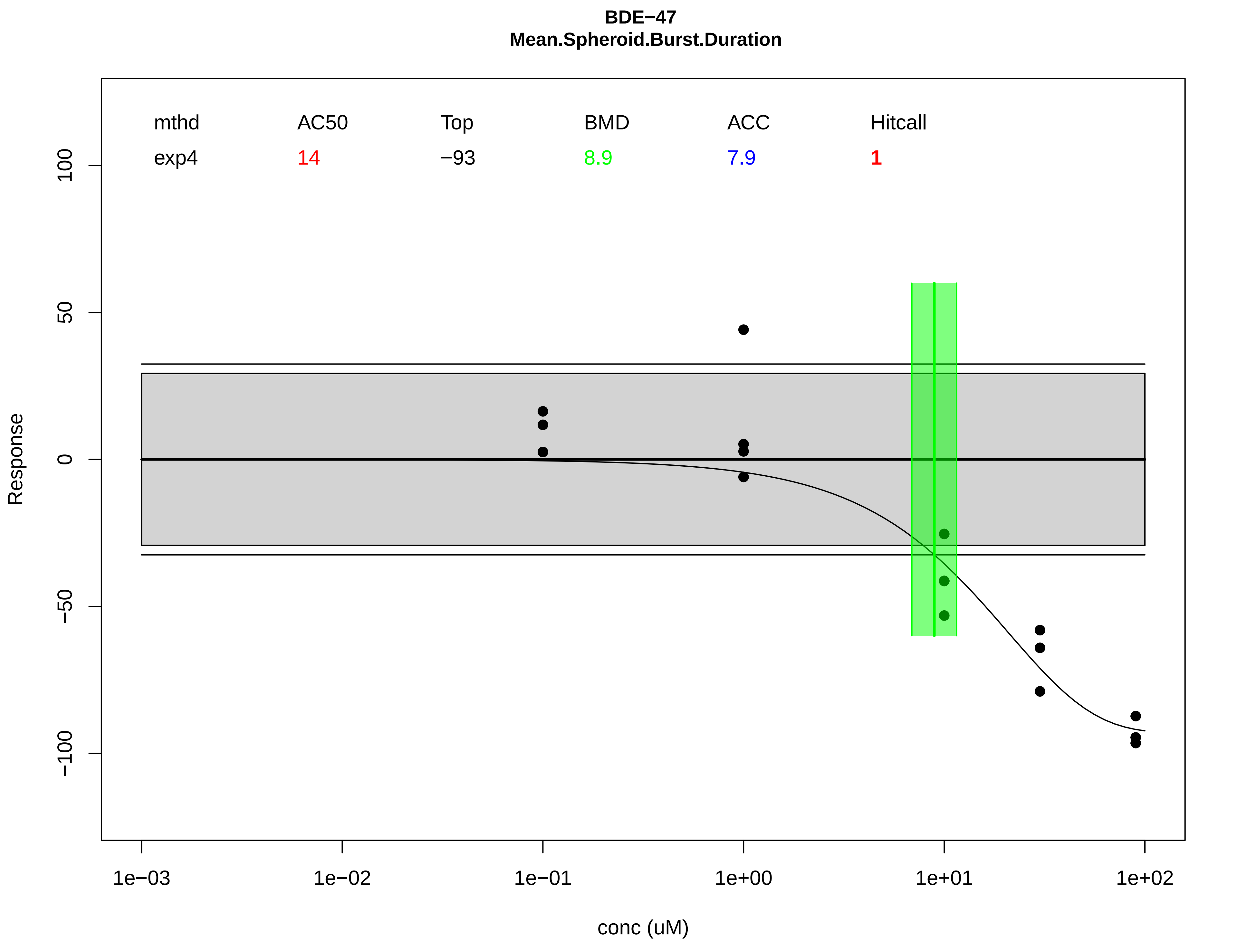
BDE−47
Mean.Spheroid.Burst.Duration
mthd
AC50
Top
BMD
ACC
Hitcall
exp4
14
−93
8.9
7.9
1
100
50
0
−50
−100
Response
1e−03
1e−02
1e−01
1e+00
1e+01
1e+02
conc (uM)

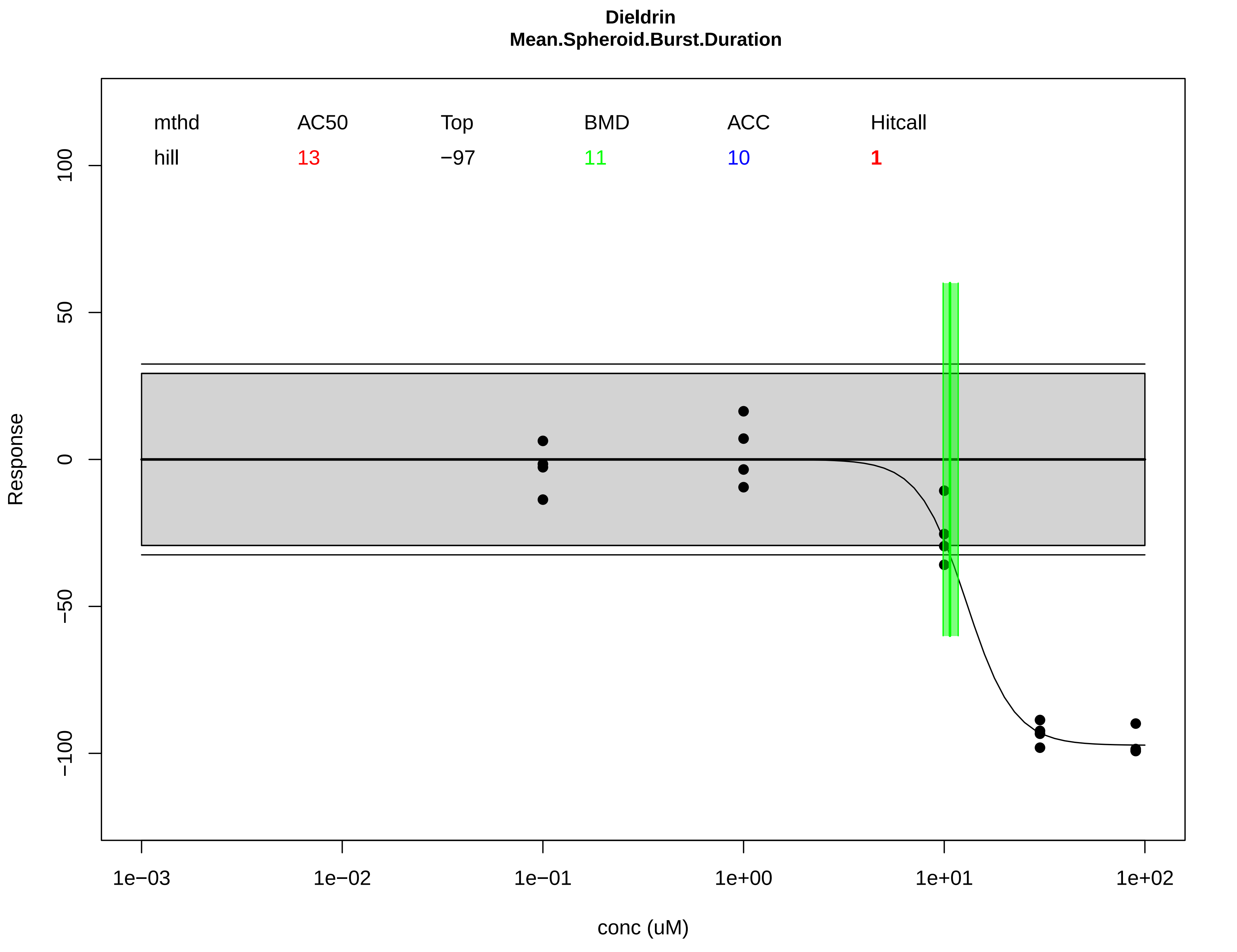
Dieldrin
Mean.Spheroid.Burst.Duration
mthd
AC50
Top
BMD
ACC
Hitcall
hill
13
−97
11
10
1
100
50
0
−50
−100
Response
1e−03
1e−02
1e−01
1e+00
1e+01
1e+02
conc (uM)

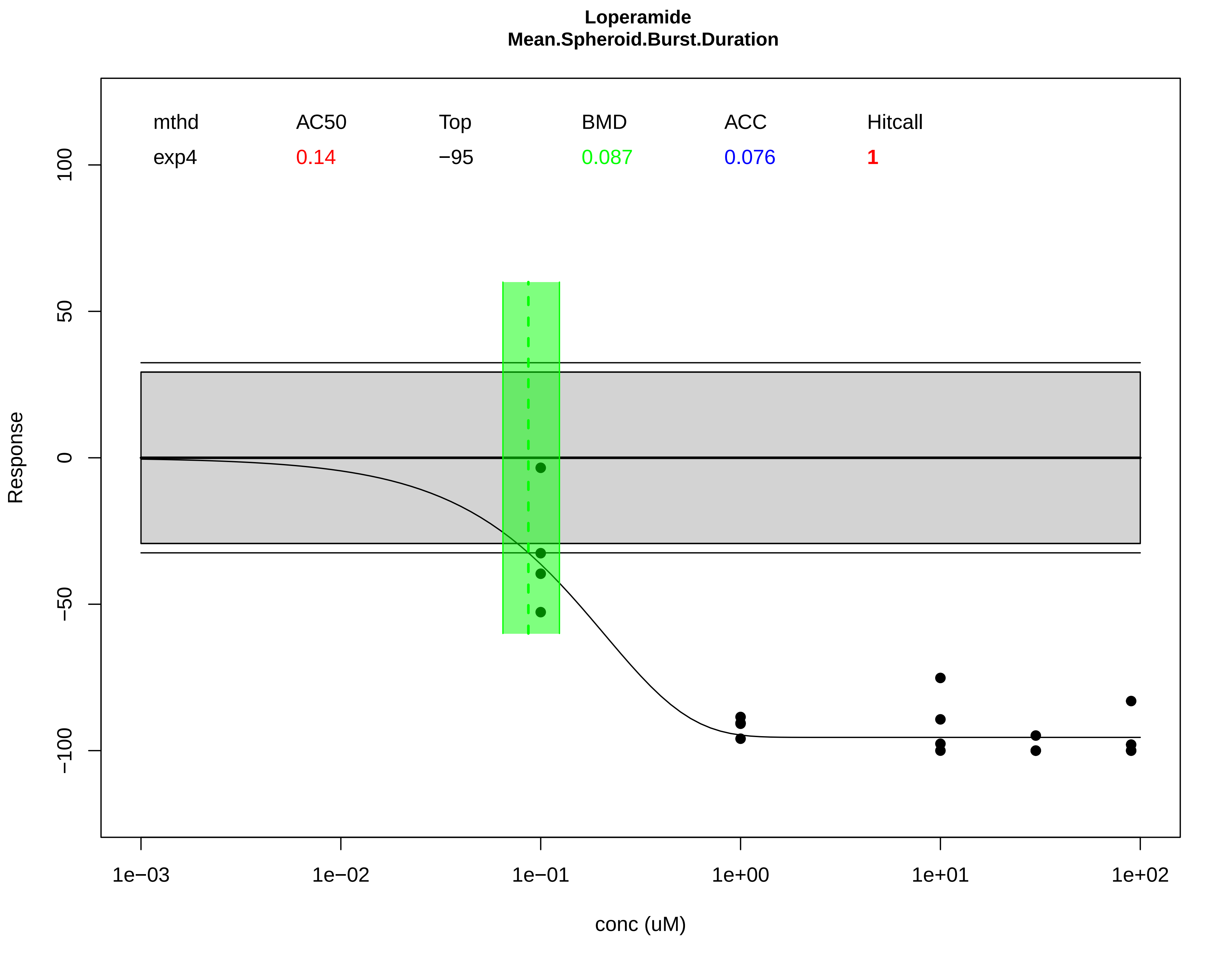
Loperamide
Mean.Spheroid.Burst.Duration
mthd
AC50
Top
BMD
ACC
Hitcall
exp4
0.14
−95
0.087
0.076
1
100
50
0
−50
−100
Response
1e−03
1e−02
1e−01
1e+00
1e+01
1e+02
conc (uM)

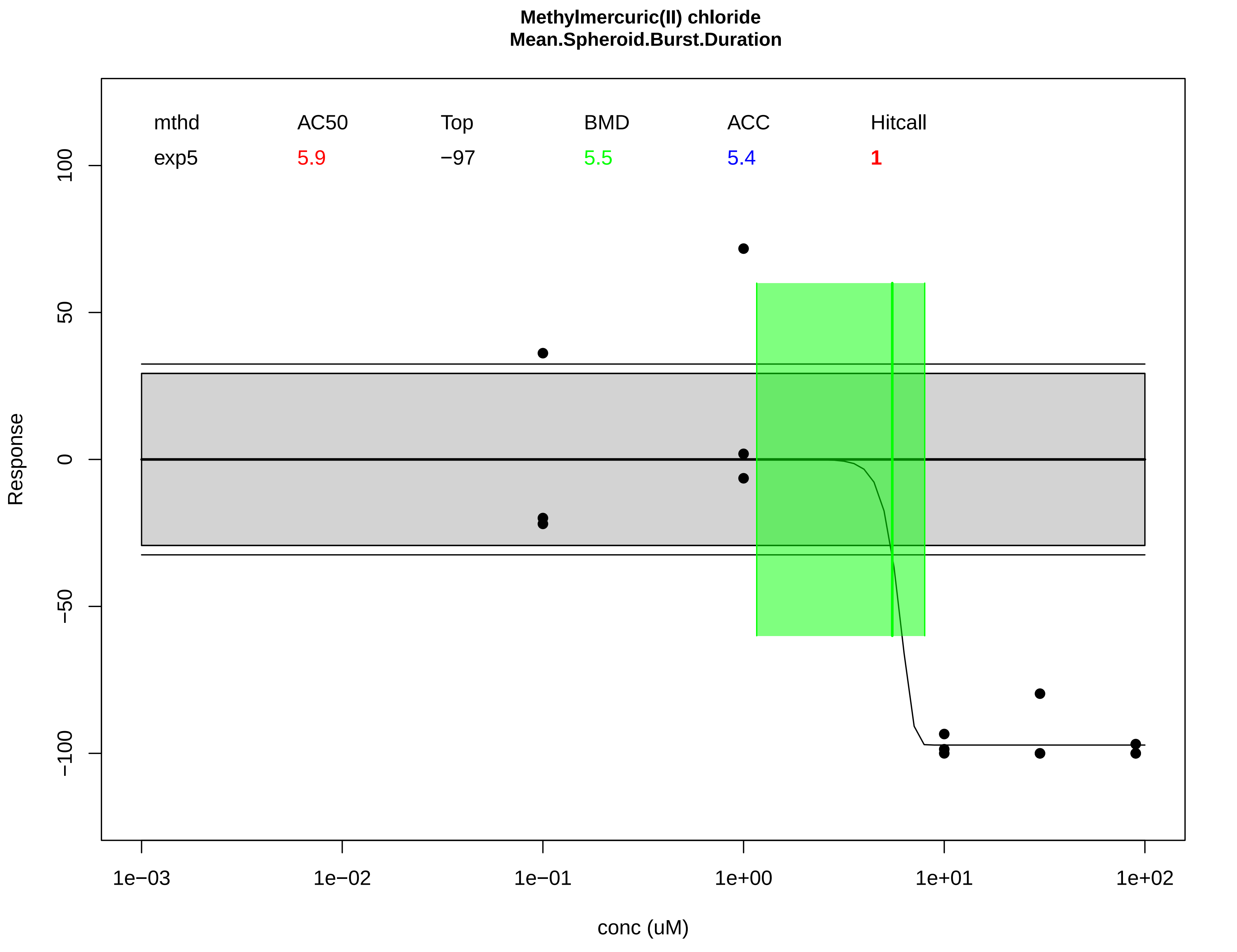

Methylmercuric(II) chloride
Mean.Spheroid.Burst.Duration
mthd
AC50
Top
BMD
ACC
Hitcall
exp5
5.9
−97
5.5
5.4
1
100
50
0
−50
−100
Response
1e−03
1e−02
1e−01
1e+00
1e+01
1e+02
conc (uM)

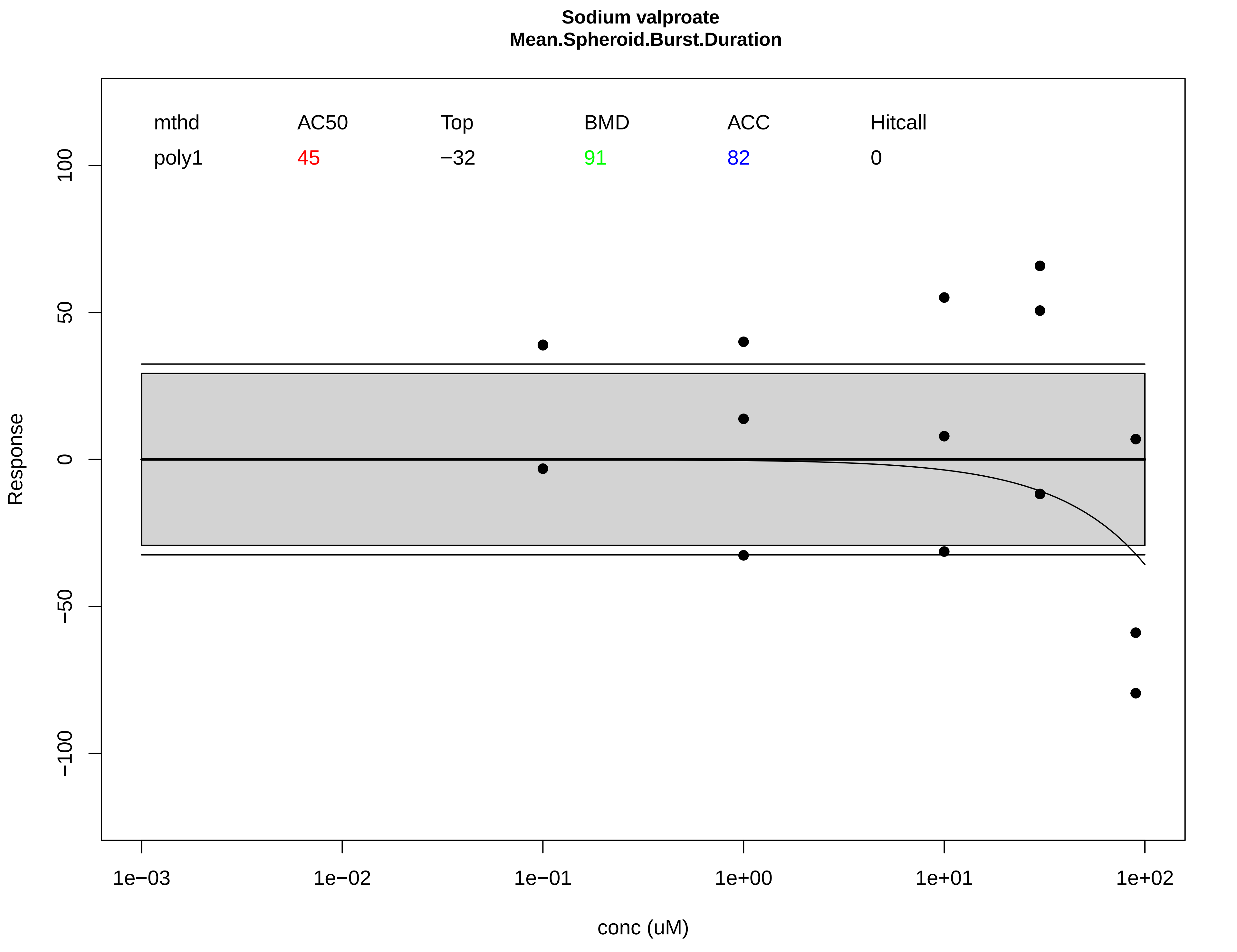

Sodium valproate
Mean.Spheroid.Burst.Duration
mthd
AC50
Top
BMD
ACC
Hitcall
poly1
45
−32
91
82
0
100
50
0
−50
−100
Response
1e−03
1e−02
1e−01
1e+00
1e+01
1e+02
conc (uM)

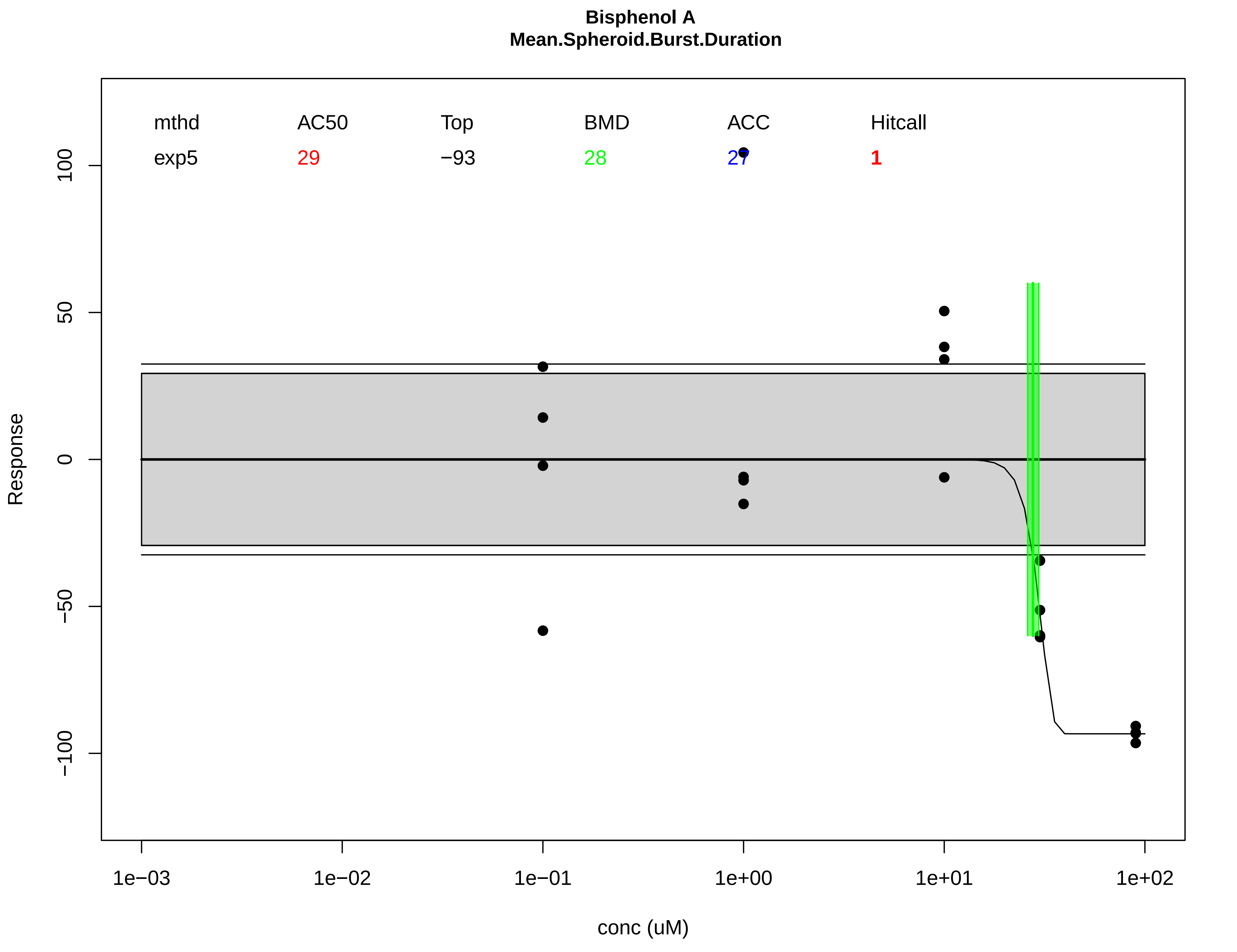

Bisphenol A
Mean.Spheroid.Burst.Duration
mthd
AC50
Top
BMD
ACC
Hitcall
exp5
29
−93
28
27
1
100
50
0
−50
−100
Response
1e−03
1e−02
1e−01
1e+00
1e+01
1e+02
conc (uM)

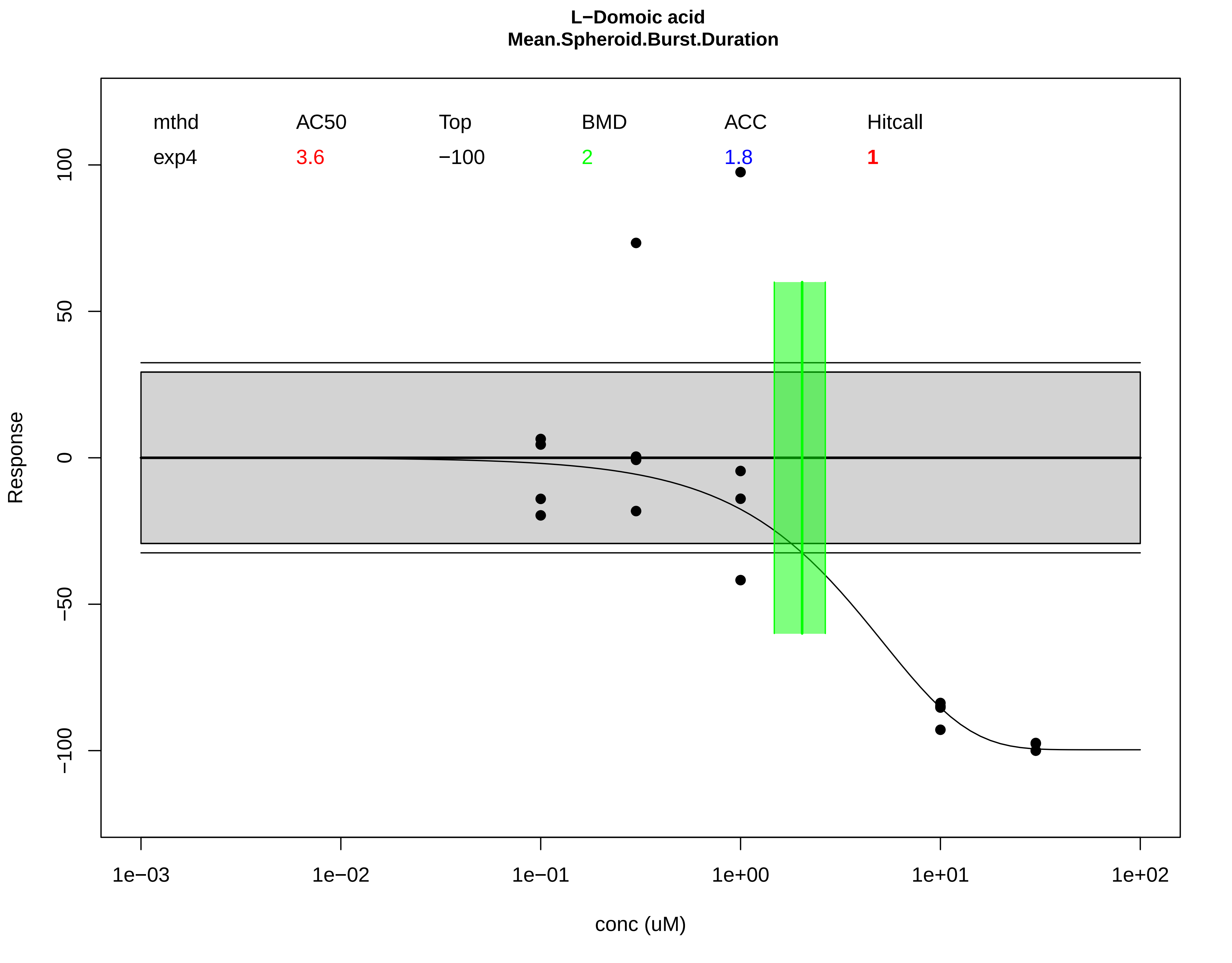
L−Domoic acid
Mean.Spheroid.Burst.Duration
mthd
AC50
Top
BMD
ACC
Hitcall
exp4
3.6
−100
2
1.8
1
100
50
0
−50
−100
Response
1e−03
1e−02
1e−01
1e+00
1e+01
1e+02
conc (uM)

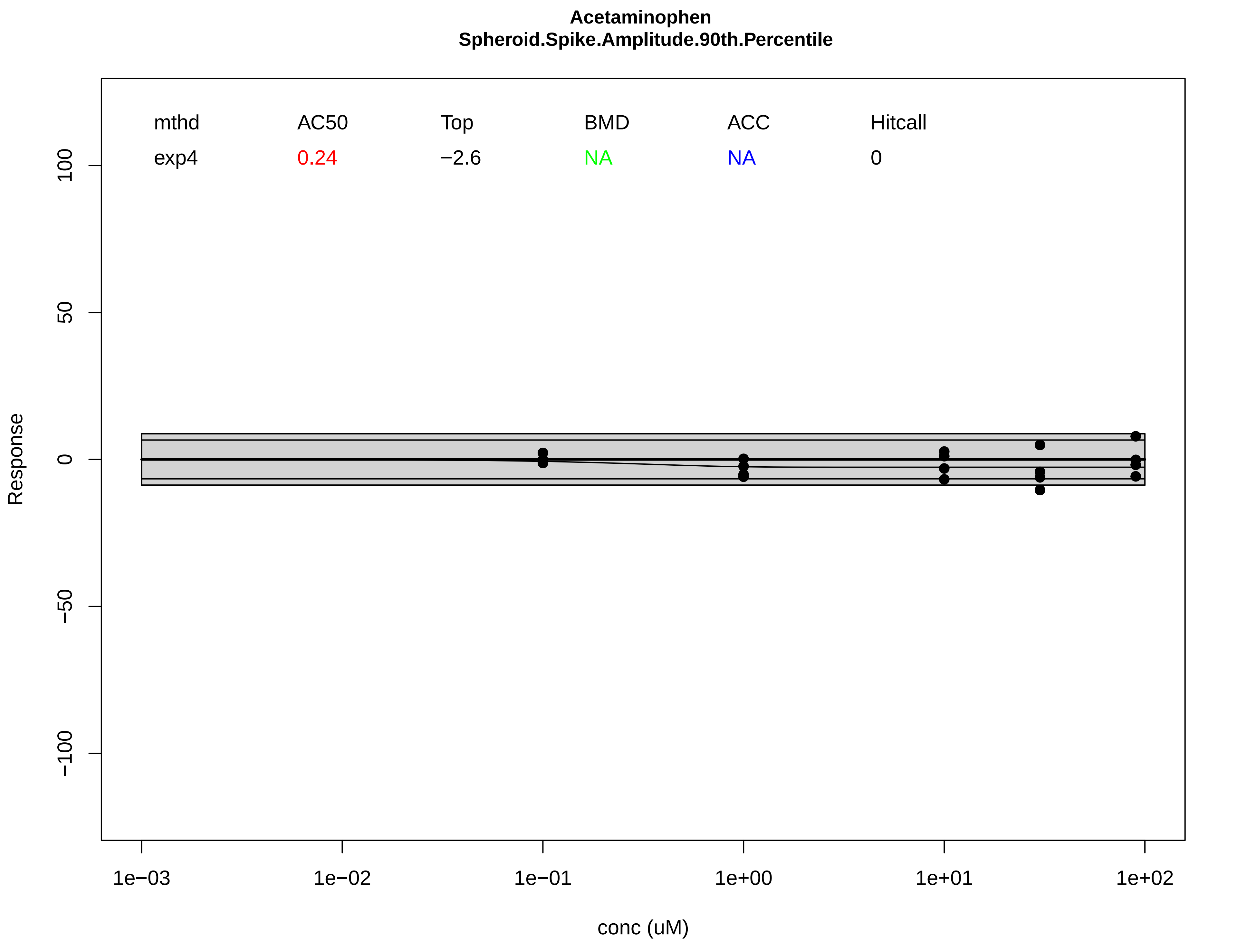

Acetaminophen
Spheroid.Spike.Amplitude.90th.Percentile
mthd
AC50
Top
BMD
ACC
Hitcall
exp4
0.24
−2.6
NA
NA
0
Response
100
50
0
−50
−100
1e−03
1e−02
1e−01
1e+00
1e+01
1e+02
conc (uM)

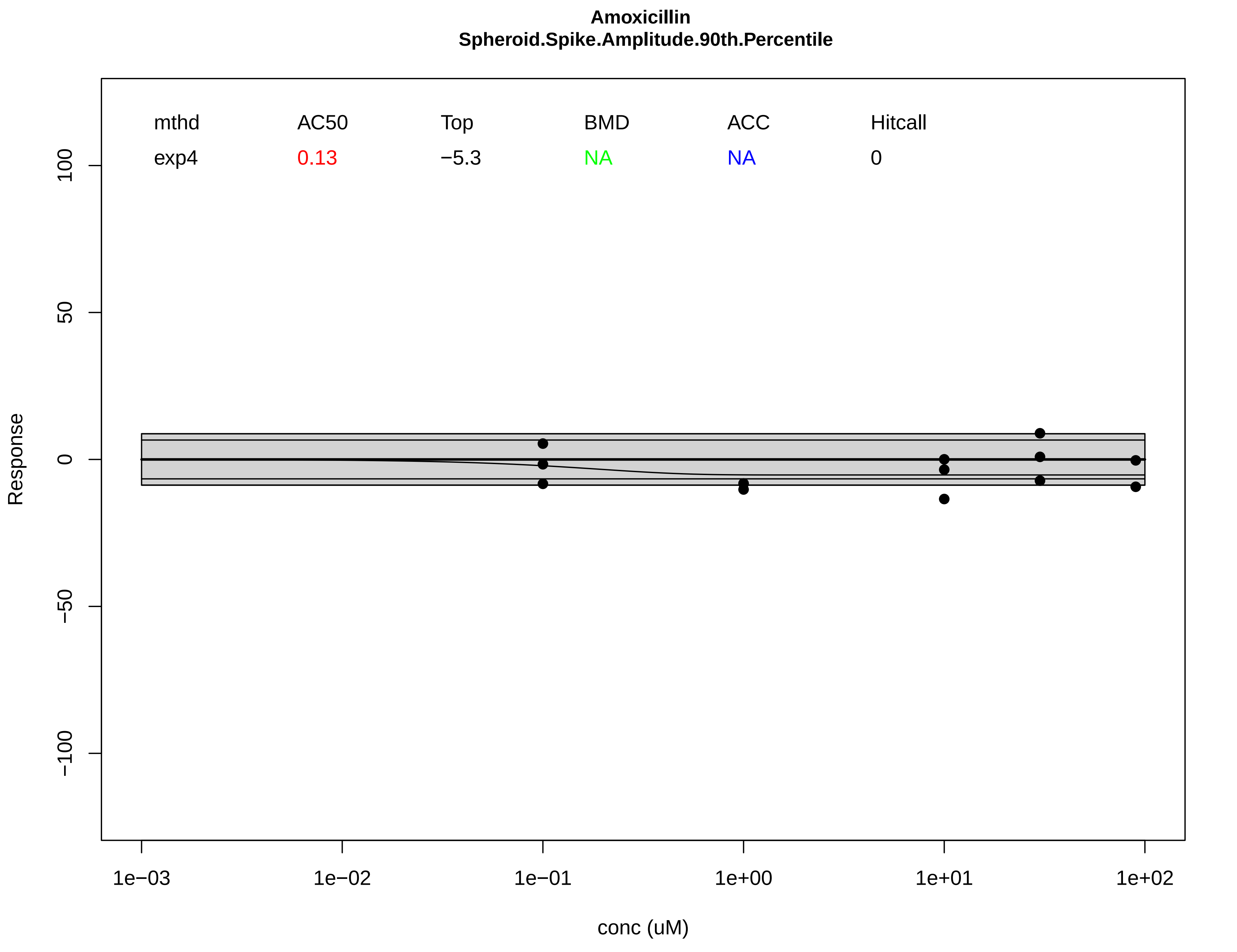
Amoxicillin
Spheroid.Spike.Amplitude.90th.Percentile
mthd
AC50
Top
BMD
ACC
Hitcall
exp4
0.13
−5.3
NA
NA
0
Response
100
50
0
−50
−100
1e−03
1e−02
1e−01
1e+00
1e+01
1e+02
conc (uM)

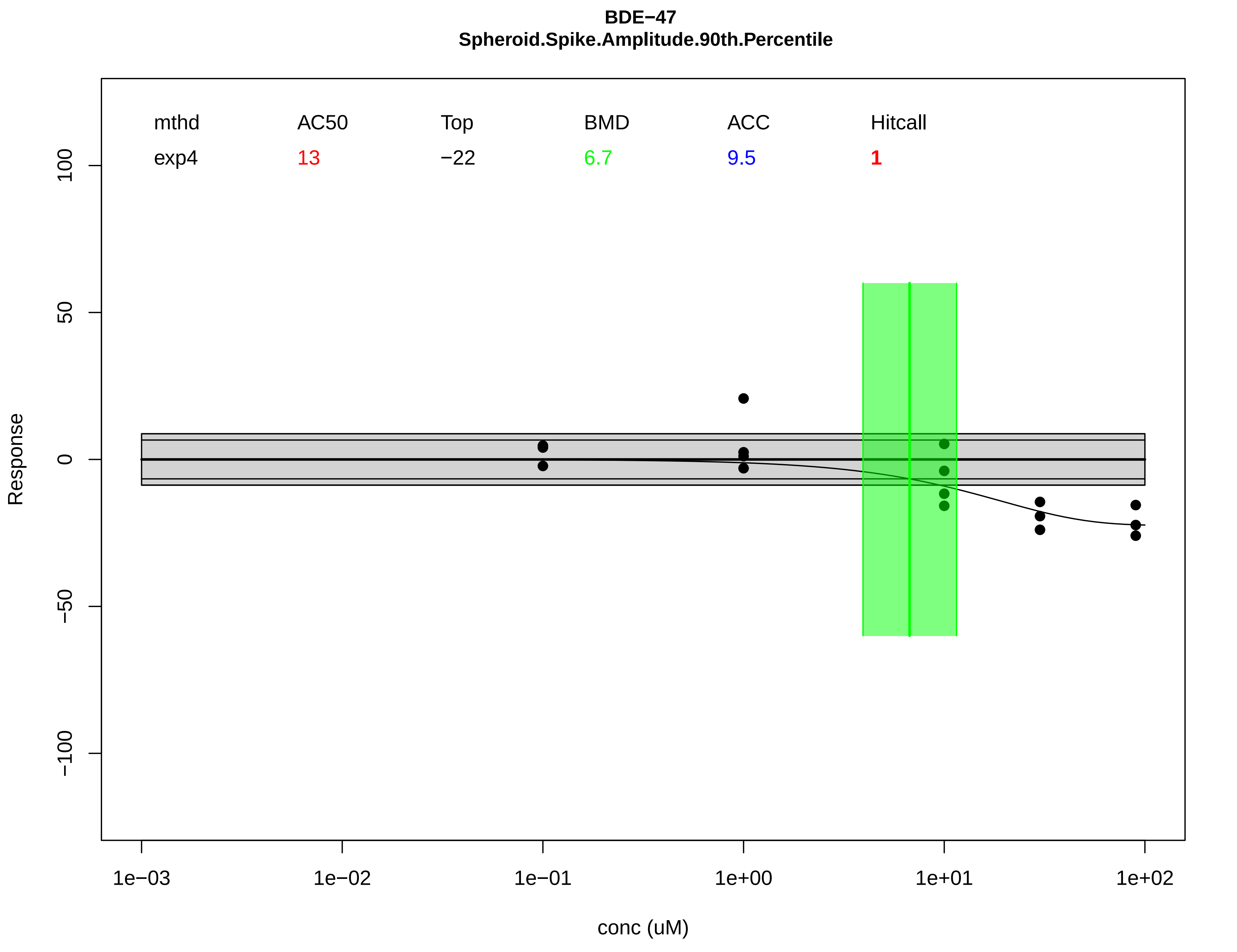

BDE−47
Spheroid.Spike.Amplitude.90th.Percentile
mthd
AC50
Top
BMD
ACC
Hitcall
exp4
13
−22
6.7
9.5
1
Response
100
50
0
−50
−100
1e−03
1e−02
1e−01
1e+00
1e+01
1e+02
conc (uM)

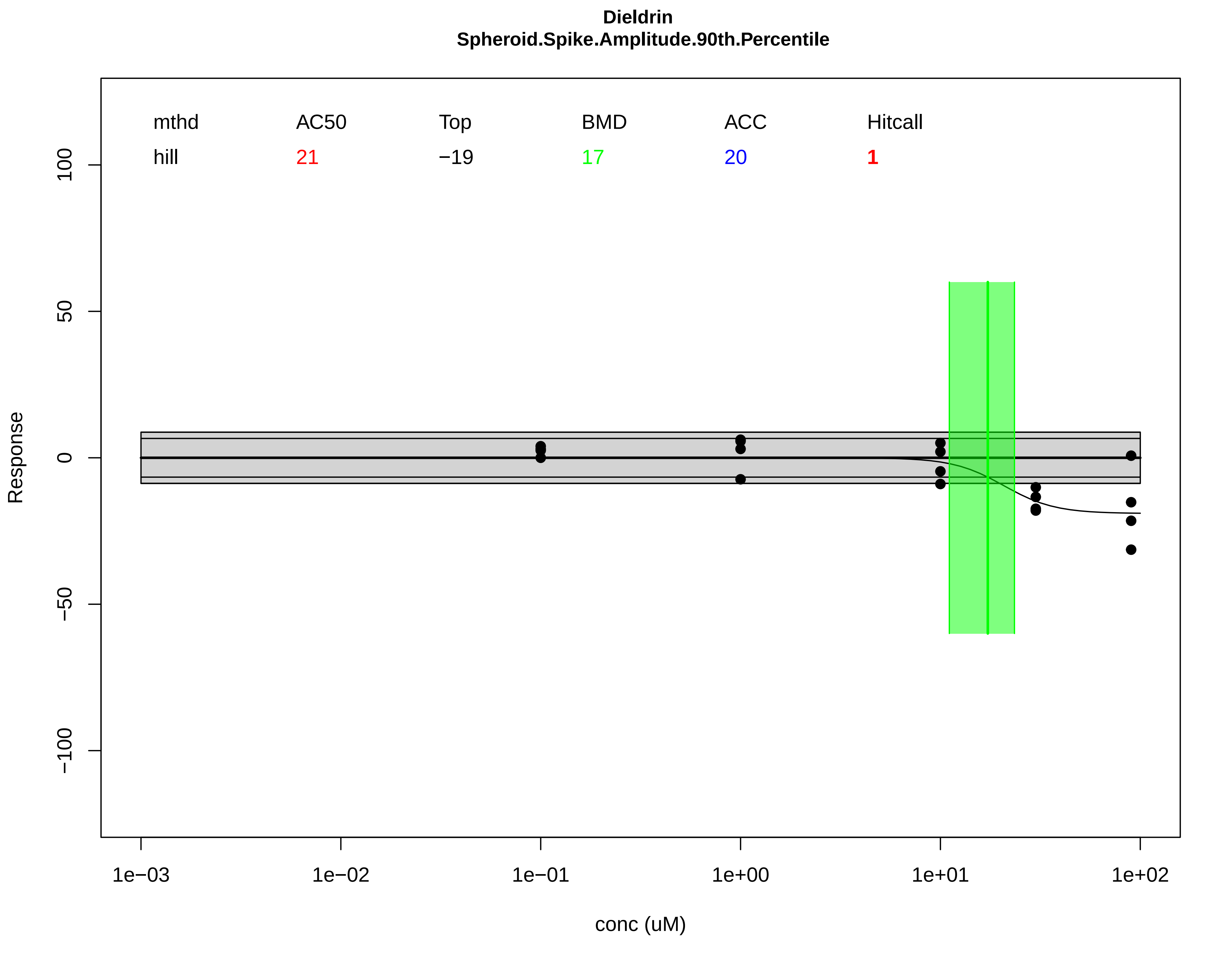
Dieldrin
Spheroid.Spike.Amplitude.90th.Percentile
mthd
AC50
Top
BMD
ACC
Hitcall
hill
21
−19
17
20
1
100
50
0
−50
−100
Response
1e−03
1e−02
1e−01
1e+00
1e+01
1e+02
conc (uM)

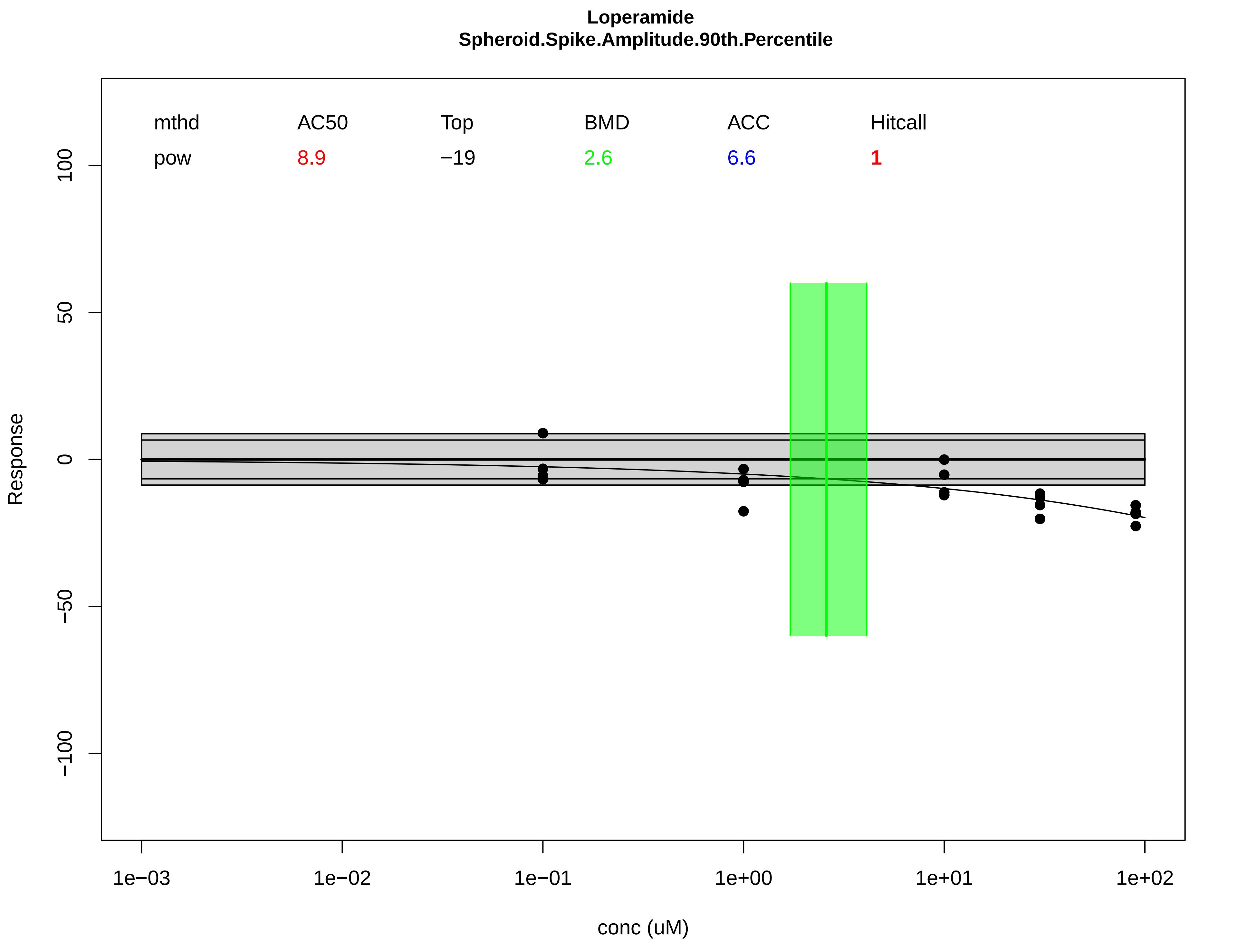
Loperamide
Spheroid.Spike.Amplitude.90th.Percentile
mthd
AC50
Top
BMD
ACC
Hitcall
pow
8.9
−19
2.6
6.6
1
100
50
0
−50
−100
Response
1e−03
1e−02
1e−01
1e+00
1e+01
1e+02
conc (uM)

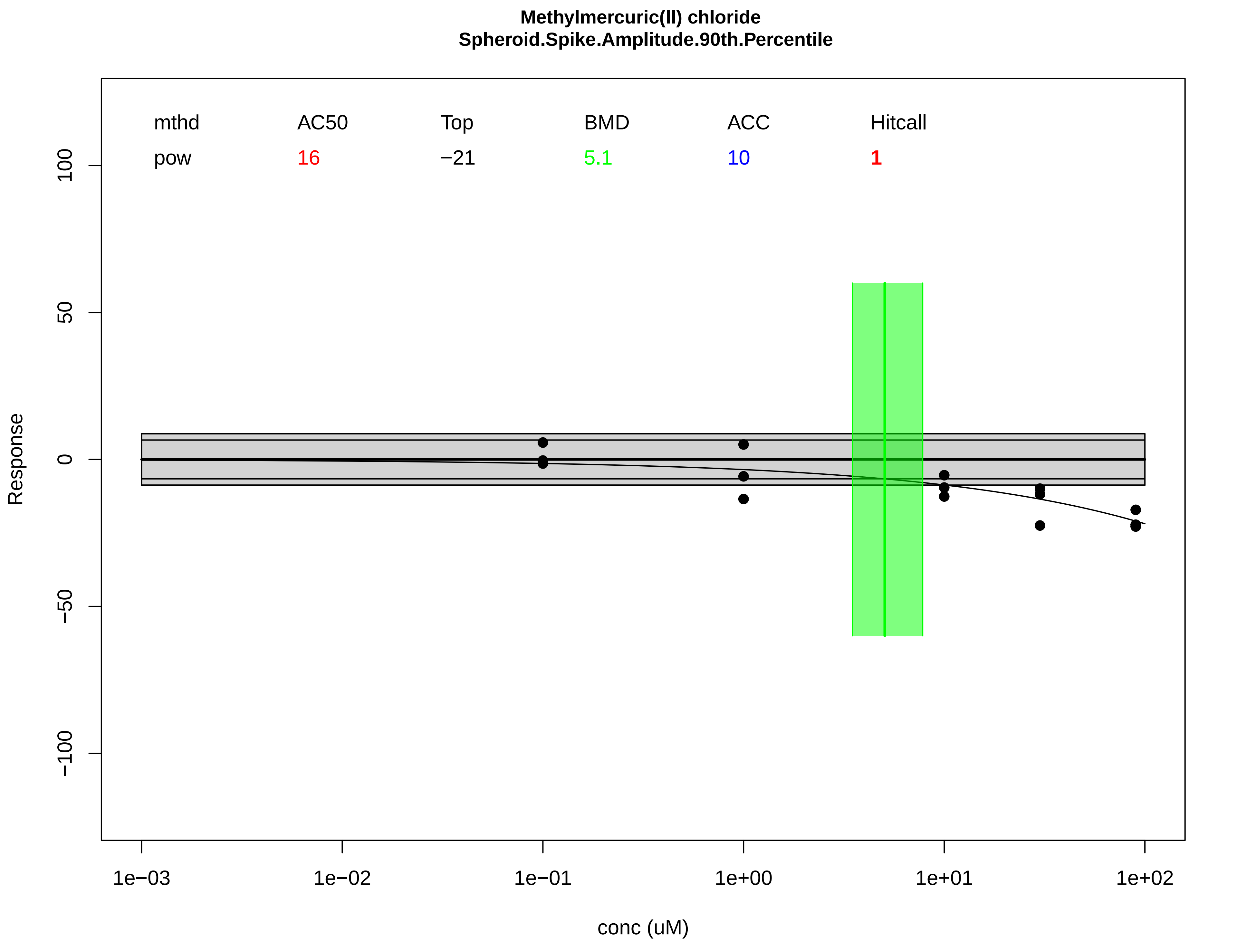
Methylmercuric(II) chloride
Spheroid.Spike.Amplitude.90th.Percentile
mthd
AC50
Top
BMD
ACC
Hitcall
pow
16
−21
5.1
10
1
Response
100
50
0
−50
−100
1e−03
1e−02
1e−01
1e+00
1e+01
1e+02
conc (uM)

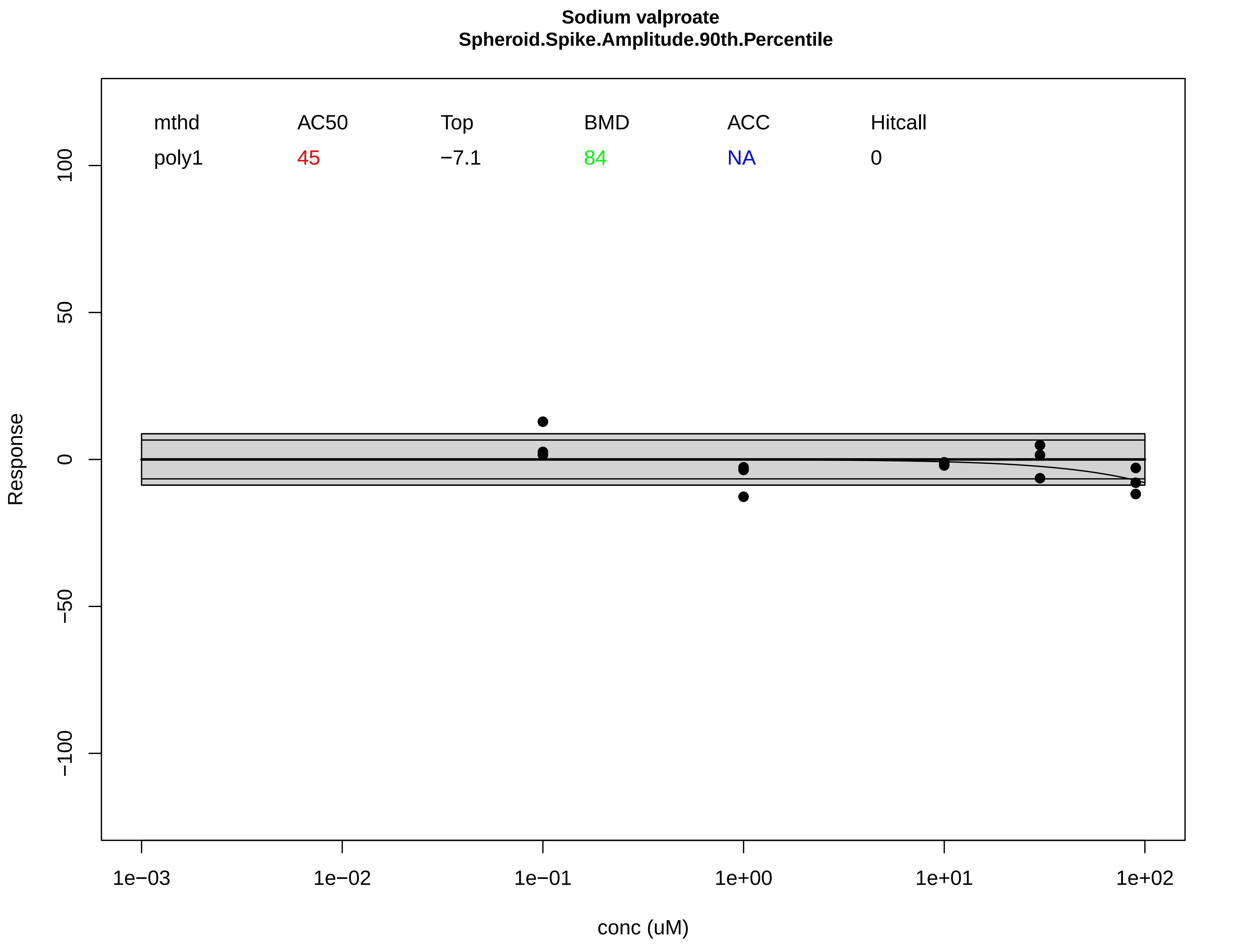
Sodium valproate
Spheroid.Spike.Amplitude.90th.Percentile
mthd
AC50
Top
BMD
ACC
Hitcall
poly1
45
−7.1
84
NA
0
100
50
0
−50
−100
Response
1e−03
1e−02
1e−01
1e+00
1e+01
1e+02
conc (uM)

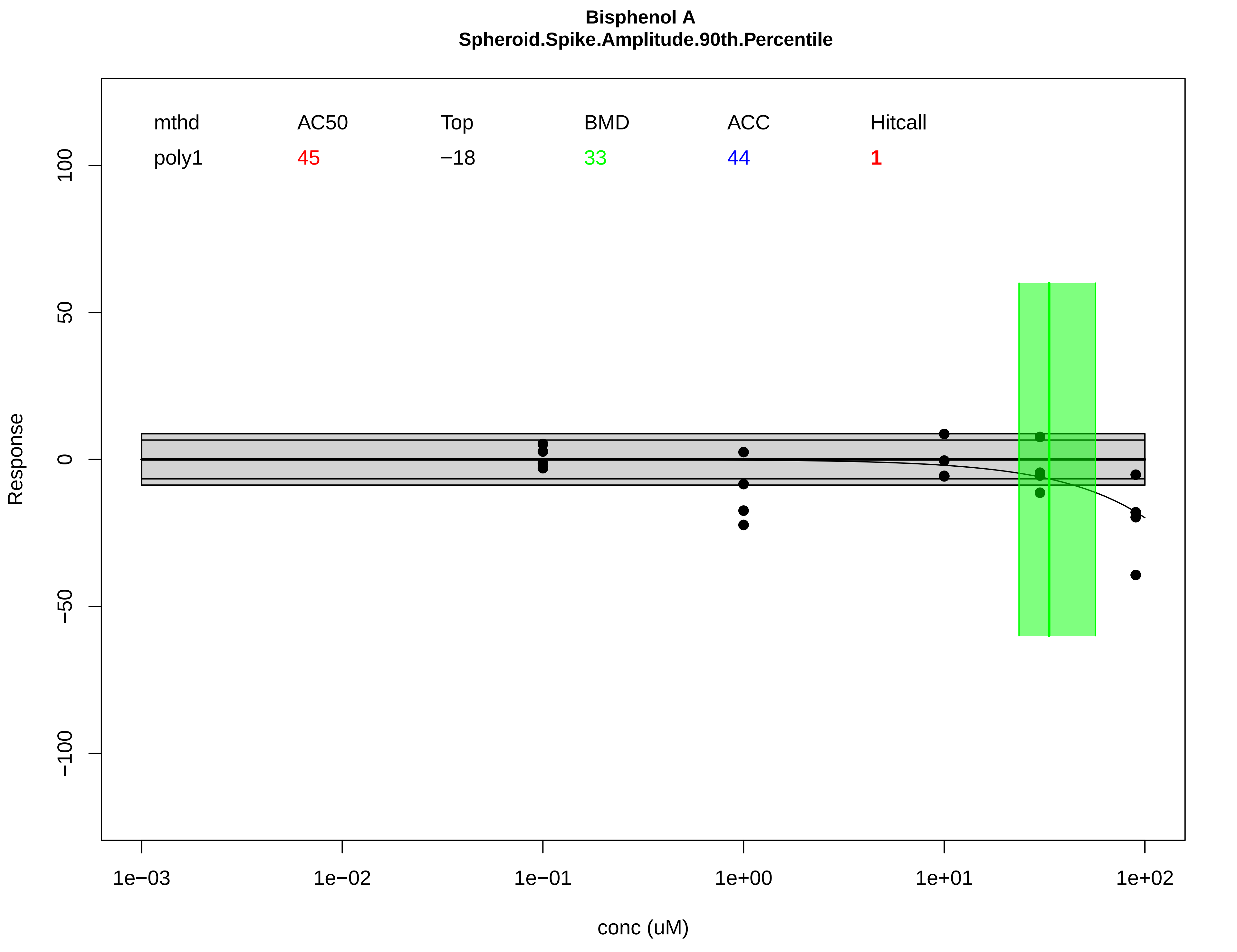

Bisphenol A
Spheroid.Spike.Amplitude.90th.Percentile
mthd
AC50
Top
BMD
ACC
Hitcall
poly1
45
−18
33
44
1
Response
100
50
0
−50
−100
1e−03
1e−02
1e−01
1e+00
1e+01
1e+02
conc (uM)

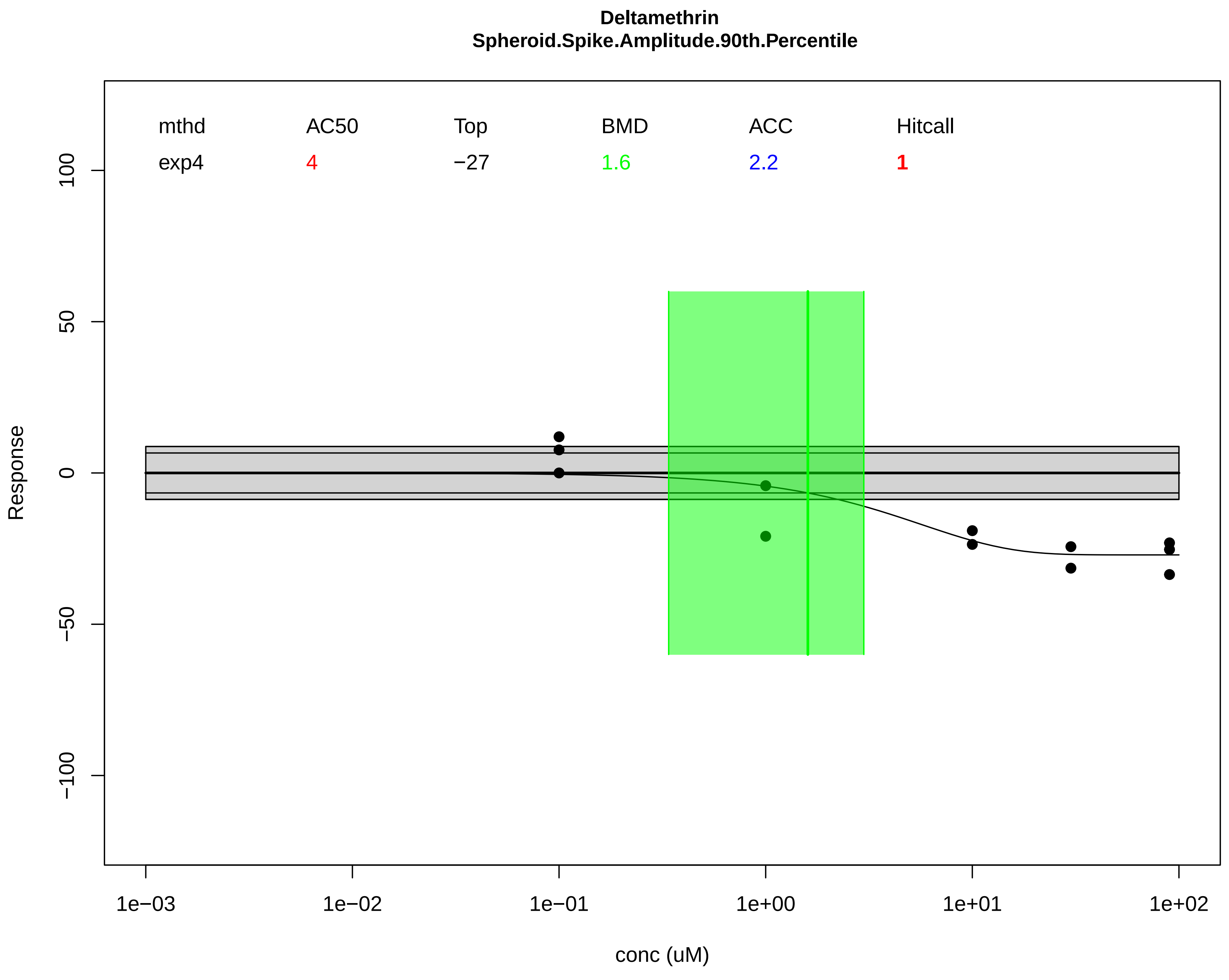
Deltamethrin
Spheroid.Spike.Amplitude.90th.Percentile
mthd
AC50
Top
BMD
ACC
Hitcall
exp4
4
−27
1.6
2.2
1
100
50
0
−50
−100
Response
1e−03
1e−02
1e−01
1e+00
1e+01
1e+02
conc (uM)

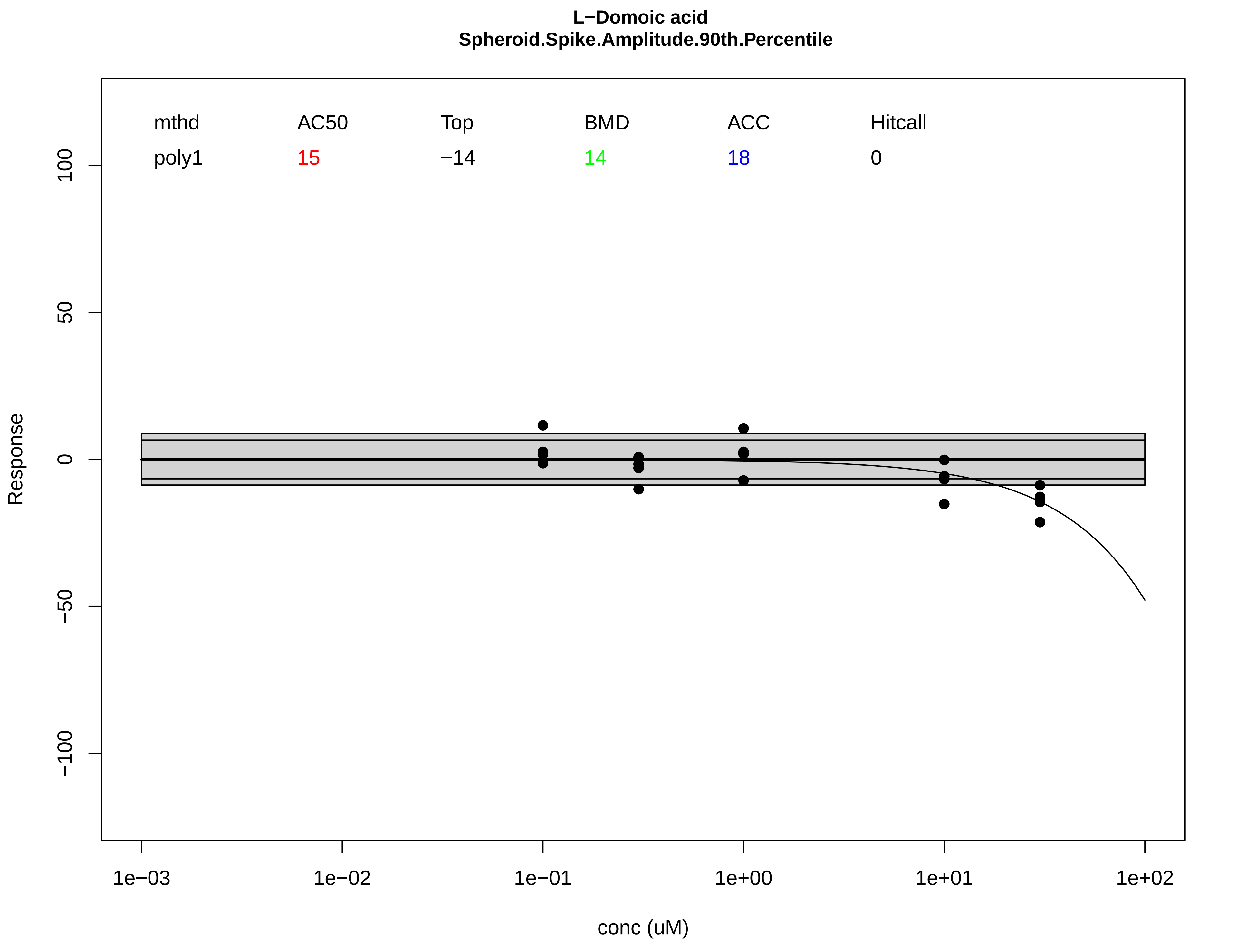

L−Domoic acid
Spheroid.Spike.Amplitude.90th.Percentile
mthd
AC50
Top
BMD
ACC
Hitcall
poly1
15
−14
14
18
0
100
50
0
−50
−100
Response
1e−03
1e−02
1e−01
1e+00
1e+01
1e+02
conc (uM)

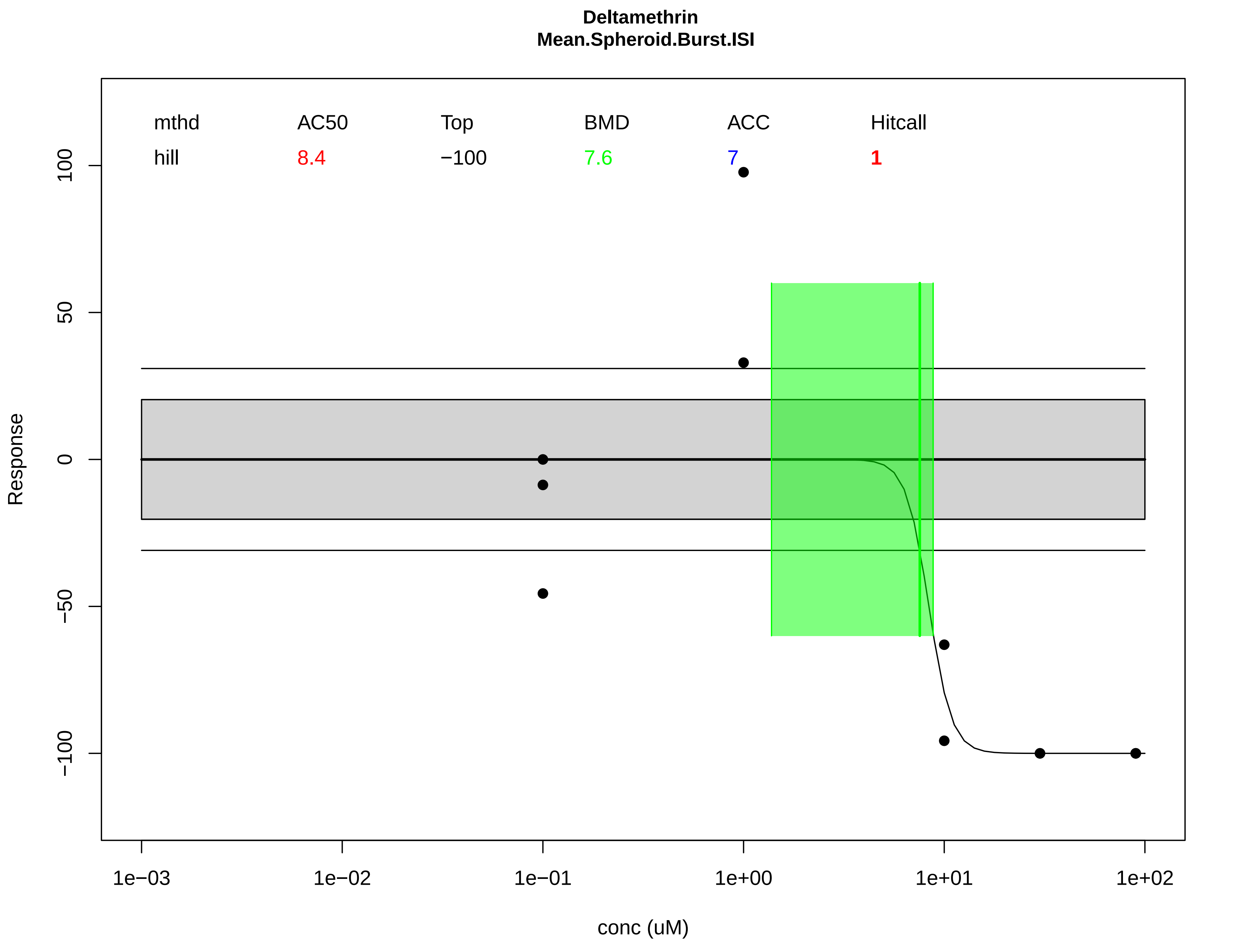

Deltamethrin
Mean.Spheroid.Burst.ISI
mthd
AC50
Top
BMD
ACC
Hitcall
hill
8.4
−100
7.6
7
1
100
50
0
−50
−100
Response
1e−03
1e−02
1e−01
1e+00
1e+01
1e+02
conc (uM)

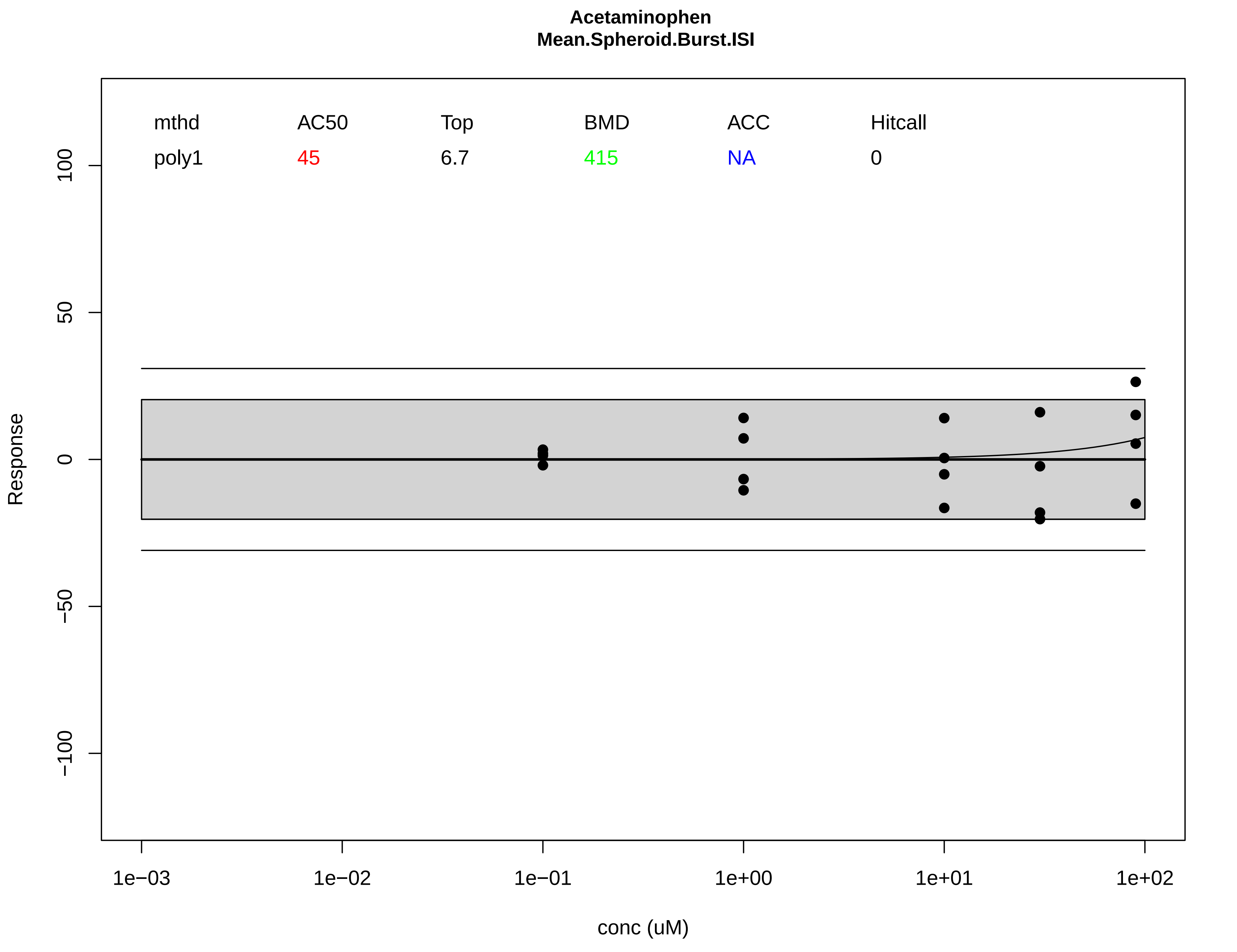

Acetaminophen
Mean.Spheroid.Burst.ISI
mthd
AC50
Top
BMD
ACC
Hitcall
poly1
45
6.7
415
NA
0
Response
100
50
0
−50
−100
1e−03
1e−02
1e−01
1e+00
1e+01
1e+02
conc (uM)

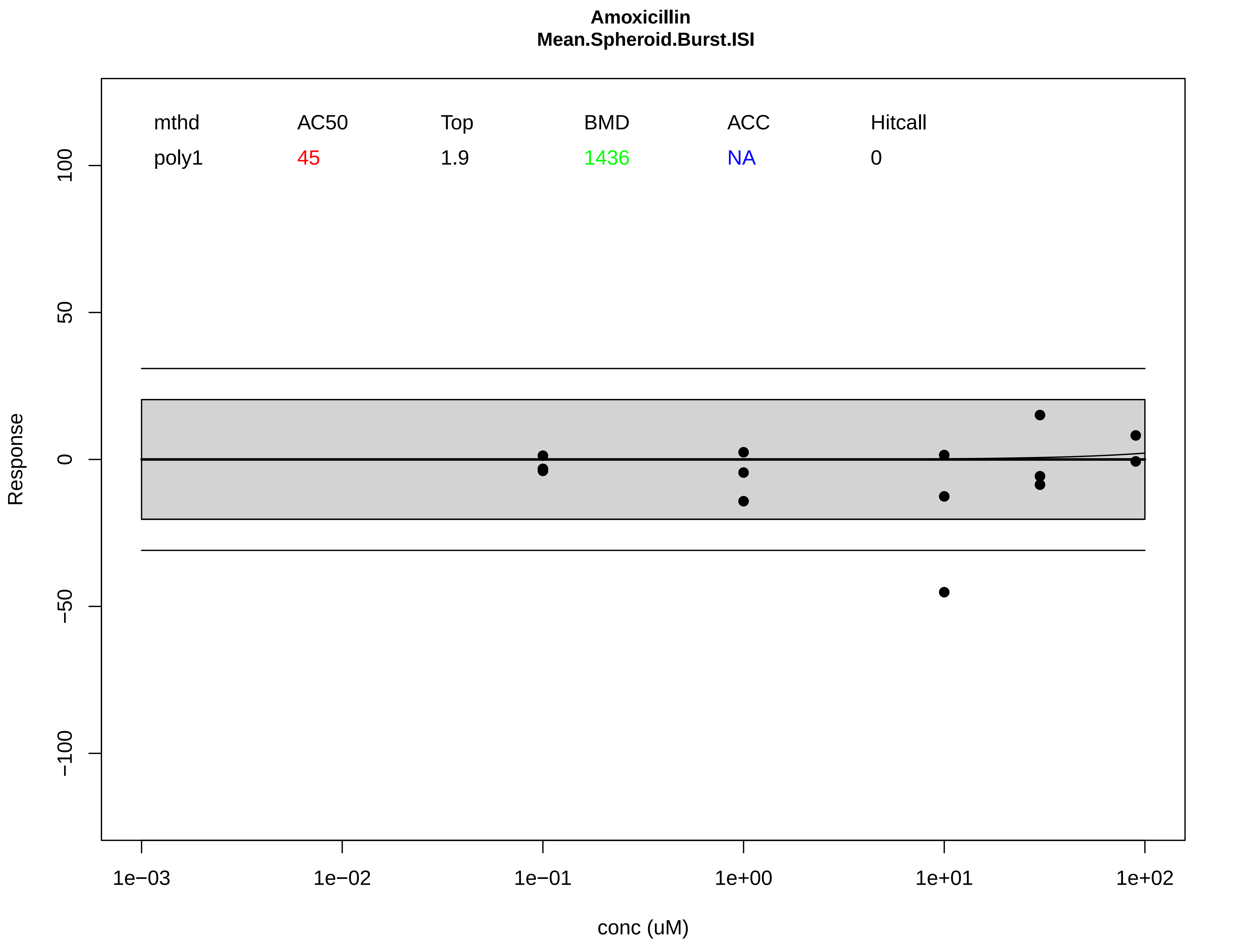
Amoxicillin
Mean.Spheroid.Burst.ISI
mthd
AC50
Top
BMD
ACC
Hitcall
poly1
45
1.9
1436
NA
0
100
50
0
−50
−100
Response
1e−03
1e−02
1e−01
1e+00
1e+01
1e+02
conc (uM)

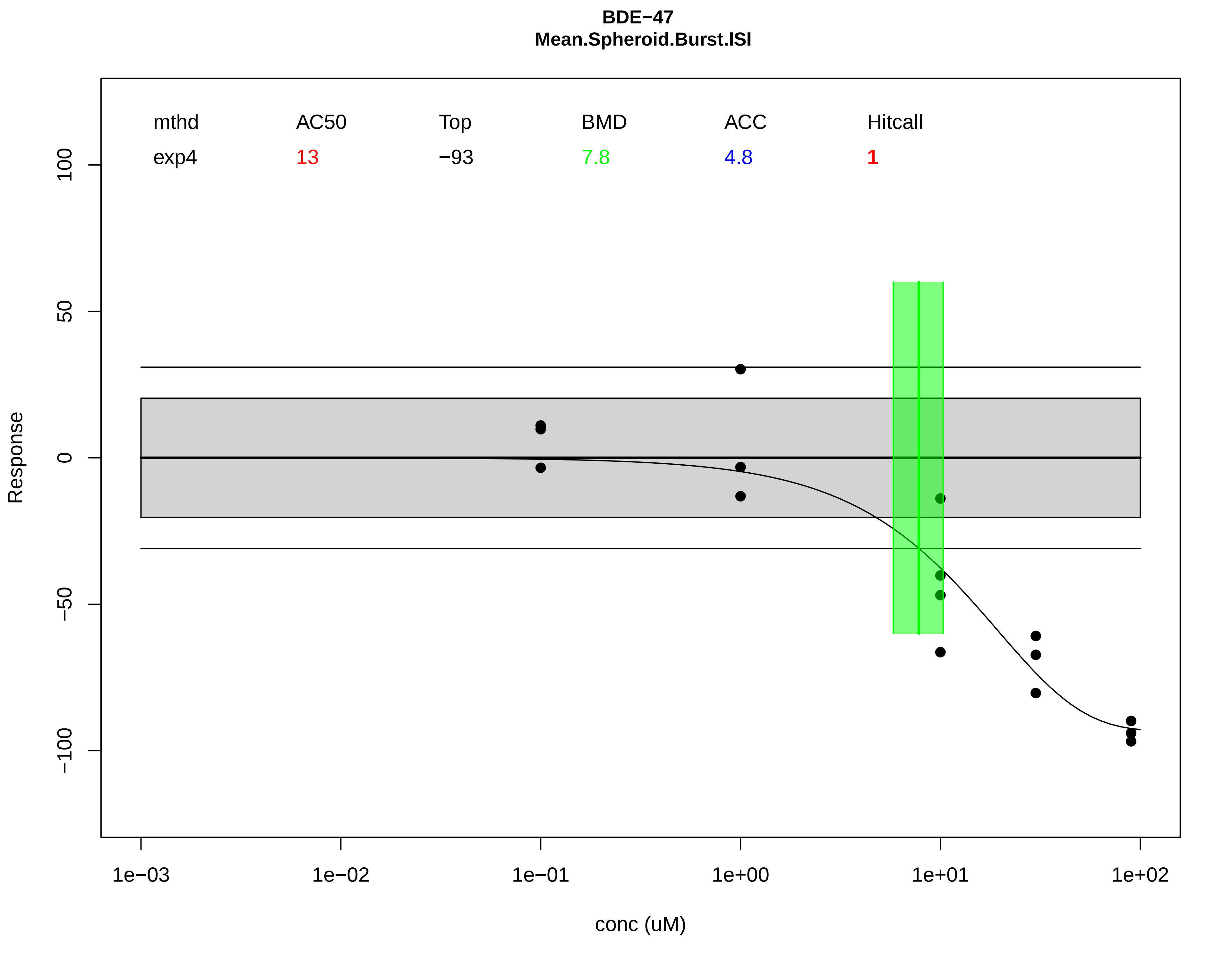

BDE−47
Mean.Spheroid.Burst.ISI
mthd
AC50
Top
BMD
ACC
Hitcall
exp4
13
−93
7.8
4.8
1
100
50
0
−50
−100
Response
1e−03
1e−02
1e−01
1e+00
1e+01
1e+02
conc (uM)

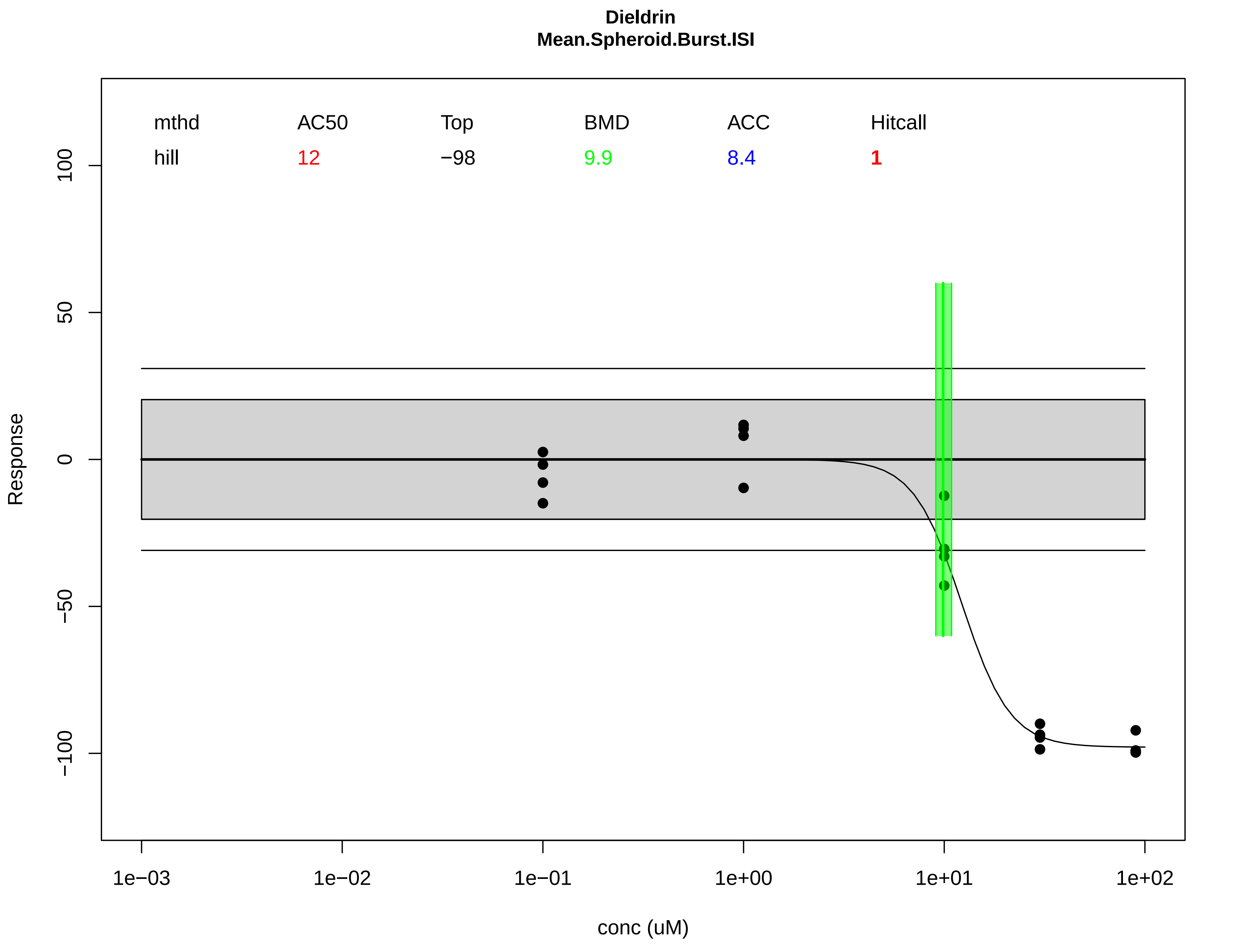
Dieldrin
Mean.Spheroid.Burst.ISI
mthd
hill
AC50
12
Top
−98
BMD
9.9
ACC
8.4
Hitcall
1
100
50
0
−50
−100
Response
1e−03
1e−02
1e−01
1e+00
1e+01
1e+02
conc (uM)

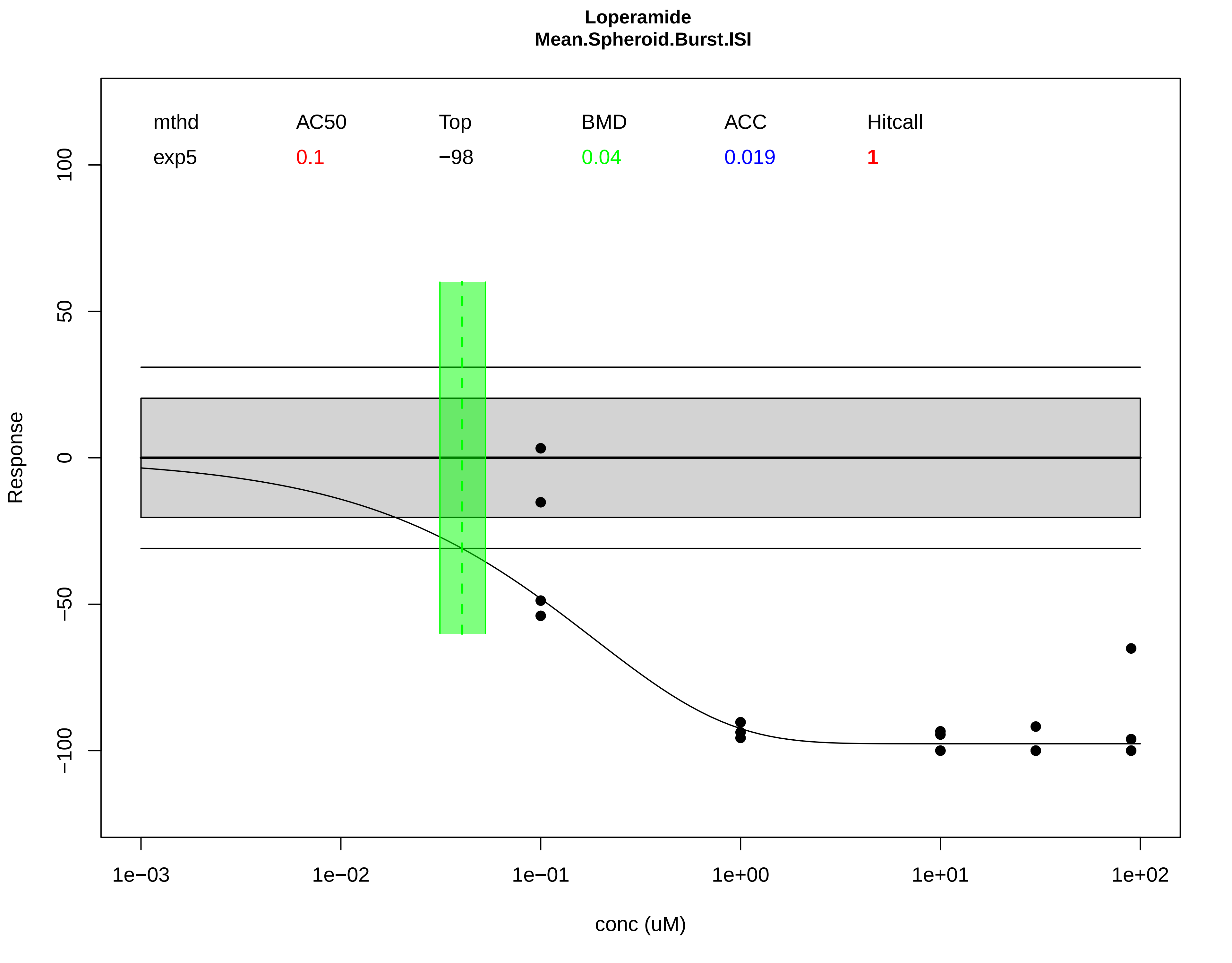
Loperamide
Mean.Spheroid.Burst.ISI
mthd
AC50
Top
BMD
ACC
Hitcall
exp5
0.1
−98
0.04
0.019
1
100
50
0
−50
−100
Response
1e−03
1e−02
1e−01
1e+00
1e+01
1e+02
conc (uM)

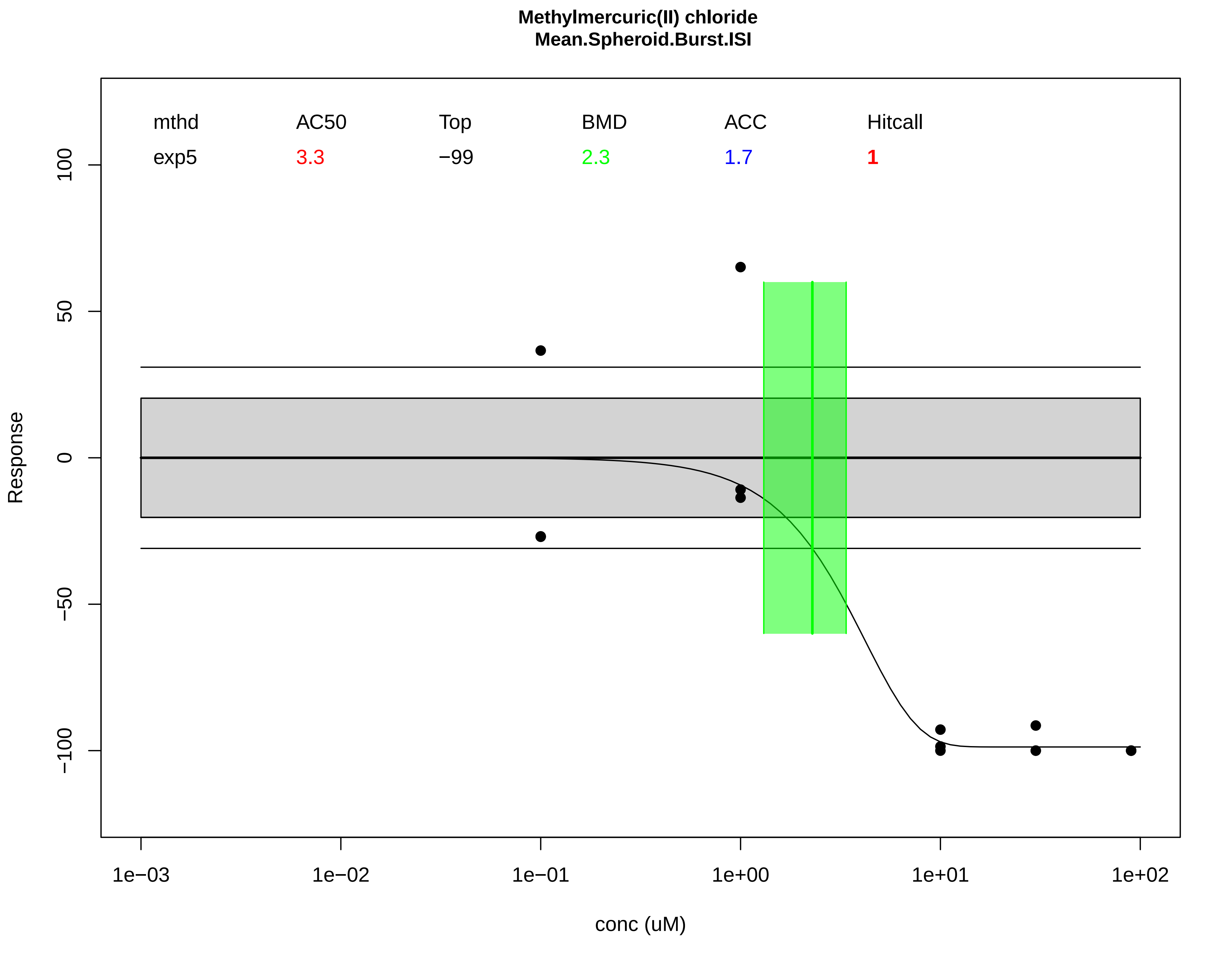

Methylmercuric(II) chloride
Mean.Spheroid.Burst.ISI
mthd
AC50
Top
BMD
ACC
Hitcall
exp5
3.3
−99
2.3
1.7
1
100
50
0
−50
−100
Response
1e−03
1e−02
1e−01
1e+00
1e+01
1e+02
conc (uM)

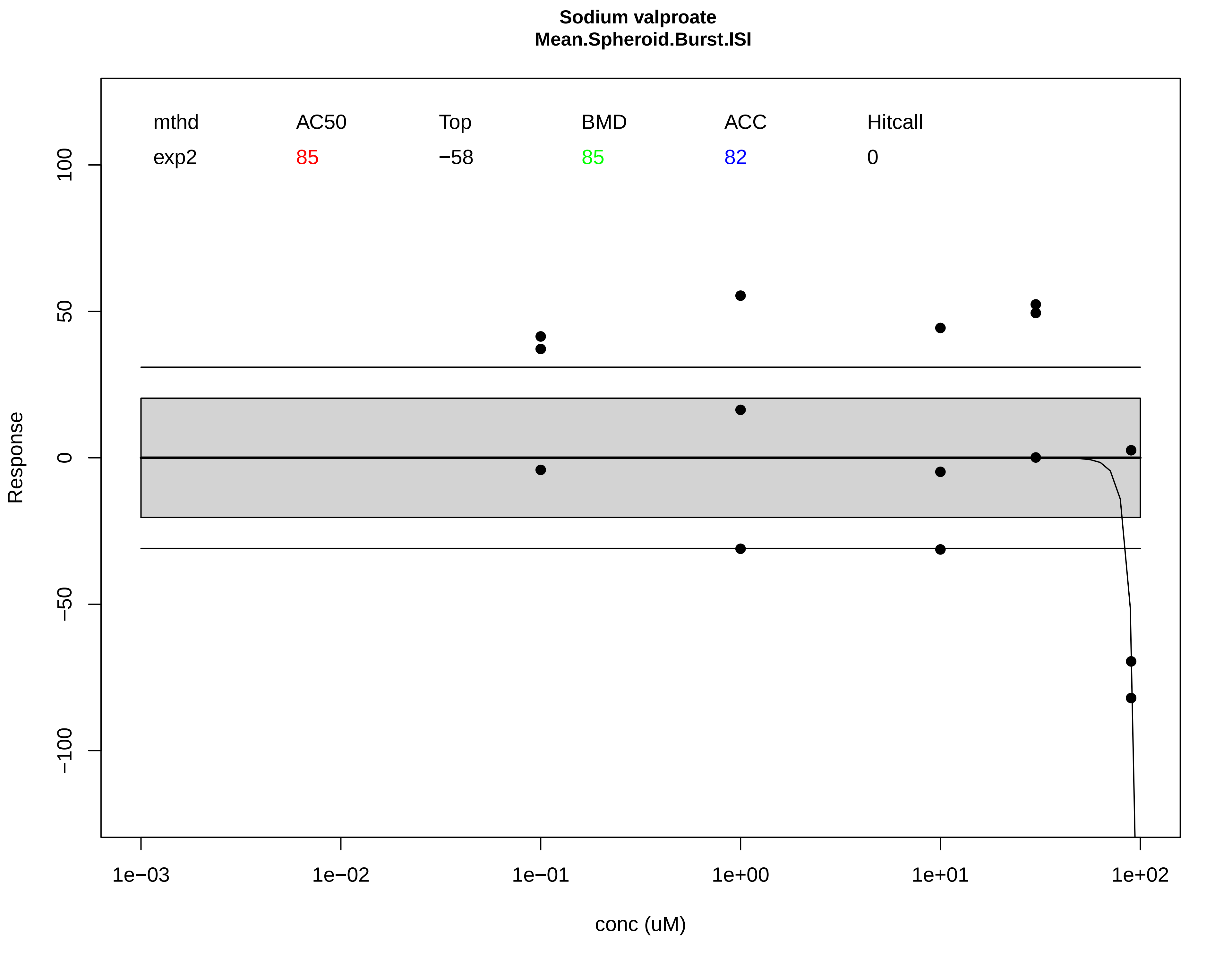

Sodium valproate
Mean.Spheroid.Burst.ISI
mthd
AC50
Top
BMD
ACC
Hitcall
exp2
85
−58
85
82
0
100
50
0
−50
−100
Response
1e−03
1e−02
1e−01
1e+00
1e+01
1e+02
conc (uM)

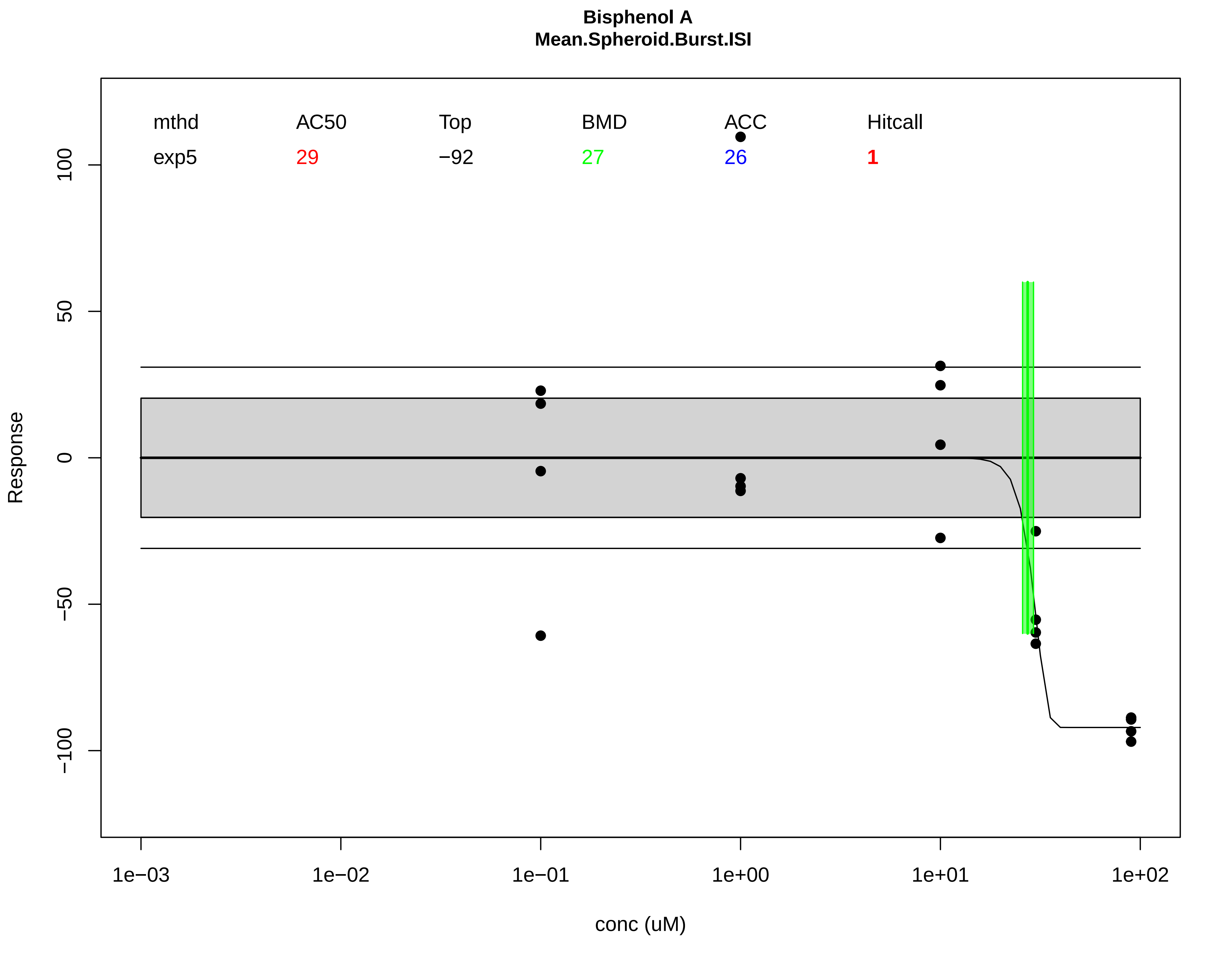

Bisphenol A
Mean.Spheroid.Burst.ISI
mthd
AC50
Top
BMD
ACC
Hitcall
exp5
29
−92
27
26
1
100
50
0
−50
−100
Response
1e−03
1e−02
1e−01
1e+00
1e+01
1e+02
conc (uM)

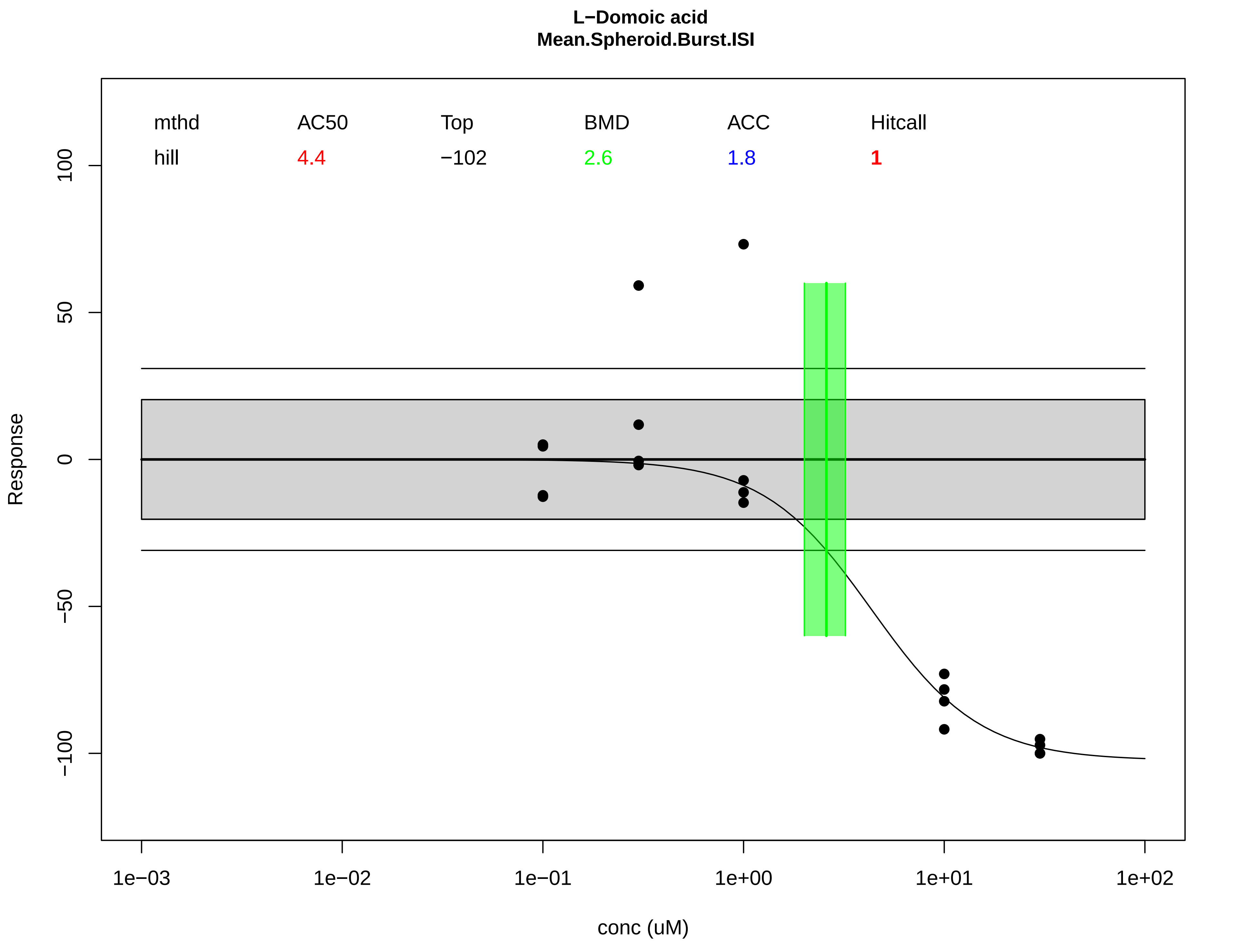

L−Domoic acid
Mean.Spheroid.Burst.ISI
mthd
AC50
Top
BMD
ACC
Hitcall
hill
4.4
−102
2.6
1.8
1
100
50
0
−50
−100
Response
1e−03
1e−02
1e−01
1e+00
1e+01
1e+02
conc (uM)

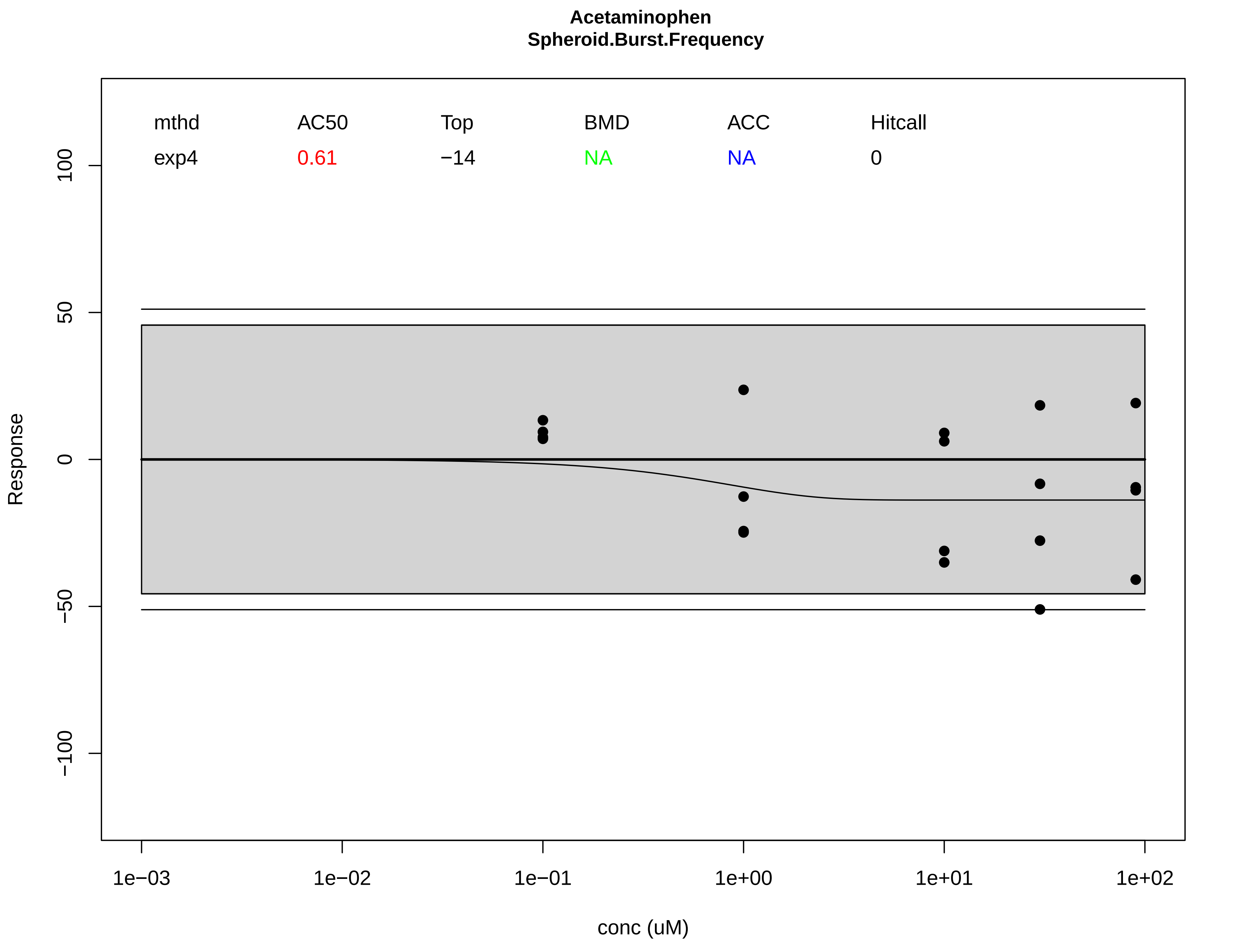
Acetaminophen
Spheroid.Burst.Frequency
mthd
AC50
Top
BMD
ACC
Hitcall
exp4
0.61
−14
NA
NA
0
Response
100
50
0
−50
−100
conc (uM)
1e−03
1e−02
1e−01
1e+00
1e+01
1e+02

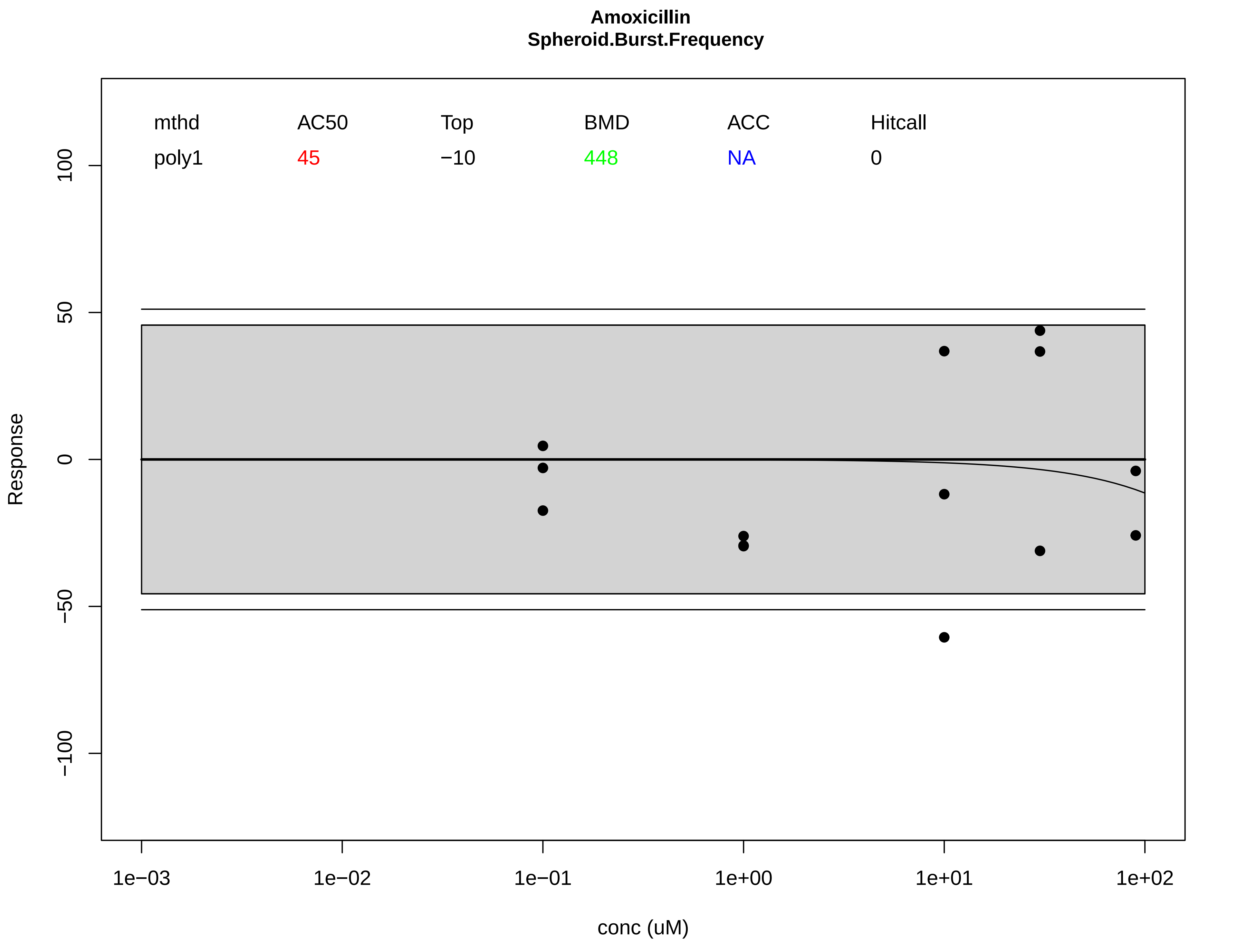

Amoxicillin
Spheroid.Burst.Frequency
mthd
AC50
Top
BMD
ACC
Hitcall
poly1
45
−10
448
NA
0
100
50
0
−50
−100
Response
1e−03
1e−02
1e−01
1e+00
1e+01
1e+02
conc (uM)

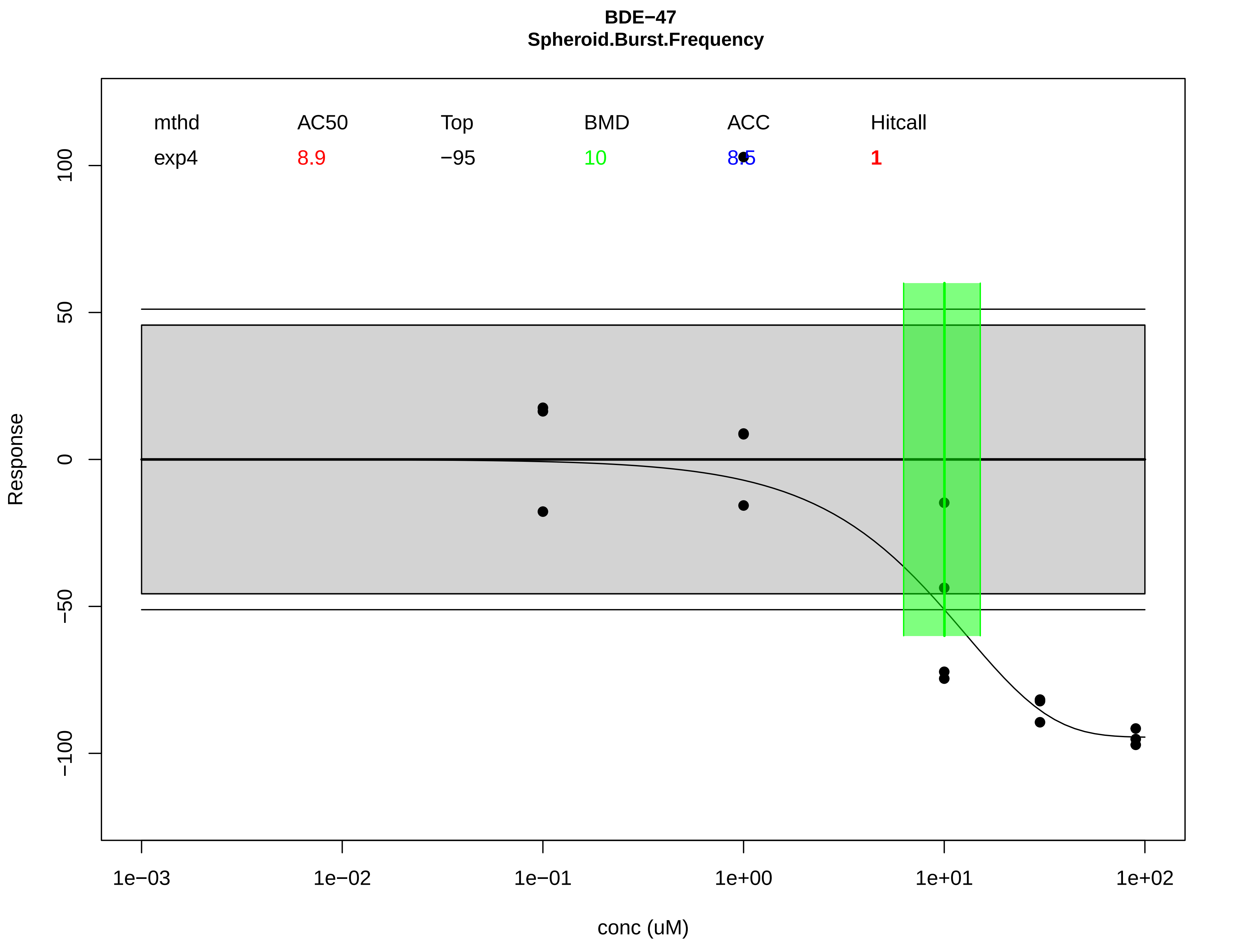

BDE−47
Spheroid.Burst.Frequency
mthd
AC50
Top
BMD
ACC
Hitcall
exp4
8.9
−95
10
8.5
1
100
50
0
−50
−100
Response
1e−03
1e−02
1e−01
1e+00
1e+01
1e+02
conc (uM)

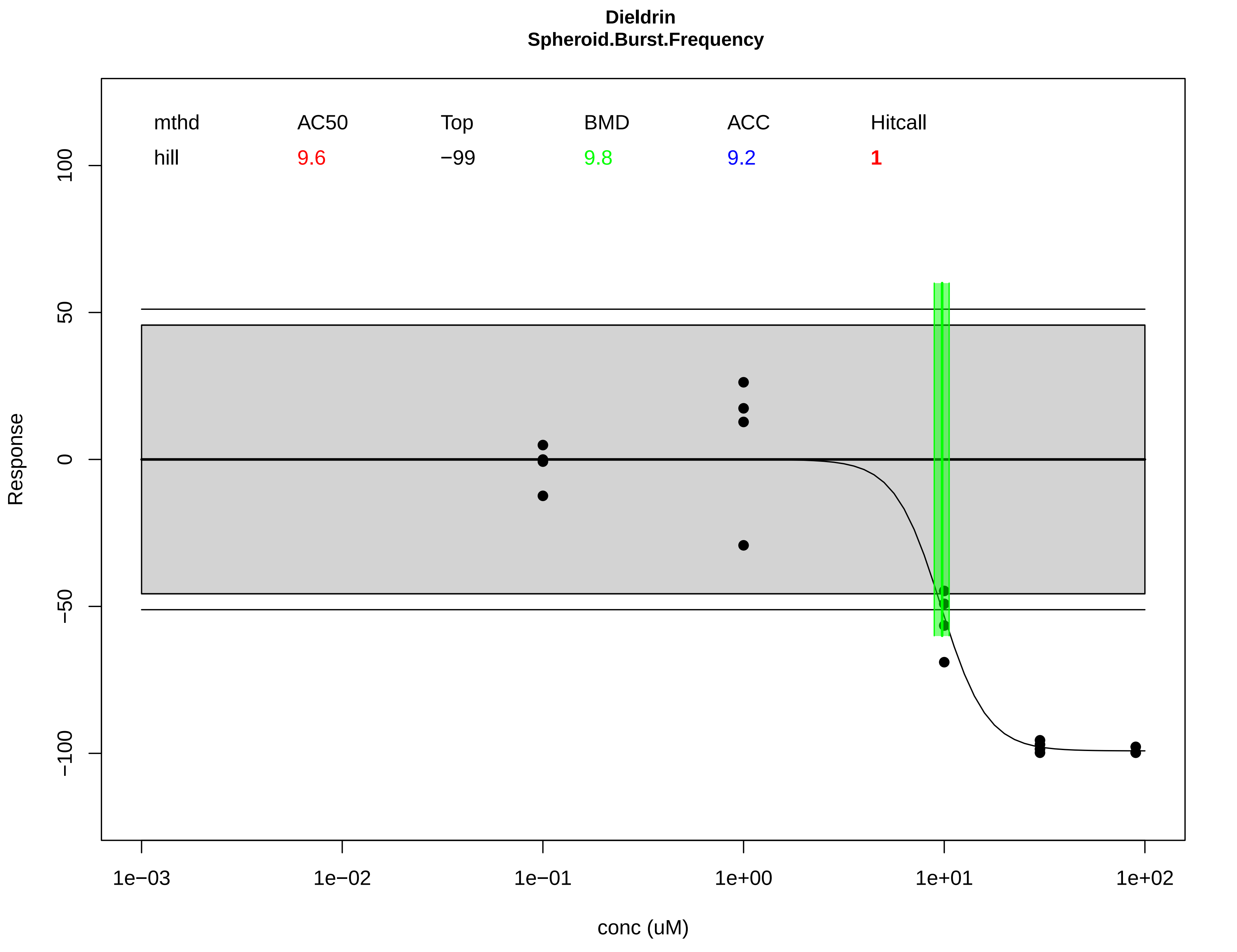

Dieldrin
Spheroid.Burst.Frequency
mthd
AC50
Top
BMD
ACC
Hitcall
hill
9.6
−99
9.8
9.2
1
100
50
0
−50
−100
Response
1e−03
1e−02
1e−01
1e+00
1e+01
1e+02
conc (uM)

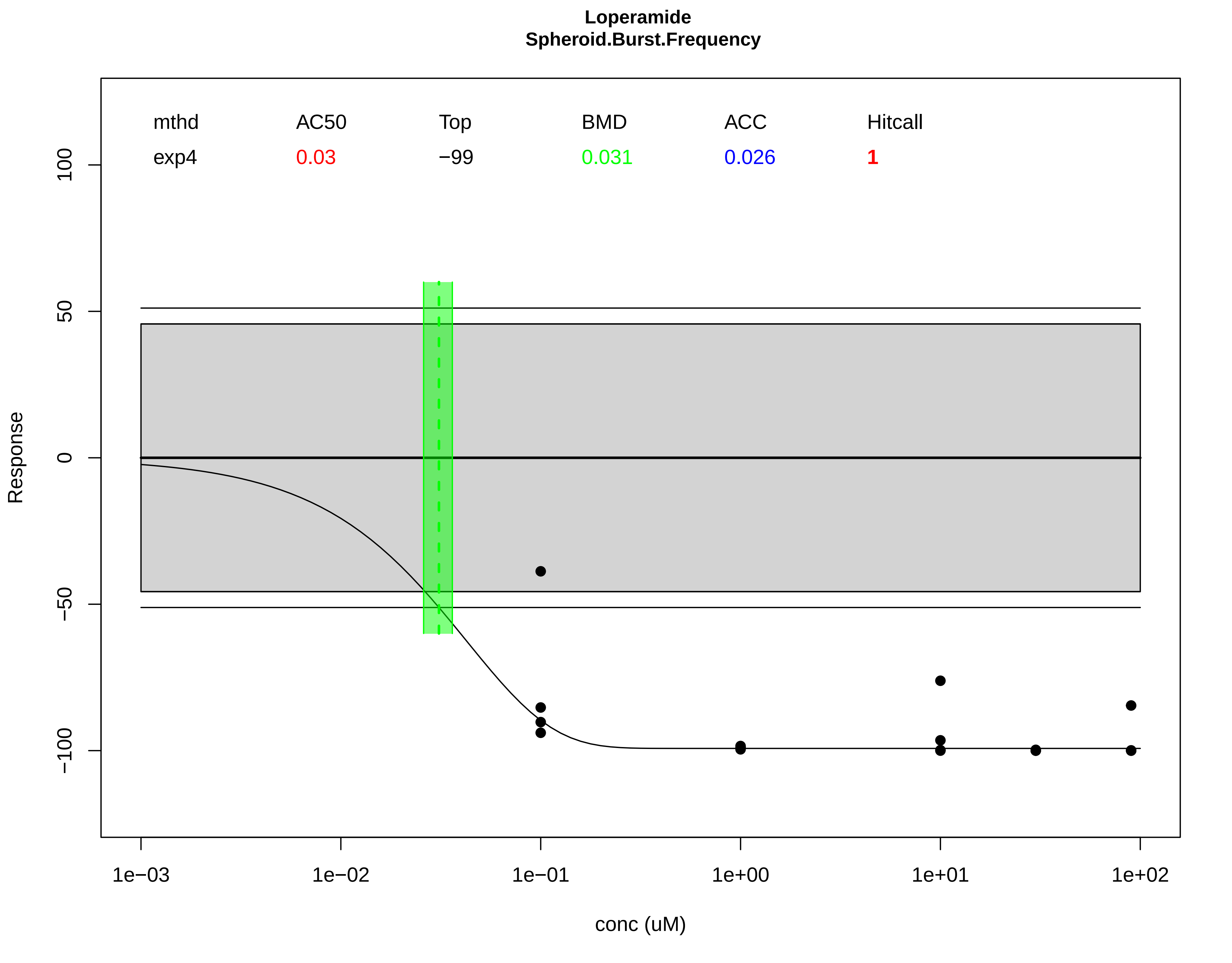

Loperamide
Spheroid.Burst.Frequency
mthd
AC50
Top
BMD
ACC
Hitcall
exp4
0.03
−99
0.031
0.026
1
100
50
0
−50
−100
Response
1e−03
1e−02
1e−01
1e+00
1e+01
1e+02
conc (uM)

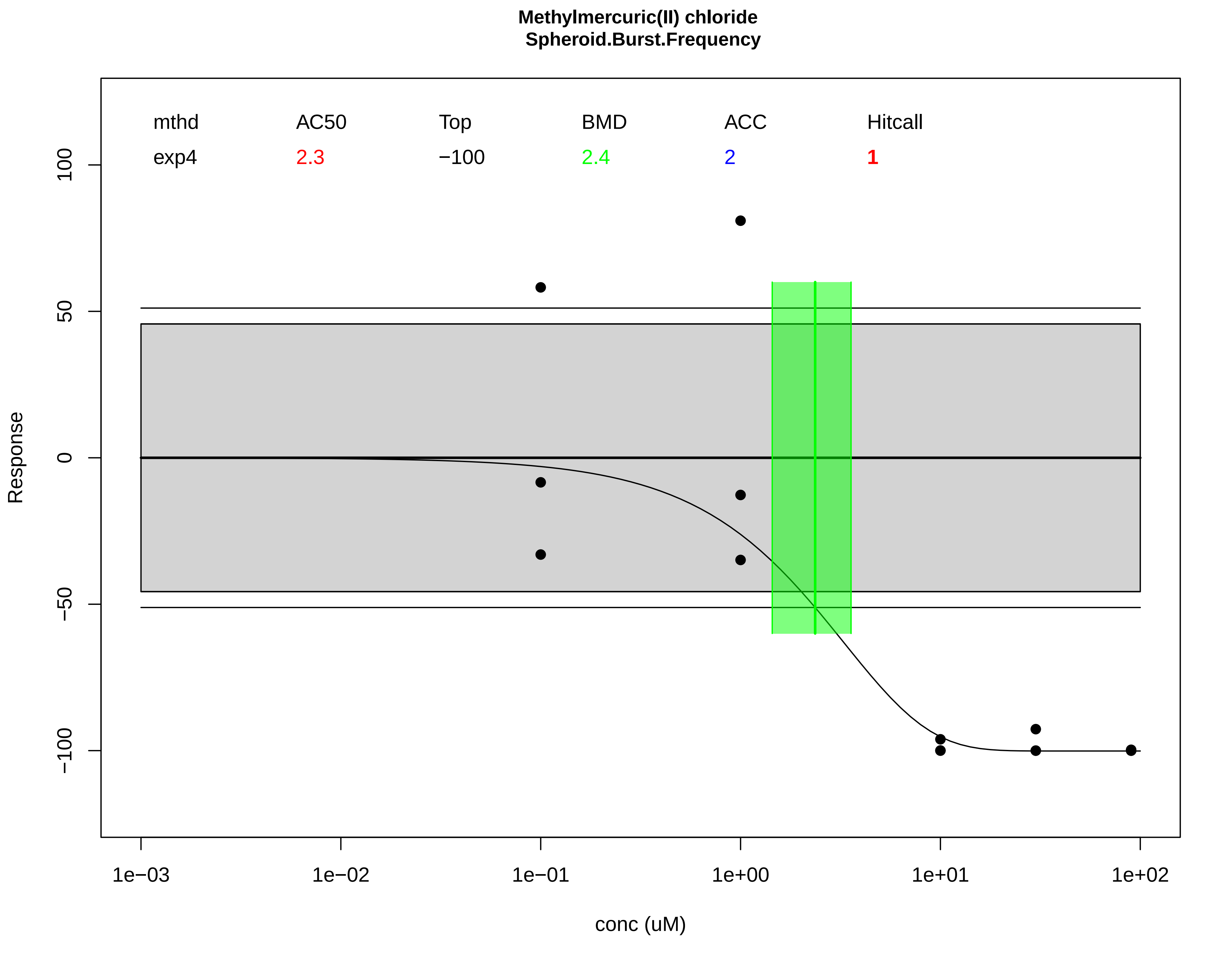
Methylmercuric(II) chloride
Spheroid.Burst.Frequency
mthd
AC50
Top
BMD
ACC
Hitcall
exp4
2.3
−100
2.4
2
1
Response
100
50
0
−50
−100
1e−03
1e−02
1e−01
1e+00
1e+01
1e+02
conc (uM)

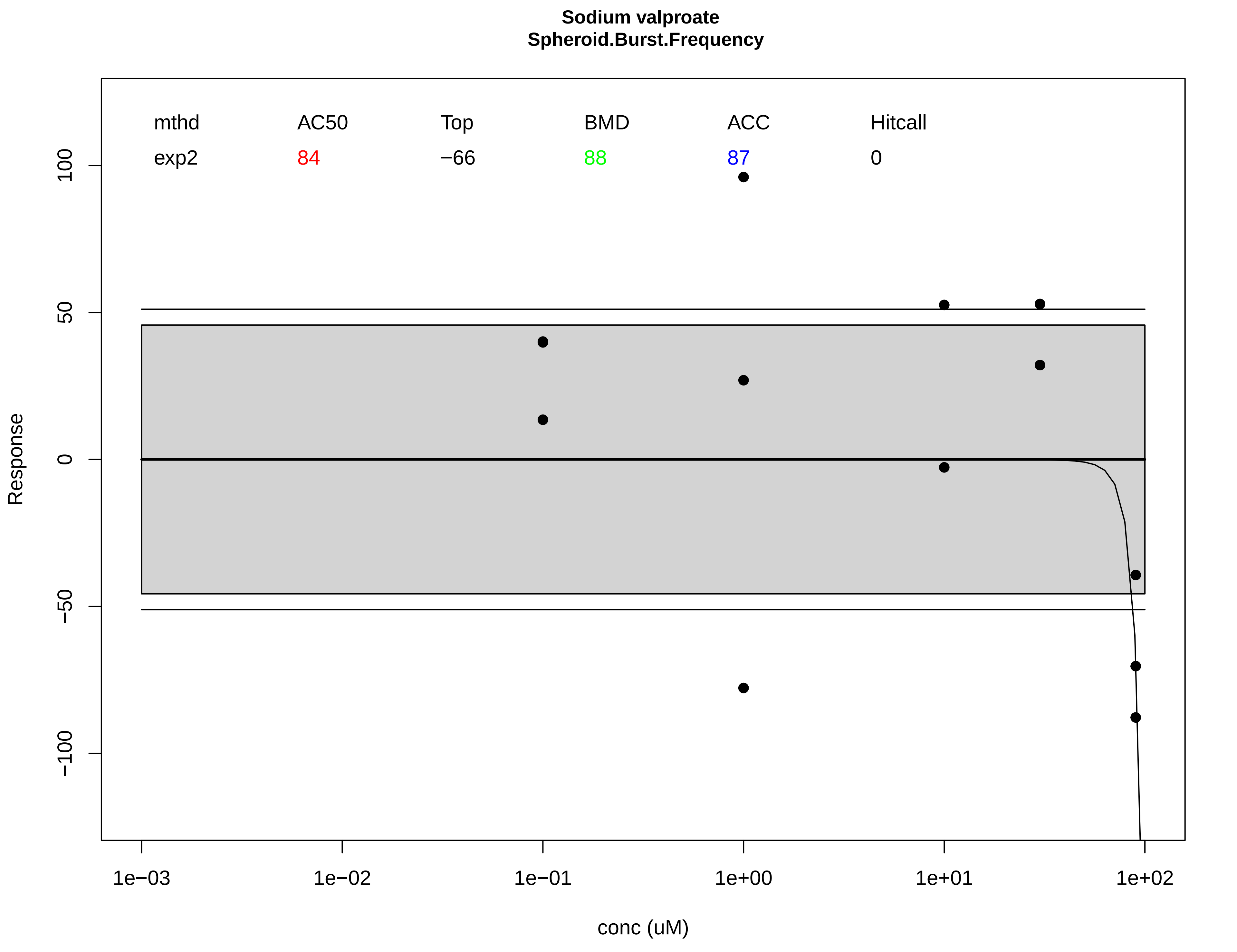
Sodium valproate
Spheroid.Burst.Frequency
mthd
AC50
Top
BMD
ACC
Hitcall
exp2
84
−66
88
87
0
Response
100
50
0
−50
−100
1e−03
1e−02
1e−01
1e+00
1e+01
1e+02
conc (uM)

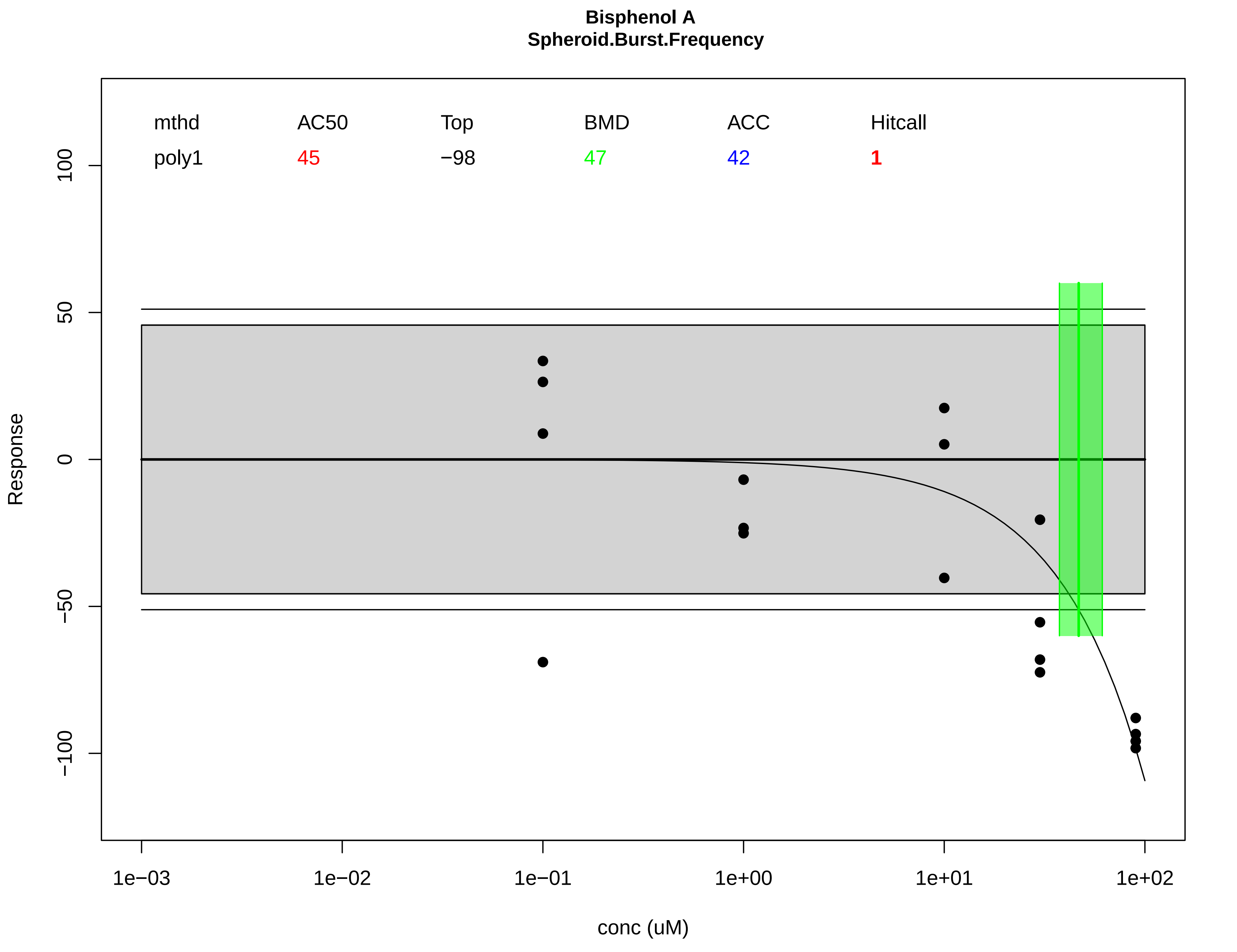
Bisphenol A
Spheroid.Burst.Frequency
mthd
AC50
Top
BMD
ACC
Hitcall
poly1
45
−98
47
42
1
Response
100
50
0
−50
−100
1e−03
1e−02
1e−01
1e+00
1e+01
1e+02
conc (uM)

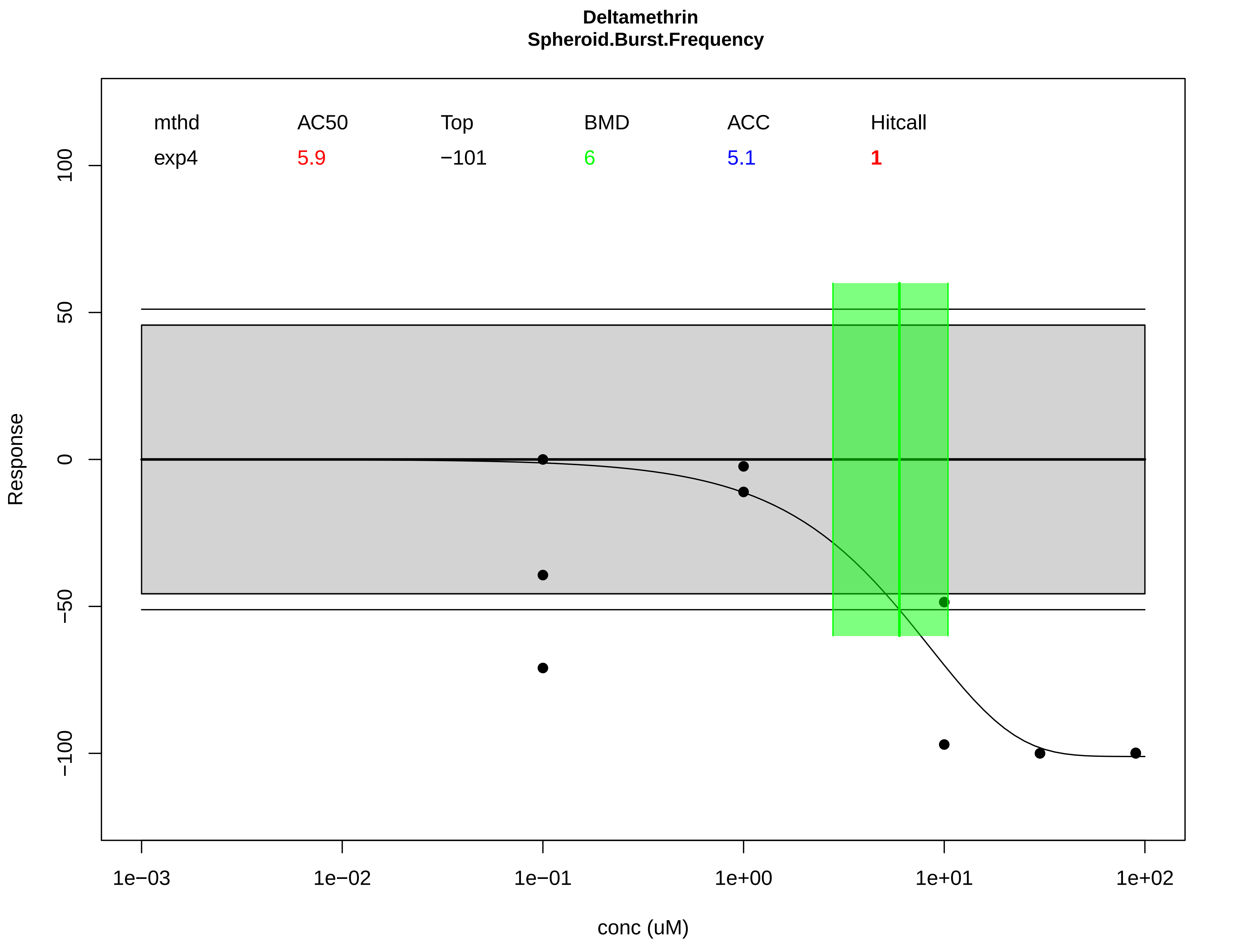

Deltamethrin
Spheroid.Burst.Frequency
mthd
AC50
Top
BMD
ACC
Hitcall
exp4
5.9
−101
6
5.1
1
Response
100
50
0
−50
−100
1e−03
1e−02
1e−01
1e+00
1e+01
1e+02
conc (uM)

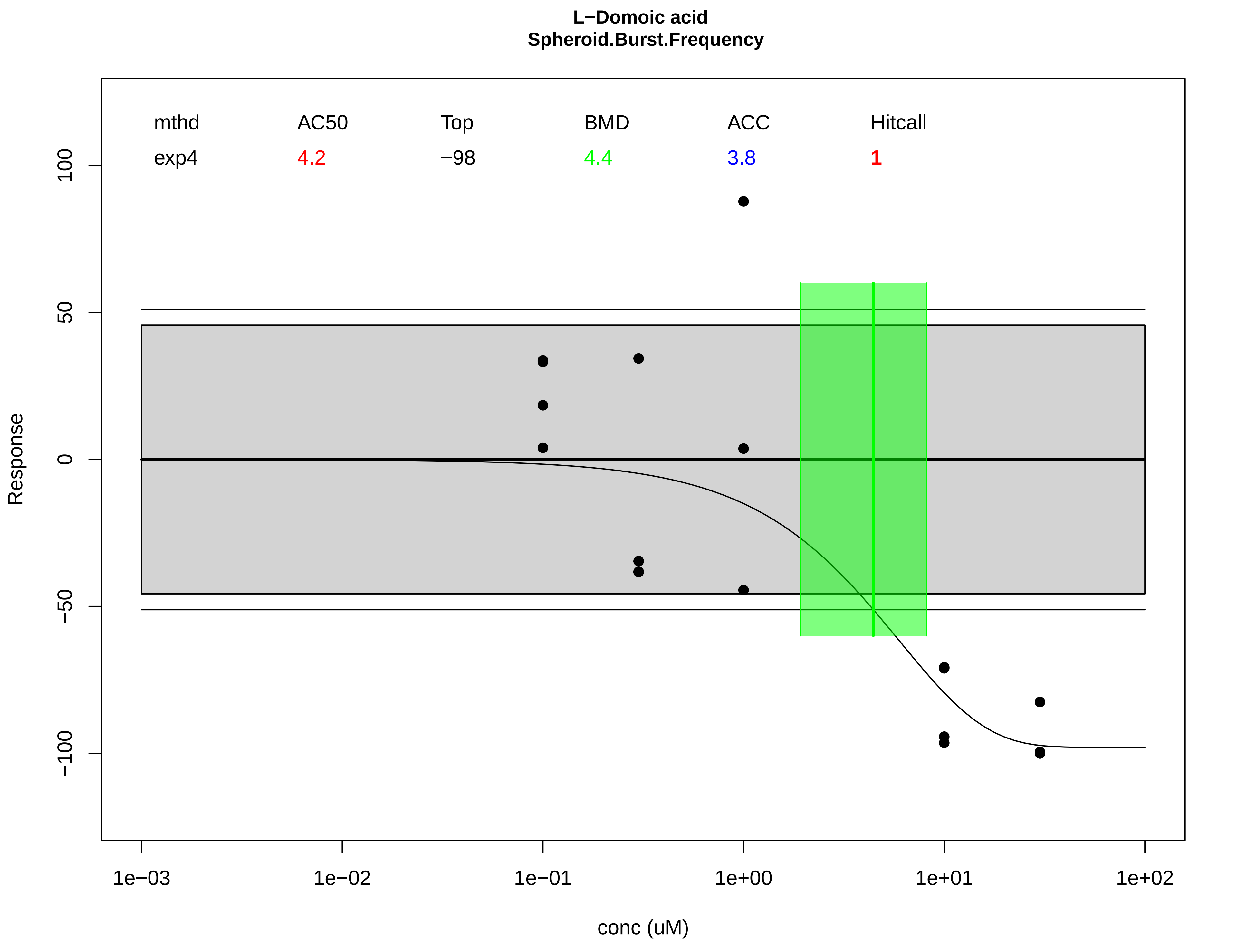
L−Domoic acid
Spheroid.Burst.Frequency
mthd
AC50
Top
BMD
ACC
Hitcall
exp4
4.2
−98
4.4
3.8
1
100
50
0
−50
−100
Response
1e−03
1e−02
1e−01
1e+00
1e+01
1e+02
conc (uM)

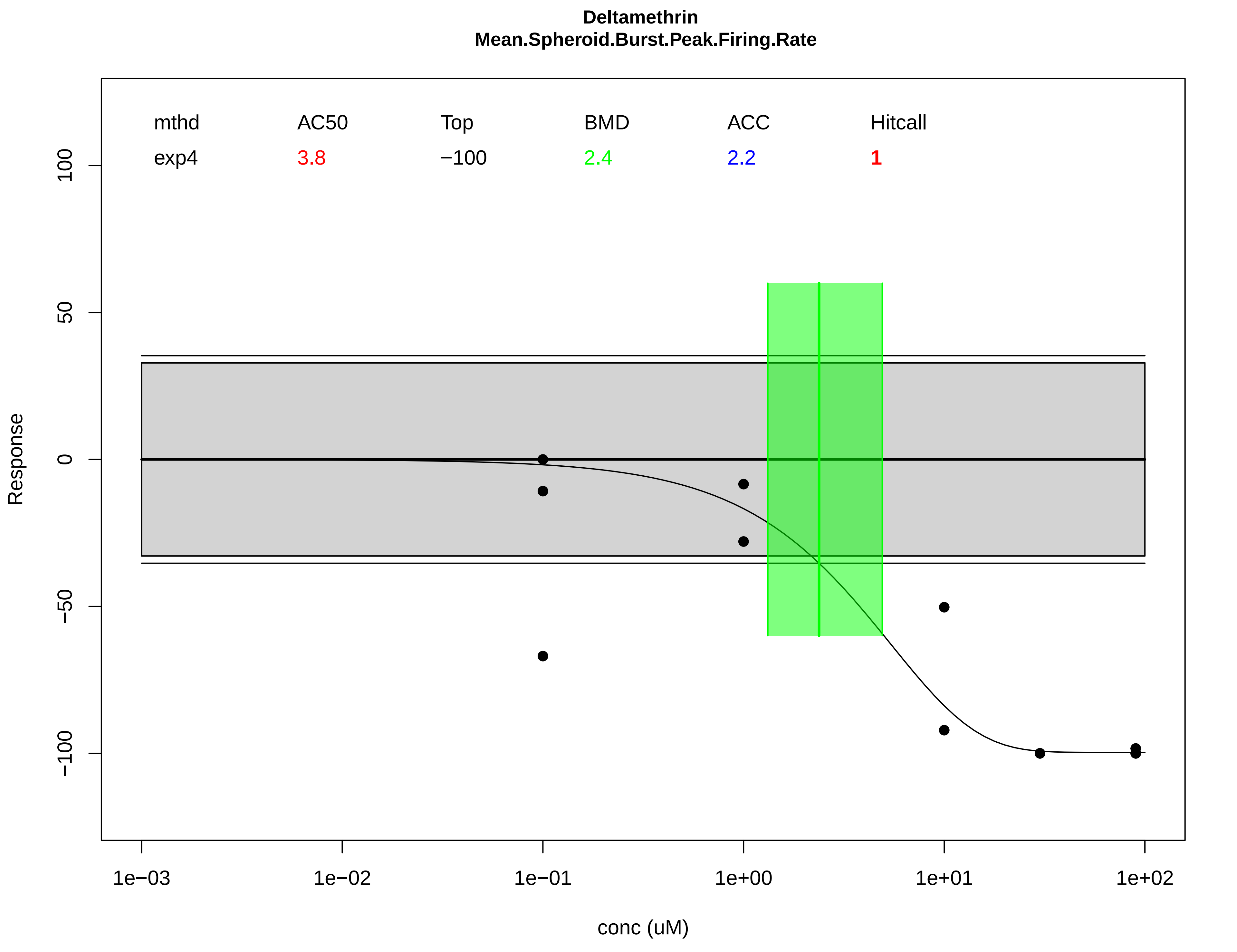

Deltamethrin
Mean.Spheroid.Burst.Peak.Firing.Rate
mthd
AC50
Top
BMD
ACC
Hitcall
exp4
3.8
−100
2.4
2.2
1
100
50
0
−50
−100
Response
1e−03
1e−02
1e−01
1e+00
1e+01
1e+02
conc (uM)

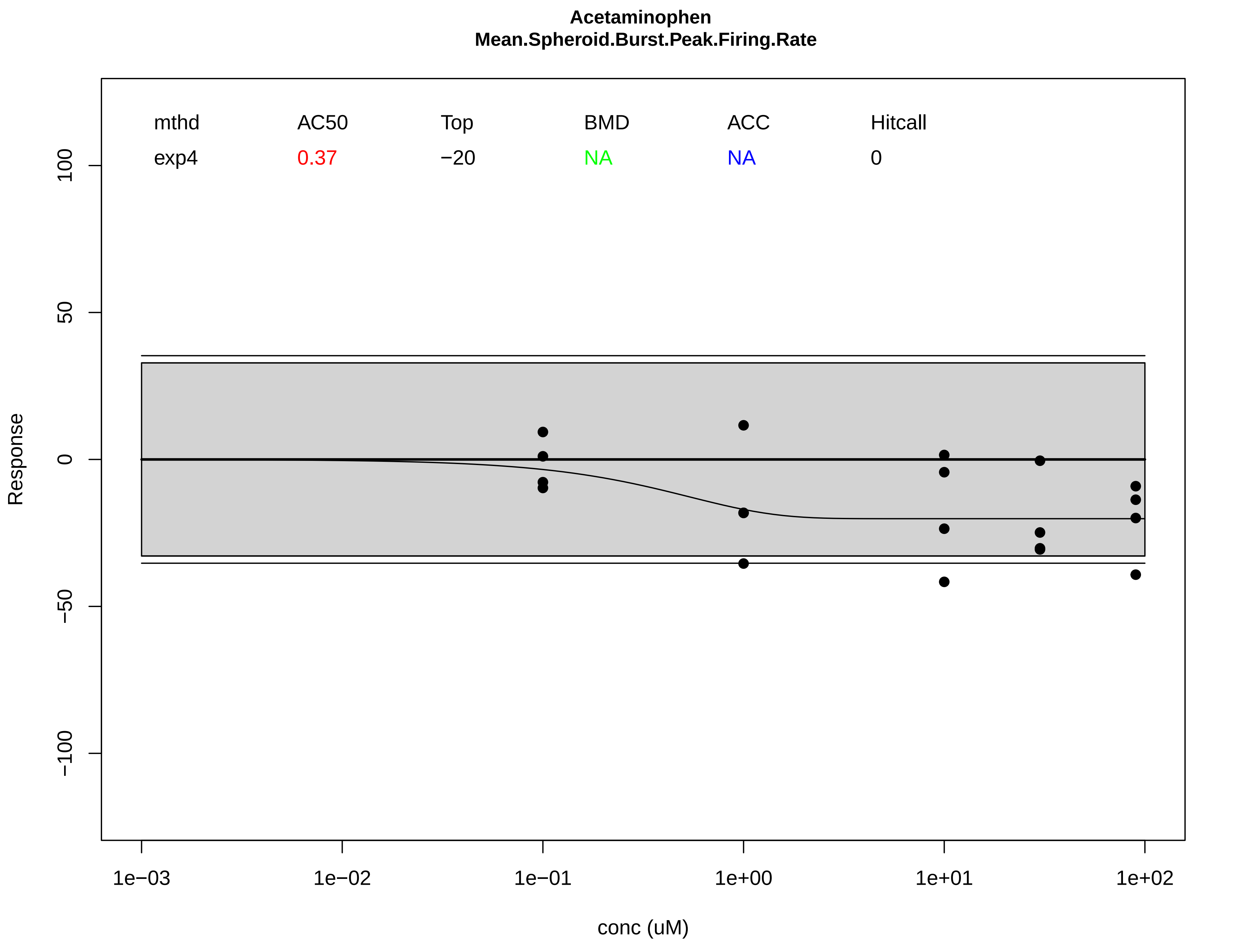
Acetaminophen
Mean.Spheroid.Burst.Peak.Firing.Rate
mthd
AC50
Top
BMD
ACC
Hitcall
exp4
0.37
−20
NA
NA
0
Response
100
50
0
−50
−100
1e−03
1e−02
1e−01
1e+00
1e+01
1e+02
conc (uM)

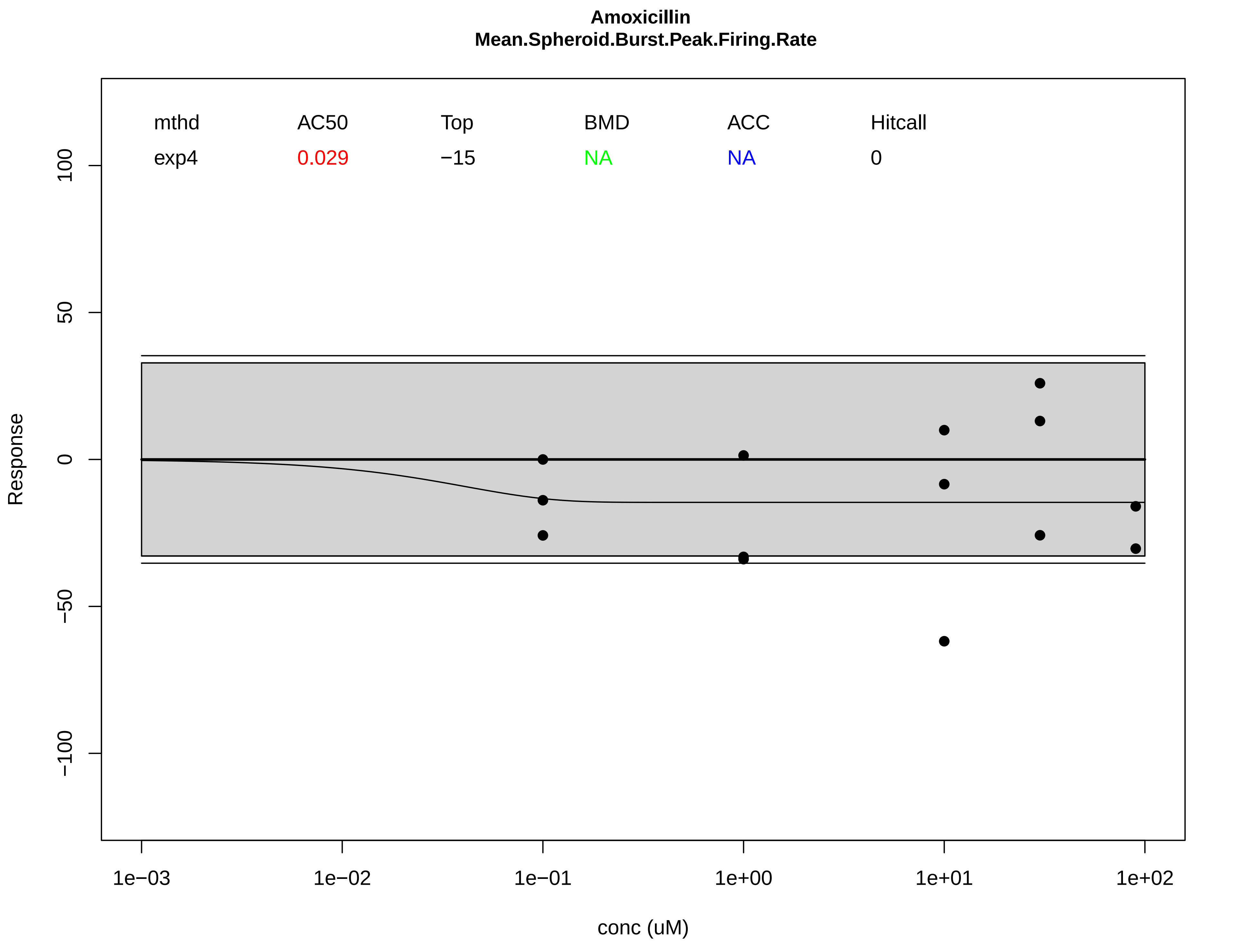
Amoxicillin
Mean.Spheroid.Burst.Peak.Firing.Rate
mthd
AC50
Top
BMD
ACC
Hitcall
exp4
0.029
−15
NA
NA
0
100
50
0
−50
−100
Response
1e−03
1e−02
1e−01
1e+00
1e+01
1e+02
conc (uM)

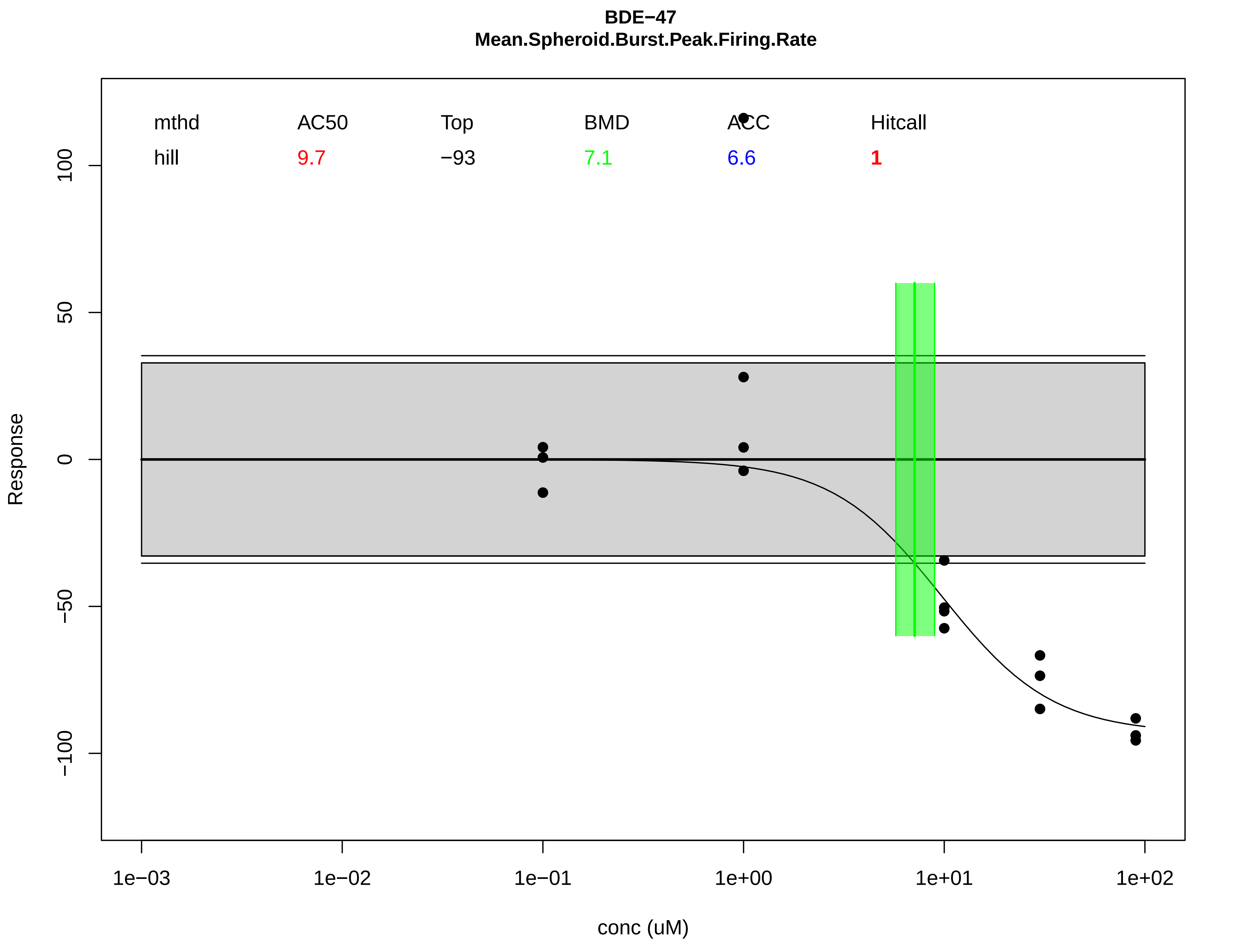
BDE−47
Mean.Spheroid.Burst.Peak.Firing.Rate
mthd
AC50
Top
BMD
ACC
Hitcall
hill
9.7
−93
7.1
6.6
1
100
50
0
−50
−100
Response
1e−03
1e−02
1e−01
1e+00
1e+01
1e+02
conc (uM)

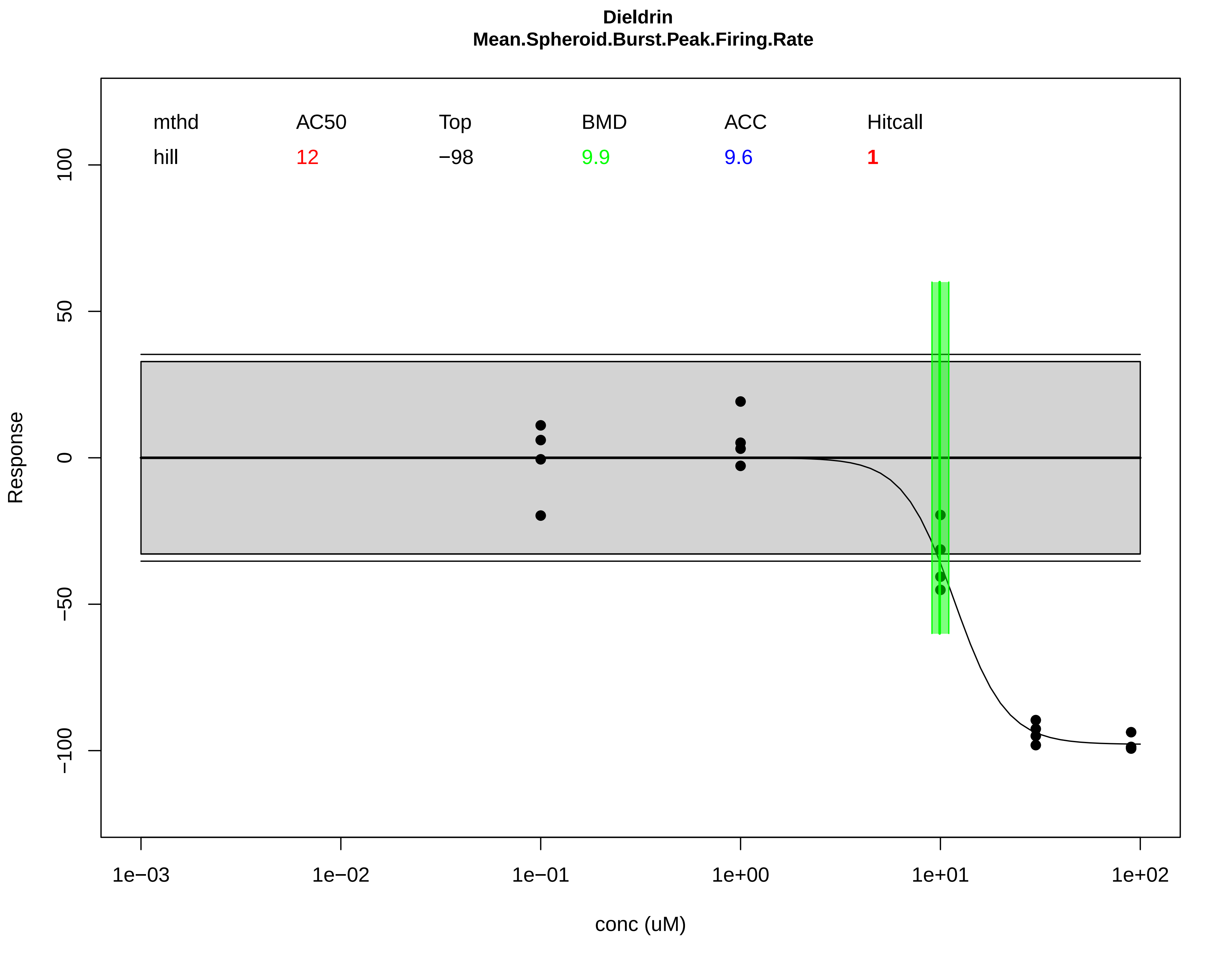
Dieldrin
Mean.Spheroid.Burst.Peak.Firing.Rate
mthd
AC50
Top
BMD
ACC
Hitcall
hill
12
−98
9.9
9.6
1
100
50
0
−50
−100
Response
1e−03
1e−02
1e−01
1e+00
1e+01
1e+02
conc (uM)

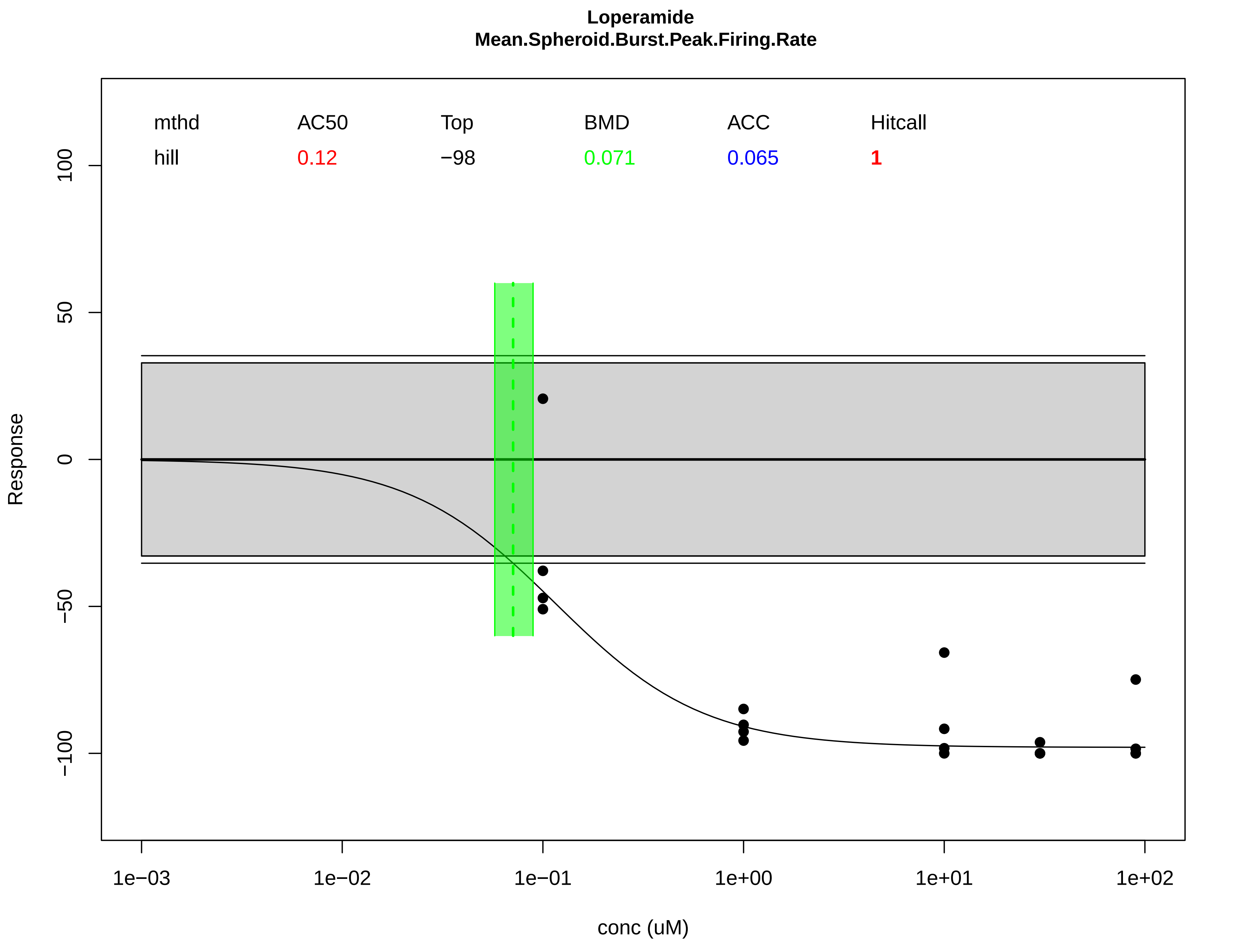

Loperamide
Mean.Spheroid.Burst.Peak.Firing.Rate
mthd
AC50
Top
BMD
ACC
Hitcall
hill
0.12
−98
0.071
0.065
1
Response
100
50
0
−50
−100
1e−03
1e−02
1e−01
1e+00
1e+01
1e+02
conc (uM)

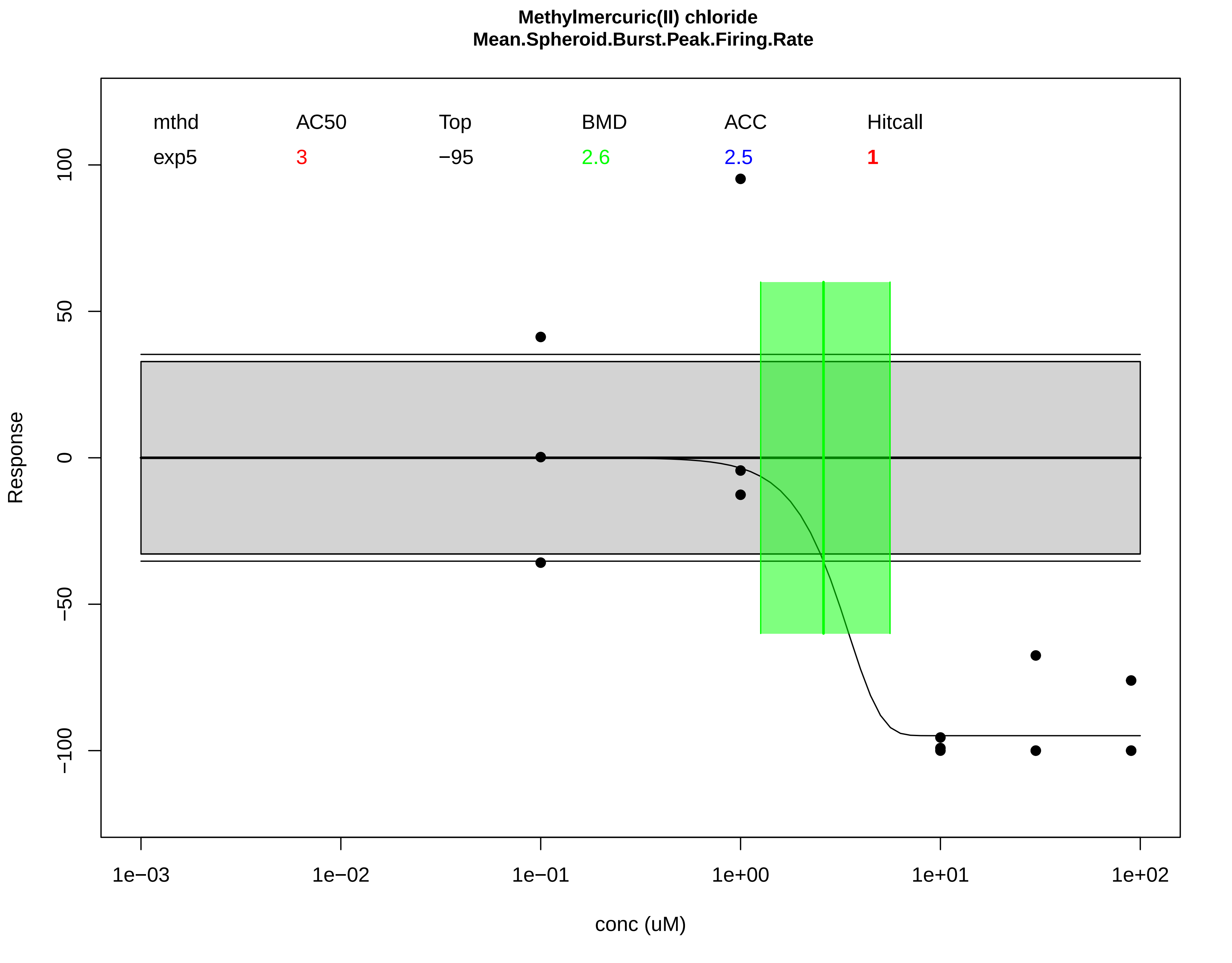

Methylmercuric(II) chloride
Mean.Spheroid.Burst.Peak.Firing.Rate
mthd
AC50
Top
BMD
ACC
Hitcall
exp5
3
−95
2.6
2.5
1
100
50
0
−50
−100
Response
1e−03
1e−02
1e−01
1e+00
1e+01
1e+02
conc (uM)

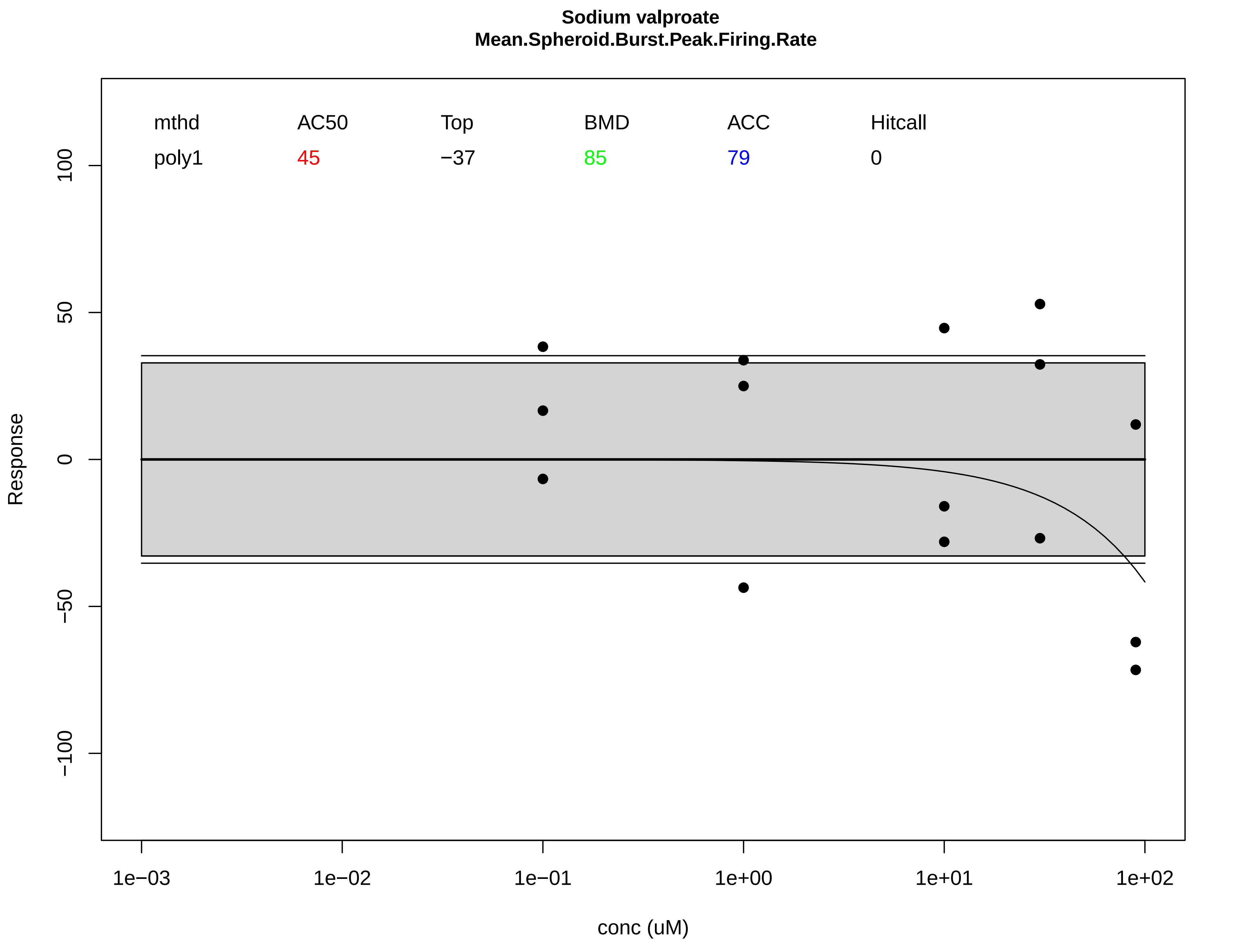
Sodium valproate
Mean.Spheroid.Burst.Peak.Firing.Rate
mthd
AC50
Top
BMD
ACC
Hitcall
poly1
45
−37
85
79
0
100
50
0
−50
−100
Response
1e−03
1e−02
1e−01
1e+00
1e+01
1e+02
conc (uM)

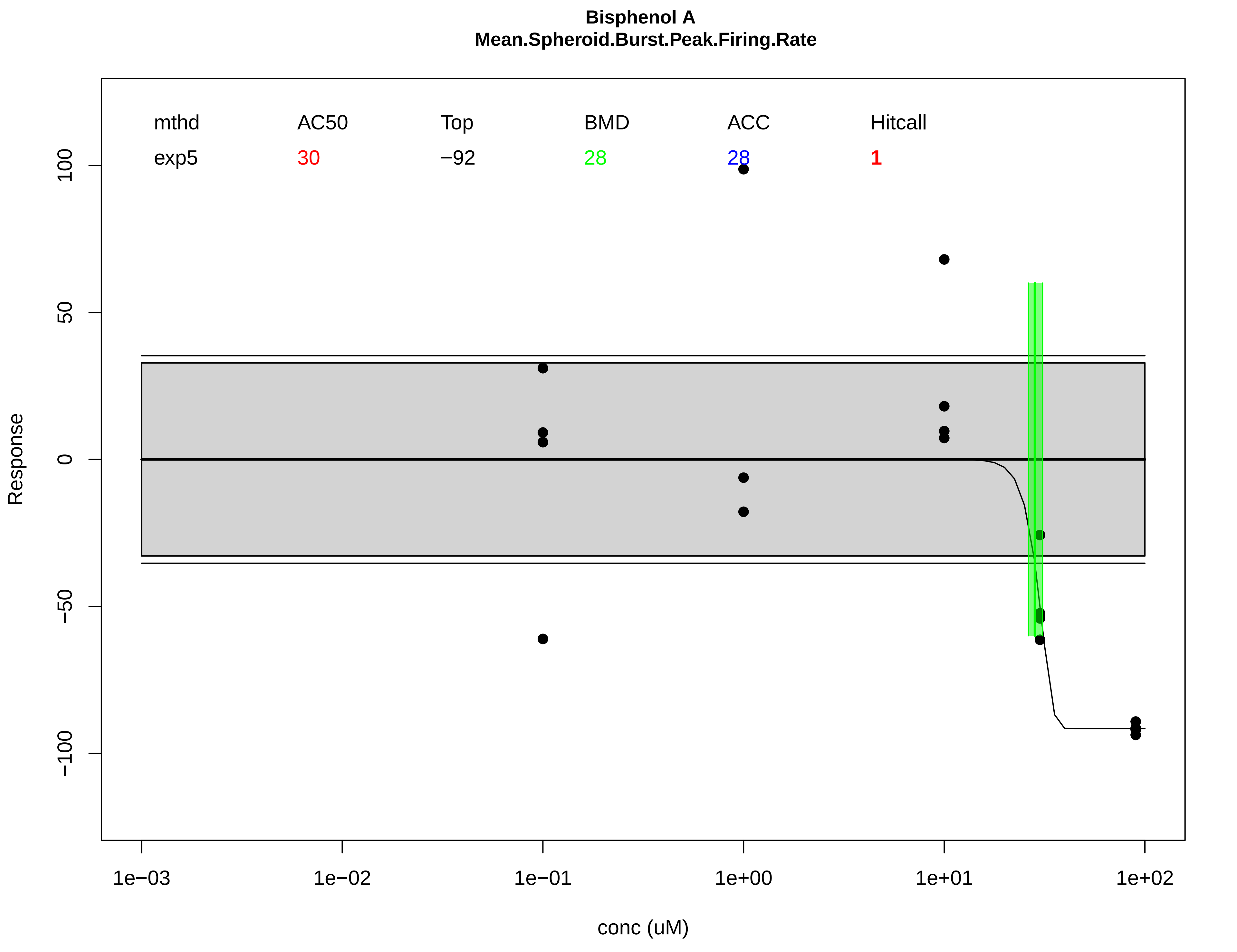
Bisphenol A
Mean.Spheroid.Burst.Peak.Firing.Rate
mthd
AC50
Top
BMD
ACC
Hitcall
exp5
30
−92
28
28
1
100
50
0
−50
−100
Response
1e−03
1e−02
1e−01
1e+00
1e+01
1e+02
conc (uM)

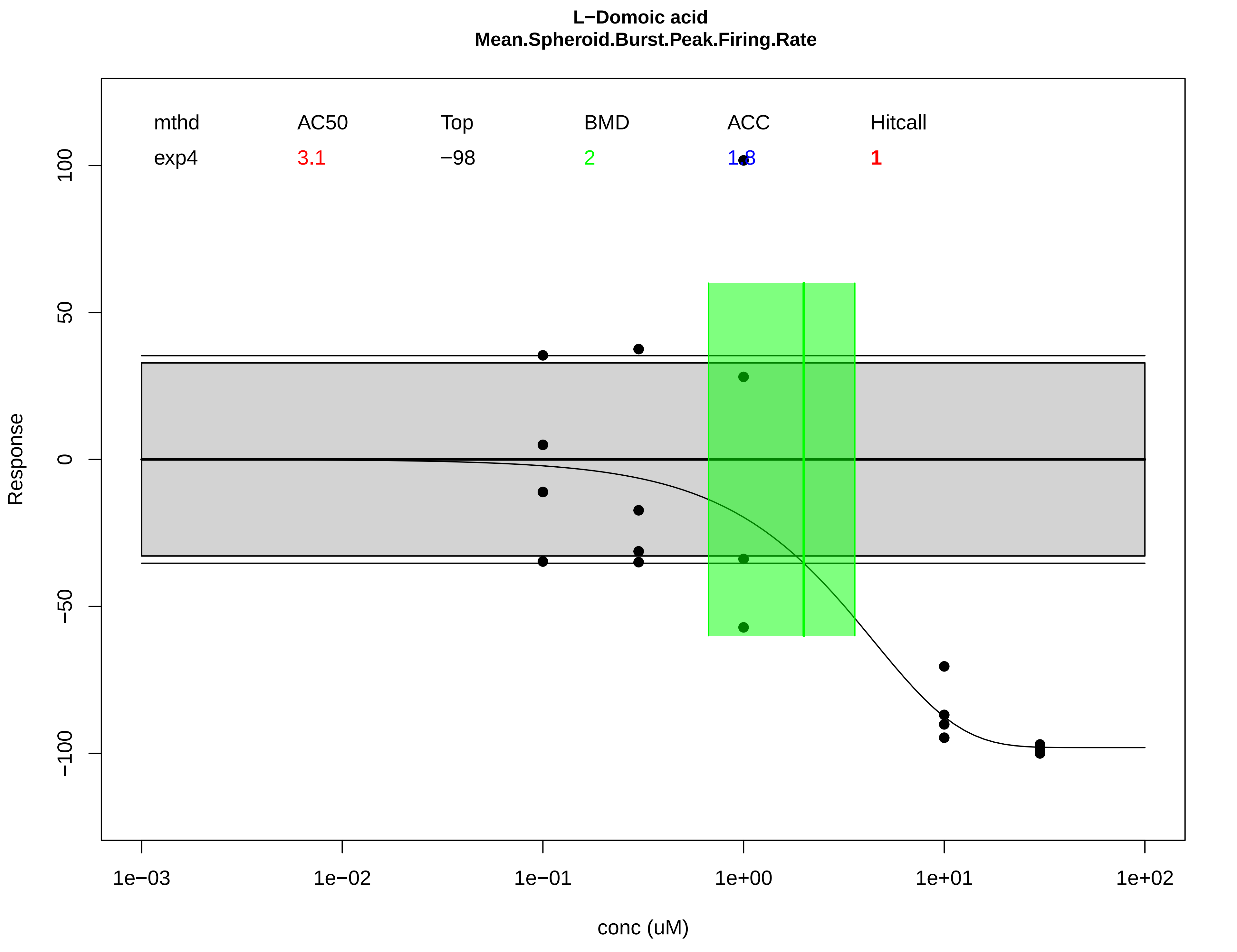
L−Domoic acid
Mean.Spheroid.Burst.Peak.Firing.Rate
mthd
AC50
Top
BMD
ACC
Hitcall
exp4
3.1
−98
2
1.8
1
Response
100
50
0
−50
−100
1e−03
1e−02
1e−01
1e+00
1e+01
1e+02
conc (uM)

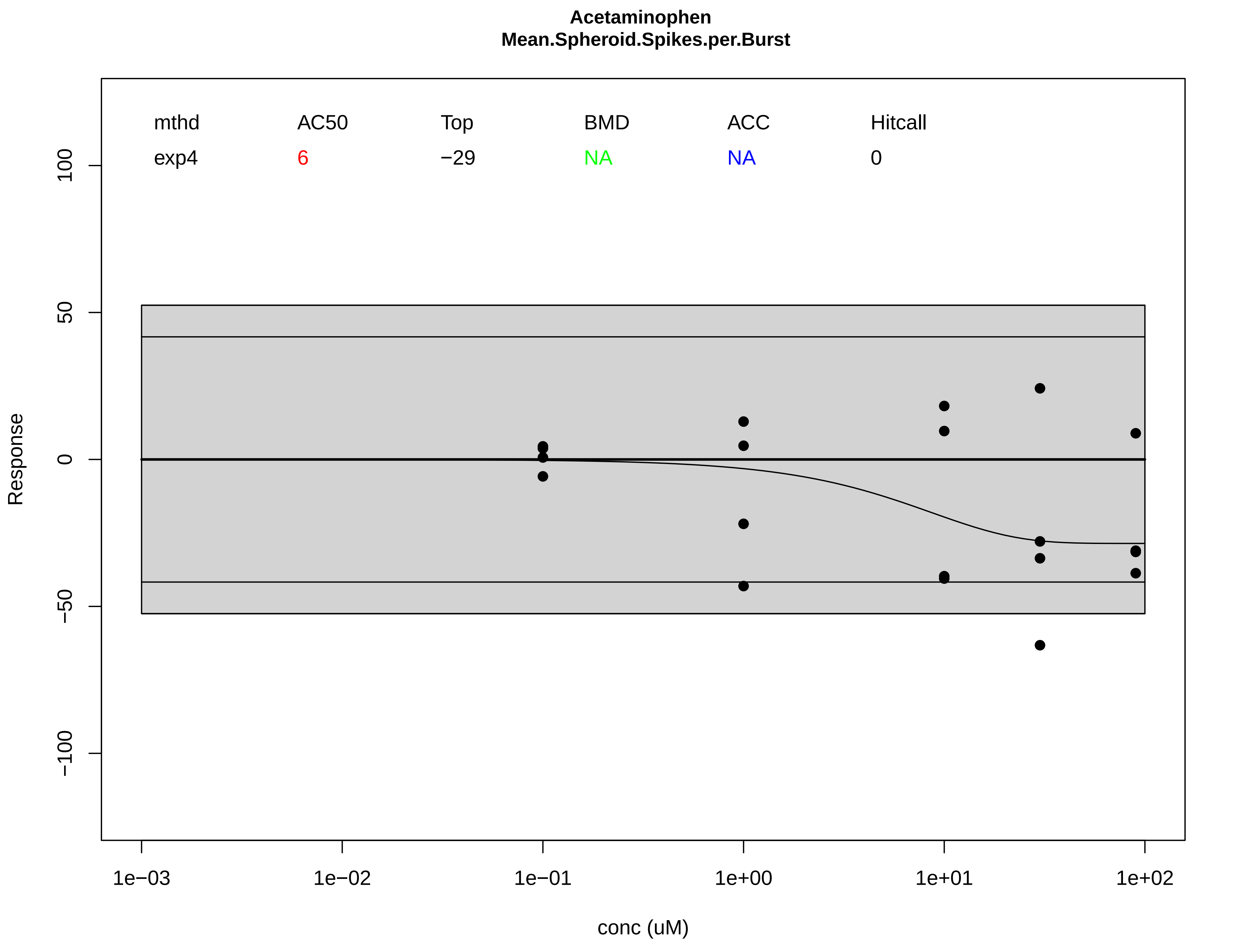

Acetaminophen
Mean.Spheroid.Spikes.per.Burst
mthd
AC50
Top
BMD
ACC
Hitcall
exp4
6
−29
NA
NA
0
100
50
0
−50
−100
Response
1e−03
1e−02
1e−01
1e+00
1e+01
1e+02
conc (uM)

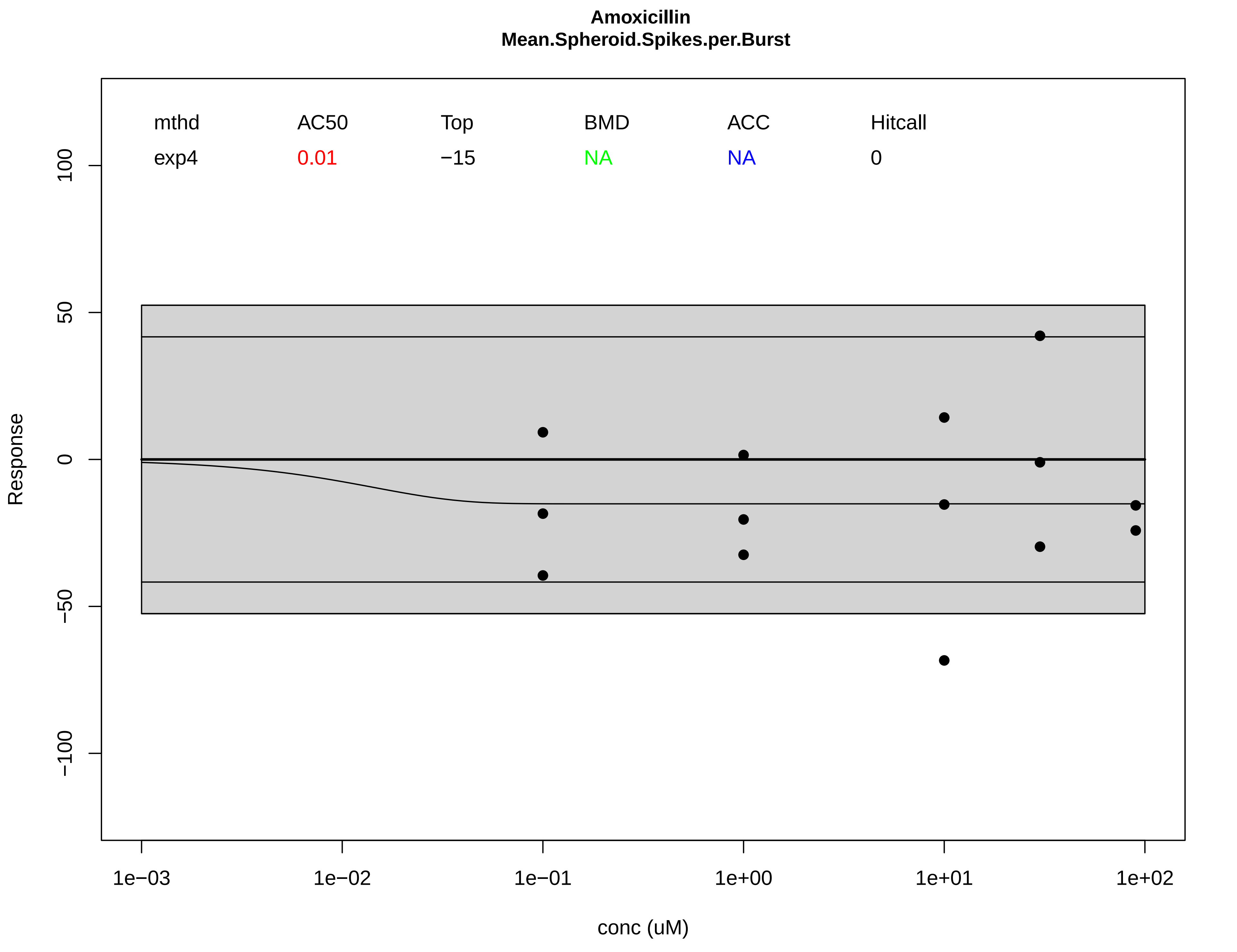
Amoxicillin
Mean.Spheroid.Spikes.per.Burst
mthd
AC50
Top
BMD
ACC
Hitcall
exp4
0.01
−15
NA
NA
0
100
50
0
−50
−100
Response
1e−03
1e−02
1e−01
1e+00
1e+01
1e+02
conc (uM)

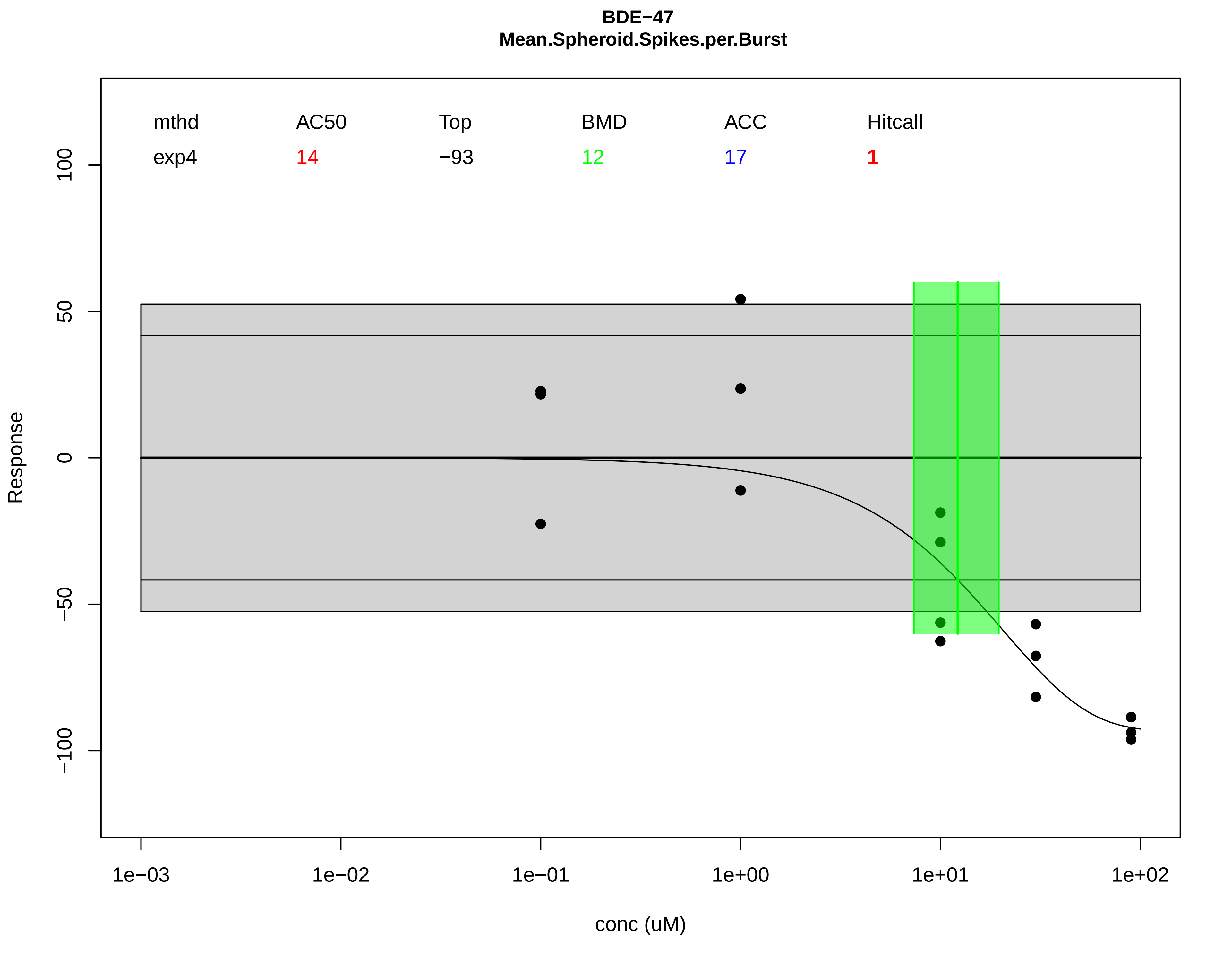
BDE−47
Mean.Spheroid.Spikes.per.Burst
mthd
AC50
Top
BMD
ACC
Hitcall
exp4
14
−93
12
17
1
Response
100
50
0
−50
−100
1e−03
1e−02
1e−01
1e+00
1e+01
1e+02
conc (uM)

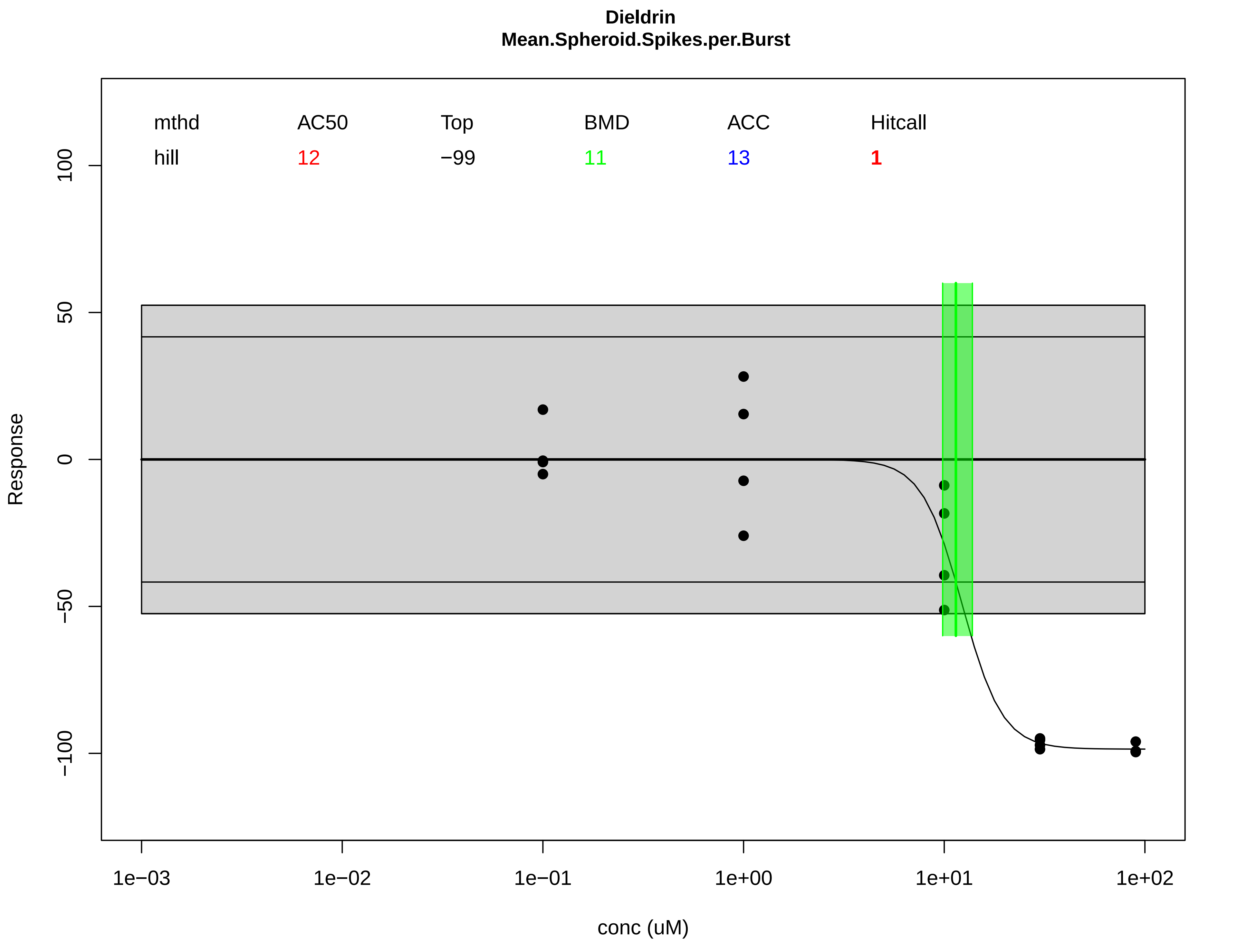

Dieldrin
Mean.Spheroid.Spikes.per.Burst
mthd
AC50
Top
BMD
ACC
Hitcall
hill
12
−99
11
13
1
100
50
0
−50
−100
Response
1e−03
1e−02
1e−01
1e+00
1e+01
1e+02
conc (uM)

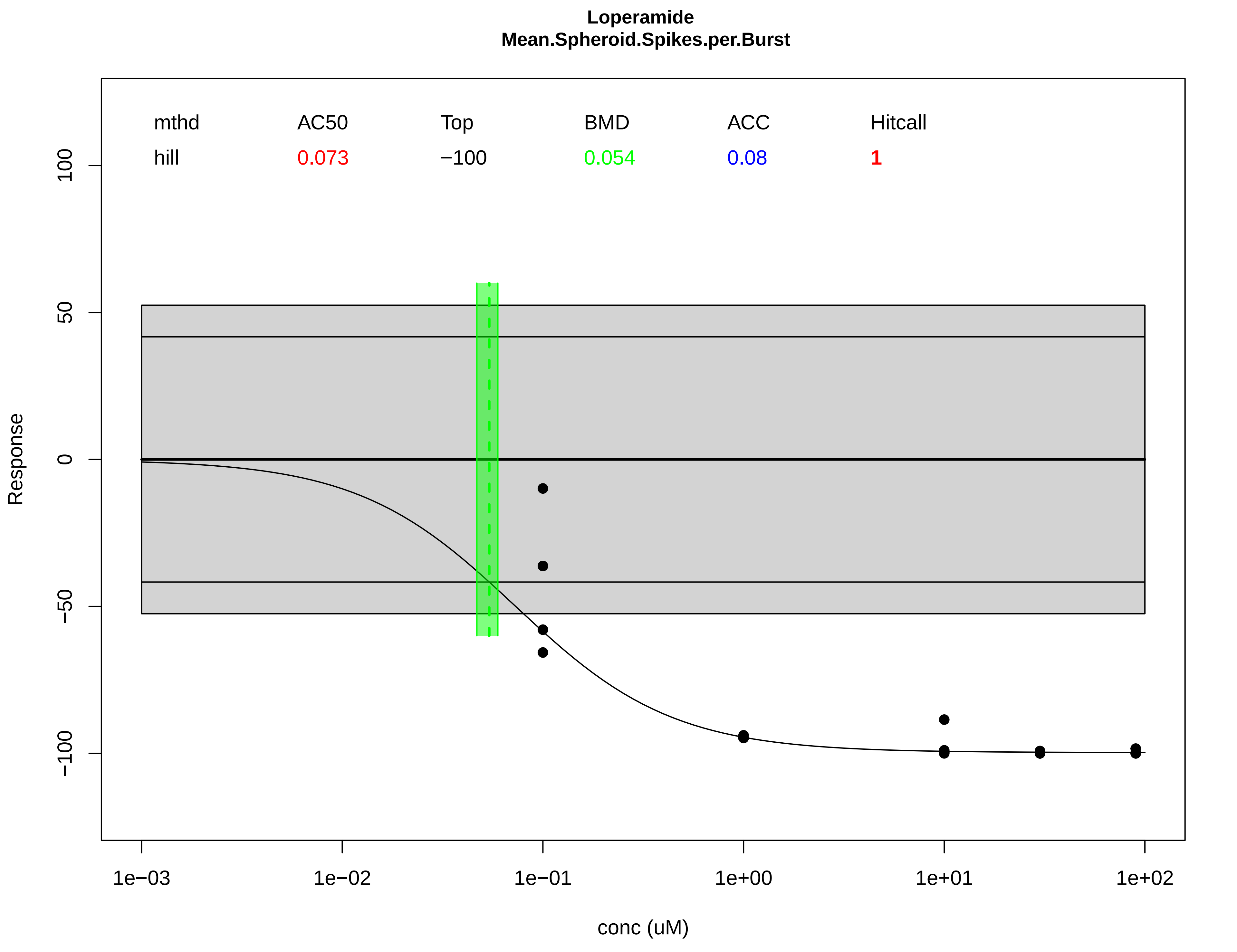

Loperamide
Mean.Spheroid.Spikes.per.Burst
mthd
AC50
Top
BMD
ACC
Hitcall
hill
0.073
−100
0.054
0.08
1
100
50
0
−50
−100
Response
1e−03
1e−02
1e−01
1e+00
1e+01
1e+02
conc (uM)

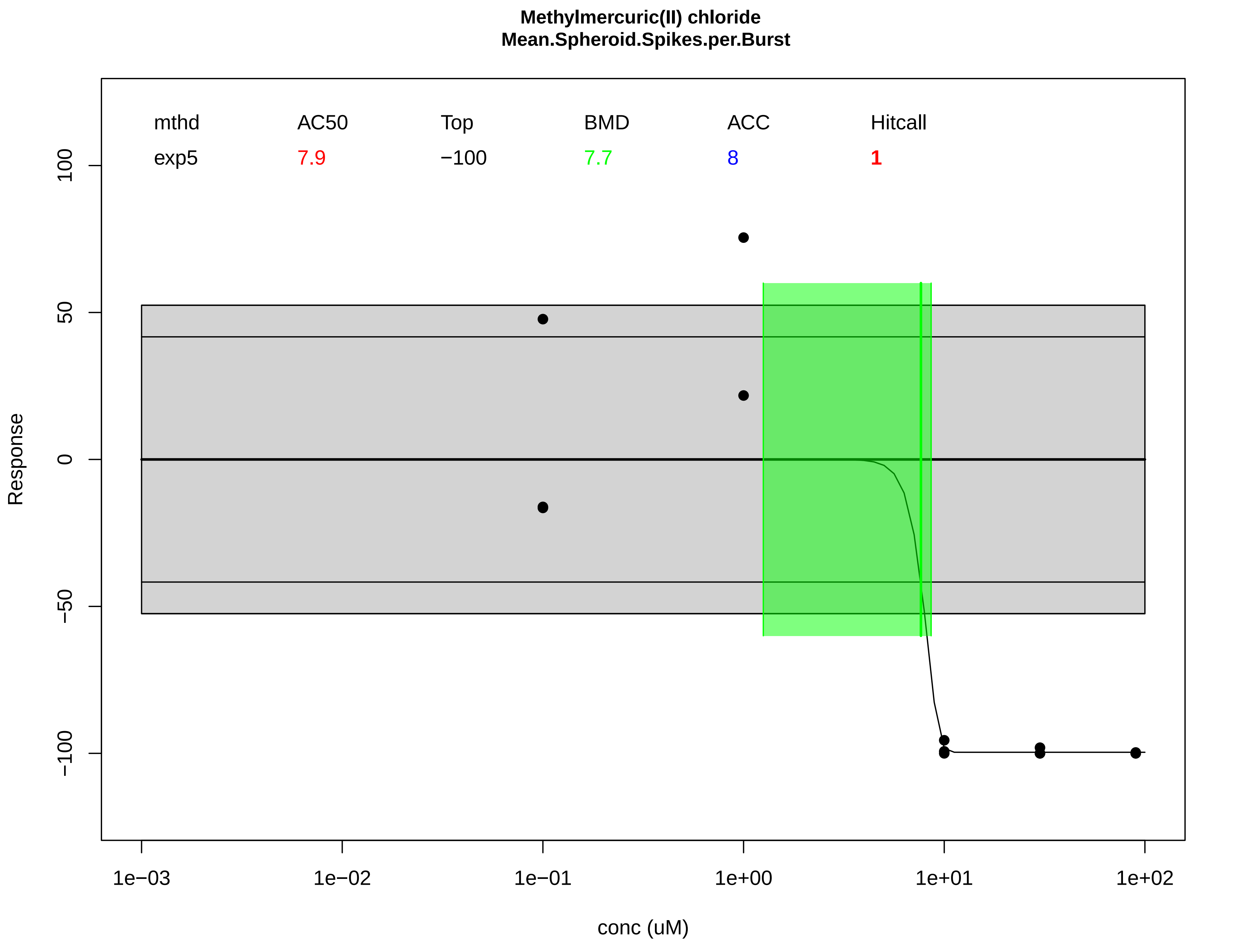
Methylmercuric(II) chloride
Mean.Spheroid.Spikes.per.Burst
mthd
AC50
Top
BMD
ACC
Hitcall
exp5
7.9
−100
7.7
8
1
Response
100
50
0
−50
−100
1e−03
1e−02
1e−01
1e+00
1e+01
1e+02
conc (uM)

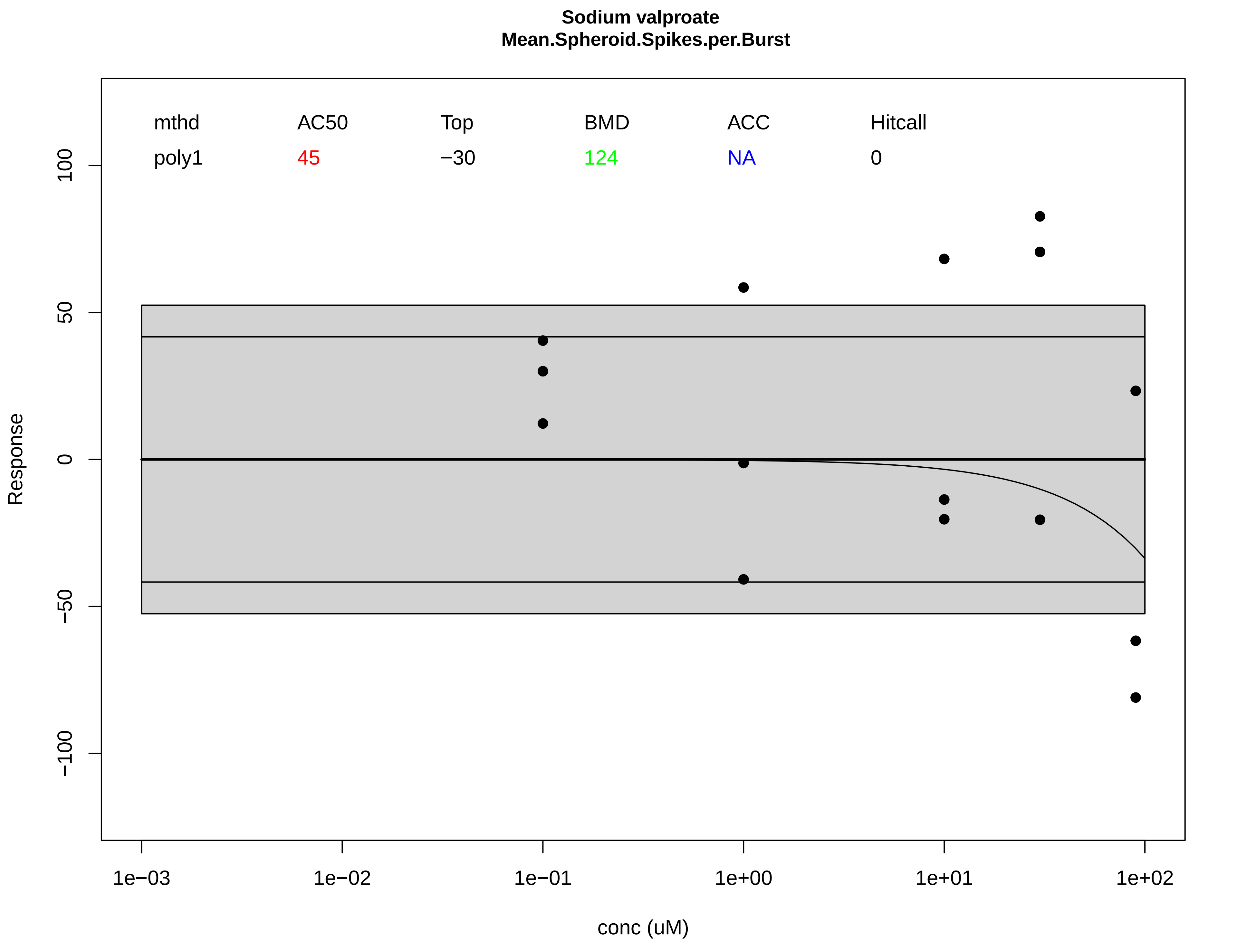
Sodium valproate
Mean.Spheroid.Spikes.per.Burst
mthd
AC50
Top
BMD
ACC
Hitcall
poly1
45
−30
124
NA
0
100
50
0
−50
−100
Response
1e−03
1e−02
1e−01
1e+00
1e+01
1e+02
conc (uM)

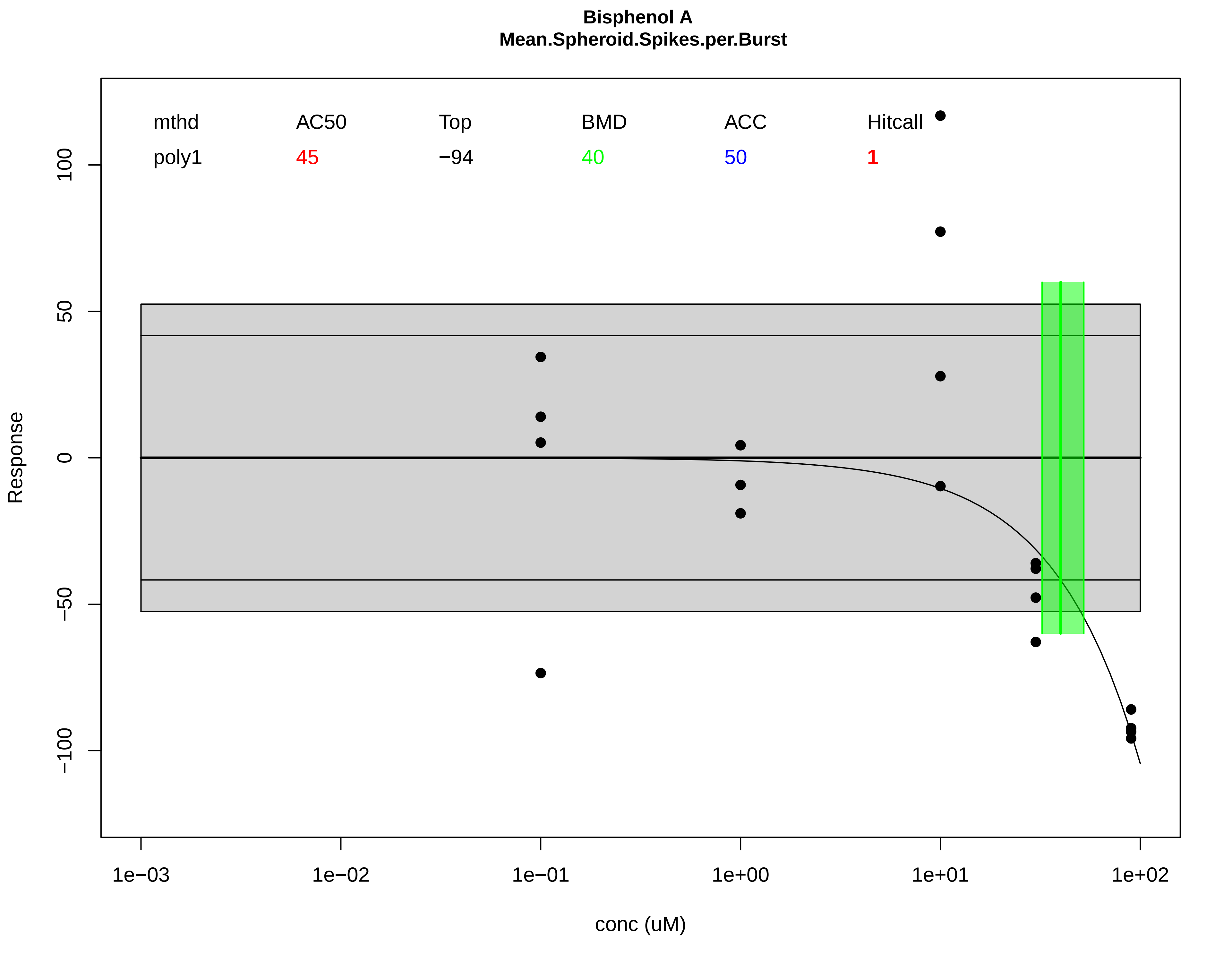
Bisphenol A
Mean.Spheroid.Spikes.per.Burst
mthd
AC50
Top
BMD
ACC
Hitcall
poly1
45
−94
40
50
1
Response
100
50
0
−50
−100
1e−03
1e−02
1e−01
1e+00
1e+01
1e+02
conc (uM)

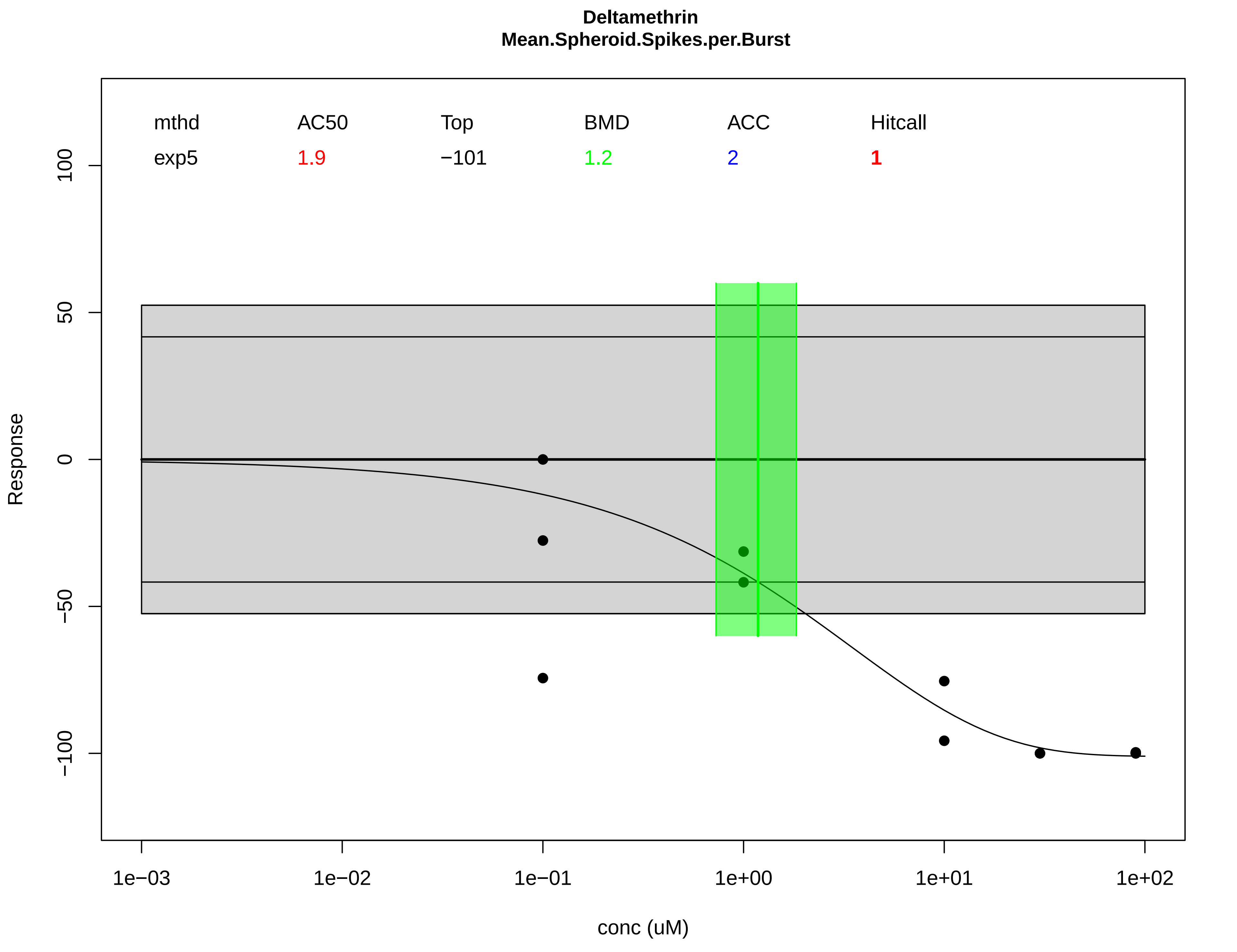
Deltamethrin
Mean.Spheroid.Spikes.per.Burst
mthd
AC50
Top
BMD
ACC
Hitcall
exp5
1.9
−101
1.2
2
1
100
50
0
−50
−100
Response
1e−03
1e−02
1e−01
1e+00
1e+01
1e+02
conc (uM)

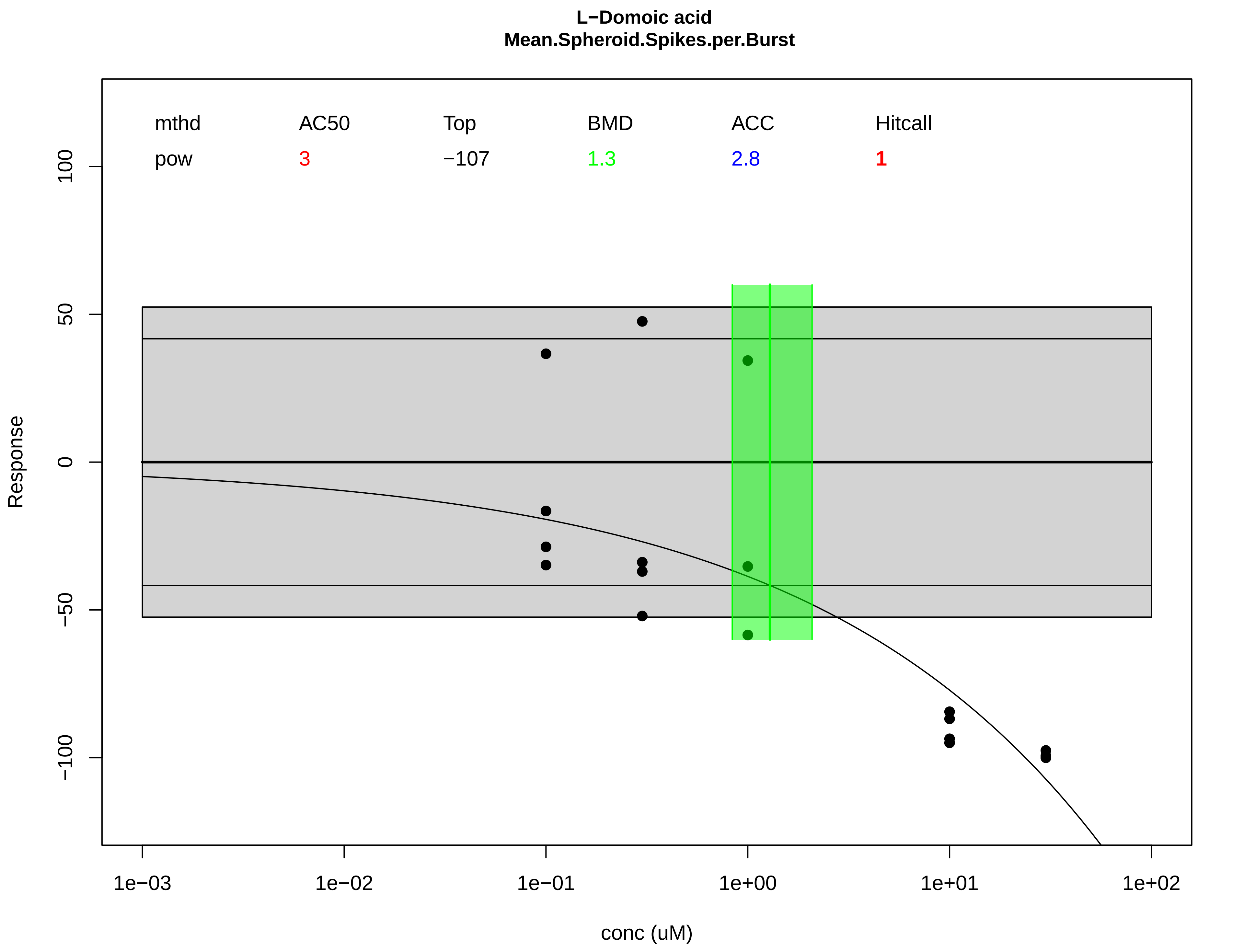

L−Domoic acid
Mean.Spheroid.Spikes.per.Burst
mthd
AC50
Top
BMD
ACC
Hitcall
pow
3
−107
1.3
2.8
1
100
50
0
−50
−100
Response
1e−03
1e−02
1e−01
1e+00
1e+01
1e+02
conc (uM)

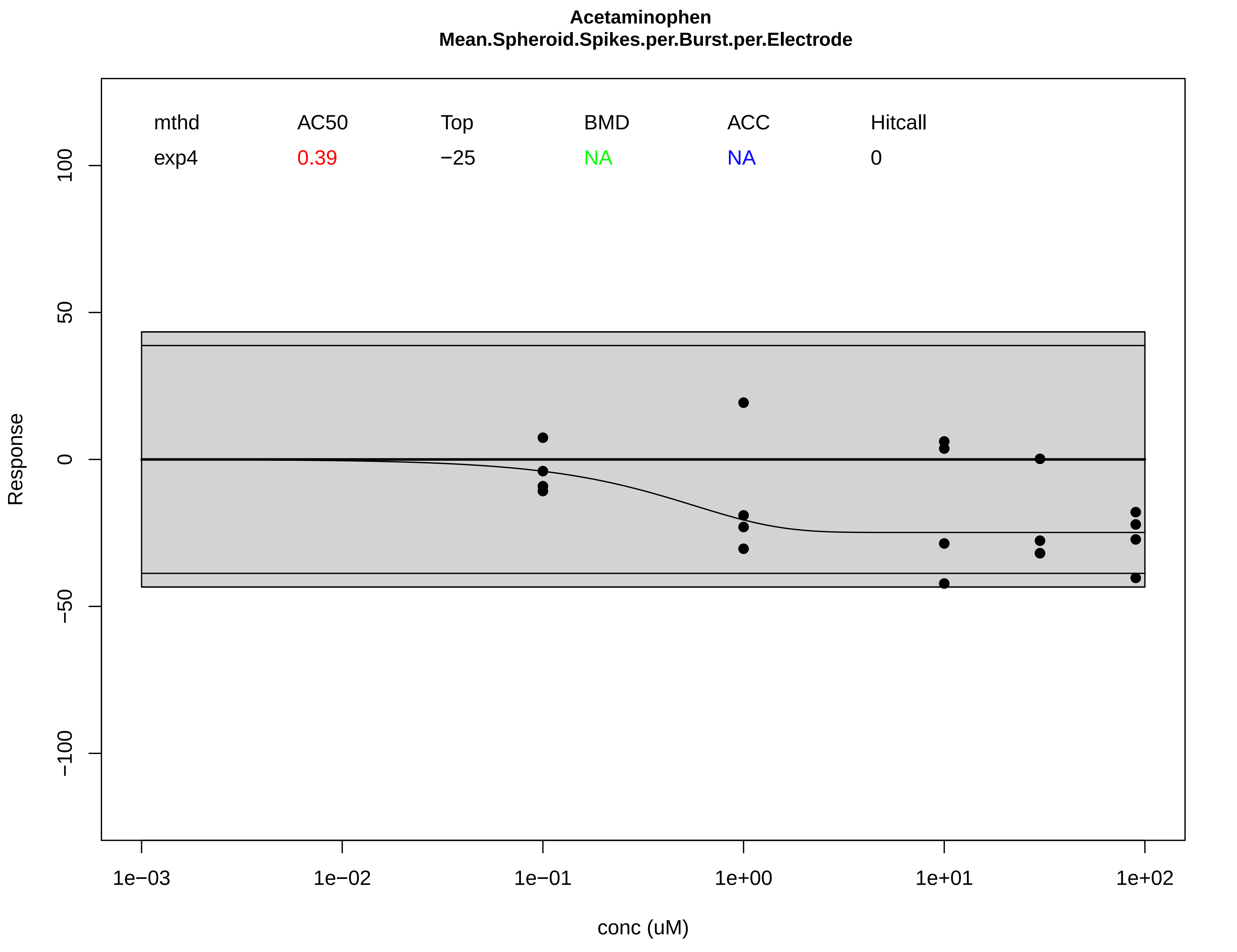
Acetaminophen
Mean.Spheroid.Spikes.per.Burst.per.Electrode
mthd
AC50
Top
BMD
ACC
Hitcall
exp4
0.39
−25
NA
NA
0
100
50
0
−50
−100
Response
1e−03
1e−02
1e−01
1e+00
1e+01
1e+02
conc (uM)

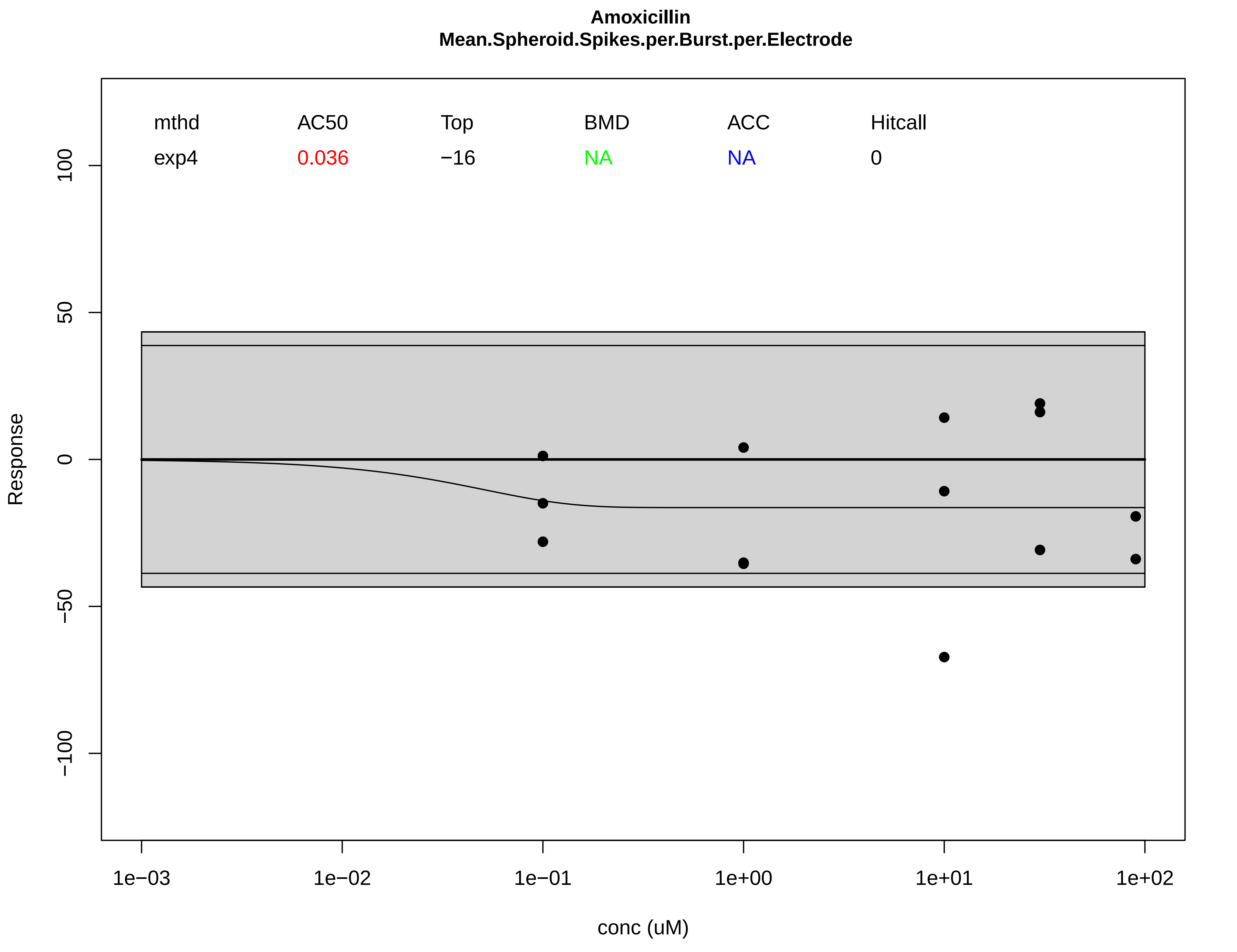

Amoxicillin
Mean.Spheroid.Spikes.per.Burst.per.Electrode
mthd
AC50
Top
BMD
ACC
Hitcall
exp4
0.036
−16
NA
NA
0
100
50
0
−50
−100
Response
1e−03
1e−02
1e−01
1e+00
1e+01
1e+02
conc (uM)

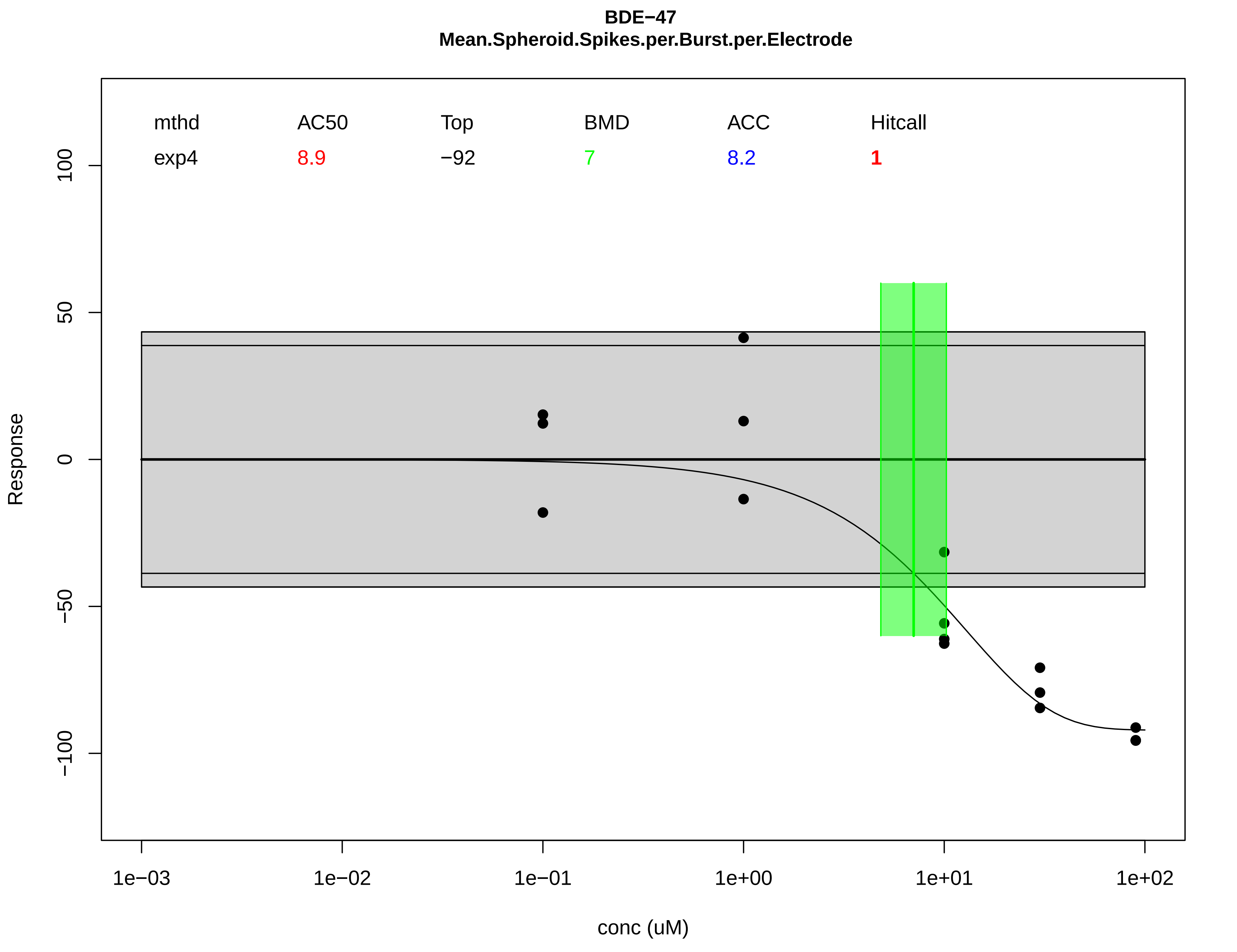

BDE−47
Mean.Spheroid.Spikes.per.Burst.per.Electrode
mthd
AC50
Top
BMD
ACC
Hitcall
exp4
8.9
−92
7
8.2
1
100
50
0
−50
−100
Response
1e−03
1e−02
1e−01
1e+00
1e+01
1e+02
conc (uM)

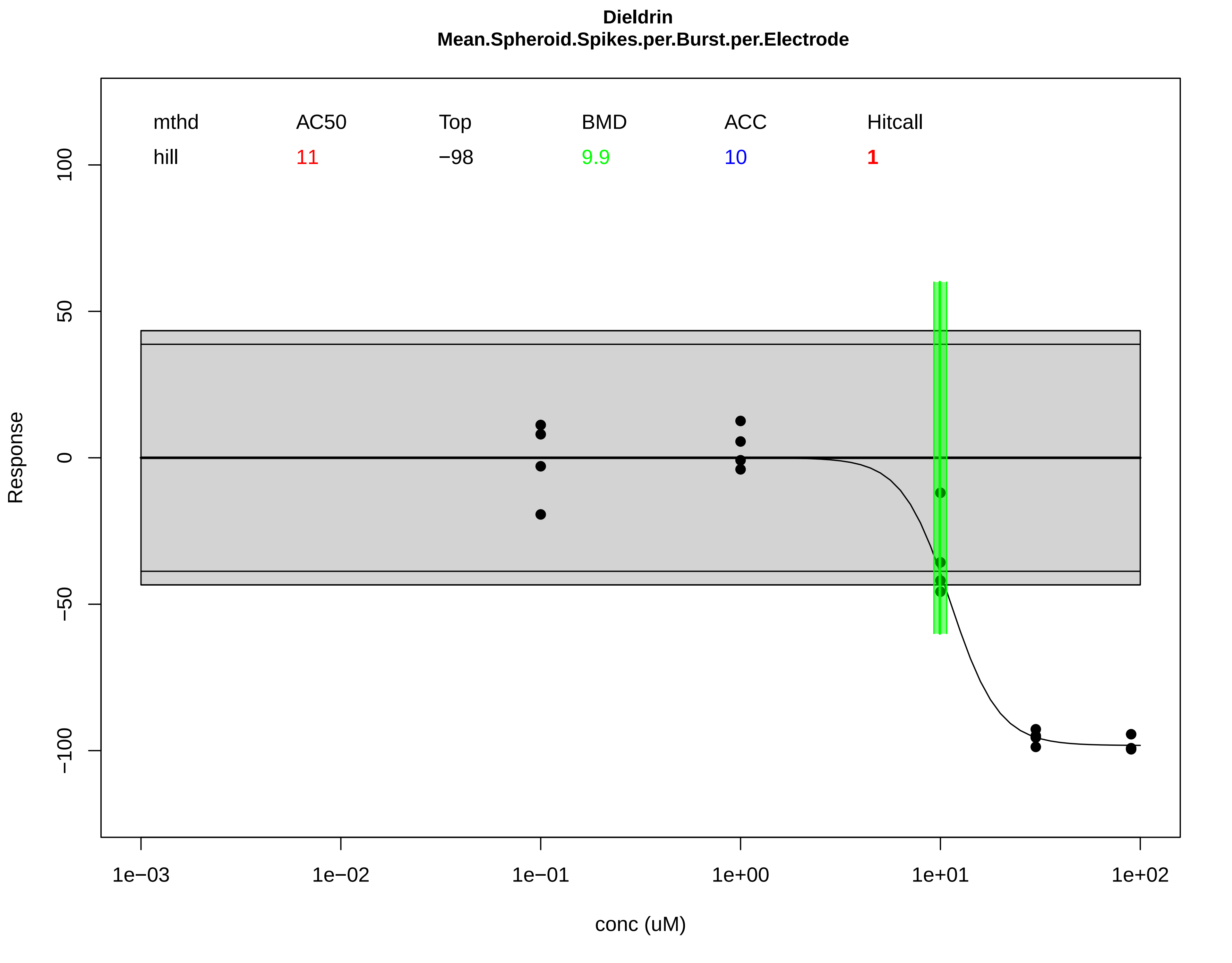

Dieldrin
Mean.Spheroid.Spikes.per.Burst.per.Electrode
mthd
AC50
Top
BMD
ACC
Hitcall
hill
11
−98
9.9
10
1
Response
100
50
0
−50
−100
1e−03
1e−02
1e−01
1e+00
1e+01
1e+02
conc (uM)

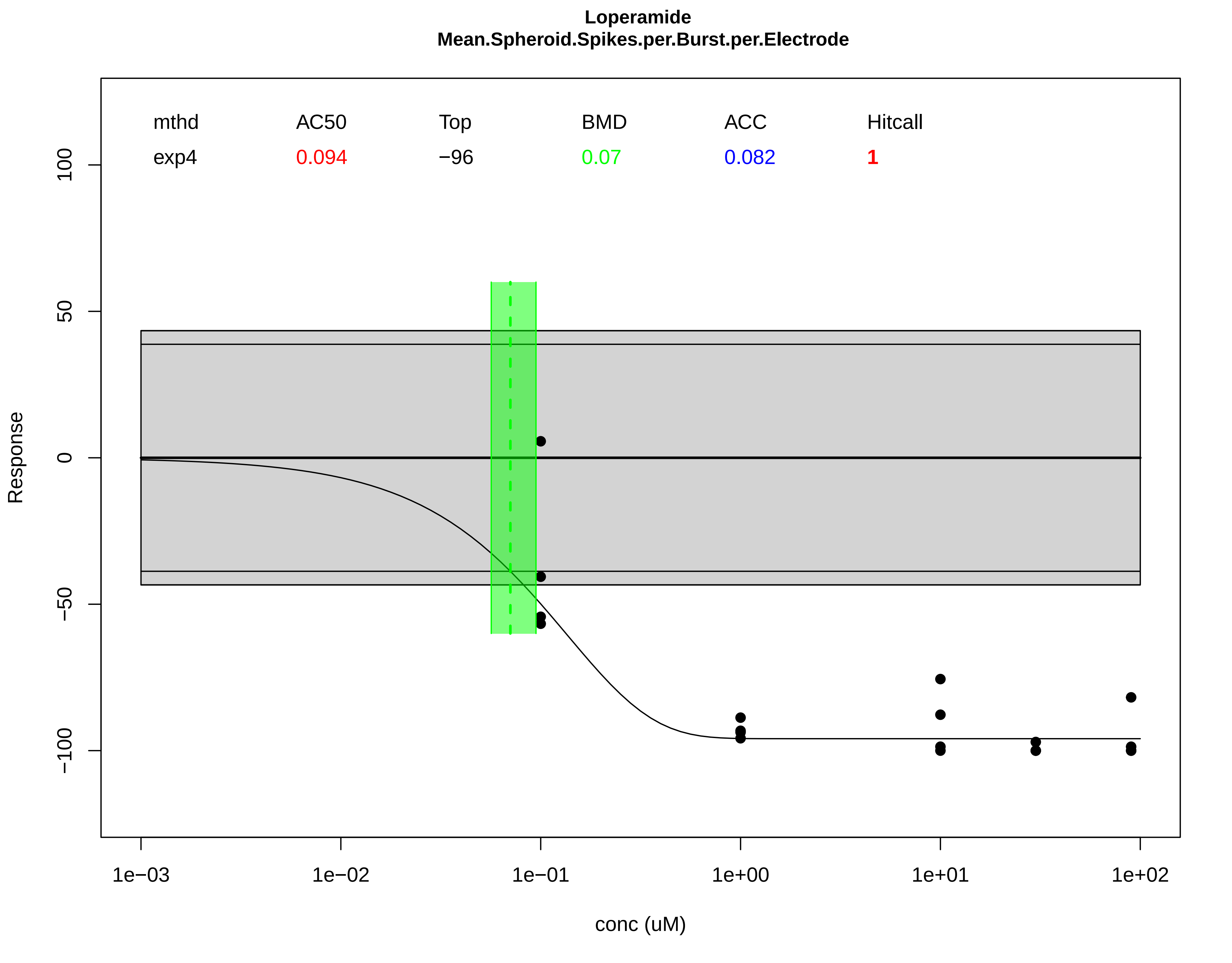
Loperamide
Mean.Spheroid.Spikes.per.Burst.per.Electrode
mthd
AC50
Top
BMD
ACC
Hitcall
exp4
0.094
−96
0.07
0.082
1
100
50
0
−50
−100
Response
1e−03
1e−02
1e−01
1e+00
1e+01
1e+02
conc (uM)

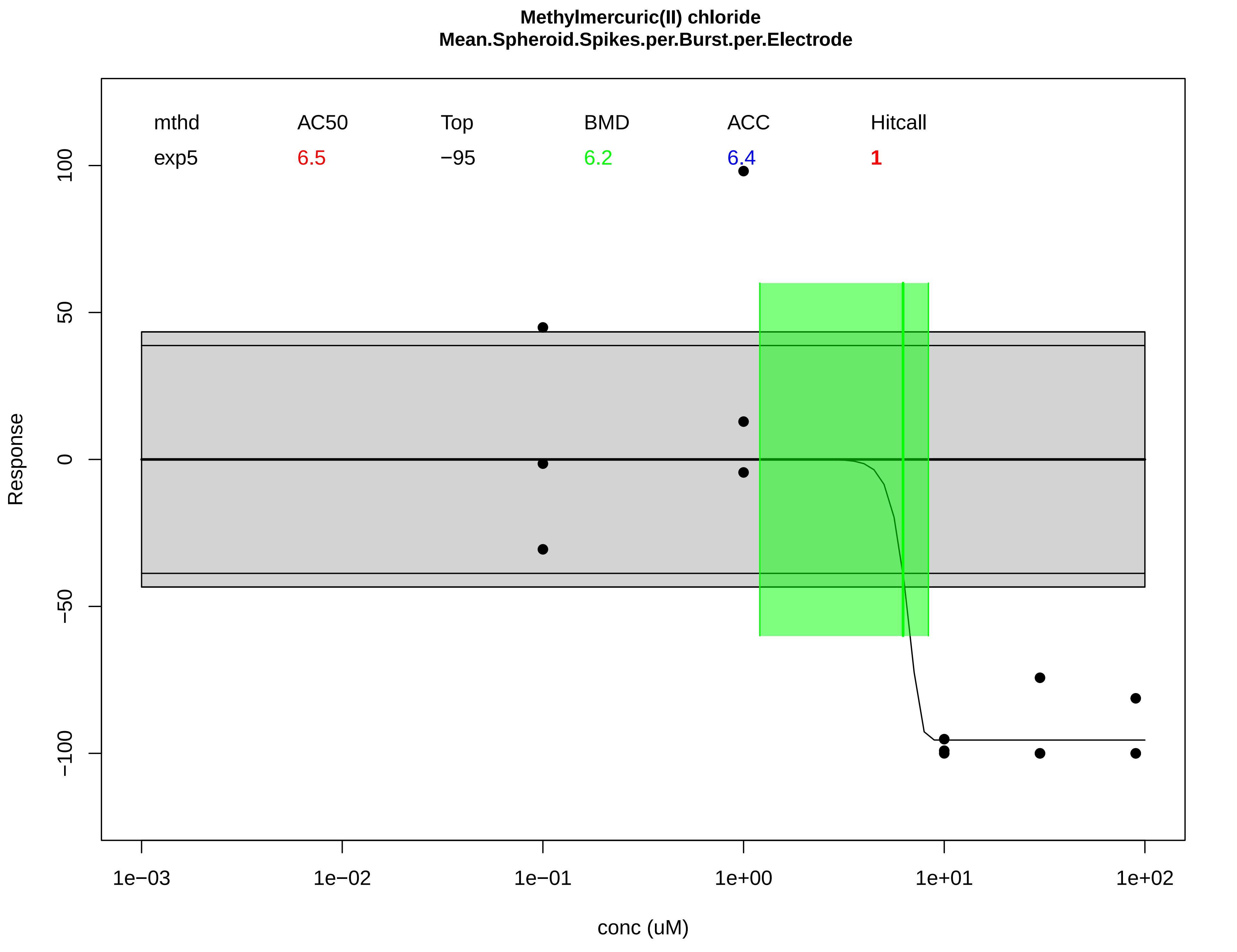
Methylmercuric(II) chloride
Mean.Spheroid.Spikes.per.Burst.per.Electrode
mthd
AC50
Top
BMD
ACC
Hitcall
exp5
6.5
−95
6.2
6.4
1
Response
100
50
0
−50
−100
1e−03
1e−02
1e−01
1e+00
1e+01
1e+02
conc (uM)

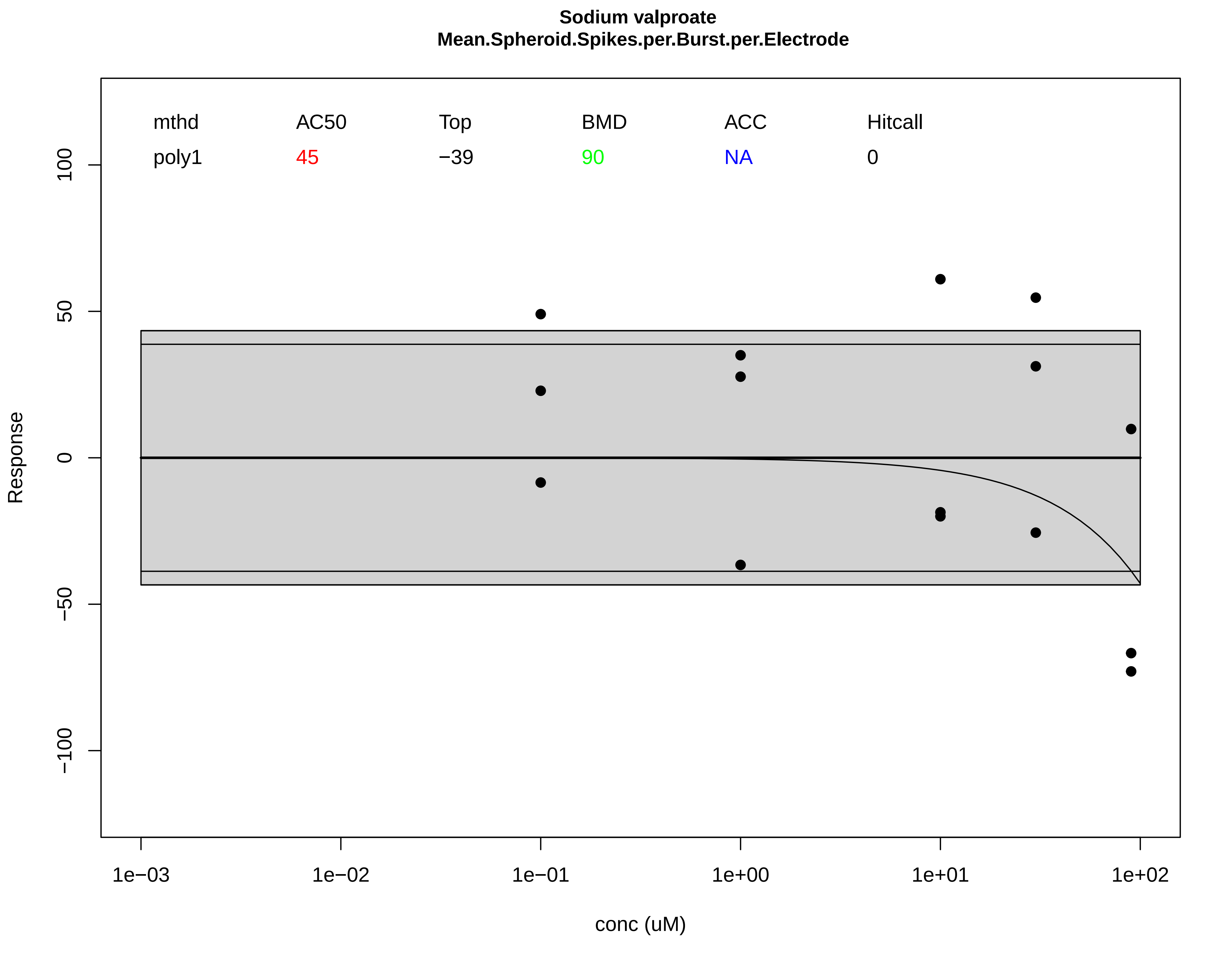
Sodium valproate
Mean.Spheroid.Spikes.per.Burst.per.Electrode
mthd
AC50
Top
BMD
ACC
Hitcall
poly1
45
−39
90
NA
0
100
50
0
−50
−100
Response
1e−03
1e−02
1e−01
1e+00
1e+01
1e+02
conc (uM)

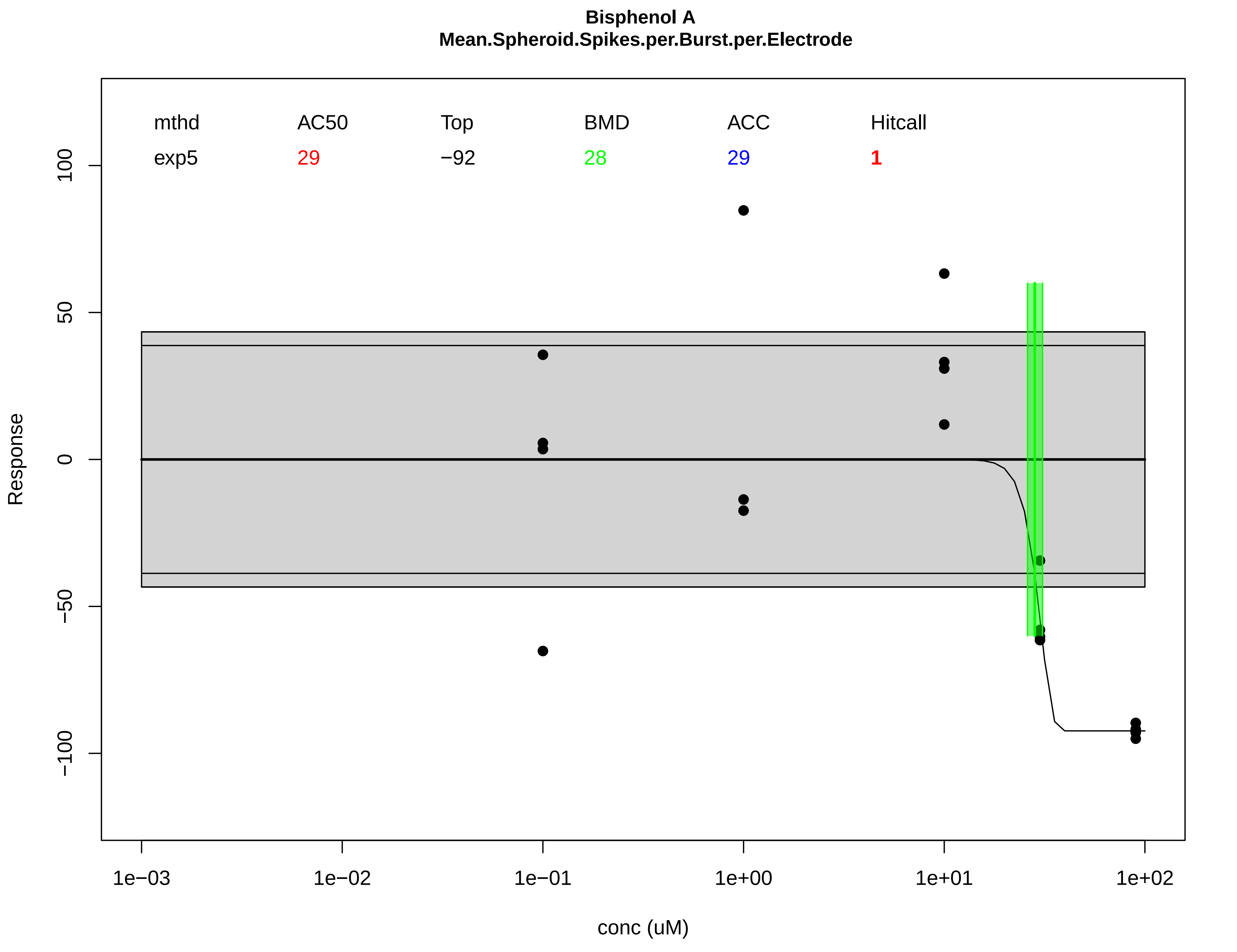
Bisphenol A
Mean.Spheroid.Spikes.per.Burst.per.Electrode
mthd
exp5
AC50
29
Top
−92
BMD
28
ACC
29
Hitcall
1
Response
100
50
0
−50
−100
1e−03
1e−02
1e−01
1e+00
1e+01
1e+02
conc (uM)

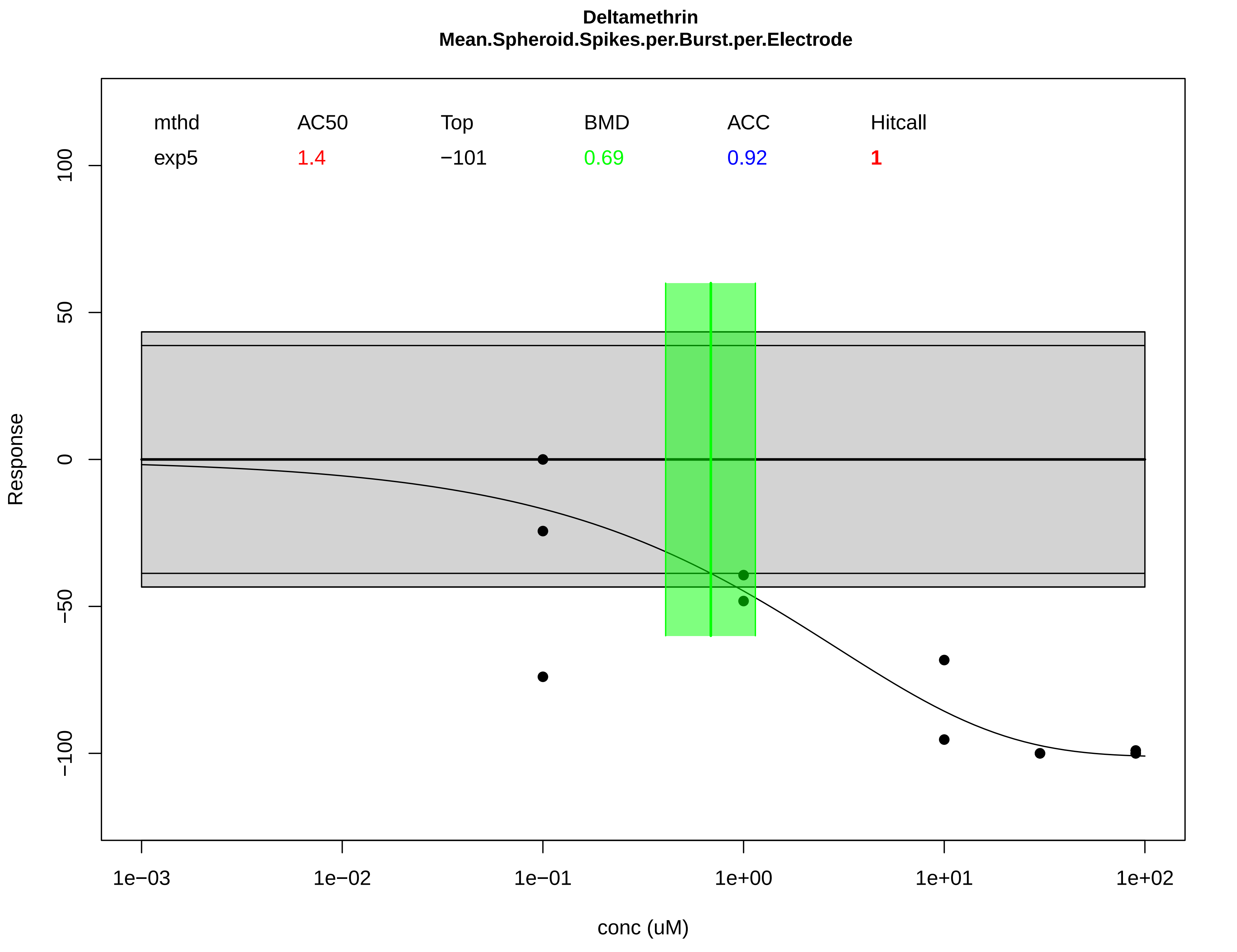
Deltamethrin
Mean.Spheroid.Spikes.per.Burst.per.Electrode
mthd
AC50
Top
BMD
ACC
Hitcall
exp5
1.4
−101
0.69
0.92
1
Response
100
50
0
−50
−100
1e−03
1e−02
1e−01
1e+00
1e+01
1e+02
conc (uM)

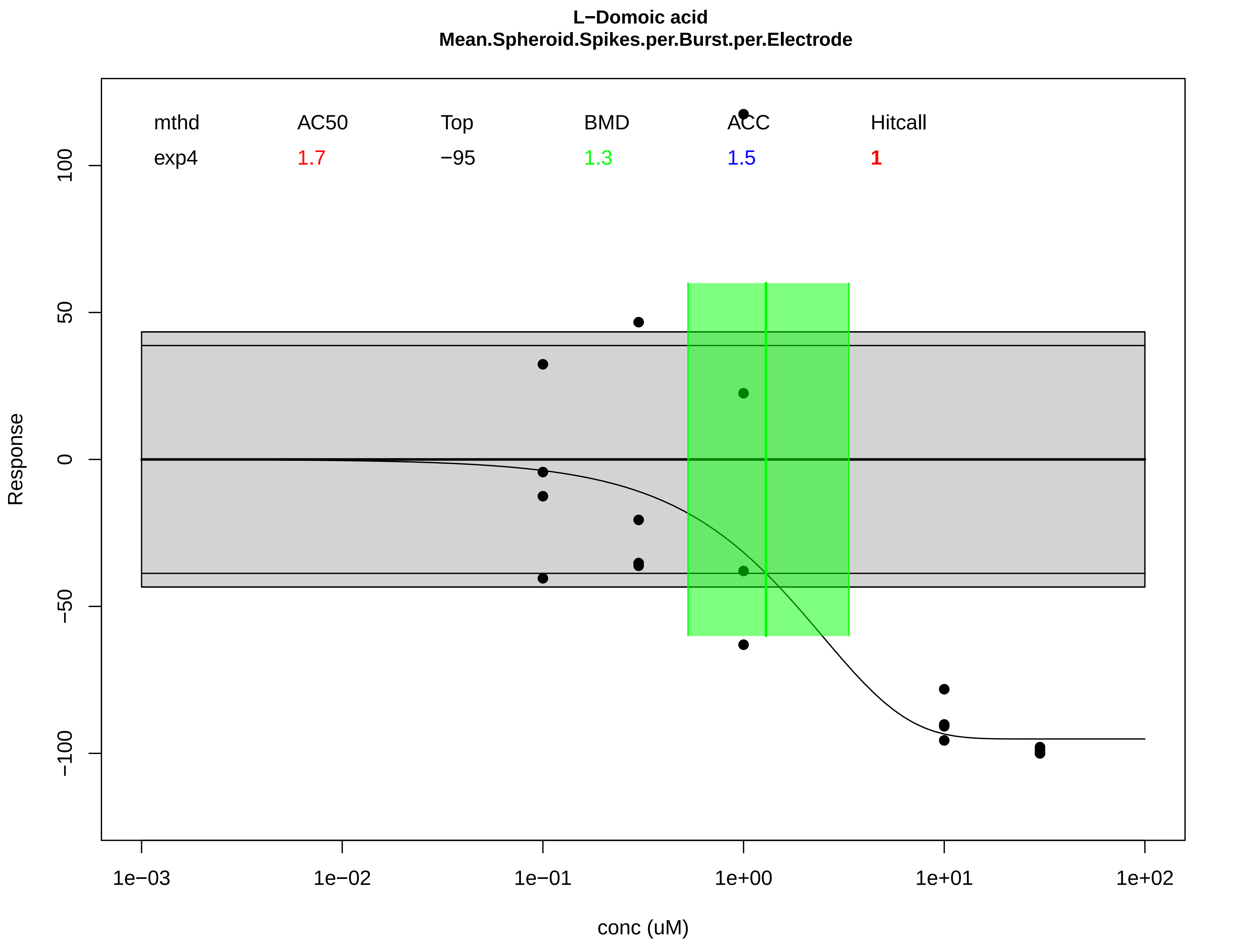
L−Domoic acid
Mean.Spheroid.Spikes.per.Burst.per.Electrode
mthd
AC50
Top
BMD
ACC
Hitcall
exp4
1.7
−95
1.3
1.5
1
Response
100
50
0
−50
−100
1e−03
1e−02
1e−01
1e+00
1e+01
1e+02
conc (uM)

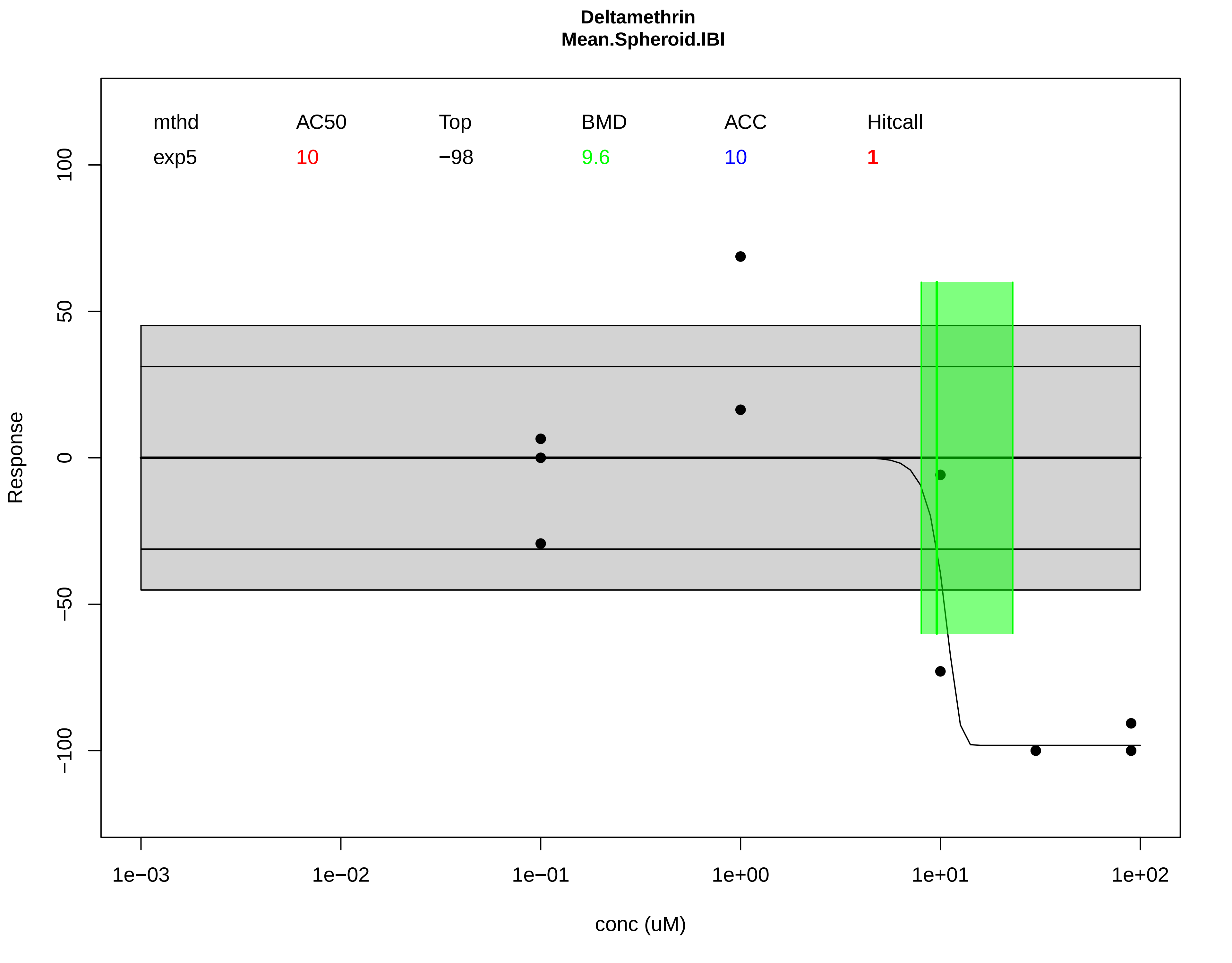

Deltamethrin
Mean.Spheroid.IBI
mthd
AC50
Top
BMD
ACC
Hitcall
exp5
10
−98
9.6
10
1
100
50
0
−50
−100
Response
1e−03
1e−02
1e−01
1e+00
1e+01
1e+02
conc (uM)

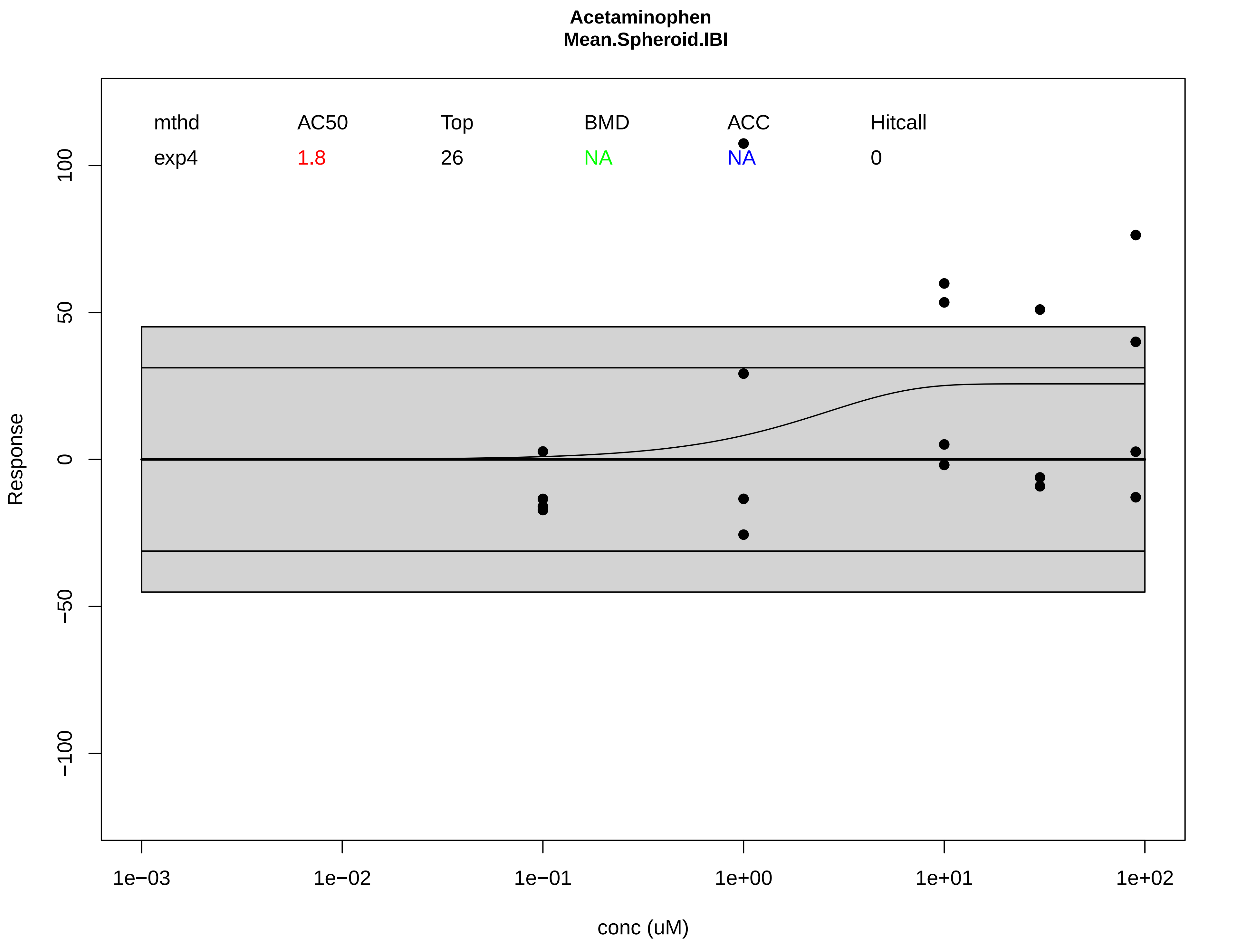

Acetaminophen
Mean.Spheroid.IBI
mthd
AC50
Top
BMD
ACC
Hitcall
exp4
1.8
26
NA
NA
0
Response
100
50
0
−50
−100
1e−03
1e−02
1e−01
1e+00
1e+01
1e+02
conc (uM)

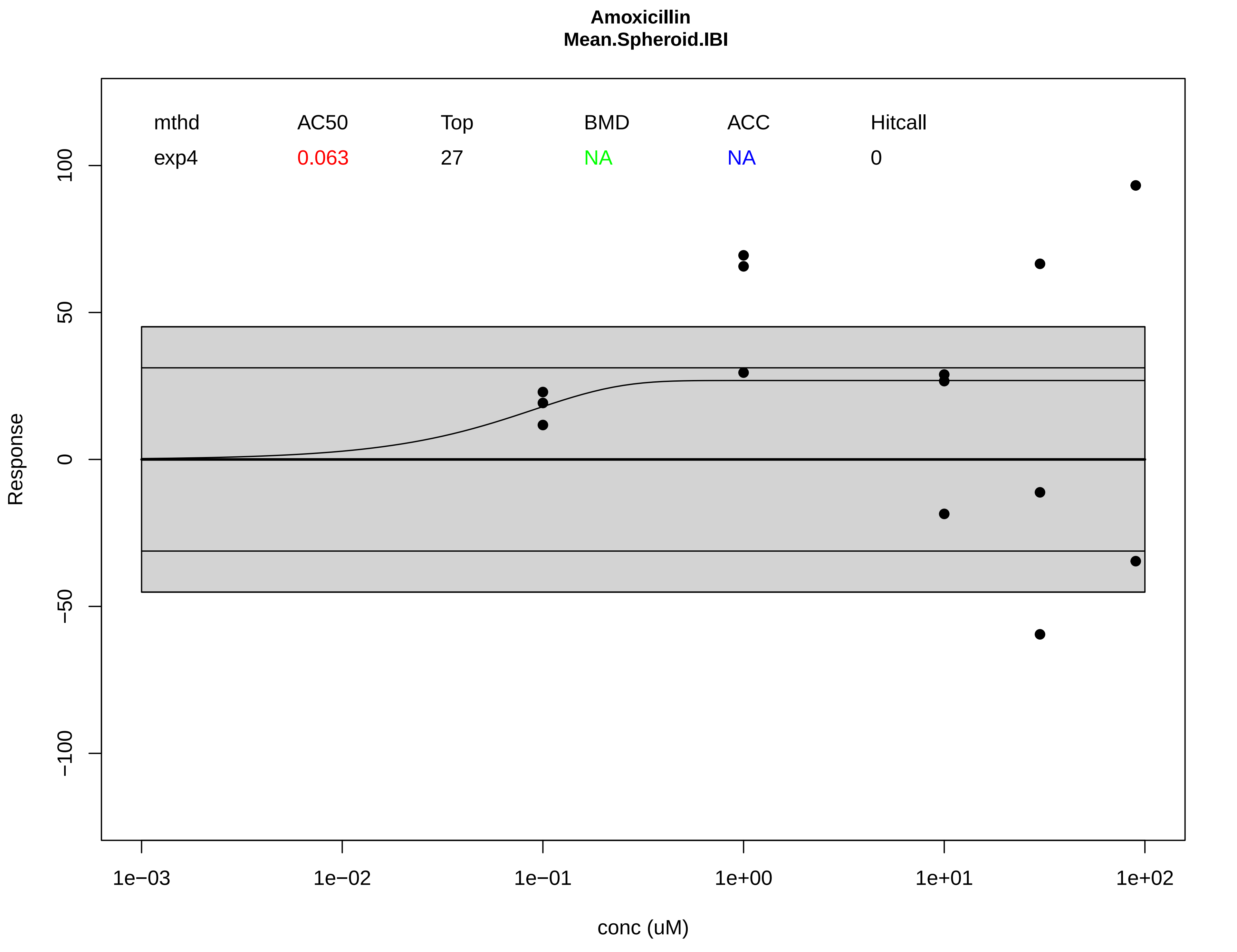
Amoxicillin
Mean.Spheroid.IBI
mthd
AC50
Top
BMD
ACC
Hitcall
exp4
0.063
27
NA
NA
0
100
50
0
−50
−100
Response
1e−03
1e−02
1e−01
1e+00
1e+01
1e+02
conc (uM)

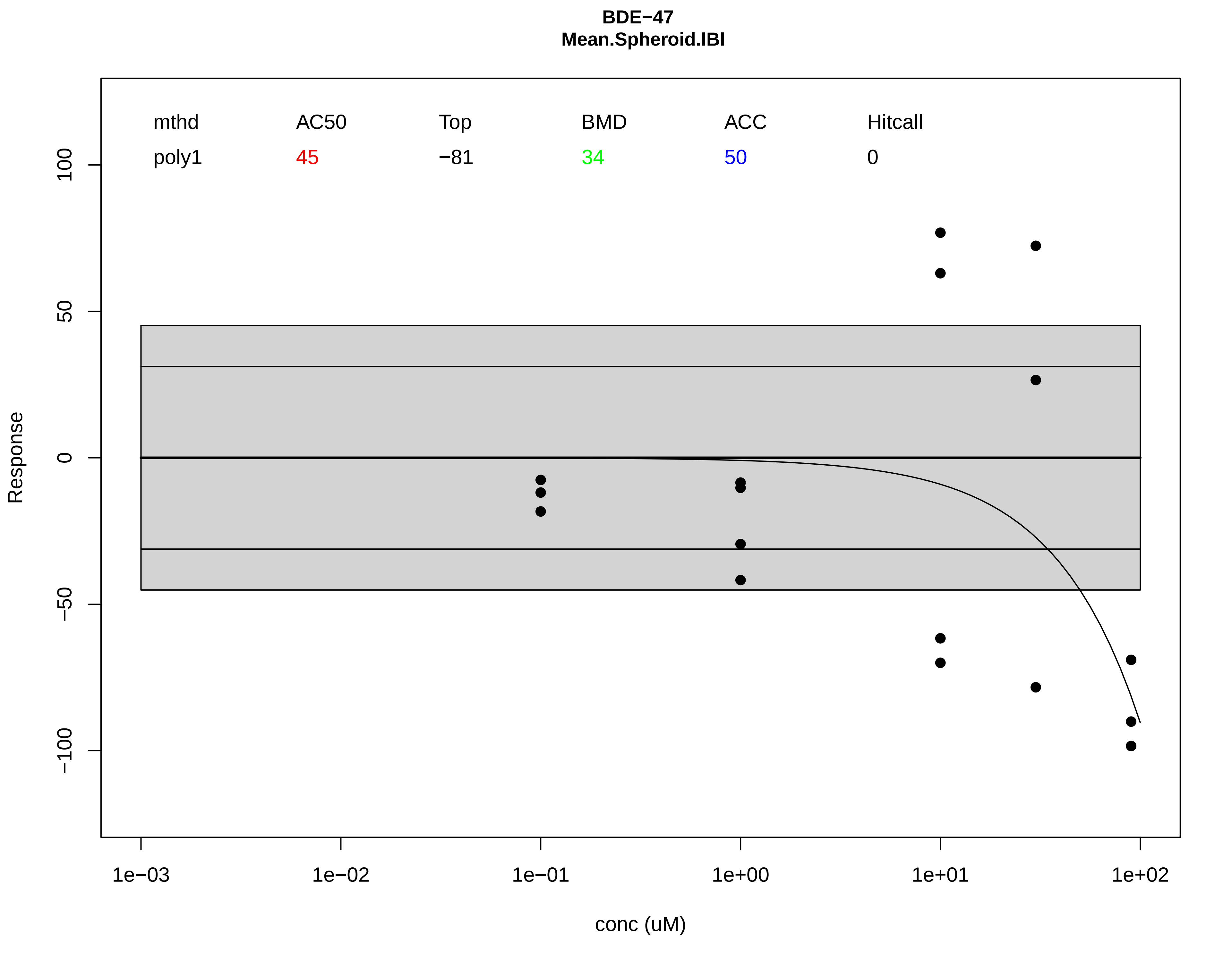
BDE−47
Mean.Spheroid.IBI
mthd
AC50
Top
BMD
ACC
Hitcall
poly1
45
−81
34
50
0
Response
100
50
0
−50
−100
1e−03
1e−02
1e−01
1e+00
1e+01
1e+02
conc (uM)

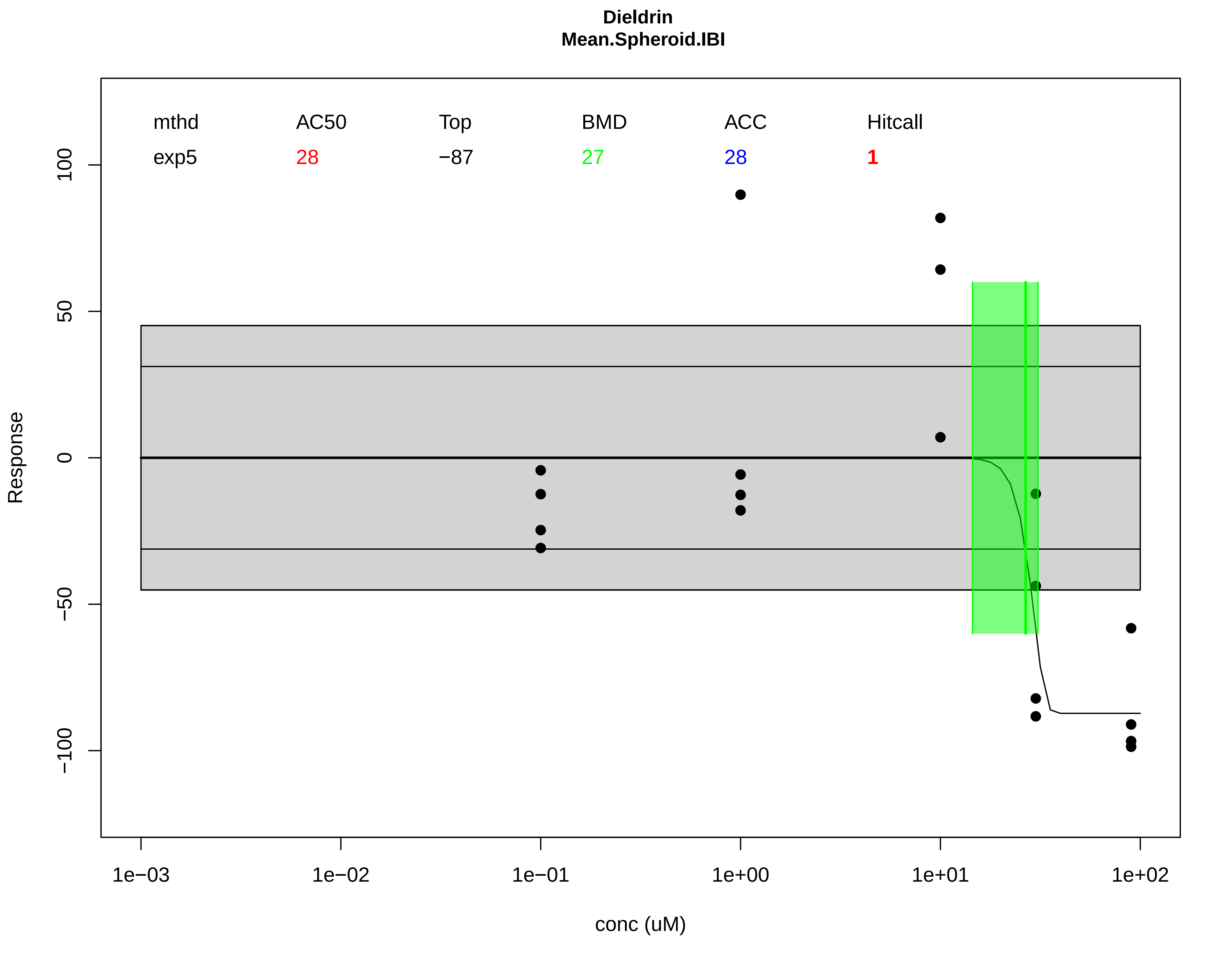
Dieldrin
Mean.Spheroid.IBI
mthd
AC50
Top
BMD
ACC
Hitcall
exp5
28
−87
27
28
1
100
50
0
−50
−100
Response
1e−03
1e−02
1e−01
1e+00
1e+01
1e+02
conc (uM)

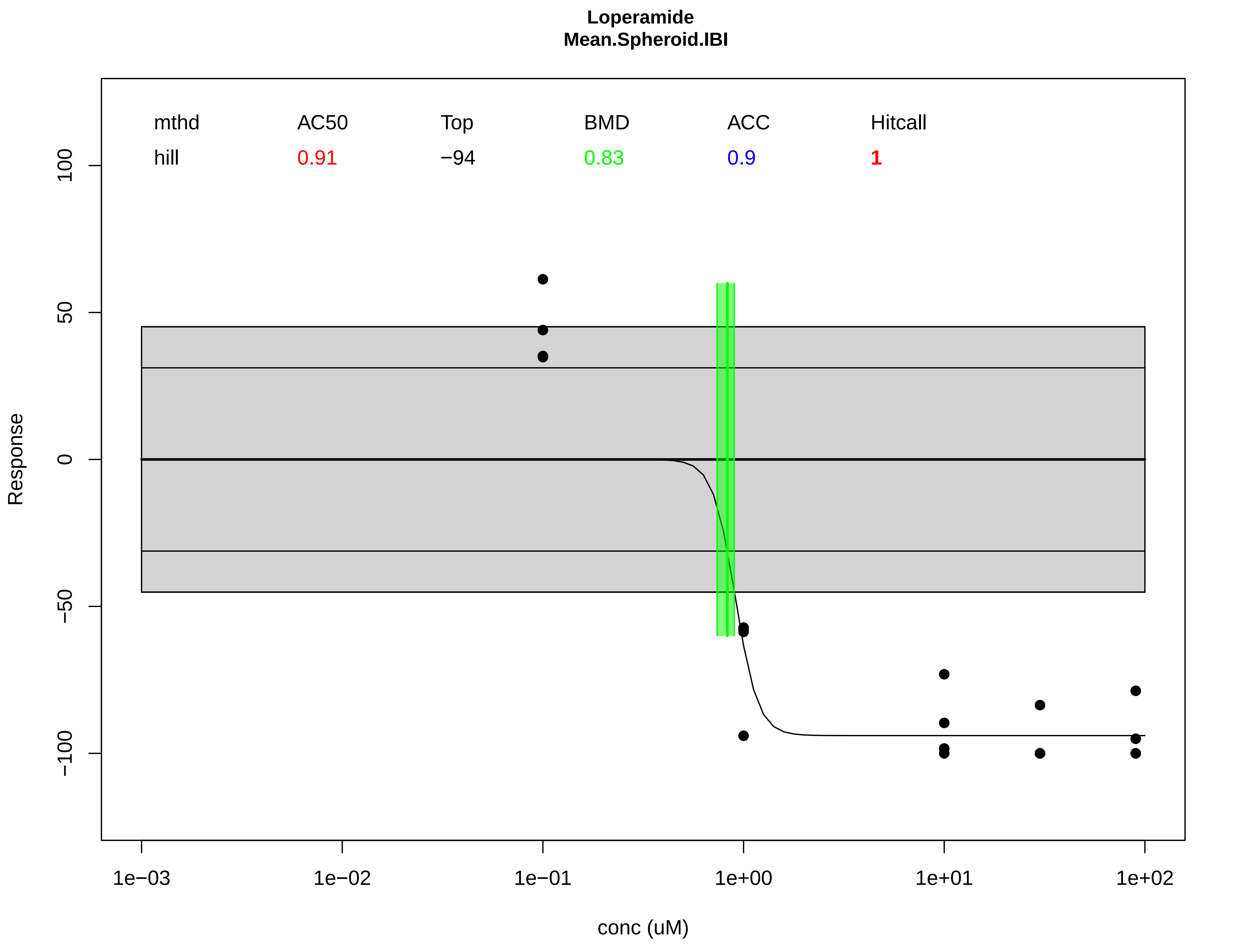

Loperamide
Mean.Spheroid.IBI
mthd
AC50
Top
BMD
ACC
Hitcall
hill
0.91
−94
0.83
0.9
1
Response
100
50
0
−50
−100
1e−03
1e−02
1e−01
1e+00
1e+01
1e+02
conc (uM)

Methylmercuric(II) chloride
Mean.Spheroid.IBI

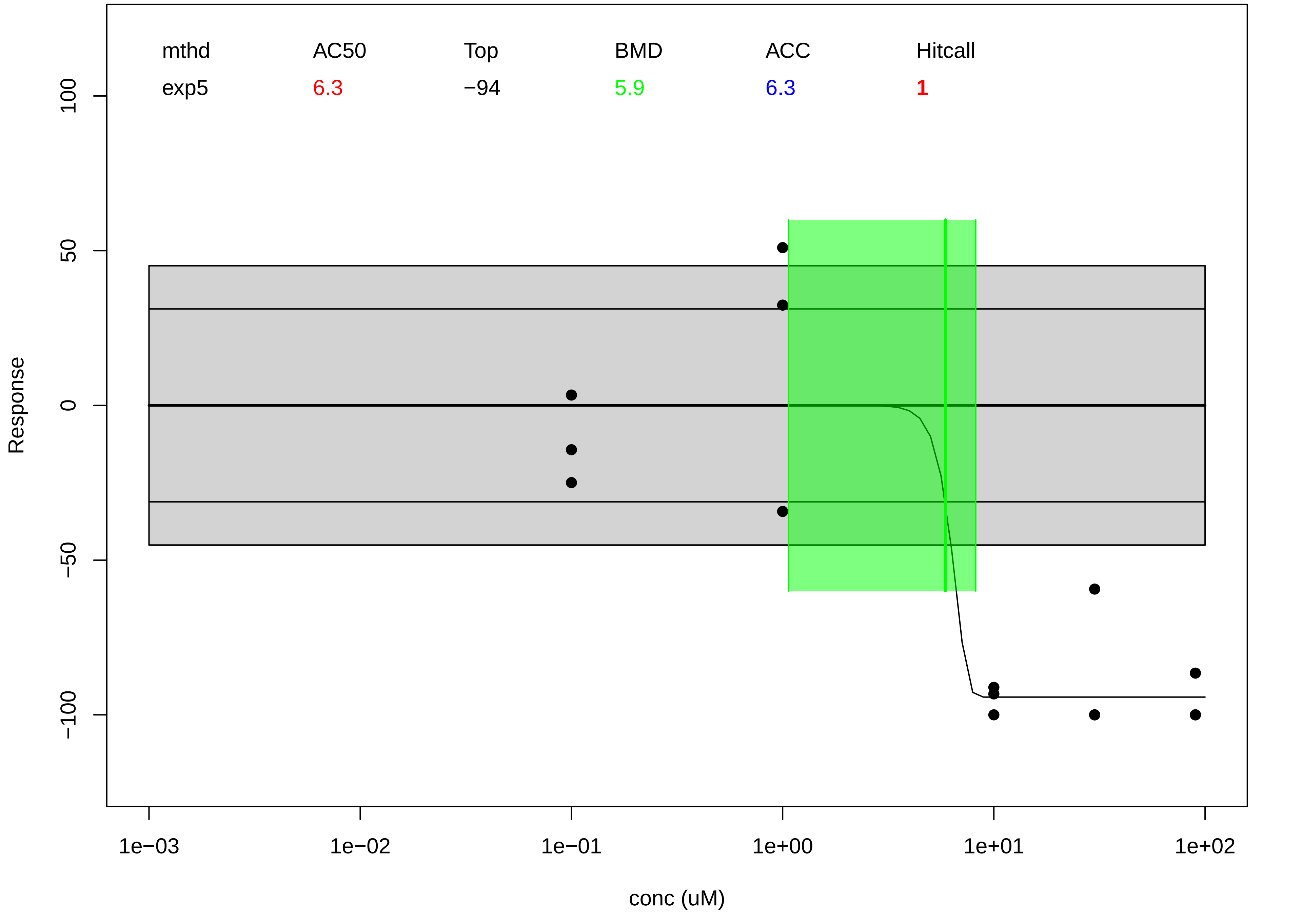

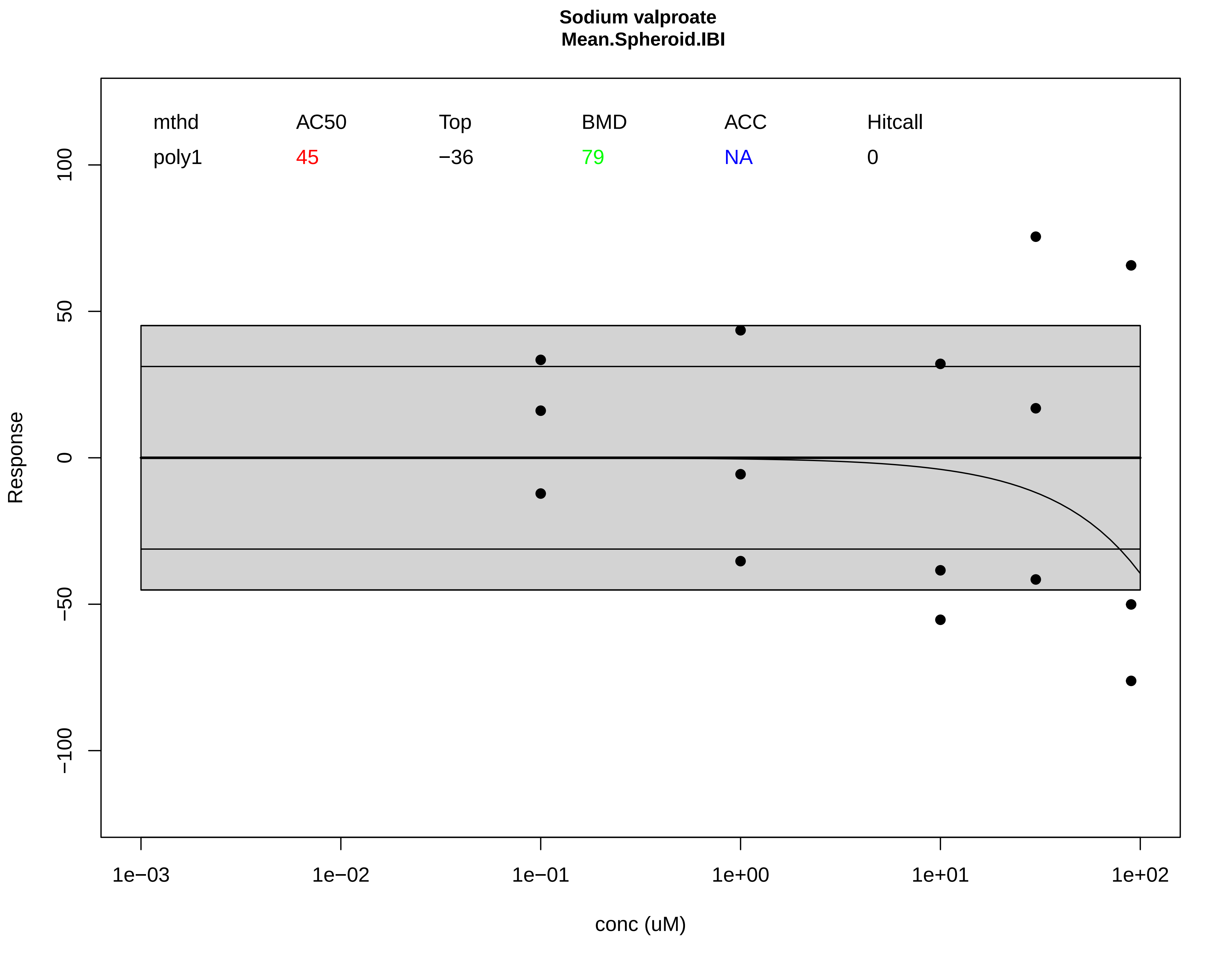

Sodium valproate
Mean.Spheroid.IBI
mthd
AC50
Top
BMD
ACC
Hitcall
poly1
45
−36
79
NA
0
100
50
0
−50
−100
Response
1e−03
1e−02
1e−01
1e+00
1e+01
1e+02
conc (uM)

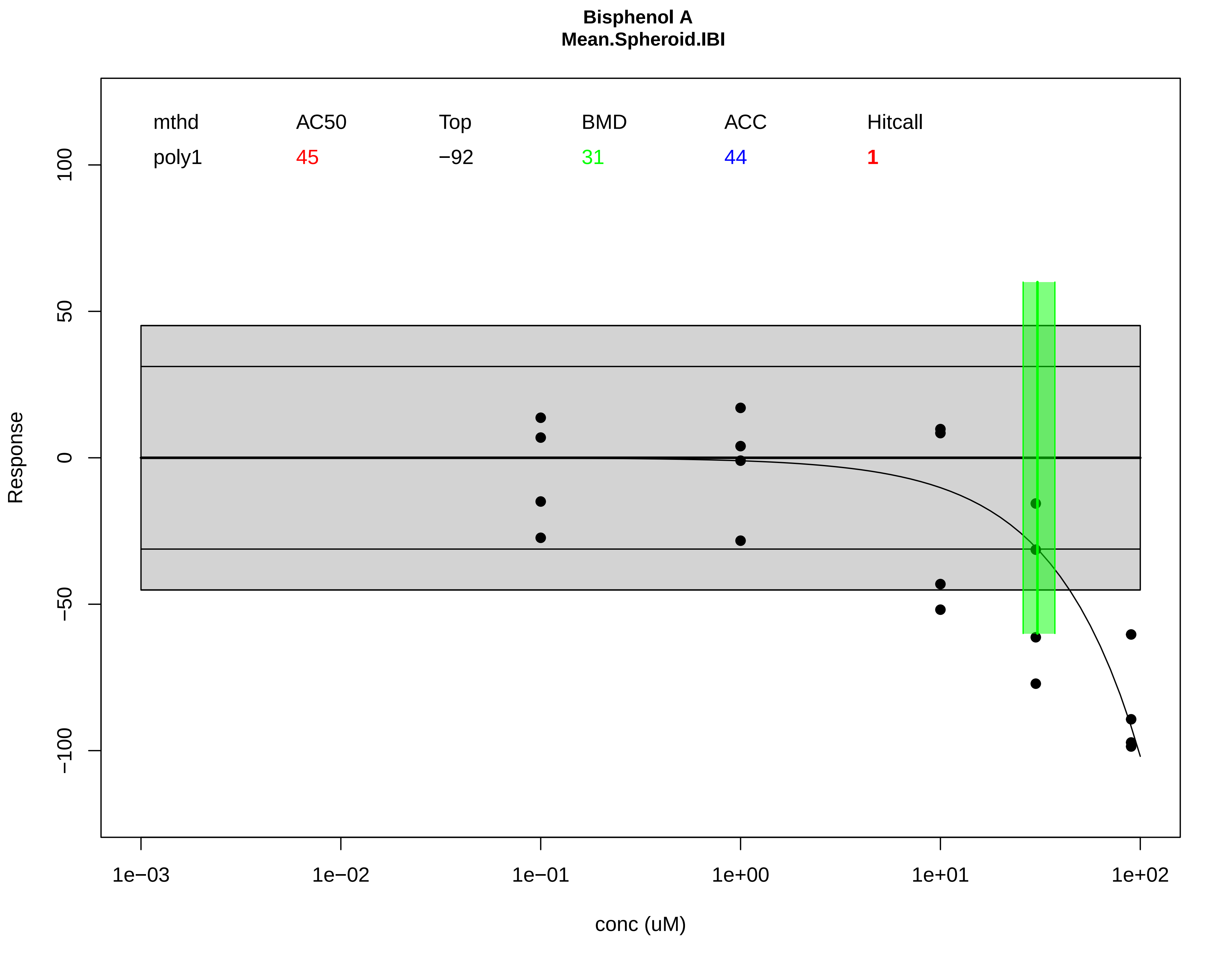
Bisphenol A
Mean.Spheroid.IBI
mthd
AC50
Top
BMD
ACC
Hitcall
poly1
45
−92
31
44
1
100
50
0
−50
−100
Response
1e−03
1e−02
1e−01
1e+00
1e+01
1e+02
conc (uM)

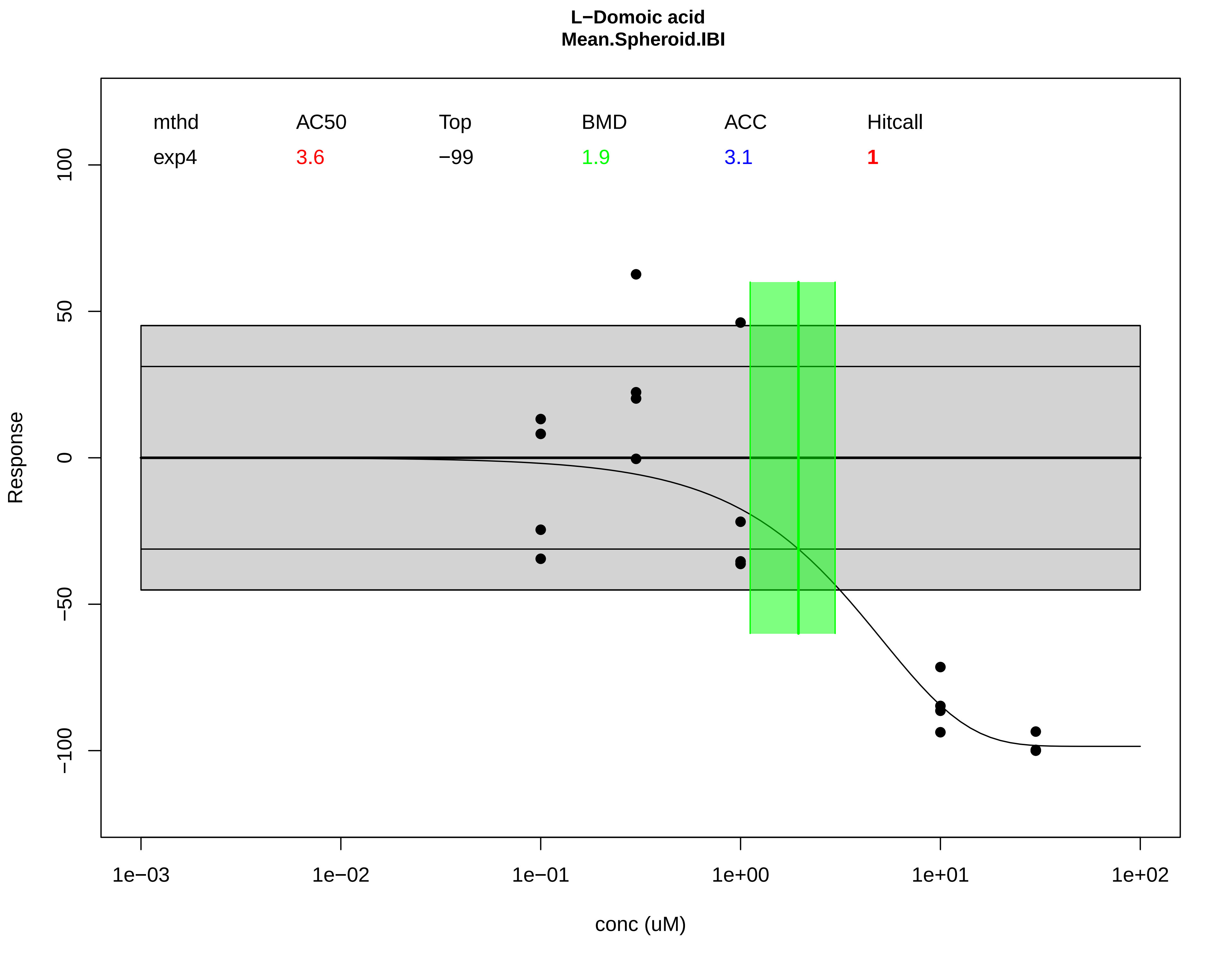
L−Domoic acid
Mean.Spheroid.IBI
mthd
AC50
Top
BMD
ACC
Hitcall
exp4
3.6
−99
1.9
3.1
1
Response
100
50
0
−50
−100
1e−03
1e−02
1e−01
1e+00
1e+01
1e+02
conc (uM)

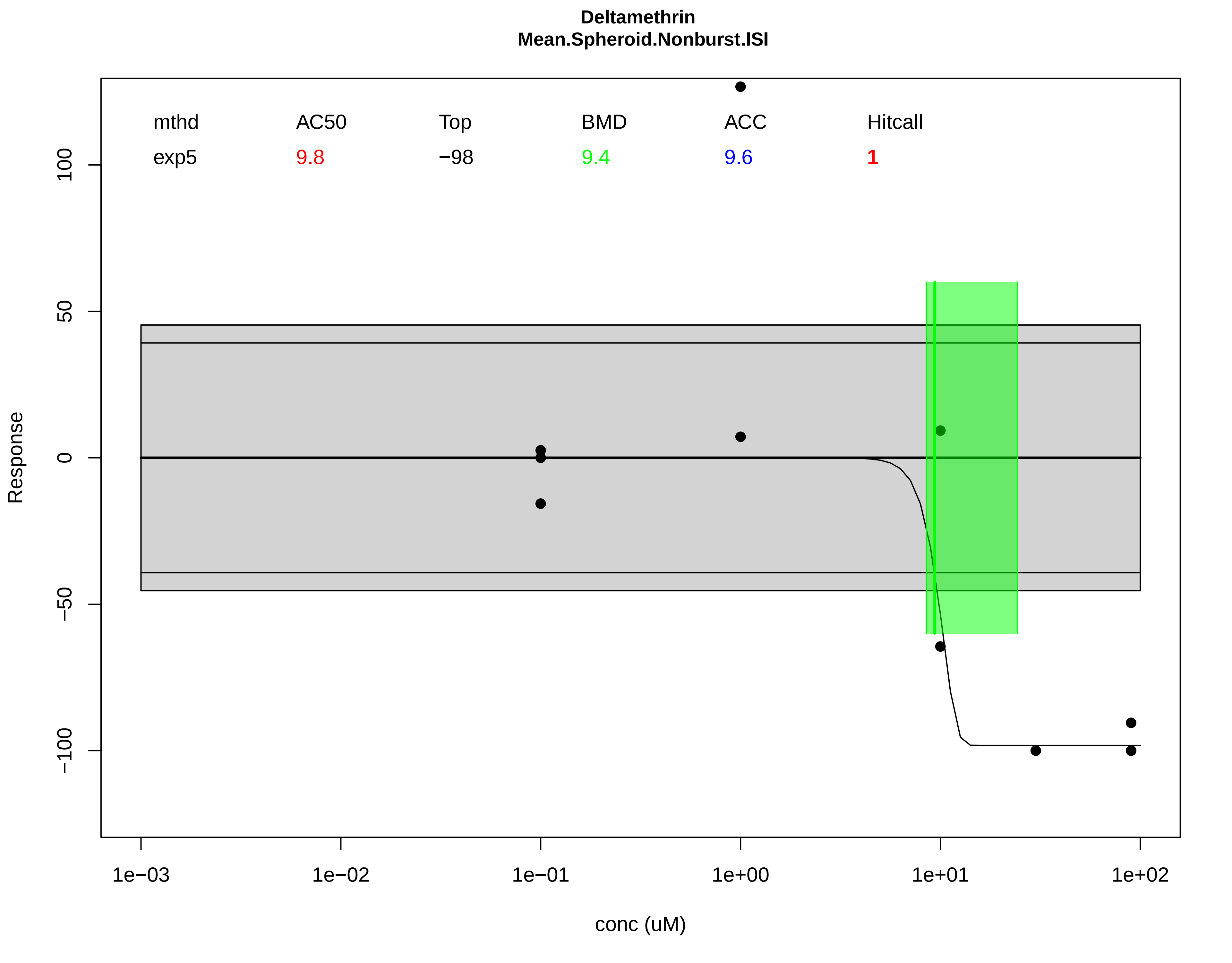

Deltamethrin
Mean.Spheroid.Nonburst.ISI
mthd
AC50
Top
BMD
ACC
Hitcall
exp5
9.8
−98
9.4
9.6
1
100
50
0
−50
−100
Response
1e−03
1e−02
1e−01
1e+00
1e+01
1e+02
conc (uM)

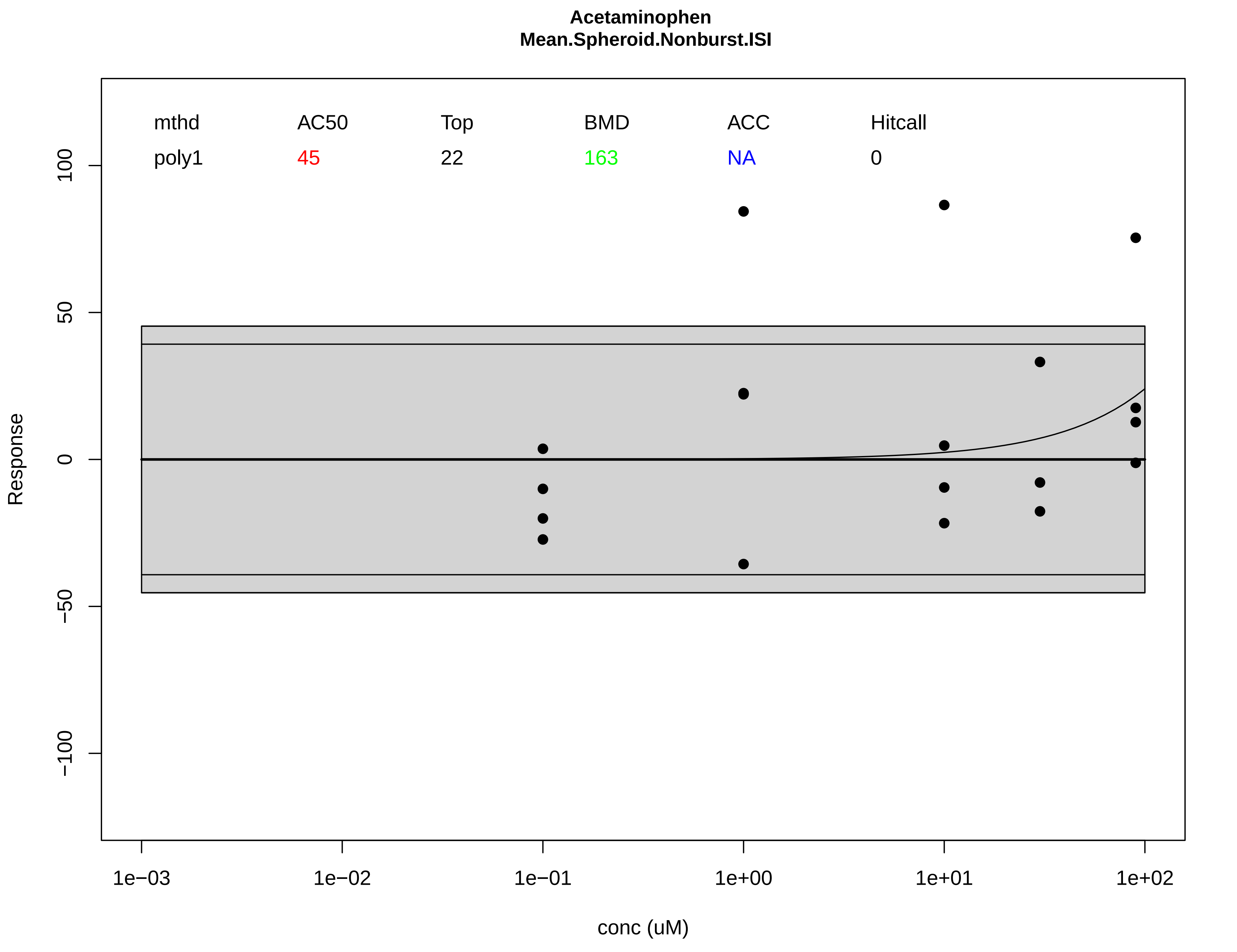
Acetaminophen
Mean.Spheroid.Nonburst.ISI
mthd
AC50
Top
BMD
ACC
Hitcall
poly1
45
22
163
NA
0
Response
100
50
0
−50
−100
1e−03
1e−02
1e−01
1e+00
1e+01
1e+02
conc (uM)

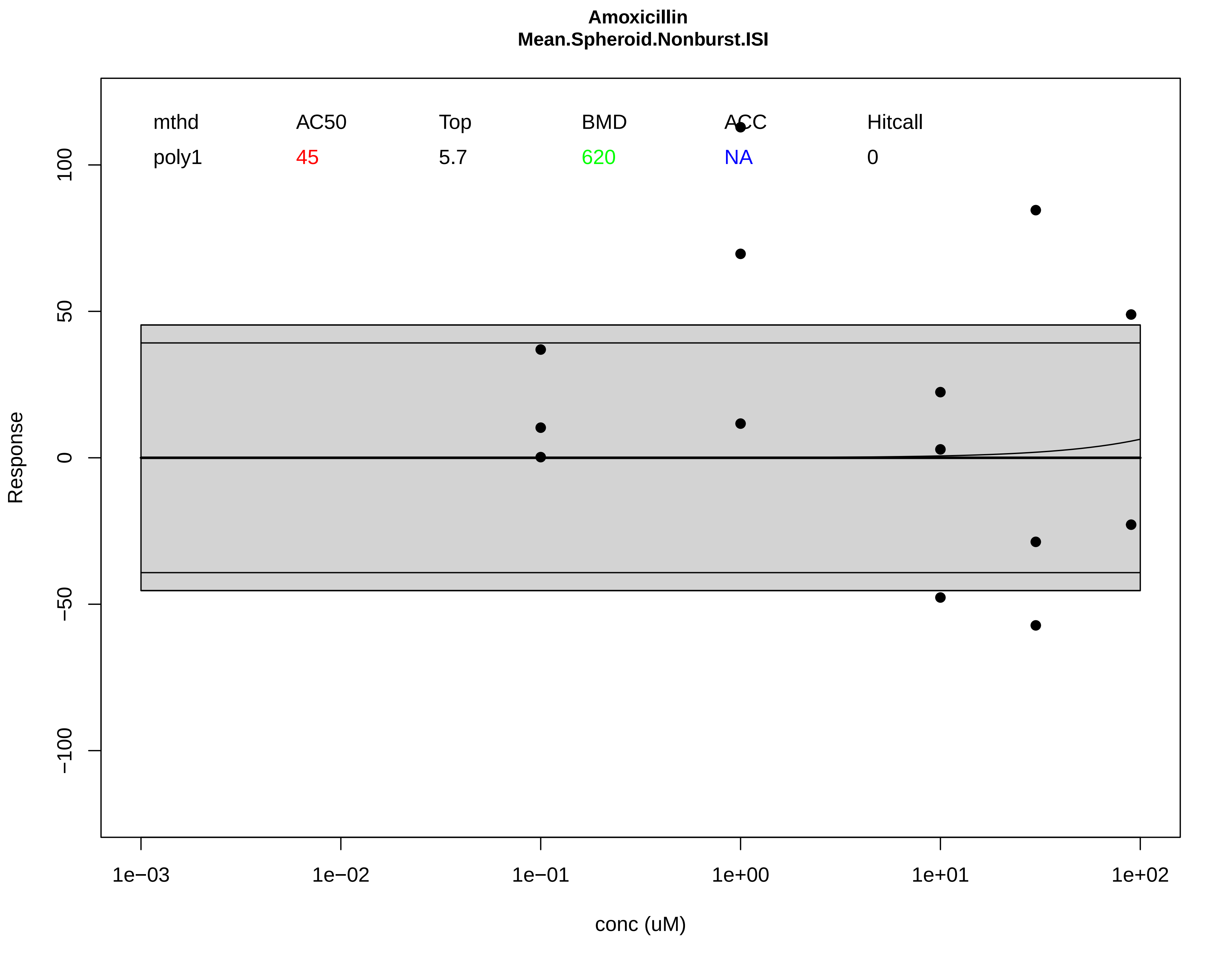
Amoxicillin
Mean.Spheroid.Nonburst.ISI
mthd
AC50
Top
BMD
ACC
Hitcall
poly1
45
5.7
620
NA
0
100
50
0
−50
−100
Response
1e−03
1e−02
1e−01
1e+00
1e+01
1e+02
conc (uM)

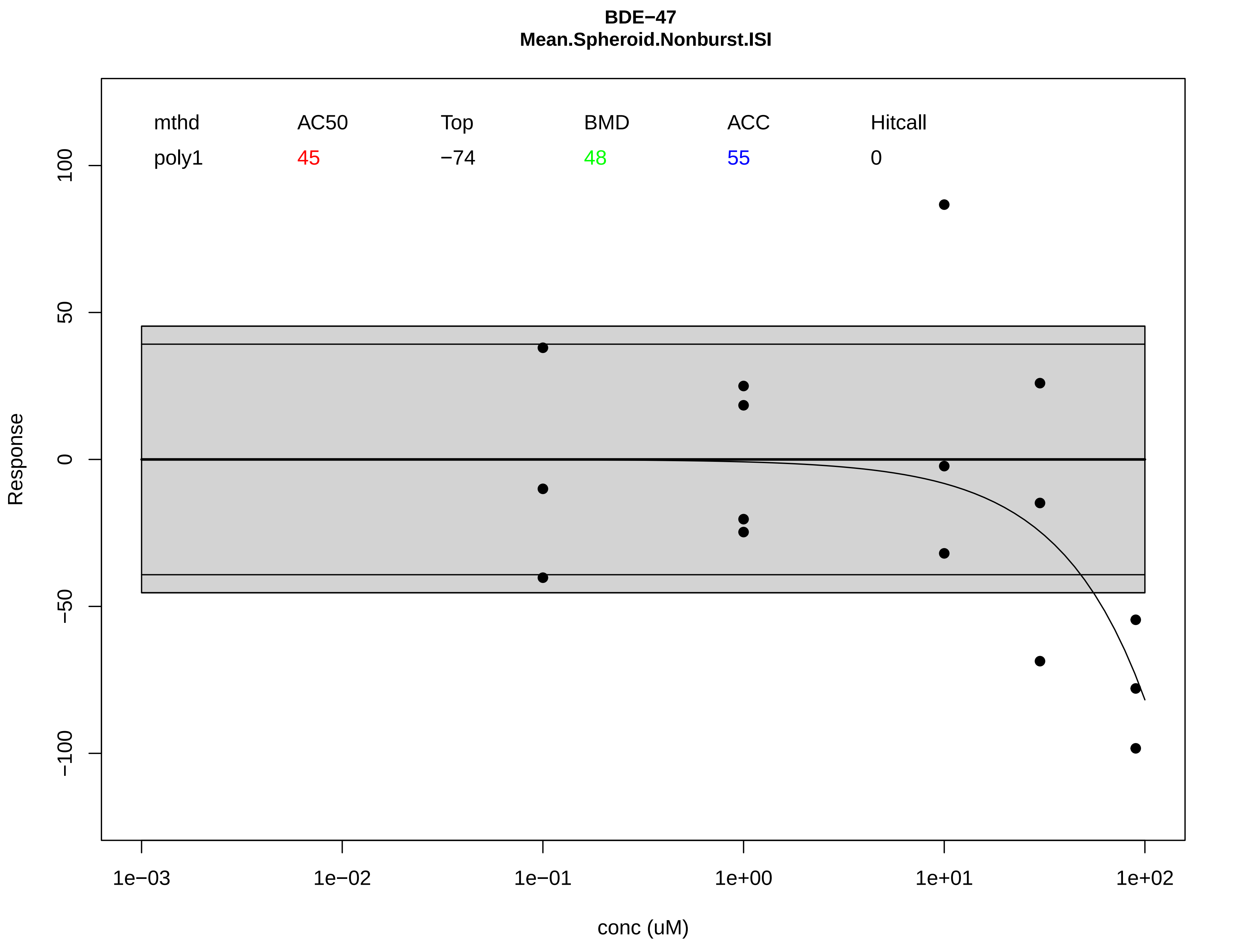
BDE−47
Mean.Spheroid.Nonburst.ISI
mthd
AC50
Top
BMD
ACC
Hitcall
poly1
45
−74
48
55
0
100
50
0
−50
−100
Response
1e−03
1e−02
1e−01
1e+00
1e+01
1e+02
conc (uM)

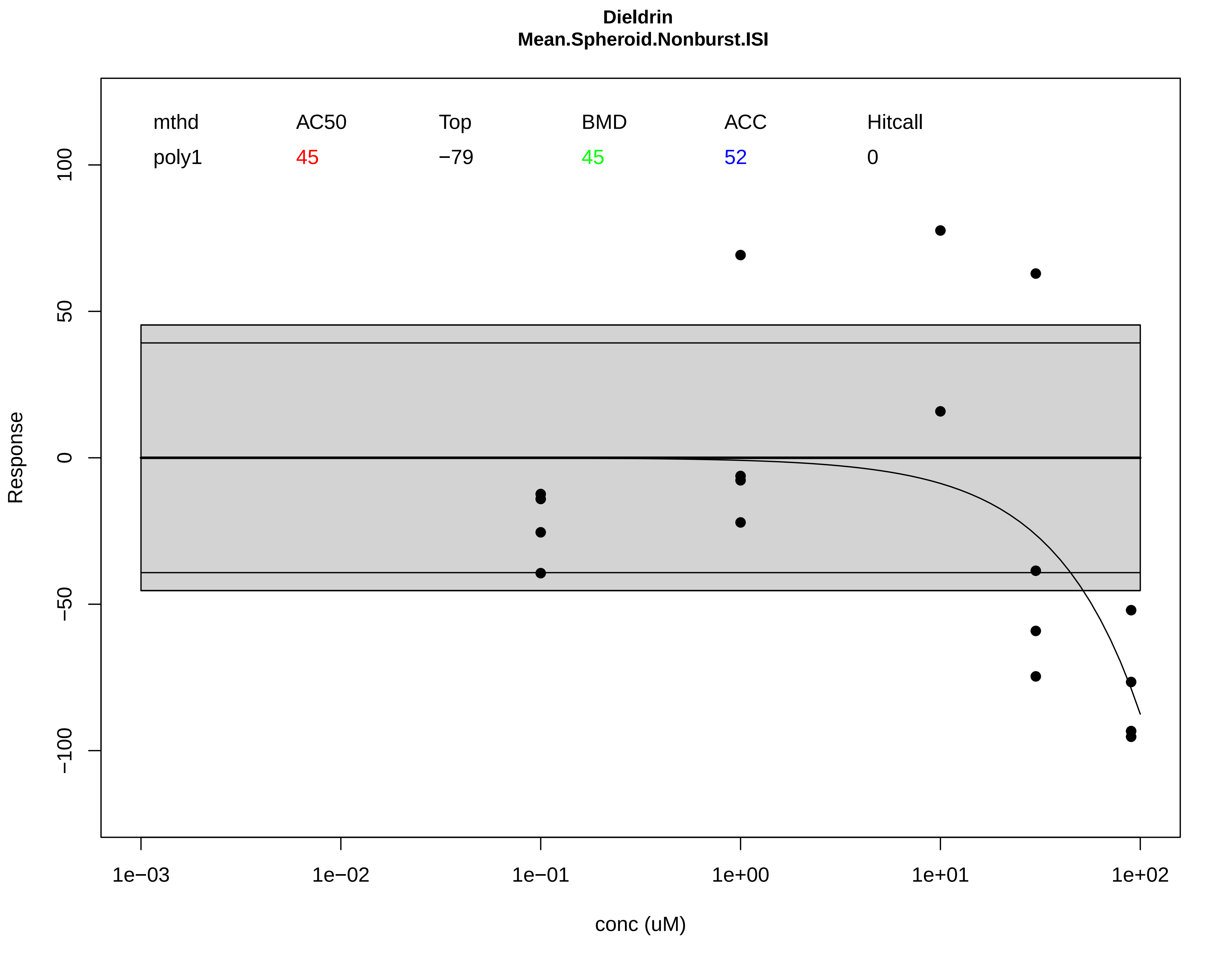

Dieldrin
Mean.Spheroid.Nonburst.ISI
mthd
AC50
Top
BMD
ACC
Hitcall
poly1
45
−79
45
52
0
100
50
0
−50
−100
Response
1e−03
1e−02
1e−01
1e+00
1e+01
1e+02
conc (uM)

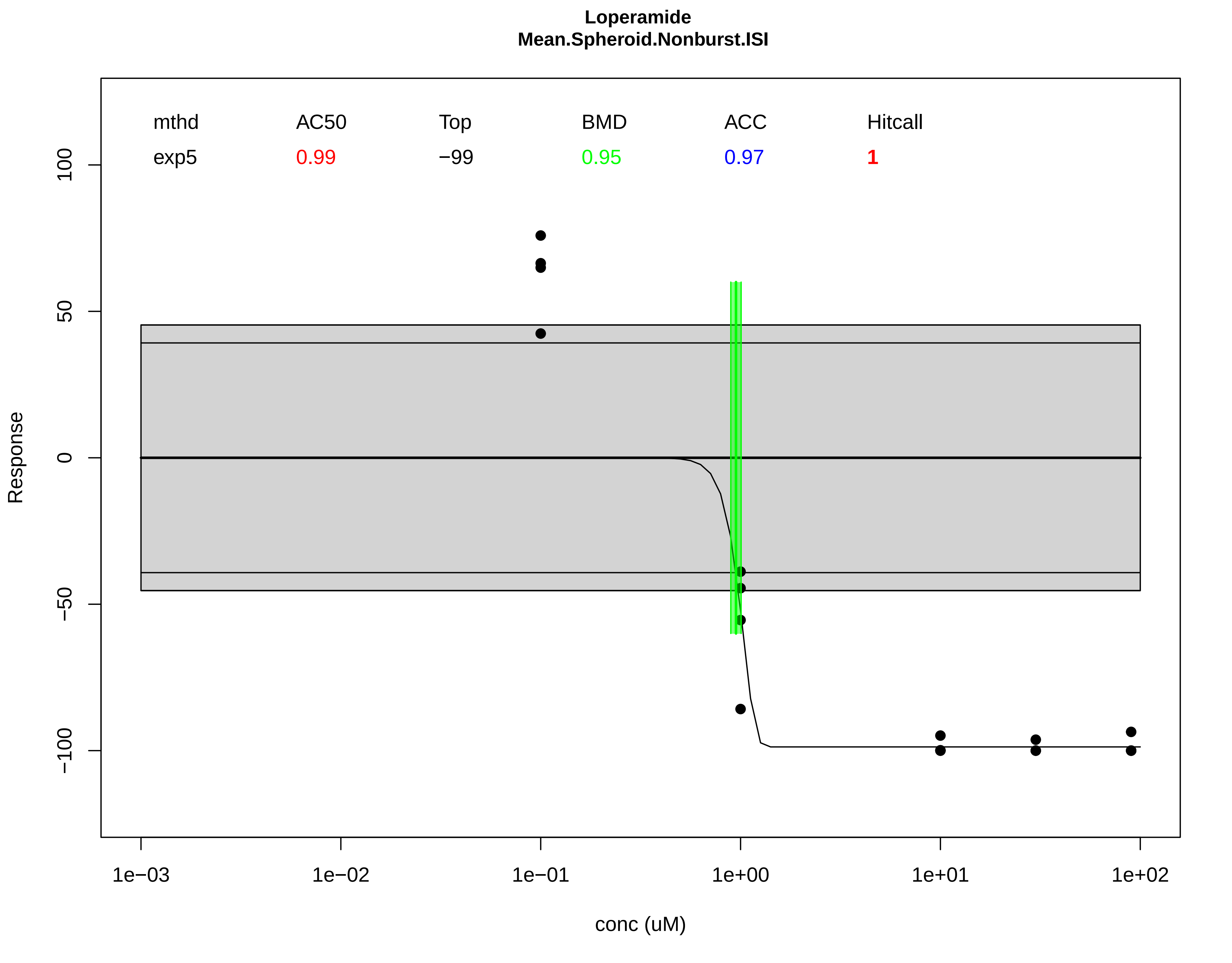
Loperamide
Mean.Spheroid.Nonburst.ISI
mthd
AC50
Top
BMD
ACC
Hitcall
exp5
0.99
−99
0.95
0.97
1
100
50
0
−50
−100
Response
1e−03
1e−02
1e−01
1e+00
1e+01
1e+02
conc (uM)

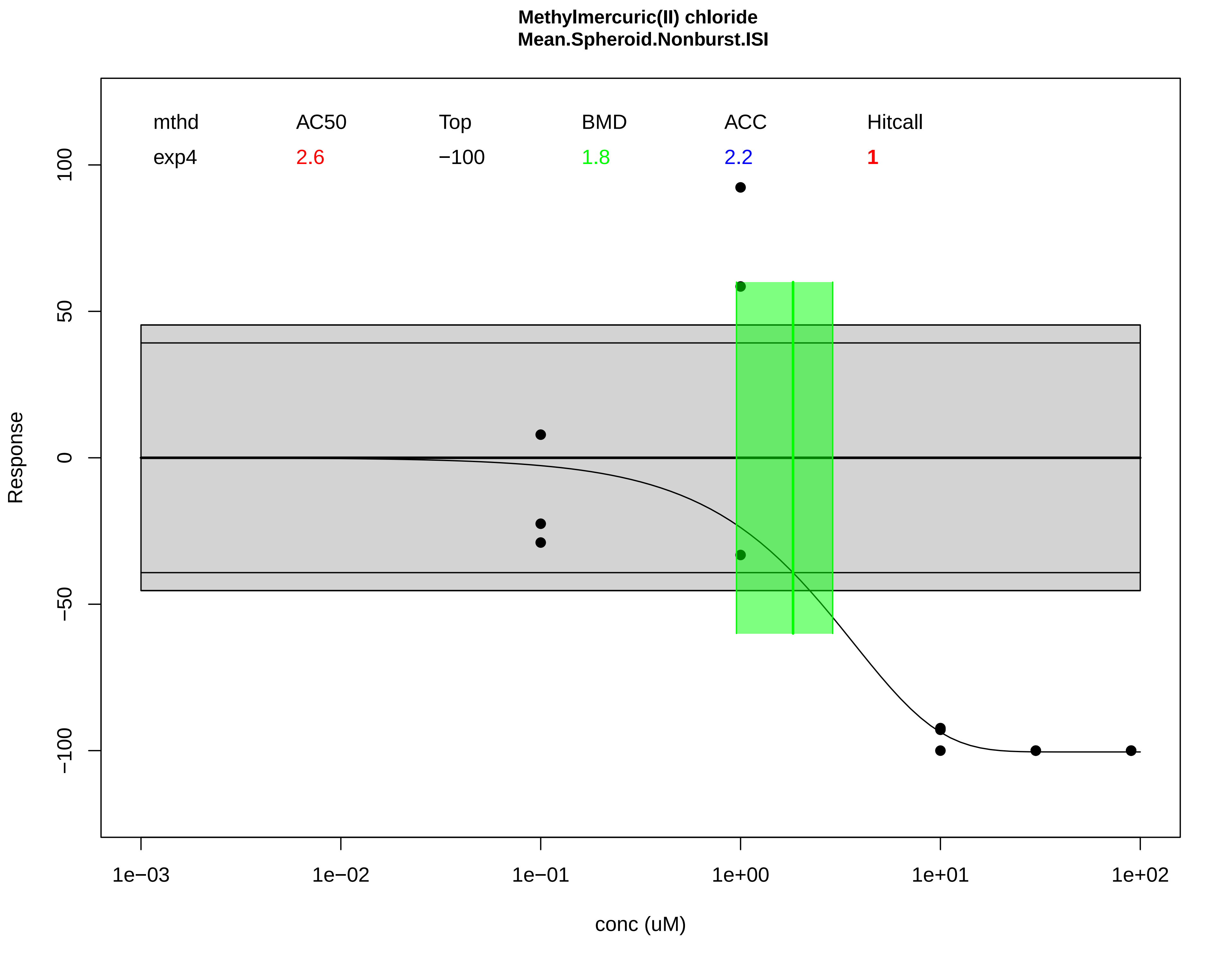
Methylmercuric(II) chloride
Mean.Spheroid.Nonburst.ISI
mthd
exp4
AC50
2.6
Top
−100
BMD
1.8
ACC
2.2
Hitcall
1
Response
100
50
0
−50
−100
1e−03
1e−02
1e−01
1e+00
1e+01
1e+02
conc (uM)

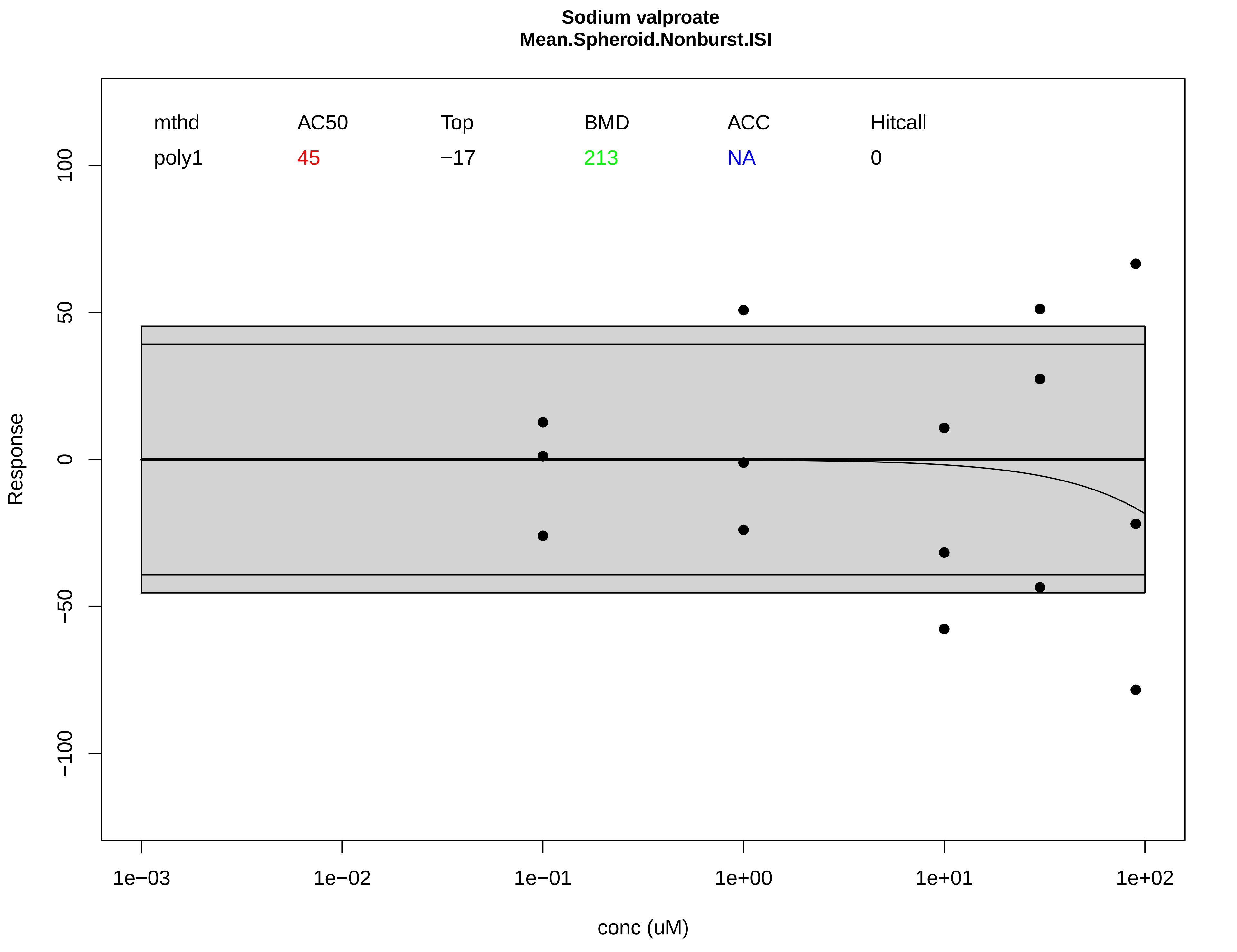
Sodium valproate
Mean.Spheroid.Nonburst.ISI
mthd
AC50
Top
BMD
ACC
Hitcall
poly1
45
−17
213
NA
0
100
50
0
−50
−100
Response
1e−03
1e−02
1e−01
1e+00
1e+01
1e+02
conc (uM)

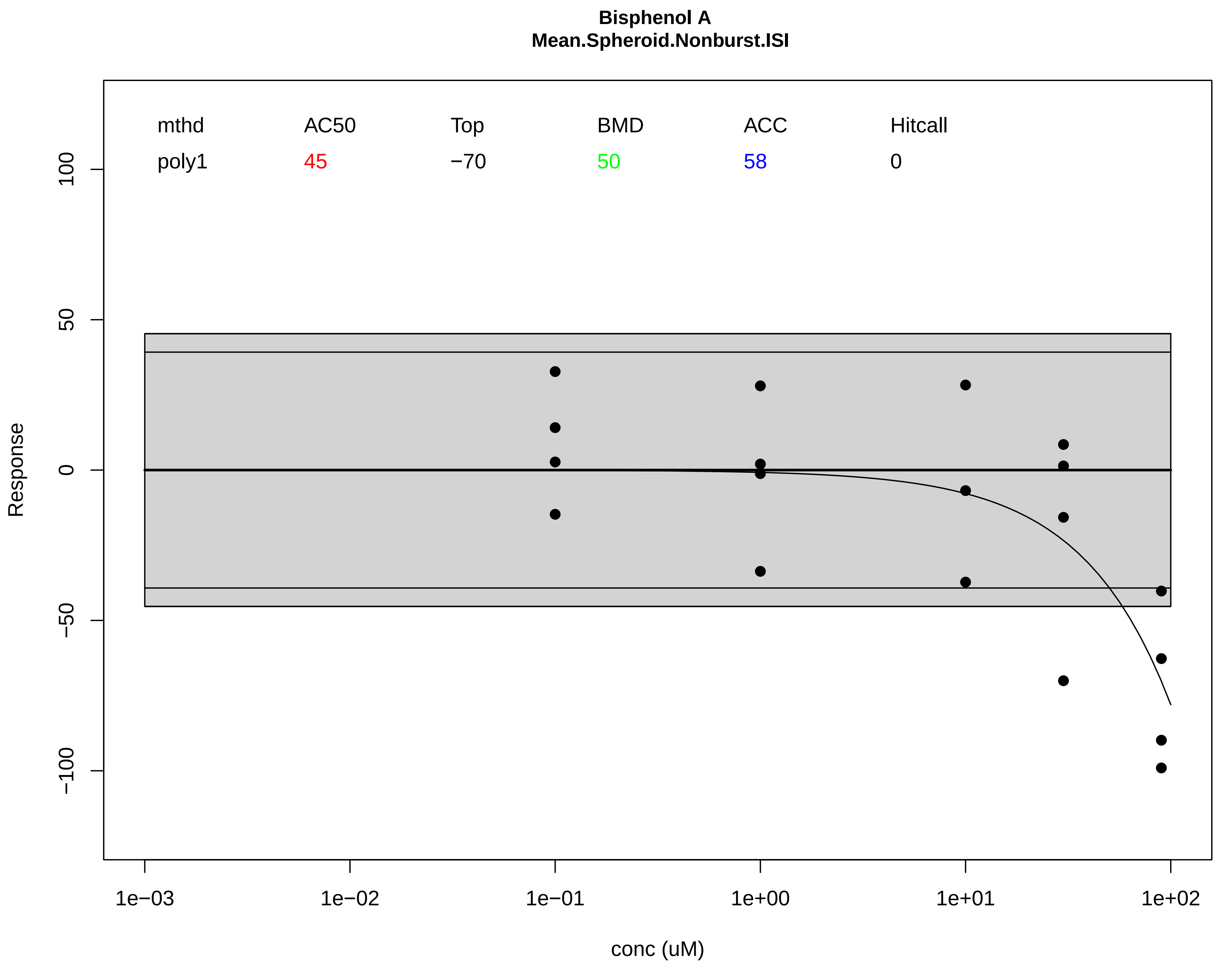
Bisphenol A
Mean.Spheroid.Nonburst.ISI
mthd
AC50
Top
BMD
ACC
Hitcall
poly1
45
−70
50
58
0
100
50
0
−50
−100
Response
1e−03
1e−02
1e−01
1e+00
1e+01
1e+02
conc (uM)

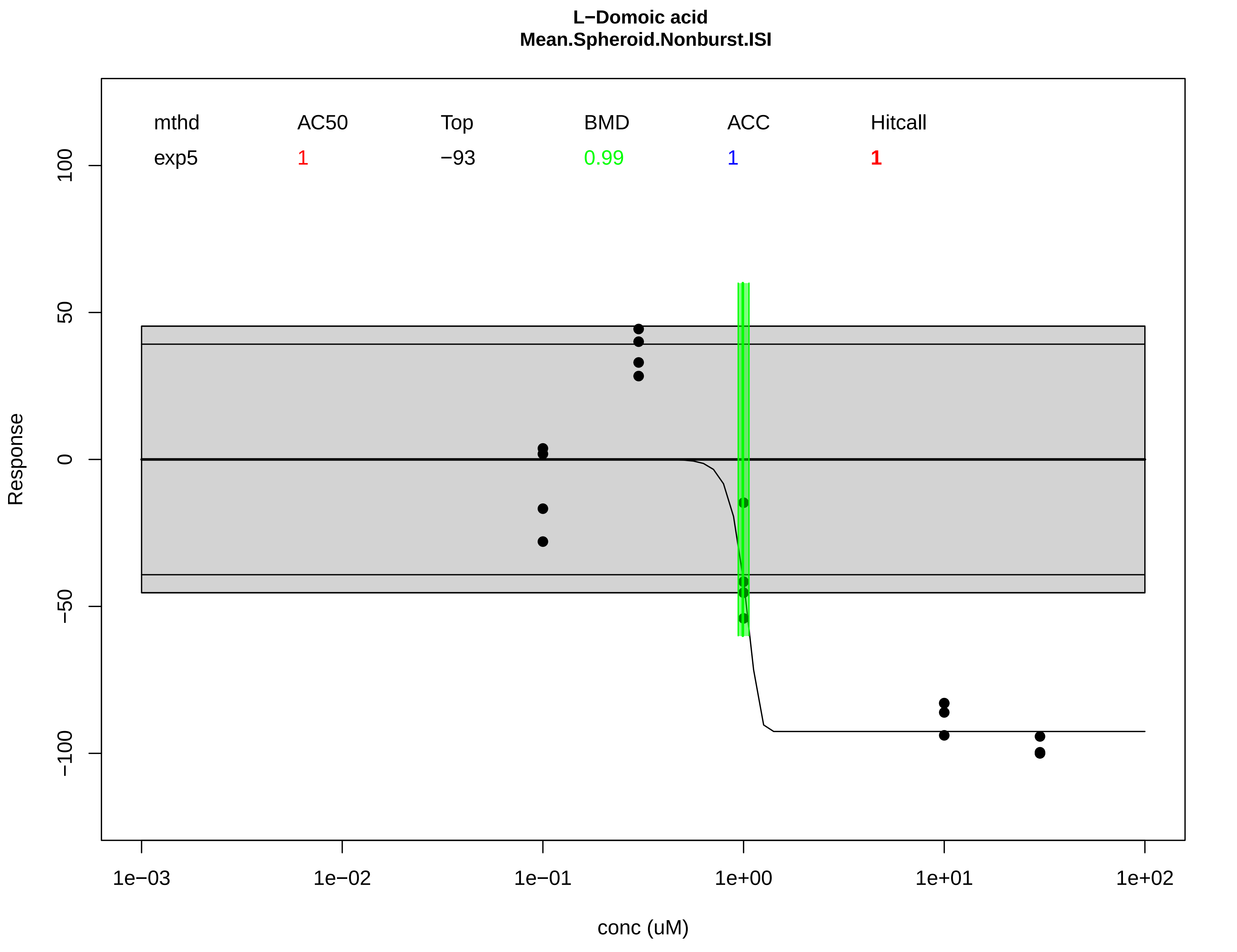

L−Domoic acid
Mean.Spheroid.Nonburst.ISI
mthd
AC50
Top
BMD
ACC
Hitcall
exp5
1
−93
0.99
1
1
100
50
0
−50
−100
Response
1e−03
1e−02
1e−01
1e+00
1e+01
1e+02
conc (uM)

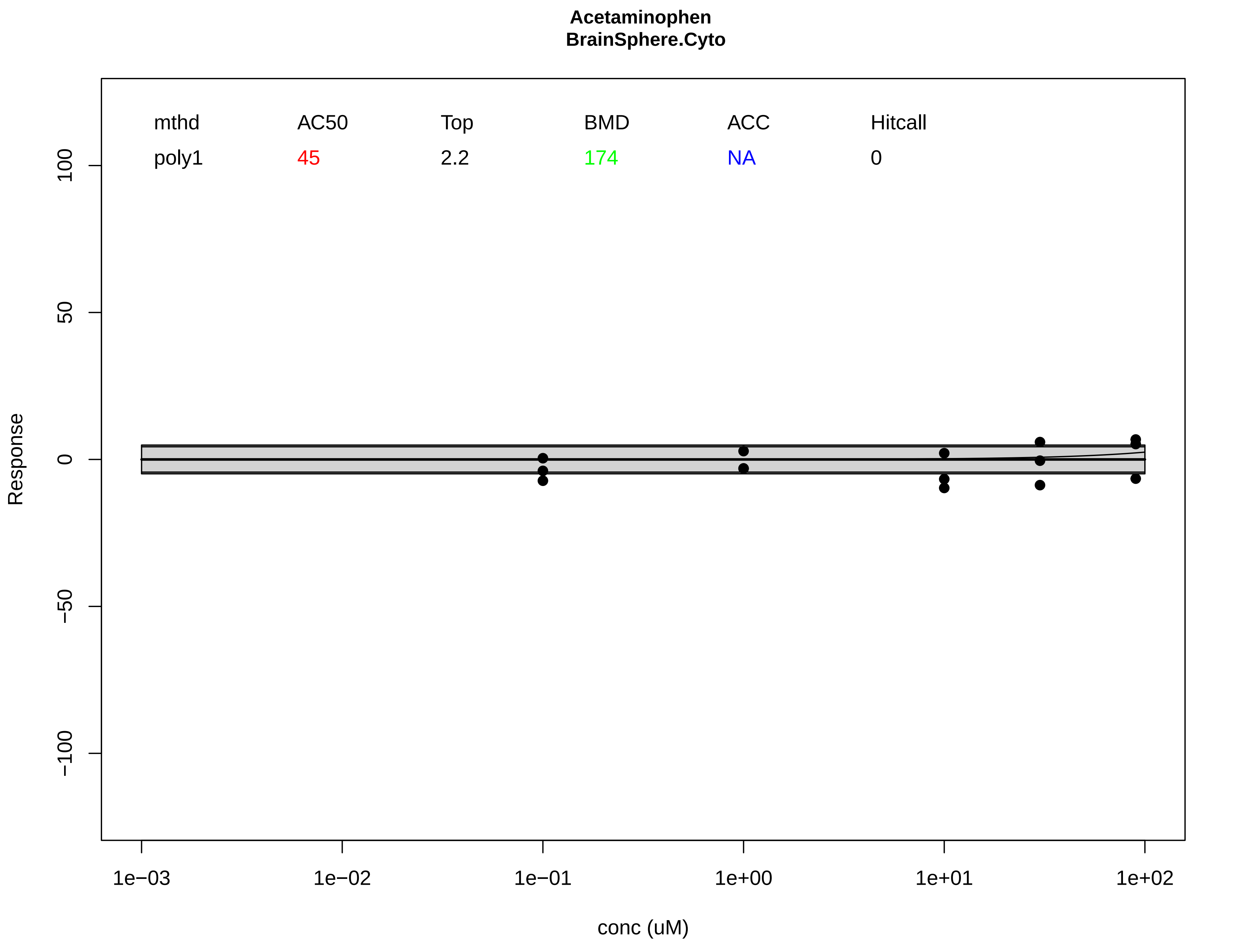
Acetaminophen
BrainSphere.Cyto
mthd
AC50
Top
BMD
ACC
Hitcall
poly1
45
2.2
174
NA
0
100
50
0
−50
−100
Response
1e−03
1e−02
1e−01
1e+00
1e+01
1e+02
conc (uM)

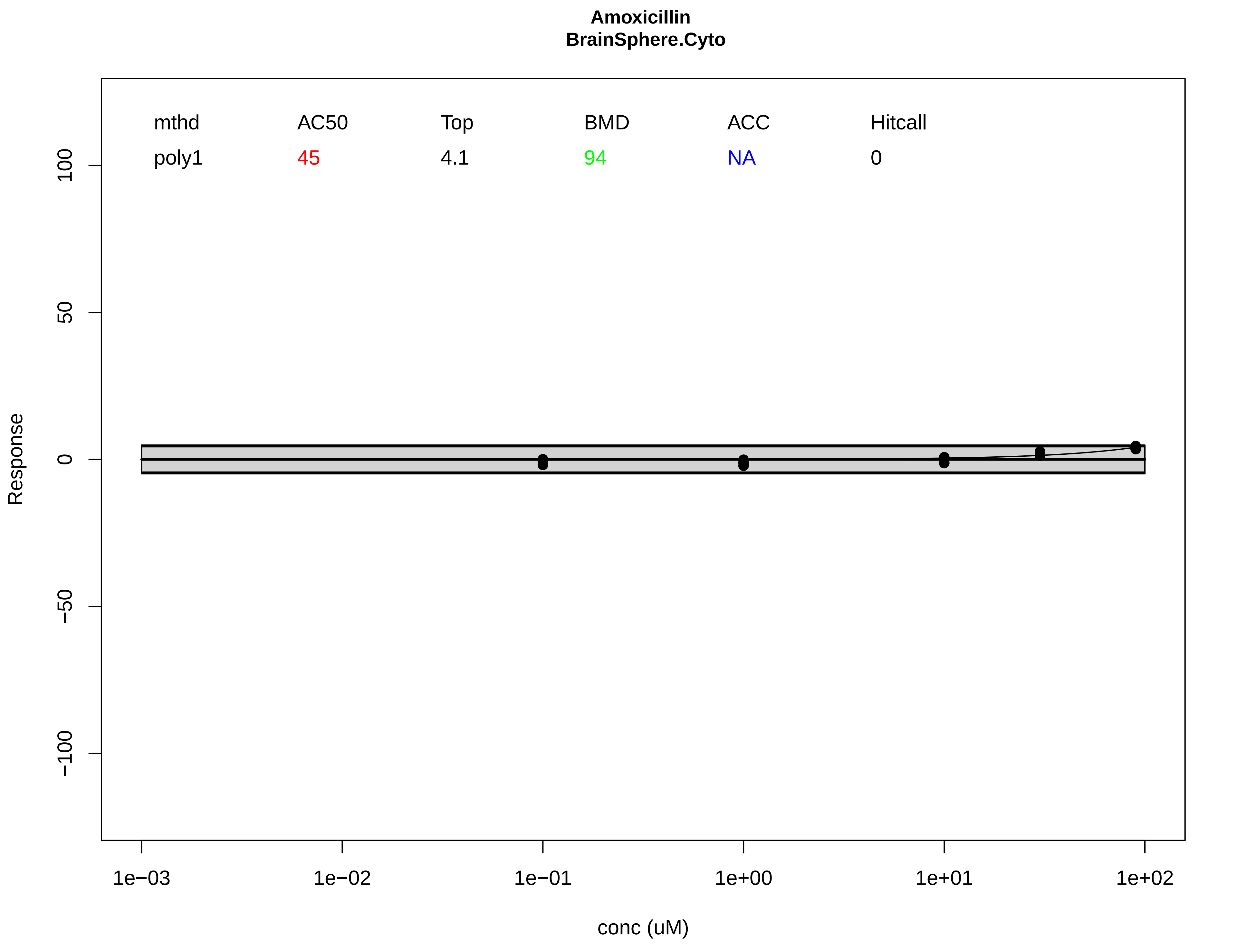

Amoxicillin
BrainSphere.Cyto
mthd
AC50
Top
BMD
ACC
Hitcall
poly1
45
4.1
94
NA
0
100
50
0
−50
−100
Response
1e−03
1e−02
1e−01
1e+00
1e+01
1e+02
conc (uM)

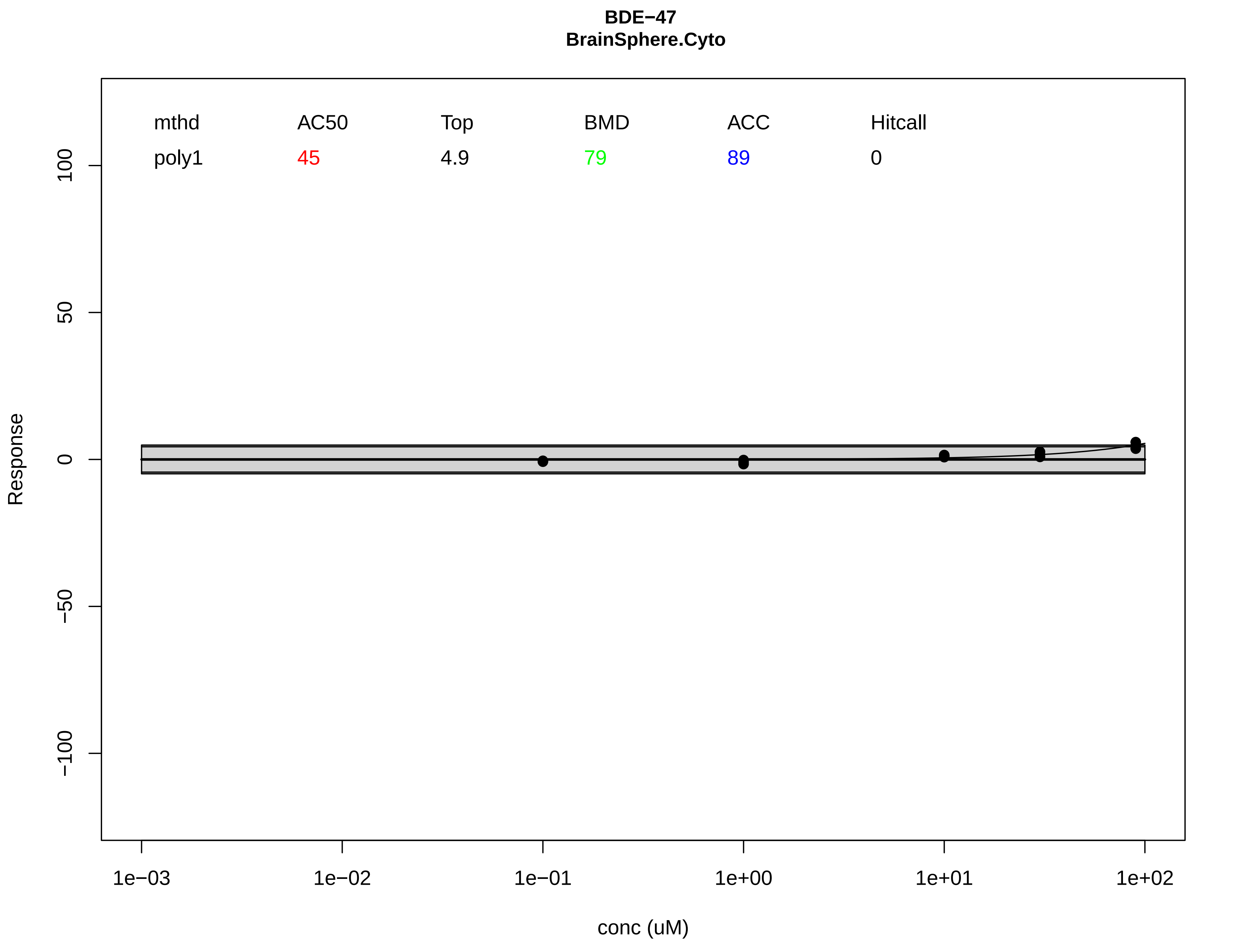
BDE−47
BrainSphere.Cyto
mthd
AC50
Top
BMD
ACC
Hitcall
poly1
45
4.9
79
89
0
100
50
0
−50
−100
Response
1e−03
1e−02
1e−01
1e+00
1e+01
1e+02
conc (uM)

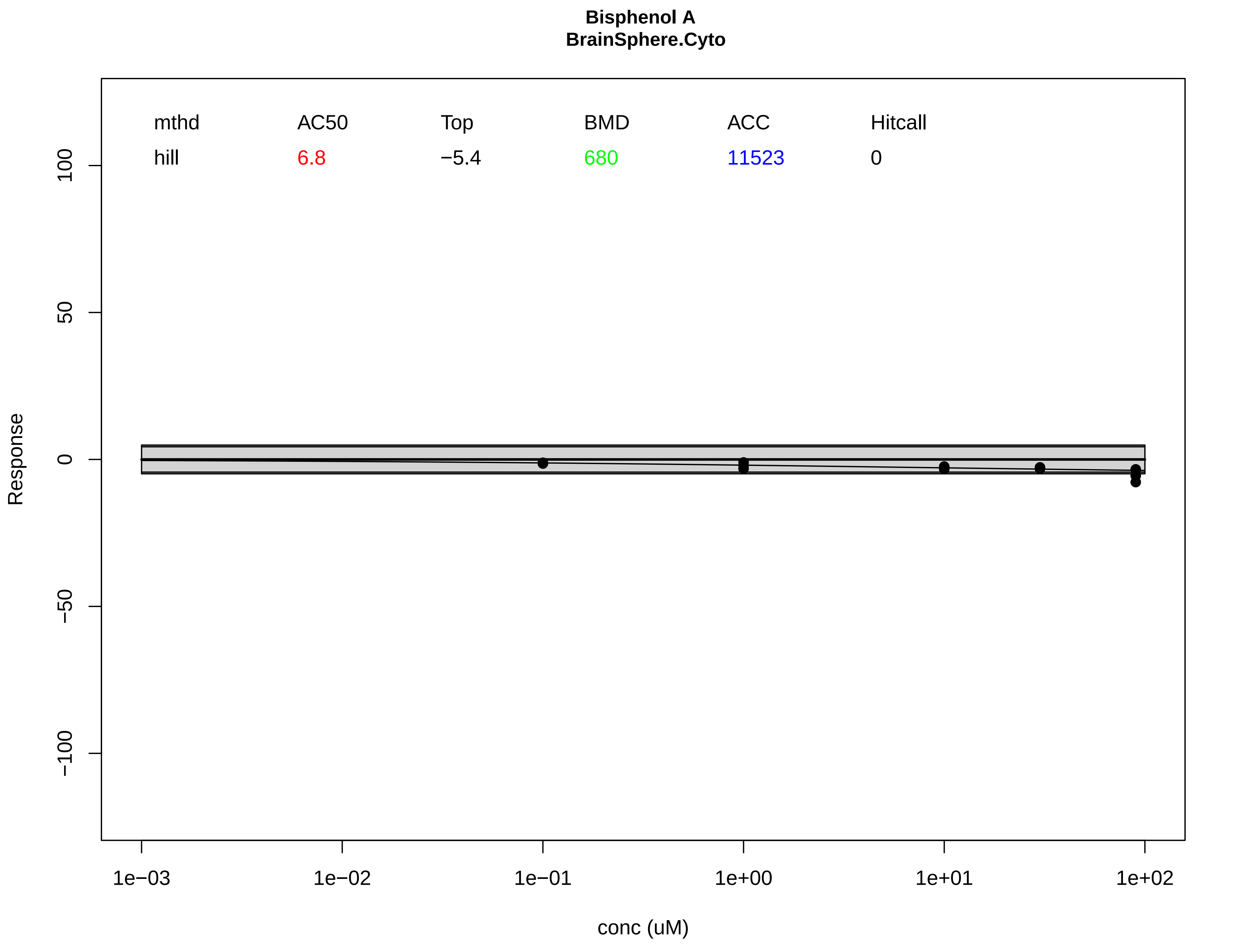
Bisphenol A
BrainSphere.Cyto
mthd
AC50
Top
BMD
ACC
Hitcall
hill
6.8
−5.4
680
11523
0
Response
100
50
0
−50
−100
1e−03
1e−02
1e−01
1e+00
1e+01
1e+02
conc (uM)

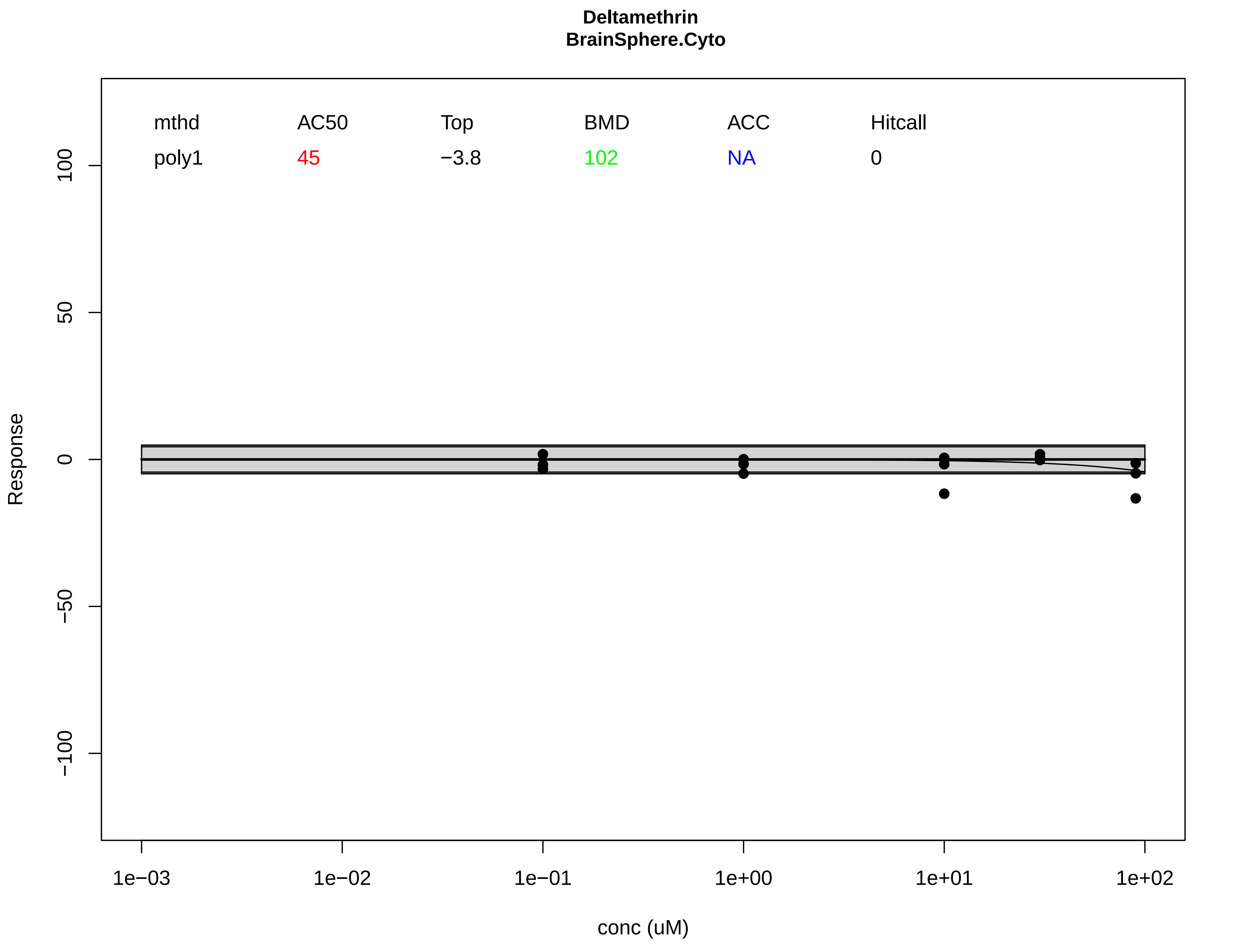
Deltamethrin
BrainSphere.Cyto
mthd
AC50
Top
BMD
ACC
Hitcall
poly1
45
−3.8
102
NA
0
100
50
0
−50
−100
Response
1e−03
1e−02
1e−01
1e+00
1e+01
1e+02
conc (uM)

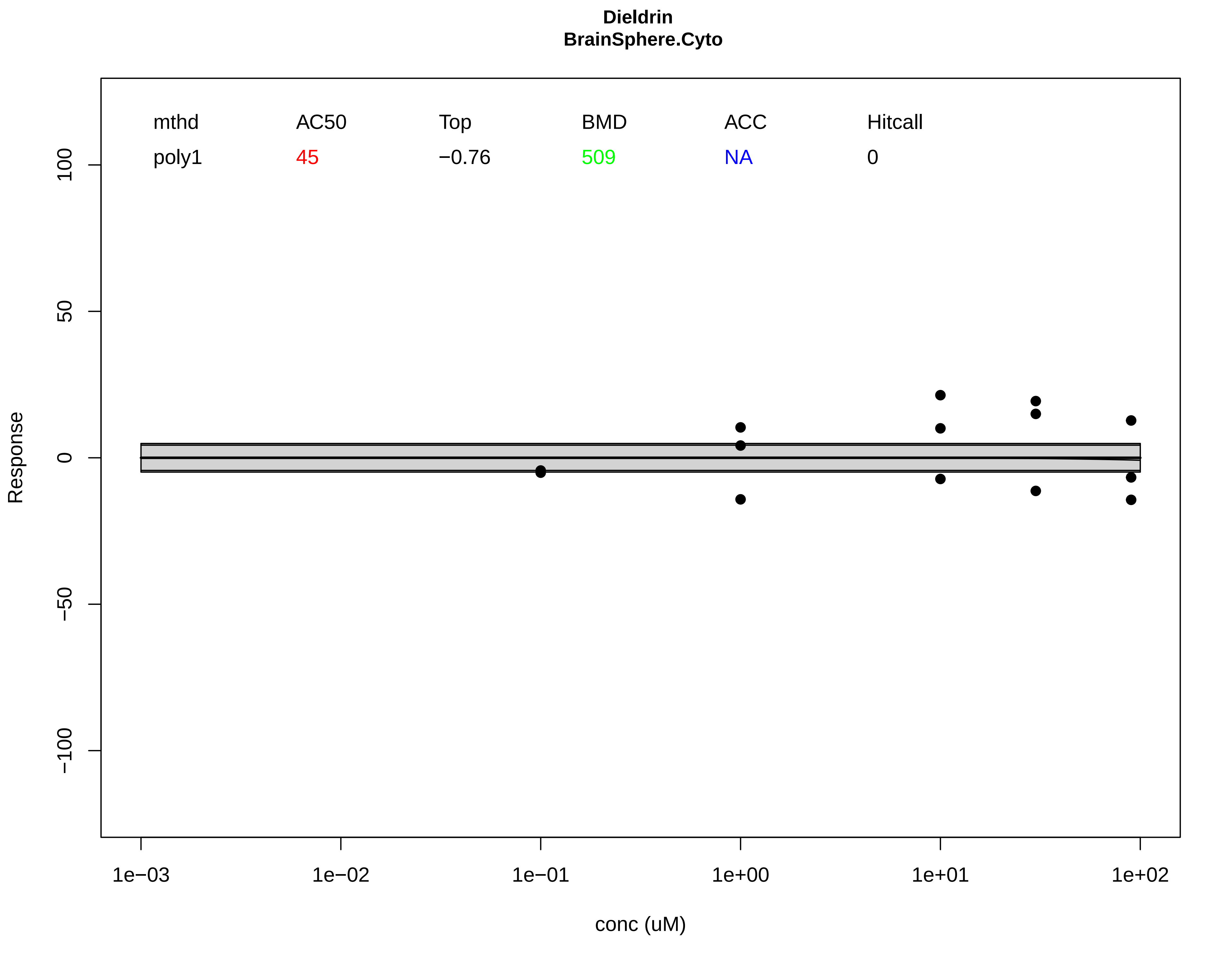
Dieldrin
BrainSphere.Cyto
mthd
AC50
Top
BMD
ACC
Hitcall
poly1
45
−0.76
509
NA
0
100
50
0
−50
−100
Response
1e−03
1e−02
1e−01
1e+00
1e+01
1e+02
conc (uM)

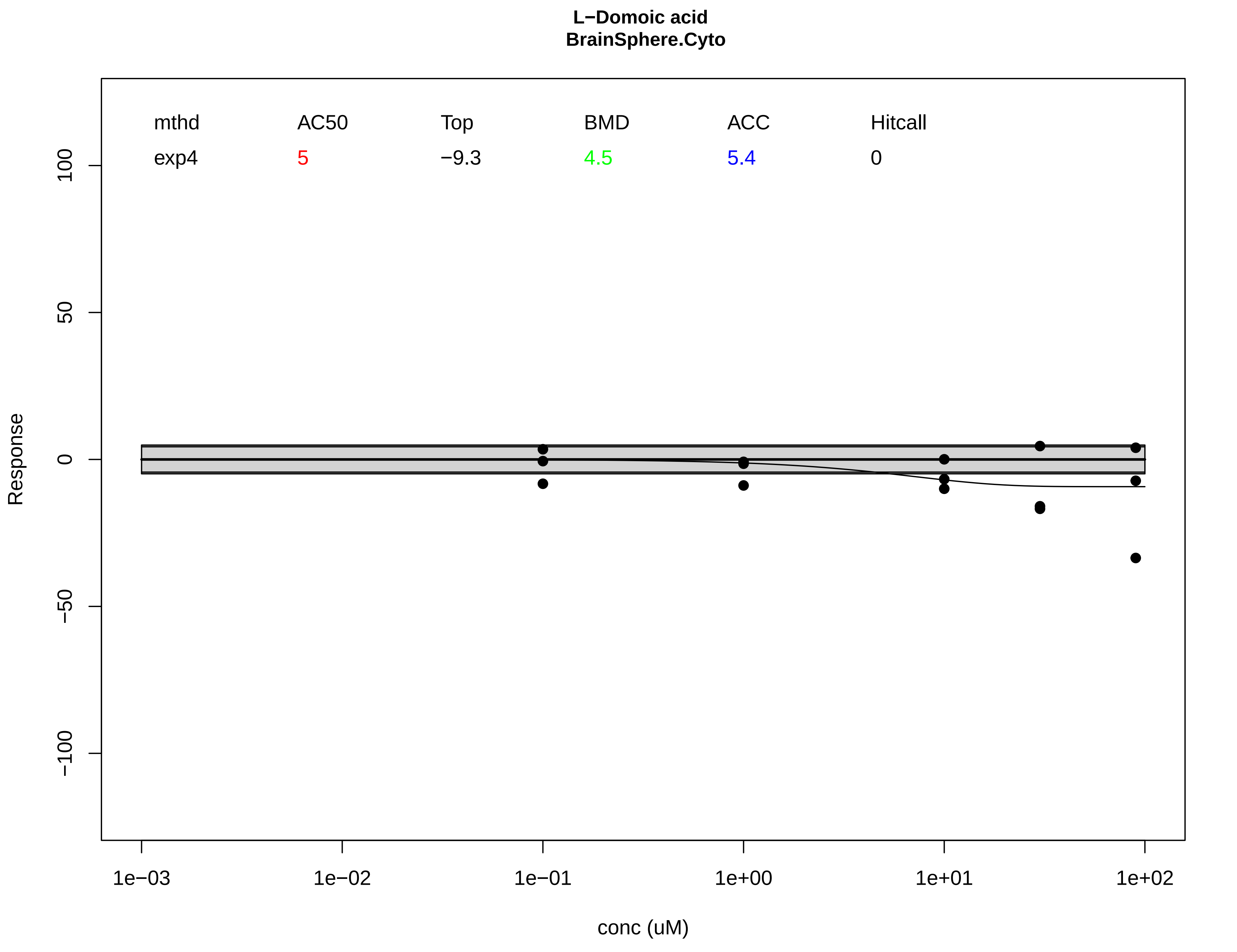

L−Domoic acid
BrainSphere.Cyto
mthd
AC50
Top
BMD
ACC
Hitcall
exp4
5
−9.3
4.5
5.4
0
100
50
0
−50
−100
Response
1e−03
1e−02
1e−01
1e+00
1e+01
1e+02
conc (uM)

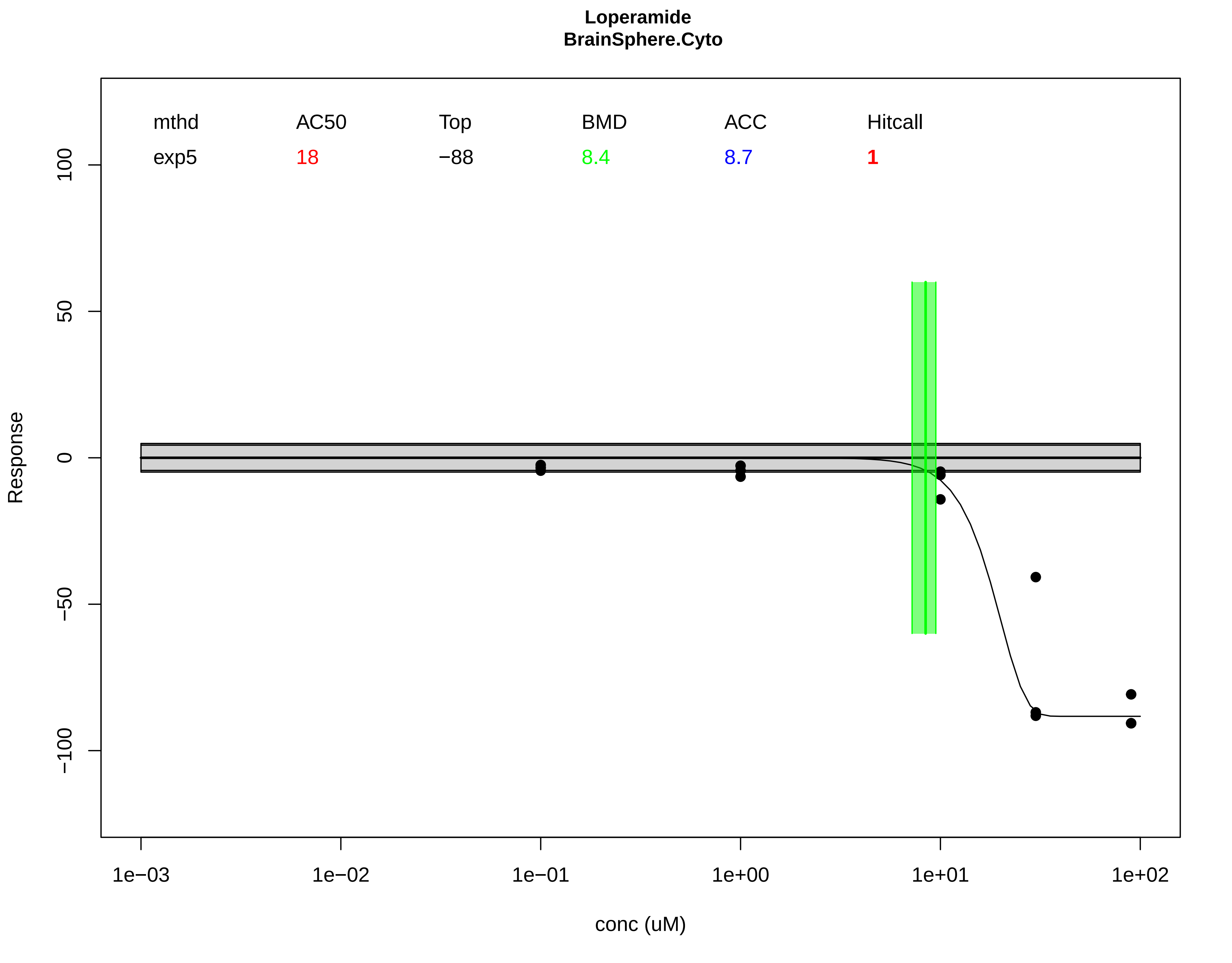

Loperamide
BrainSphere.Cyto
mthd
AC50
Top
BMD
ACC
Hitcall
exp5
18
−88
8.4
8.7
1
100
50
0
−50
−100
Response
1e−03
1e−02
1e−01
1e+00
1e+01
1e+02
conc (uM)

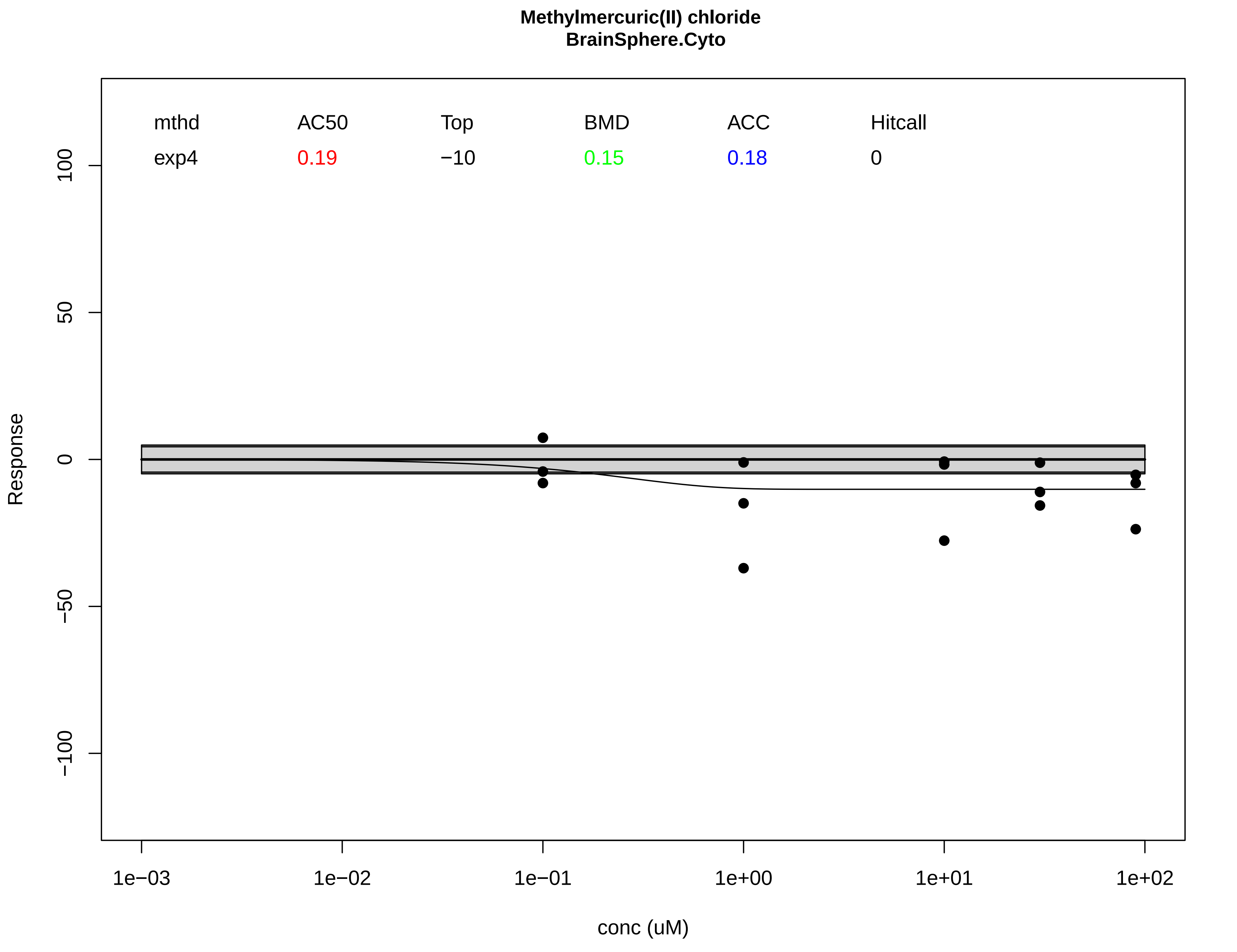

Methylmercuric(II) chloride
BrainSphere.Cyto
mthd
AC50
Top
BMD
ACC
Hitcall
exp4
0.19
−10
0.15
0.18
0
Response
100
50
0
−50
−100
1e−03
1e−02
1e−01
1e+00
1e+01
1e+02
conc (uM)

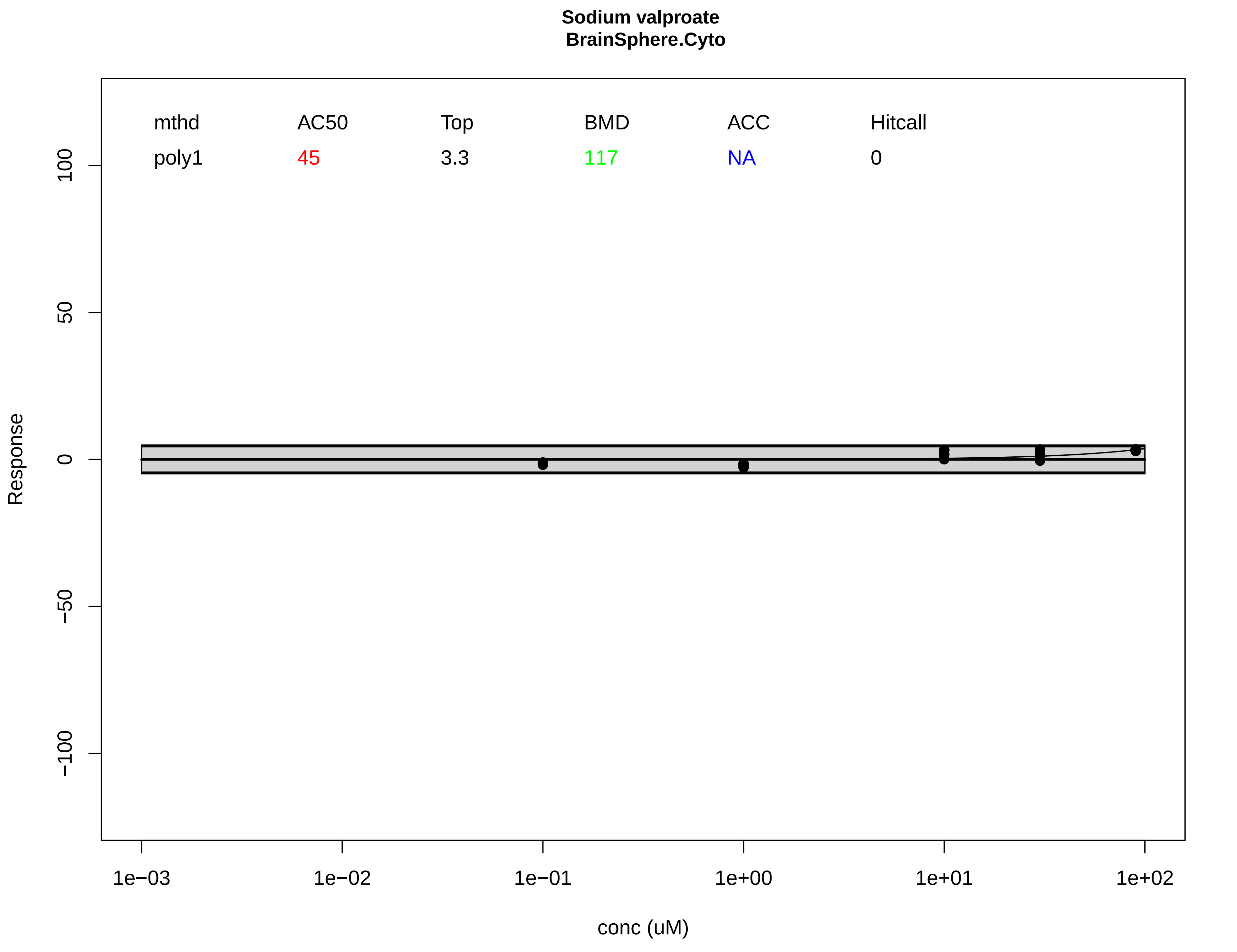
Sodium valproate
BrainSphere.Cyto
mthd
AC50
Top
BMD
ACC
Hitcall
poly1
45
3.3
117
NA
0
100
50
0
−50
−100
Response
1e−03
1e−02
1e−01
1e+00
1e+01
1e+02
conc (uM)
